# Synthetic small molecule GLP-1 secretagogues prepared by means of a three-component indole annulation strategy

**DOI:** 10.1038/srep28934

**Published:** 2016-06-29

**Authors:** Oleg G. Chepurny, Colin A. Leech, Martin Tomanik, Maria C. DiPoto, Hui Li, Xinping Han, Qinghe Meng, Robert N. Cooney, Jimmy Wu, George G. Holz

**Affiliations:** 1 Department of Medicine, State University of New York (SUNY), Upstate Medical University, Syracuse, New York, USA; 2 Department of Chemistry, Dartmouth College, Hanover, New Hampshire, USA; 3 Department of Surgery, State University of New York (SUNY), Upstate Medical University, Syracuse, New York, USA; 4 Department of Pharmacology, State University of New York (SUNY), Upstate Medical University, Syracuse, New York, USA

## Abstract

Rational assembly of small molecule libraries for purposes of drug discovery requires an efficient approach in which the synthesis of bioactive compounds is enabled so that numerous structurally related compounds of a similar basic formulation can be derived. Here, we describe (4 + 3) and (3 + 2) indole annulation strategies that quickly generate complex indole heterocycle libraries that contain novel cyclohepta- and cyclopenta[*b*]indoles, respectively. Screening of one such library comprised of these indoles identifies JWU-A021 to be an especially potent stimulator of glucagon-like peptide-1 (GLP-1) secretion *in vitro*. Surprisingly, JWU-A021 is also a potent stimulator of Ca^2+^ influx through TRPA1 cation channels (EC_50_ *ca*. 200 nM), thereby explaining its ability to stimulate GLP-1 release. Of additional importance, the available evidence indicates that JWU-A021 is one of the most potent non-electrophilic TRPA-1 channel agonists yet to be reported in the literature.

The indole heterocycle is among the most important nitrogen-containing heterocycles in both medicine and the broader spectrum of biologically active compounds. Indeed, it prominently occupies a spot in the top 10 most frequently occurring nitrogen heterocycles of FDA approved drugs in the US^1^. The significance of indole is further underscored by the fact that it is also the side-chain of L-tryptophan, one of only 21 proteinogenic amino acids found in eukaryotes. It is perhaps not surprising then that a subset of the indole motif, cycloalka[*b*]indoles, has garnered considerable attention from pharmaceutical and biotechnology companies as a promising pharmacophore for new drugs. In 2012, our group reported a novel (4 + 3) strategy for preparing cyclohepta[*b*]indoles in a single chemical transformation by means of a three-component annulation reaction beginning with an indole, a carbonyl, and a diene^2^. Because each of the starting materials can be independently varied, it was possible to quickly produce a large library of these compounds. Density functional theory (DFT) calculations suggested that the reaction proceeds through a step-wise mechanism, rather than a concerted, pericyclic 6πe^−^ process. No longer constrained by the Woodward-Hoffmann selection rules that govern pericyclic transformations, we then extended the methodology to include (3 + 2) annulation reactions in which the dienes were substituted by styrenyl substrates (total 4πe^−^) to furnish cyclopenta[*b*]indoles.

These theoretical considerations prompted us to investigate if cyclohepta[*b*]indole libraries could be screened to allow the identification of small molecules with biologically significant properties. To achieve proof of concept, we evaluated the capacity of cyclohepta[*b*]indoles to act as glucagon-like peptide-1 (GLP-1) secretagogues. GLP-1 is synthesized and secreted from intestinal L-cells, and it is the prototype of a new class of blood glucose-lowering agents that are now in use for the treatment of type 2 diabetes mellitus (T2DM)^3^. In this manuscript we report: **1)** for the first time our findings on the (3 + 2) methodology, **2)** expanded substrate scope for the (4 + 3) annulation, **3)** the identification of one of these compounds, JWU-A021, as a potent GLP-1 secretagogue, and **4)** experimental evidence indicating that JWU-A021 likely operates in the intestinal L-cells by elevating the intracellular [Ca^2+^] by means of activating the Transient Receptor Potential Ankyrin 1 channel (TRPA1)^4^.

## Results

### Identification of cyclohepta[*b*]indoles with GLP-1 releasing actions

**Figure 1:**
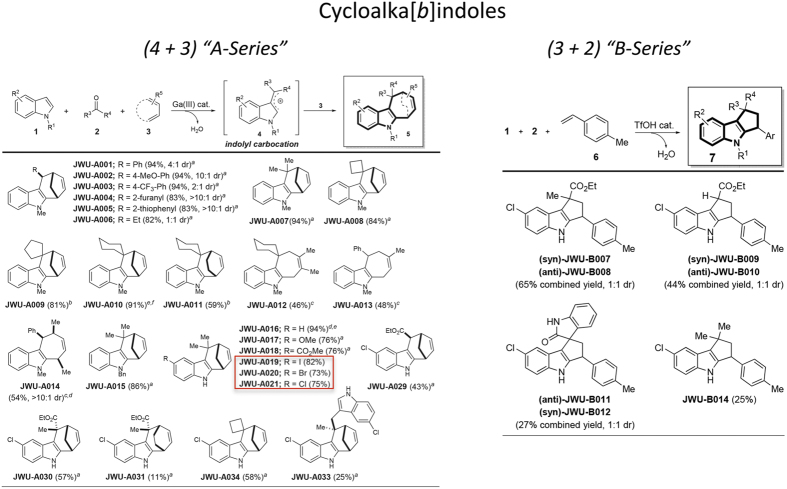
Cyclohepta[*b*]indole synthesis by (4 + 3) and (3 + 2) cycloaddition reactions. (**Left panel**) “A-Series” heterocyclics generated by (4 + 3) cycloaddition reactions. ^a^indole (1 equiv), carbonyl (2 equiv), diene (5 equiv), GaBr_3_ (10 mol%), rt. ^b^indole (1 equiv), carbonyl (2 equiv), diene (5 equiv), Ga(OTf)_3_ (10 mol%), rt. ^c^indole (1 equiv), carbonyl (1.1 equiv), diene (5 equiv), Ga(OTf)_3_ (20 mol%), rt. ^d^Single-crystal X-ray analysis. ^e^2 mmol scale. (**Right panel**) “B-Series” heterocyclics generated by (3 + 2) cycloaddition reactions. ^a^indole (1 equiv), carbonyl (2 equiv), diene (1.5 equiv), TfOH (20 mol%), rt.

**Figure 2:**
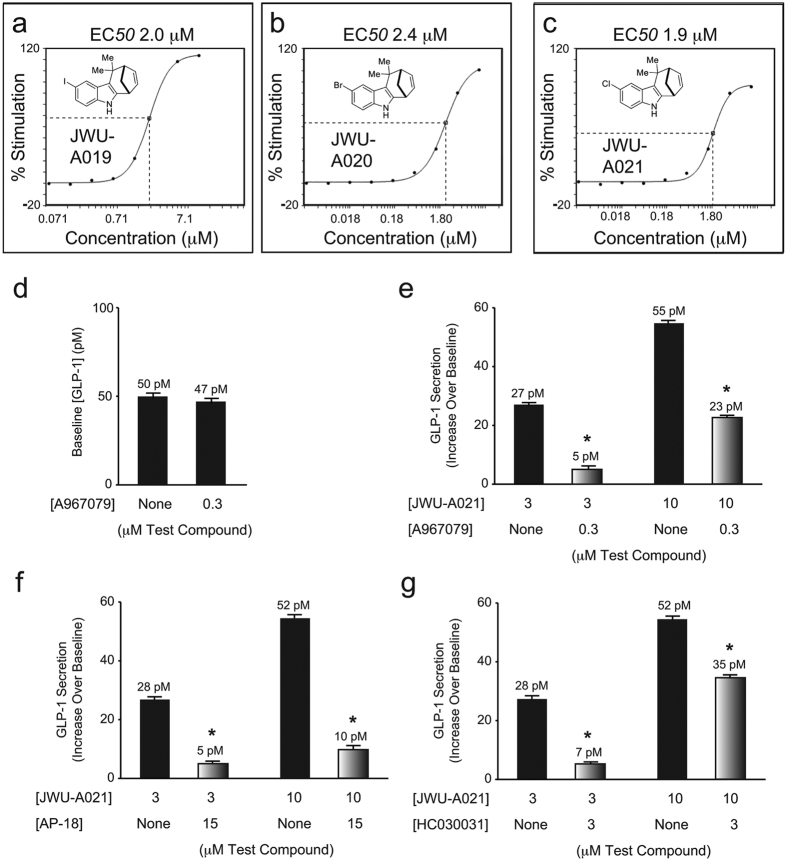
Cyclohepta[*b*]indole-stimulated GLP-1 release: effects of TRPA1 channel blockers. (**a–c**) JWU-A019, JWU-A020, and JWU-A021 stimulated GLP-1 secretion from STC-1 cells. (**d**) Basal GLP-1 secretion was not significantly altered by A967079. (**e–g**) GLP-1 secretion stimulated by JWU-A021 was reduced by A967079, AP-18, and HC030031. Data are the mean + s.d. of *N = 3* independent experiments (*p < 0.05; paired *t* test).

**Figure 3:**
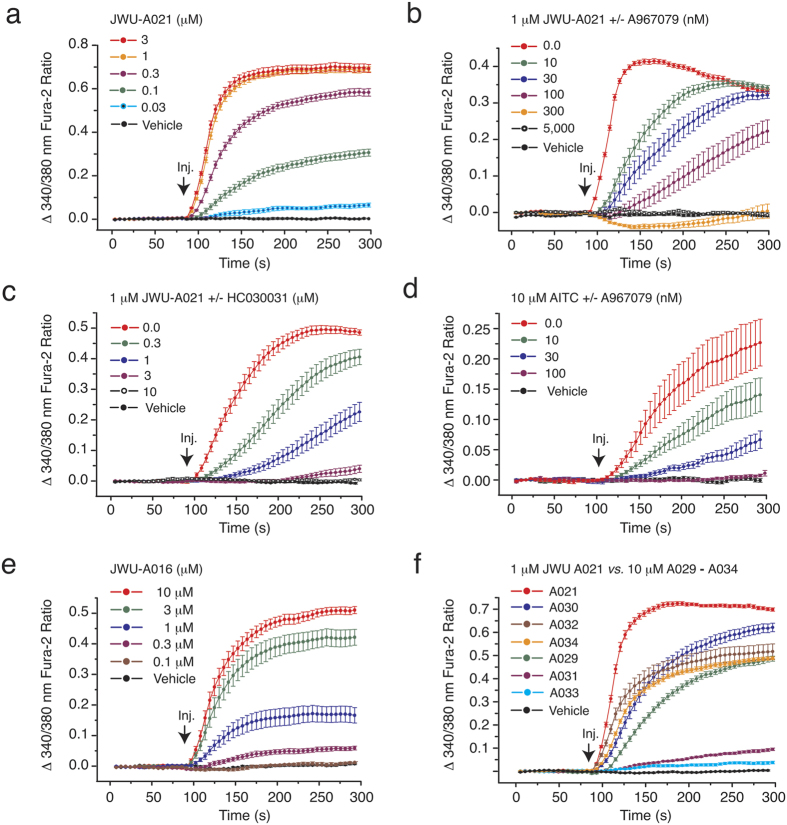
Actions of JWU-A021 and other A-series compounds to increase [Ca^2+^]_i_. (**a**) Fura-2 assays of STC-1 cell monolayers demonstrated the concentration-dependent action of JWU-A021 to increase [Ca^2+^]_i_. (**b,c**) A967079 and HC030031 each exerted concentration-dependent actions to counteract the stimulatory effect of JWU-A021 (1 μM) in STC-1 cells. (**d**) The TRPA1 channel activator AITC (10 μM) increased [Ca^2+^]_i_, and this action of AITC was also reduced by A967079 and HC030031 in STC-1 cells. For these panels and subsequent figures, JWU-A021 was administered by bolus injection (Inj.). (**e,f**) Ca^2+^-elevating actions of JWU-A016 (**e**) and the A029-A034 series of test agents (**f**). For all examples depicted here, the findings are representative of a single experiment repeated a minimum of five times on five different occasions with similar results.

Using a three-component (4 + 3) annulation methodology developed by one of our groups (see Supplemental Material)^2^, we generated a library of cyclohepta[*b*]indoles (Fig. 1, left panel). These products were screened for their capacity to stimulate GLP-1 release from mouse STC-1 intestinal enteroendocrine cells^5^,^6^,^7^. Three cyclohepta[*b*]indoles with GLP-1 releasing properties were identified, each of which contain either I, Br, or Cl substitutions that append an identical core structure. These “A-Series” compounds include JWU-A019, JWU-A020, and JWU-A021 (red box, Fig. 1, left panel), each of which dose-dependently stimulated GLP-1 release by ca. 2-fold over baseline (Fig. 2a–c), with JWU-A021 being the most potent (EC_50_ 1.9 μM). Surprisingly, the GLP-1 secretagogue action of JWU-A021 was reduced by the selective TRPA1 cation channel blockers A967079, AP-18, and HC030031 (Fig. 2d–g). This finding indicated that JWU-A021 might be a TRPA1 cation channel activator. In fact, JWU-A021 raised the [Ca^2+^]_i_ in fura-2 loaded STC-1 cell monolayers (Fig. 3a). Furthermore, this Ca^2+^-elevating action of JWU-A021 (EC_50_ ca. 200 nM) was abrogated by the TRPA1 channel blockers A967079 and HC030031 (Fig. 3b,c). Consistent with prior reports that TRPA1 channels are expressed in STC-1 cells^8^,^9^, and that TRPA1 channel activation leads to GLP-1 secretion from this cell line^9^, the established TRPA1 channel activator allyl isothiocyanate (AITC) raised the [Ca^2+^]_i_, and this effect was abrogated by A967079 (Fig. 3d). Since an increase of [Ca^2+^]_i_ triggers GLP-1 release from intestinal L-cells^10^, and since L-cells express TRPA1 channels^9^, these findings indicated that JWU-A021 might serve as the prototype of a new class of small molecule GLP-1 secretagogues with TRPA1 channel activating properties.

### Structure-function properties of the A-Series of cyclohepta[*b*]indoles

**Figure 4:**
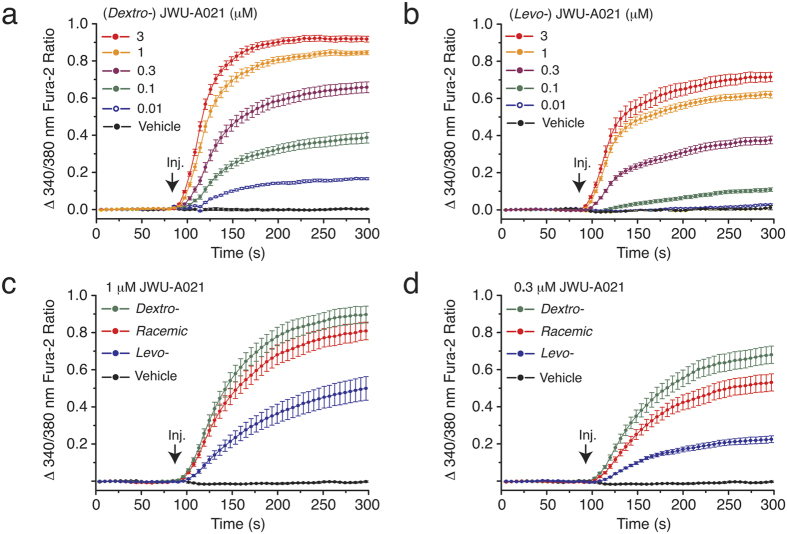
Differential actions of JWU-A021 enantiomers to increase [Ca^2+^]_i_. (**a,b**) The dextrorotatory (*Dextro*-) enantiomer (+)-(6*R*,9*S*)-JWU-A021 exerted a more powerful stimulatory effect in comparison to the levorotatory (*Levo*-) enantiomer (+)-(6*S*,9*R*)-JWU-A021 when it was tested in the fura-2 assay using monolayers of STC-1 cells. (**c,d**) Differential stimulation of an increase of [Ca^2+^]_i_ by the dextrorotatory, levorotatory, and racemic forms of either 1 μM or 0.3 μM JWU-A021. For all examples depicted here, the findings are representative of a single experiment that was repeated a minimum of three times on three different occasions with similar results.

Although JWU-A021 contains a chlorine substitution (Fig. 1, left panel), this halogenation was not essential to its biological activity since the non-halogenated indole JWU-A016 also exerted a dose-dependent stimulatory effect to raise the [Ca^2+^]_i_ (Fig. 3e). Other compounds of the A-series designated as JWU A029 through A034 (see Fig. 1, left panel) were considerably less potent (EC_50_ > 3 μM) in this assay (Fig. 3f) despite their structural similarity to JWU-A021. Furthermore, for JWU-A021, the dextrorotatory enantiomer (+)-(6*R*,9*S*)-JWU-A021^11^ stimulated a larger increase of [Ca^2+^]_i_ in comparison to the levorotatory enantiomer (−)-(6*S*,9*R*)-JWU-A021^11^ (Fig. 4a,b), whereas racemic JWU-A021 exerted a stimulatory effect that was intermediate between that of the dextrorotatory and levorotatory enantiomers (Fig. 4c,d).

The A-Series of cyclohepta[*b*]indoles derived by the (4 + 3) annulation methodology (Fig. 1, left panel) were then compared with a B-series of cyclopenta[*b*]indoles derived by a (3 + 2) annulation methodology (see Fig. 1, right panel). These B-series compounds (JWU B007 through B014) were obtained by modifying the annulation reaction so that the Lewis acid catalyst GaBr_3_ was replaced with 20 mol% of TfOH (see Supplemental Material). When the B-series compounds were evaluated in the fura-2 assay using STC-1 cells, they were established to be especially weak Ca^2+^ elevating agents such that a test concentration of 10 μM was necessary to detect any increase of [Ca^2+^]_i_ (data not shown). In summary, these findings demonstrated that there existed a clear structural specificity for cycloalka[*b*]indoles as Ca^2+^-elevating agents in STC-1 cells. The A-Series cyclohepta[*b*]indole JWU-A021 was bioactive at nM concentrations, whereas all B-series cyclopenta[*b*]indoles were ineffective.

### JWU-A021 activates TRPA1 channels to promote Ca^2+^ influx

**Figure 5:**
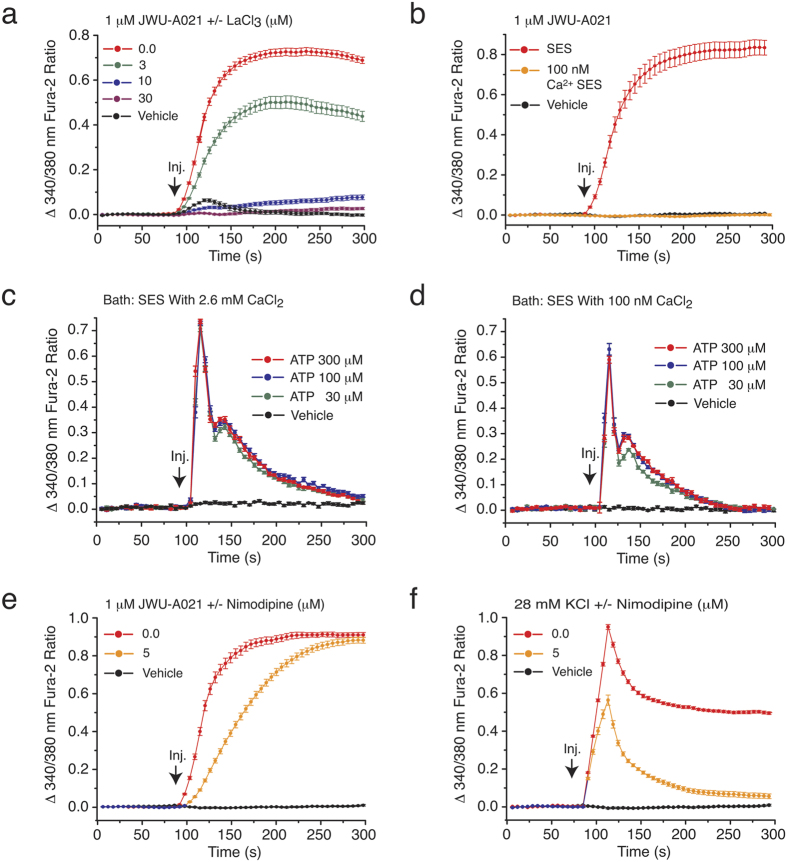
JWU-A021 promotes Ca^2+^ influx rather than Ca^2+^ mobilization. (**a**) The Ca^2+^ channel blocker La^3+^ exerted a concentration-dependent action to abrogate the increase of [Ca^2+^]_i_ stimulated by JWU-A021 (1 μM). (**b**) The action of JWU-A021 (1 μM) to increase [Ca^2+^]_i_ was abrogated when the Ca^2+^ concentration of the SES was reduced to 100 nM. (**c,d**) ATP dose-dependently increased the [Ca^2+^]_i_ measured under conditions in which the SES contained either 2.6 mM CaCl_2_ (**c**) or 100 nM CaCl_2_ (**d**). (**e**) The L-type Ca^2+^ channel blocker nimodipine (5 μM) slowed the rate of onset but failed to reduce the end-point increase of [Ca^2+^]_i_ measured in response to JWU-A021 (1 μM). (**f**) The effectiveness of nimodipine (5 μM) as an inhibitor of Ca^2+^ influx was demonstrated by its ability to fully block the end-point increase of [Ca^2+^]_i_ measured in response to 28 mM KCl-induced depolarization. For all examples depicted here, the findings are representative of a single experiment repeated a minimum of three times on three different occasions with similar results.

We next sought to identify the molecular target that mediates the action of JWU-A021 to stimulate an increase of [Ca^2+^]_i_ in STC-1 cells. Initially, we found that the Ca^2+^-elevating action of JWU-A021 was blocked by inclusion of La^3+^ in the standard extracellular saline (SES) (Fig. 5a). This finding is significant because La^3+^ blocks Ca^2+^ entry through non-selective cation channels (NSCCs) and voltage-dependent Ca^2+^ channels (VDCCs)^4^,^12^. We also found that JWU-A021 failed to stimulate an increase of the [Ca^2+^]_i_ when the extracellular [Ca^2+^] was lowered from 2.6 mM to 100 nM (Fig. 5b). This finding is expected if JWU-A021 promotes Ca^2+^ influx rather than Ca^2+^ mobilization from intracellular Ca^2+^ stores. Since it might be argued that Ca^2+^ stores were simply depleted under conditions in which the SES contained 100 nM Ca^2+^, we compared the Ca^2+^ mobilizing properties of a purinergic receptor agonist (ATP) under conditions in which the SES contained 2.6 mM Ca^2+^ or 100 nM Ca^2+^. This control experiment revealed that ATP retained its ability to mobilize Ca^2+^ even when the extracellular Ca^2+^ was set to 100 nM (c.f., Fig. 5c,d). Thus, a depletion of Ca^2+^ stores did not explain why JWU-A021 failed to raise the [Ca^2+^]_i_ when the SES contained 100 nM Ca^2+^. Instead, the SES containing 100 nM Ca^2+^ failed to support the action of JWU-A021 to promote Ca^2+^ influx.

Ca^2+^ influx stimulated by JWU-A021 might result solely from TRPA1 channel activation since GLP-1 secretion stimulated by JWU-A021 was reduced by TRPA1 channel blockers (Fig. 2d–g). In fact, TRPA1 channels are NSCCs that allow Ca^2+^ permeation^4^. However, it is important to take into account the possibility that binding of JWU-A021 to TRPA1 channels leads to membrane depolarization and action potential generation, thereby stimulating additional Ca^2+^ influx through VDCCs. Therefore, a systematic analysis was performed to evaluate potential effects of selective VDCC blockers^12^. Nimodipine, a blocker of L-type VDCCs, slowed the initial increase of [Ca^2+^]_i_ measured in response to JWU-A021 (Fig. 5e), but the sustained increase of [Ca^2+^]_i_ occurring at the assay’s end-point was unaffected (Fig. 5e). As a positive control, we confirmed the ability of nimodipine to suppress the increase of [Ca^2+^]_i_ that resulted from 28 mM KCl-induced membrane depolarization (Fig. 5f). Thus, L-type VDCCs played a minor role as mediators of sustained Ca^2+^ influx stimulated by JWU-A021. Since the selective TRPA1 channels blockers A967079 and HC030031 fully abolished all Ca^2+^-elevating actions of JWU-A021 (Fig. 3b,c), the increase of [Ca^2+^]_i_ measured at the end-point was instead explained by TRPA1 channel activation in response to JWU-A021.

**Figure 6:**
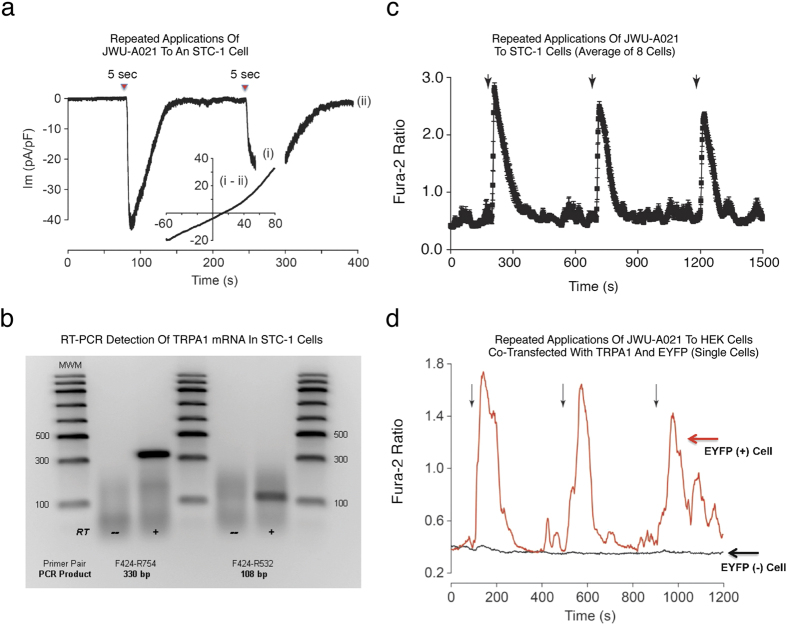
Membrane currents and Ca^2+^ transients activated by JWU-A021. (**a**) Whole-cell patch clamp analysis (V_h_ −60 mV) demonstrated inward membrane currents activated by repeated 5 sec focal applications of JWU-A021 (3 μM; red triangles) to a single STC-1 cell. The inset provides a current-voltage (I-V) relationship for the current activated by JWU-A021 (I_m_, membrane current in pA normalized to membrane capacitance in pF). It is the difference current obtained by subtracting the IV relationships measured during (time point “i”) and after recovery (time point “ii”) of the response. Findings are representative of a single patch clamp experiment that was repeated with similar results using *N = 10* cells. (**b**) RT-PCR validation that STC-1 cells express TRPA1 channel mRNA, as detected using two different primer pairs (RT, reverse transcriptase; MWM, molecular weight markers). Findings are representative of a single experiment repeated twice with similar results. (**c**) Averaged Ca^2+^ transients obtained from STC-1 cells stimulated by focal application (arrows) of JWU-A021 (3 μM) to *N = 8* cells. (**d**) Ca^2+^ transients stimulated by focal application (arrows) of JWU-A021 (3 μM) to a single HEK-293 cell transfected with rat TRPA1 cDNA fused to EYFP cDNA (red trace), or a HEK-293 cell transfected with EYFP cDNA but not rat TRPA1 cDNA (black trace). EYFP fluorescence was used as a marker to positively identify cells that were transfected so that fura-2 based assays of [Ca^2+^]_i_ could be performed using these cells. Findings are representative of a single experiment repeated a minimum of three times on three different occasions with similar results.

We also tested T-type (kurtoxin, mibefradil), N-type (omega-conotoxin GVIA), and P/Q-type (omega-agatoxin IVA) blockers of VDCCs. None of these agents altered the end-point increase of [Ca^2+^]_i_ measured in response to JWU-A021 (data not shown). Using whole-cell patch clamp analysis in the voltage-clamp mode, we then demonstrated that brief focal application of JWU-A021 to STC-1 cells led to the appearance of an inward current when the membrane potential was set to −60 mV (Fig. 6a). By using a ramp stimulus protocol to vary the holding potential, it was established that the current activated by JWU-A021 exhibited outward rectification (Fig. 6a, inset), as is expected for TRPA1 channels^4^. Using RT-PCR analysis, the expression of TRPA1 mRNA in STC-1 cells was confirmed (Fig. 6b). Finally, live-cell imaging demonstrated reversible actions of JWU-A021 to raise the [Ca^2+^]_i_ in STC-1 cells (Fig. 6c). This was also the case for HEK-293 cells transfected with a rat TRPA1 cDNA (Fig. 6d). Thus, target validation was achieved in which JWU-A021 was established to be a TRPA1 channel activator.

### JWU-A021 retains its ability to activate mutant C622S TRPA1

**Figure 7:**
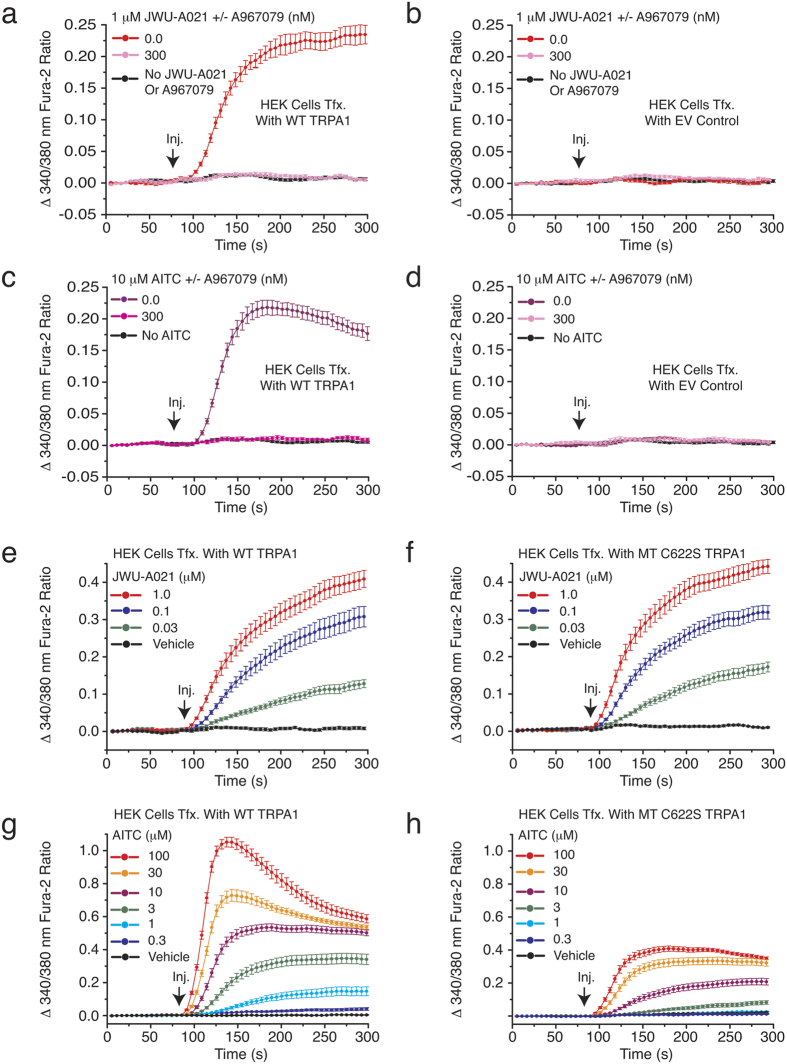
Studies with HEK-293 cells transfected with recombinant TRPA1. (**a,b**) HEK-293 cell monolayers transfected with wild-type (WT) rat TRPA1 cDNA, but not a negative control empty vector (EV), exhibited an increase of [Ca^2+^]_i_ in response to JWU-A021 (1 μM), and this action of JWU-A021 was abrogated by the TRPA1 channel blocker A967079. (**c,d**) HEK-293 cells transfected with WT rat TRPA1 cDNA, but not a negative control EV, exhibited an increase of [Ca^2+^]_i_ in response to AITC (10 μM), and this action of AITC was abrogated by the TRPA1 channel blocker A967079. This experiment confirmed the expected failure of HEK-293 cells to express endogenous TRPA1 channels. (**e**) A concentration-dependent action of JWU-A021 to increase [Ca^2+^]_i_ was measured in HEK-293 cell monolayers transfected with wild-type (WT) rat TRPA1 cDNA. (**f**) HEK-293 cell monolayers transfected with mutant C622S rat TRPA1 cDNA responded to JWU-A021 in a manner nearly identical to that of cells transfected with WT TRPA1 (compare panels e,f). Thus, the non-electrophile JWU-A021 acted independently of C622 covalent modification. (**g,h**) The TRPA1 activator AITC stimulated an increase of [Ca^2+^]_i_ in HEK-293 cell monolayers transfected with WT rat TRPA1, and this action of AITC to increase [Ca^2+^]_i_ was greatly diminished in HEK-293 cells transfected with mutant C622S TRPA1 cDNA (compare panels g,h). Thus, the electrophile AITC must covalently modify C622 in order to fully activate the channel. For all examples depicted here, the findings are representative of a single experiment repeated a minimum of three times on three different occasions with similar results.

Electrophiles such as AITC activate TRPA1 channels by covalently modifying cysteine residues located near the cytosolic N-terminus of the channel^4^,^13^. Although the structure of JWU-A021 indicates that it is unlikely to act as an electrophile, we sought experimental evidence that this is the case. Thus, the action of JWU-A021 was evaluated in HEK-293 cells transfected with a wild-type (WT) TRPA1, an empty vector (EV), or a mutant (MT) C622S TRPA1 channel that has reduced sensitivity to electrophiles^14^. For cells transfected with the WT TRPA1 channel, JWU-A021 stimulated an increase of [Ca^2+^]_i_, and this effect was blocked by A967079 (Fig. 7a). However, for cells transfected with the EV, there was no effect of JWU-A021 (Fig. 7b). As a control, we verified that AITC also increased the [Ca^2+^]_i_ in cells transfected with the WT TRPA1 channel, but not the EV (Fig. 7c,d), and that this effect of AITC was inhibited by A967079 (Fig. 7c).

Further analysis revealed that the C622S TRPA1 channel was activated by JWU-A021 in a manner nearly identical to that of the WT (Fig. 7e,f). However, the C622S TRPA1 channel responded poorly to AITC (Fig. 7g,h). The reduced AITC sensitivity of the mutant channel is expected since the cysteine 622 residue that is implicated in the control of channel activity, by covalent modification with AITC, is missing in the mutant channel^14^. Collectively, such findings provide support for a model in which JWU-A021 acts independently of covalent cysteine modification to activate TRPA1 channels. This model is supported by our single cell imaging studies in which it was demonstrated that the Ca^2+^-elevating action of JWU-A021 was repeatable and rapidly reversible following wash out of JWU-A021 (Fig. 6c,d). Thus, JWU-A021 might activate TRPA1 channels through reversible binding to a receptor that corresponds to the channel, itself.

### JWU-A021 stimulates GLP-1 release from primary intestinal cell cultures

**Figure 8:**
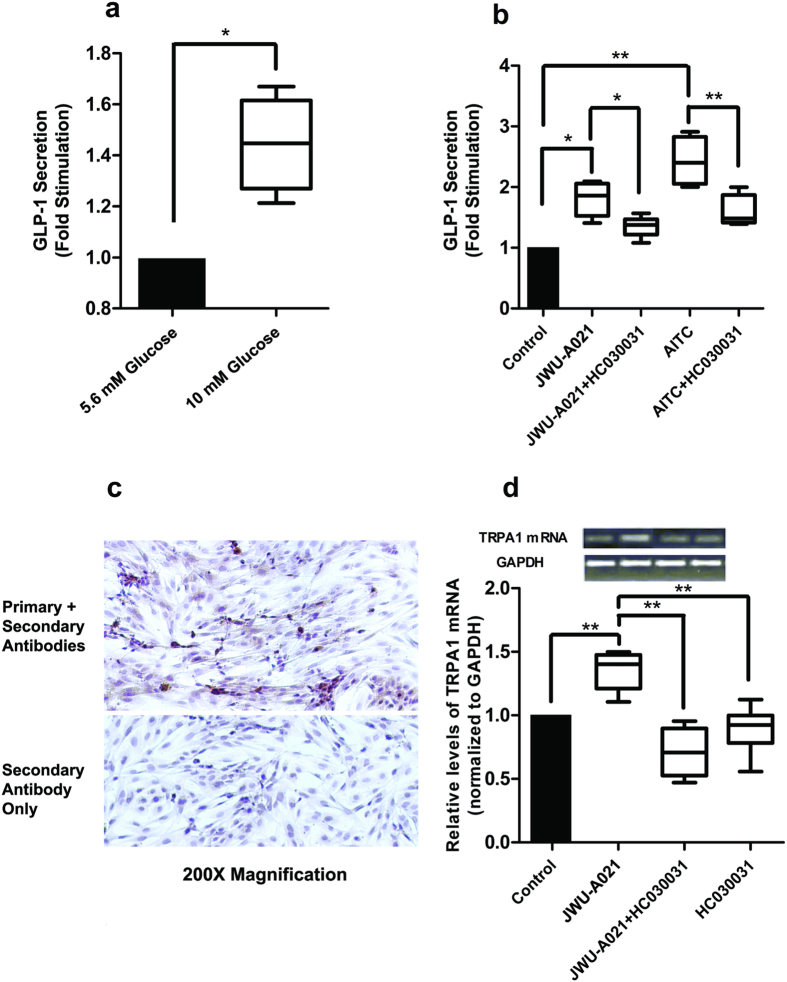
JWU-A021 stimulates GLP-1 release from mouse intestinal cells. (**a**) Primary cultures were stimulated for 30 min using serum-free DMEM assay buffer containing either 5.6 or 10 mM glucose so that glucose-stimulated GLP-1 secretion could be measured. Data are the mean + s.d. of 4 independent assays (*p < 0.05; paired *t* test) and are expressed as the fold-stimulation of GLP-1 release, so that a value of 1.0 corresponds to GLP-1 release measured for buffer containing 5.6 mM glucose. (**b**) HC030031 (10 μM) inhibited the actions of JWU-A021 (3 μM) and AITC (100 μM) to stimulate GLP-1 secretion from primary cultures. HC030031 was administered 15 minutes prior to addition of JWU-A021 or AITC, and it was also present during the 30 minutes test interval during which cells were exposed to JWU-A021 or AITC dissolved in serum free DMEM assay buffer containing 5.6 mM glucose. Data are the mean + s.d. of 4–6 independent assays (*p < 0.05; **p < 0.01; ANOVA with Bonferroni post test). (**c**) Immunocytochemical detection of GLP-1 in primary cell cultures. The top panel illustrates specific GLP-1 immunoreactivity (brown), as detected using the anti-GLP-1 monoclonal primary antibody in combination with an HRP conjugated secondary antiserum. The bottom panel illustrates negative control non-specific labeling obtained when using the secondary antiserum only. (**d**) qRT-PCR analysis demonstrated that JWU-A021 (3 μM) increased the relative abundance of TRPA1 channel mRNA in primary cell cultures, and that this effect was reduced by HC030031 (10 μM). For this analysis, cultures were maintained for 30 minutes in serum-free DMEM assay buffer containing 5.6 mM glucose and the test compounds. Data are the mean + s.d. of 6 independent assays (**p < 0.01; ANOVA with Bonferroni post test). The top inset illustrates qRT-PCR products detected by agarose gel electrophoresis.

The GLP-1 secretagogue action of JWU-A021 was also tested using mouse intestine primary cell cultures enriched in L-cells. These cultures exhibited glucose-stimulated GLP-1 release (Fig. 8a), and they also released GLP-1 in response to JWU-A021 and AITC (Fig. 8b). Furthermore, the GLP-1 secretagogue actions of JWU-A021 and AITC were inhibited by the TRPA1 channel blocker HC030031 (Fig. 8b). Immunocytochemical analysis using a GLP-1 specific monoclonal antibody in combination with a horseradish peroxidase (HRP) conjugated secondary antiserum revealed that ca. 20% of the cells comprising these cultures contained GLP-1 (Fig. 8c, top panel). However, a negative control demonstrated that GLP-1 immunoreactivity was not measurable using the secondary antiserum alone (Fig. 8c, bottom panel). Interestingly, quantitative reverse transcriptase polymerase chain reaction (qRT-PCR) analysis revealed that JWU-A021 significantly increased levels of TRPA1 channel mRNA by ca. 40% in these cultures (Fig. 8d). Furthermore, this action of JWU-A021 was inhibited by HC030031 (Fig. 8d). Thus, Ca^2+^ entry through TRPA1 channels seems to exert a positive feedback effect on TRPA1 channel mRNA expression. Collectively, such findings are in agreement with the report of Emery and co-workers that TRPA1 channel activation by AITC leads to GLP-1 release from mouse L-cells^9^. In summary, the new method of cycloalka[*b*]indole library construction reported here has identified JWU-A021 to be a GLP-1 secretagogue with potent TRPA1 activating properties, not only in STC-1 cells, but also in mouse L-cells.

## Discussion

Rational assembly of small molecule libraries for purposes of drug discovery requires an efficient approach in which the synthesis of bioactive compounds is enabled so that numerous structurally related compounds of a similar basic formulation can be derived. Here, we describe (4 + 3) and (3 + 2) annulation strategies that quickly generate complex indole heterocycle libraries that contain novel cyclohepta and cyclopenta [*b*]indoles, respectively. We demonstrate that these indole heterocycle libraries are amenable to screening so that new GLP-1 secretagogues can be identified. Thus, the primary outcome of this study is that the cyclohepta[*b*]indole JWU-A021 is revealed to be a novel stimulator of GLP-1 release from STC-1 cells and mouse intestinal L-cells. These findings are of potential medical importance due to the fact that small molecule GLP-1 secretagogues are now under investigation for use in the treatment of T2DM^3^.

It is intriguing that JWU-A021 exerts its GLP-1 secretagogue action by stimulating Ca^2+^ influx through TRPA1 channels. This finding expands on the already established role of TRPA1 channels in peripheral sensory neuron function^13^, and it is consistent with the new view that members of the *Trp* ion channel family participate in the control of multiple intestinal cell functions^15^,^16^. Since Ca^2+^-dependent exocytosis of GLP-1 from L-cells is stimulated by ingested nutrients^17^,^18^,^19^, orally administered JWU-A021 might activate intestinal TRPA1 channels so that it replicates the GLP-1 secretagogue effect of such nutrients. If so, synthetic small molecule TRPA1 channel activators might constitute a new class of blood glucose-lowering agents. This general line of thinking is consistent with an emerging field of investigation in which TRPA1 channel-targeted drug discovery is applied for disease processes unrelated to sensory neuron function^20^.

When considering a possible use of JWU-A021 in therapeutics, it is noteworthy that the TRPA1 channel activator cinnamaldehyde exerts blood glucose-lowering and weight-reducing actions in mice^21^. However, cinnamaldehyde is an electrophile that is not a specific TRPA1 channel activator owing to its promiscuous alkylating properties. JWU-A021 avoids such non-specificity since it is not an electrophile and most likely acts independently of covalent TRPA1 channel modification. While a few non-electrophilic, non-covalent TRPA1 activators are reported (e.g., carvacrol)^4^, only one (PF-4840154) is especially potent^22^. Unfortunately, PF-4840154 is a hERG K^+^ channel inhibitor and it has the predicted adverse side effect to induce cardiac arrhythmia^22^.

One prior *in vivo* study failed to detect elevated levels of GLP-1 in the blood after oral administration of the TRPA1 channels activators cinnamaldehyde and methyl syringate to mice^23^. However, levels of the intestinal hormone peptide YY (PYY) were elevated in the blood, and this effect was suppressed by a TRPA1 channel blocker^23^. Since GLP-1 and PYY are co-secreted from L-cells^24^, the failure of cinnamaldehyde and methyl syringate to raise circulating levels of GLP-1 seems paradoxical. However, detection of circulating GLP-1 is complicated owing to its rapid degradation and inactivation by dipeptidylpeptidase-4 (DPP-4), as well as its quick clearance from the systemic circulation^25^. Ideally, quantification of secreted GLP-1 must be based on its concentration immediately at the site of its release in the intestine where it exerts a local effect to activate vagal sensory neurons within the intestinal wall so that vasovagal reflexes important to global metabolic homeostasis can be initiated^26^,^27^. Although prior studies were performed using mice, a rationale exists for future studies examining TRPA1 channel-dependent regulation of GLP-1 secretion from human L-cells.

Although not a focus of the report here, we recently found that JWU-A021 failed to stimulate GLP-1 release from GLUTag cells, a mouse L-cell line^28^. Furthermore, JWU-A021 failed to increase the [Ca^2+^]_i_ in GLUTag cells, whereas AITC exerted only a weak effect (data not shown). Such findings are consistent with one prior report documenting low level expression of TRPA1 mRNA in GLUTag cells, whereas higher levels exist in STC-1 cells and mouse L-cells^9^. Evidently, GLUTag cells do not recapitulate the TRPA1 channel expression that is characteristic of mouse L-cells. For these reasons, species-specific and cell line-specific actions of TRPA1 channel activators such as JWU-A021 must be taken into account when planning high throughput screening approaches targeted at the identification of new GLP-1 secretagogues.

## Conclusion

Summarized here are (4 + 3) and (3 + 2) annulation strategies that we believe will be generally applicable to the synthesis of small molecule cycloalka[*b*]indole libraries that are useful for drug screening purposes. The feasibility of this approach is established herein by demonstrating the rapid synthesis, purification, identification, and characterization of cyclohepta[*b*]indoles with potent GLP-1 releasing properties. The power of this approach is further emphasized in that the (4 + 3) annulation strategy is revealed to be an effective means with which to generate novel TRPA1 channel activators. Therefore, findings presented here validate a new strategy for small molecule combinatorial library construction. Although *in vivo* testing to determine the safety and efficacy of JWU-A021 still remains to be achieved, cyclohepta[*b*]indoles based on the structure of JWU-A021 might find a role in therapeutics as a new class of GLP-1 secretagogues.

## Methods

### STC-1 and HEK-293 cell culture

STC-1 and HEK-293 cells were obtained from the ATCC (Manassas, VA). Culture medium was comprised of Dulbecco’s Modified Eagle Medium (DMEM) containing 25 mM glucose, 10% fetal bovine serum (FBS), 100 units ml^−1^ penicillin G, and 100 μg/ml streptomycin. Cultures were passaged once a week while maintained at 37 °C in a humidified incubator gassed with 5% CO_2_. Cultures harvested by trypsinization were plated at a density of 40,000–50,000 cells per well on rat tail collagen (RTC) coated 96-well Costar 3904 plates two days prior to each experiment. Cultures were 85–95% confluent on the day of the experiment. Culture media, additives, Costar plates, and RTC were from Gibco/Thermo Fisher Scientific (Waltham, MA).

### Fura-2 based assays of [Ca^2+^]_i_

The fura-2 loading solution for all assays was comprised of a standard extracellular solution (SES; 295 milliosmoles/L) containing (in mM): 138 NaCl, 5.6 KCl, 2.6 CaCl_2_, 1.2 MgCl_2_, 10 HEPES (adjusted to pH 7.4 with NaOH), and supplemented with 11.1 mM glucose, 20 μl ml^−1^ FBS, 1 μl ml^−1^ Pluronic F-127, and 1 μM fura-2 acetoxymethyl ester (fura 2-AM; Thermo Fisher Sci.)^29^. Measurements of [Ca^2+^]_i_ from monolayers of fura-2 loaded cells were performed at 25 °C using a FlexStation 3 microplate reader under the control of SoftMaxPro v5.4 software (Molecular Devices, Sunnyvale, CA)^29^. Spectrofluorimetry was performed using excitation light at 335/9 and 375/9 nm (center/bandpass wavelengths) delivered using a 455 nm dichroic mirror. Emitted light was detected at 505/15 nm and the ratio of emission light intensities due to excitation at 335 and 375 nm was calculated. Raw data were exported to Origin v7.5 (Origin Lab., Northhampton, MA) for processing. Fura-2 based single cell meaurements of [Ca^2+^]_i_ were performed with minor modifications, as described previously^30^,^31^,^32^.

### Patch clamp electrophysiology

Membrane currents were recorded in the whole-cell configuration under conditions of voltage clamp in which cells were bathed in SES^33^,^34^. The patch pipette solution contained (in mM): 140 Cs-glutamate, 10 NaCl, 1 MgCl_2_, 0.2 EGTA, 2 MgATP, and 5 HEPES adjusted to pH 7.4 with CsOH. Test compounds were added to SES as indicated in the text. Patch pipettes were pulled from thin walled glass capillaries (G85150T-4, Warner Instruments, Hamden, CT) using a P-97 pipette puller (Sutter Inst., Novato, CA) and had resistances of 2–5 MΩ when filled with the pipette solution. Measurements of membrane currents were obtained using an EPC-9 amplifier controlled using PatchMaster software (HEKA Electronik, Lambrecht/Pfalz, Germany). Test solutions containing JWU-A021 were applied to single cells from a puffer pipette using a PicoSpritzer III pressure ejection system (Parker Hannifin, Hollis, NH). TRPA1 current-voltage relationships were obtained by subtracting the background membrane current from the activated TRPA1 current under conditions in which there was a 1 V/s shift of the holding potential, applied as a linear voltage ramp. Whole-cell currents were digitally sampled at a frequency of 10 kHz after filtering at 1–3 kHz. Current amplitudes were normalized to cell capacitance.

### Expression of recombinant TRPA1

JM109 competent *E. coli* (Promega, Madison, WI) were transformed with plasmid DNA, and antibiotic resistance selection was used to obtain single bacterial colonies expressing the indicated rat TRPA1 plasmid DNAs. These plasmids were isolated from bacteria using a HiSpeed Midi Kit (Qiagen, Valencia, CA). Transient transfection of HEK-293 cells with these plasmids was performed using Lipofectamine and Plus reagent according to the manufacturer’s protocol (Thermo Fisher Scientific).

### Sources of plasmids, channel blockers, and activators

A mutant rat C622S TRPA1 construct, and also the wild-type TRPA1 coding sequence (GenBank No. AY496961.1) fused at its 5′ terminus to the yellow fluorescent protein (YFP), were provided by Prof. Emily Liman (University of Southern California, USA)^14^,^35^. A967079, AP-18, and HC030031 were from Tocris Biosci. (Minneapolis, MN). AITC, ATP, and nimodipine were from Sigma-Aldrich (St. Louis, MO).

### STC-1 cell GLP-1 secretion assay

STC-1 cells were maintained in RTC-coated 96-well cell culture plates and were allowed to reach 85–95% confluence. On the day of the experiment, the culture medium was replaced with DMEM containing 5.6 mM glucose and 0.1% bovine serum albumin (BSA) and the cells were then serum starved for 3 hours while equilibrated in a tissue culture incubator. The medium was then replaced with the fresh DMEM containing 5.6 mM glucose and 0.1% BSA with or without the indicated test solutions so that there were four wells per each experimental condition. STC-1 cells were exposed to these test solutions for 30 min while again being equilibrated in a cell culture incubator. Medium from each of the four wells was collected and stored at −80 °C prior to immunoassays. GLP-1 in these samples was detected using a GLP-1 Total ELISA kit (Cat. No. EZGLP1T-36K; EMD Millipore, Billerica, MA) according to the manufacturer’s instructions. O.D. values for ELISA assay samples were measured using a Benchmark Plus plate-reading spectrophotometer under the control of Microplate Manager software (Bio-Rad Laboratories, Hercules, CA). Each experiment was repeated 3 times so that the data are the average of *N = 3* experiments. Data were evaluated for statistical significance by a paired *t* test. A *p*-value of <0.05 was considered to be statistically significant.

#### Ethical use of vertebrate animals

All experiments using mice were performed in accordance with relevant guidelines and regulations specified in the Animal Welfare Act (AWA) (7 U.S.C. § 2131) per United States of America federal government law. Ethical use of mice for the experiments reported here were also in accordance with an animal use protocol (IACUC #338) that was approved by the Institutional Animal Care and Use Committee of SUNY Upstate Medical University.

#### Neonatal mouse intestinal cell culture

Newborn mice (C57BL/6) from Charles River Laboratories (Wilmington, MA) were used for preparation of mixed primary intestinal cell cultures enriched with L-cells using a modification of previously published techniques^36^,^37^,^38^.

**Step #1.** Newborn mice (C57BL/6) from Charles River Laboratories (Wilmington, MA) were euthanized according to a SUNY Upstate Medical University animal use protocol (IACUC #338, mice for a separate project donated by Dr. Li-Ru Zhao). The entire intestine was removed, rinsed, and chopped into 1–2 mm pieces in ice-cold Ca^2+^-free Hank’s balanced salt solution containing 0.65 mM dithiothreitol, 1% BSA, penicillin, streptomycin, 9.05 mM N_a_HCO_3_, and 20 mM HEPES. The tissue was then digested for 15 min in a 37 °C shaking water bath incubator using 5 ml of 100 U/ml collagenase type I (Sigma) in Basal Medium Eagle (Thermo Fisher Scientific) and supplemented with 1% BSA, 26.4 mM N_a_HCO_3_, and 10 mM HEPES (pH 6.9). Digested tissue was centrifuged at 120 × g and the resulting pellet was re-suspended in DMEM containing 5.6 mM glucose (Cat. #11885, Gibco/Thermo Fisher), penicillin, streptomycin, and 5% FBS.

**Step #2.** The re-suspended tissue digest derived by centrifugation was filtered through an 80 μm nylon filter (Merk Millilipore, Darmstadt, Germany). The filtered eluent containing intestinal cells was subjected to two rounds of centrifugation at 120 × g for 3 min each cycle in DMEM containing 2% sorbitol. The final pellet was re-suspended in DMEM containing penicillin, streptomycin, 10% FBS, 1 μg/ml insulin (Sigma), and 20 ng/ml epidermal growth factor (EGF, Sigma). Cells were added to culture plates (Cat. No. 10062-892, VWR International, LLC, Radnor, PA) for culture at 37 °C in DMEM containing 5.6 mM glucose, penicillin, streptomycin, and 5% FBS.

### GLP-1 release assay

Primary cell cultures enriched with mouse L-cells were allowed to achieve 70–90% confluence in a humidified incubator gassed with 5% C0_2_. On the day of the experiment, they were equilibrated for 3 hours in serum-free DMEM containing 5.6 mM glucose, penicillin, streptomycin, and 0.1% BSA. After washing the cultures three times with serum-free DMEM, GLP-1 release assays were performed for 30 minutes during which the cells were exposed to test compounds dissolved in serum-free DMEM containing 5.6 mM glucose and 0.1% BSA. An EIA-GLP-1 ELISA kit (RayBio, Norcross, GA) was used to detect GLP-1 released into the assay medium. All samples were assayed in duplicate.

### RT-PCR for TRPA1 mRNA

RNA was isolated from STC-1 cells using RNeasy kits (Qiagen). Quantitect Reverse Transcriptase (RT) kits (Qiagen) were used to generate cDNA per kit instructions with control reactions performed without RT. The PCR primers were designed against a mouse sequence for TRPA1(GenBank NM_177781.4) and had the following sequences: Sense primer [ATGTCACCCCTTCACATAGC]; Anti-sense primer #1 [CGTGTTCCCATTCTCTCCTT]; Anti-sense primer #2 [GGCTGGCTTTCTTGTGATTC]. Both anti-sense primers were used with the same sense primer. PCR products spanned one or two exons and had predicted product sizes of 109 and 331 bp, repectively. PCR reactions were performed using a Mini-Opticon cycler (Bio-Rad, Hercules, CA) and a QuantiTect SYBR-Green PCR kit (Qiagen). The thermal cycle parameters were: 95 °C for 15 min followed by 35 cycles of 95 °C for 15 s, 57 °C for 30 s and 72 °C for 30 s. A melting curve analysis was performed from 60 °C to 85 °C, and products were run on 2% agar gels to test product specificity using GelRed loading buffer and a pre-stained DNA marker (GenScript, Piscataway, NJ). PCR products were extracted from these gels using a QIAquick Gel Extraction kit (Qiagen).

### qRT-PCR for TRPA1 mRNA

Quantitative real-time polymerase chain reaction (qRT-PCR) analysis was used to detect TRAP1 mRNA expression in mixed primary L-cell cultures. Cells were lysed in Trizol (Invitrogen) and total RNA was extracted. First-strand cDNA synthesis was performed with 1 μg of total RNA in 20 μl reactions using an iScript cDNA Synthesis Kit (Bio-Rad, Hercules, CA). qRT-PCR was then performed using iQ SYBR Green Mix (Bio-Rad, Hercules, CA). Relative gene expression was determined by the CT method, and TRPA1 mRNA levels were normalized relative to the levels of glyceraldehyde phosphodehydrogenase (GAPDH) mRNA. Primers used for TRAP1 were: sense 5′-CCATGACCTGGCAGAATACC-3′ and antisense 5′-TGGAGAGCGTCCTTCAGAAT-3′. Primers used for GAPDH were: sense 5′-CAATGTGTC CGTGGA-3′ and antisense 5′-GATGCCTGCTTCACCACC-3.

### Immunocytochemistry for detection of GLP-1

Cultures of mouse intestinal cells were fixed for 10 min at room temperature in PBS containing 4% paraformaldehyde, and were then permeabilized by incubation for 10 min in PBS containing 0.25% Triton X-100. The blocking buffer was PBS-Tween containing 1% BSA. Cells were exposed at 4 °C overnight to PBS-Tween containing a 1:50 dilution of a mouse monoclonal anti-GLP-1 antibody (Cat. No. AB23468, Abcam, Cambridge, MA). A goat anti-mouse polyclonal antiserum conjugated to horseradish peroxidase (HRP) served as the secondary antibody (Cat. No. 1706516, Bio-Rad, Hercules, CA). A DAB Stain kit (Vector, CA) was used to detect the HRP reaction product that signified GLP-1 immunoreactivity, whereas hematoxylin was used to detect nuclei. Photomicrographs were taken with an Eclipse TE 2000-U microscope (Nikon).

### Primary and secondary screens of a cycloalka[*b*]indole library

The ELISA-based primary screen to identify JWU-A021 as a GLP-1 secretagogue was performed as part of the Open Innovation Drug Discovery Program (OIID) of Eli Lilly and Company. Subsequent identification of JWU-A021 as a TRPA1 channel activator was achieved at the Holz laboratory in a fura-2-based secondary screen that used a panel of calcium channel blockers or activators. OIID screening data presented in Fig. 2a–c was supplied courtesy of Eli Lilly and Company-used with Lilly’s permission. To learn more about the Lilly Open Innovation Drug Discovery Program, please visit the program website at https://openinnovation.lilly.com (last accessed on 05-04-2016).

### Statistical analyses

The repeatability of findings was confirmed by performing all experiments a minimum of three times. GLP-1 secretion assay data and qRT-PCR data were evaluated for statistical significance by Student’s paired *t* test or by ANOVA analysis followed by a Bonferroni post test, as indicated in the figure legends. For all assays, a p-value of <0.05 was considered to be statistically significant. Appropriate sample size was determined post-hoc so that the sample size could be increased, if necessary, so that statistical significance would be achieved if it existed.

## Additional Information

**How to cite this article**: Chepurny, O. G. *et al*. Synthetic small molecule GLP-1 secretagogues prepared by means of a three-component indole annulation strategy. *Sci. Rep*. **6**, 28934; doi: 10.1038/srep28934 (2016).

## Supplementary Material

Supplementary Information
