# Supplementary material for: Synthetic small molecule GLP-1 secretagogues prepared by means of a three-component indole annulation strategy
Source: Sci Rep. 2016 Jun 29;6:28934. doi: 10.1038/srep28934 (PMC4926213; doi:10.1038/srep28934)
Supplement: Supplementary Information [file srep28934-s1.pdf]

Synthetic small molecule GLP-1 secretagogues prepared by means of a three-component indole annulation strategy

Oleg G. Chepurny[1], Colin A. Leech[1], Martin Tomanik[2], Maria C. DiPoto[2], Hui Li[2], Xinping Han[2,†], Qinghe Meng[3], Robert N. Cooney[3], Jimmy Wu[2]*, George G. Holz[1,4]*

Departments of Medicine[1], Surgery[3], and Pharmacology[4], State University of New York (SUNY), Upstate Medical University, Syracuse, New York, USA

Department of Chemistry[2], Dartmouth College, Hanover, New Hampshire, USA

[†]Current Address: Department of Biochemistry, UT Southwestern, Dallas TX 75390

Table of Contents

General Information S2

Synthetic Experimental Procedures S3

Characterization of Cyclohepta[*b*]indole products S4

Characterization of Cyclopenta[*b*]indole products S7

References S11

NMR Spectra

- JWU-A029 .... S12
- JWU-A030 .... S18
- JWU-A031 .... S24
- JWU-A033 .... S30
- JWU-A034 .... S36
- (syn)-JWU-B007 .... S42
- (anti)-JWU-B008 .... S47
- (syn)-JWU-B009 .... S53
- (anti)-JWU-B010 .... S59
- (anti)-JWU-B011 .... S65
- (syn)-JWU-B012 .... S71
- JWU-B014 .... S77

X-Ray Crystallography Data for (–)-JWU-A021 .... S82

General Information

^1H NMR data were recorded on a Bruker Avance III 500 MHz spectrometer (TBI probe) and Bruker Avance III 600 MHz (BBFO probe) with calibration spectra to $CHCl_3$ (7.26 ppm) and CH_2Cl_2 (5.32 ppm) at ambient temperature. Multiplicities are indicated as s (singlet), d (doublet), t (triplet), and m (multiplet). ^{13}C NMR data were recorded at 125 MHz on Bruker Avance III 600 MHz spectrometer (BBFO probe) at ambient temperature and expressed in ppm using solvent as the internal standard CD_2Cl_2 (53.84 ppm) and $CDCl_3$ (77.16 ppm). IR spectra were recorded on Jasco FT-IR 4100 Series spectrophotometer, v_{max} (cm^{-1}) are partially reported. Analytical thin layer chromatography (TLC) was performed on SILICYCLE pre-coated TLC plates (silica gel 60 F-254, 0.25mm). Flush column chromatography was performed on silica gel 60 (SILICYCLE 230−400 mesh). Visualization was accomplished with UV light and ceric ammonium molybdate (CAM). High-resolution mass spectroscopy data were acquired from Mass Spectrometry Laboratory of the University of Illinois (Urbana-Champaign, IL).

All reactions were carried out in oven-dried glassware with magnetic stirring. Solvents were freshly distilled. All reagents and starting materials were purchased from commercial vendors and used without further purification.

Experimental Procedures

General Procedure ***A*** *for the Synthesis of Cyclohepta[b]indoles*

A round-bottom flask was charged with indole (0.66 mmol, 1 equiv), aldehyde or ketone (1.32 mmol, 2 equiv), and diene (3.30 mmol, 5 equiv). Then, CH_2Cl_2 (2.0 mL) was added followed by $GaBr_3$ (0.07 mmol, 0.1 equiv). The reaction was stirred at room temperature until it was complete as judged by thin layer chromatography. The volatiles were concentrated *in vacuo* and the residue was purified via silica gel flash chromatography (EtOAc/Hexanes) to yield the desired products.

General Procedure ***B*** *for the Synthesis of Cyclopenta[b]indoles*

A round-bottom flask was charged with indole (0.66 mmol, 1 equiv), aldehyde or ketone (1.32 mmol, 2 equiv), and styrene (3.30 mmol, 5 equiv). Then, dichloroethane (2.0 mL) was added followed by TfOH (0.13 mmol, 0.2 equiv). The reaction was stirred at room temperature until it was complete as judged by thin layer chromatography. The volatiles were concentrated *in vacuo* and the residue was purified via silica gel flash chromatography (EtOAc/Hexanes) to yield the desired products.

Characterization of Cyclohepta[b]indole products

The preparation of **JWU-A001** through **JWU-A021** were carried out as previously described.[1] All characterization data were identical to those previously reported.[1]

FIG. S1

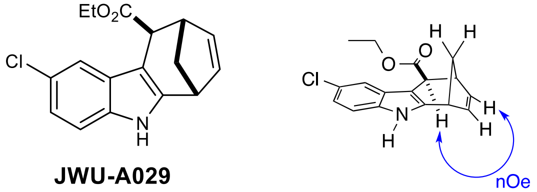

JWU-A029

By following the general procedure **A** described above, **JWU-A029** was prepared in 43% yield. **^{1}H NMR** (CD_2Cl_2, 600 MHz): δ, ppm 8.04 (1H, s), 7.40 (1H, d, J = 1.5 Hz), 7.14 (1H, d, J = 8.8 Hz), 6.93 (1H, dd, J = 2.5, 9.0 Hz), 6.34 (1H, dd, J = 3.0, 5.6 Hz), 5.85 (1H, dd, J = 3.0, 6.3 Hz), 4.13 (2H, q, J = 7.4 Hz), 3.47 (1H, d, J = 1.0 Hz), 3.40 (1H, dd, J = 3.3, 4.8 Hz), 3.19 (1H, t, J = 4.2 Hz), 2.28 (1H, d, J = 10.4 Hz), 2.13−2.09 (1H, m), 1.23 (3H, t, J = 8.3 Hz); **^{13}C NMR** (CD_2Cl_2, 150 MHz): δ 173.6, 142.4, 140.5, 133.0, 131.6, 129.5, 125.2, 120.5, 117.9, 111.6, 101.6, 60.8, 42.3, 41.3, 39.6, 38.8, 14.0; **IR** (film, cm^{-1}): 3401, 2957, 2846, 1713, 1644, 1468, 1259, 732; **HRMS (ESI) calcd.** for $C_{17}H_{16}ClNO_2$ (m/z $M+H^+$): 302.0948, found: 302.0950.

FIG. S2

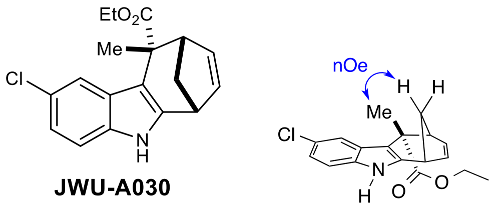

JWU-A030

By following the general procedure **A** described above, **JWU-A030** was prepared in 57% yield. **^{1}H NMR** (CD_2Cl_2, 600 MHz): δ, ppm 8.01 (1H, s), 7.29 (1H, d, J = 2.1 Hz), 7.13 (1H, d, J = 9.0 Hz), 6.91 (1H, dd, J = 1.7, 8.0 Hz), 6.36 (1H, dd, J = 3.0, 5.4 Hz), 5.70 (1H, dd, J = 3.2, 5.2 Hz), 4.17−4.11 (1H, m), 4.06−3.98 (1H, m), 3.29 (1H, t, J = 3.7 Hz), 3.01 (1H, dd, J = 3.0, 4.8 Hz), 2.24 (2H, m, J = 4.9 Hz), 2.07 (1H, d, J = 9.9 Hz), 1.63 (3H, s), 1.16 (2H, t, J = 6.7 Hz); **^{13}C NMR** (CD_2Cl_2, 150 MHz): δ 175.2, 140.7, 140.4, 132.9, 131.5, 129.0, 124.7, 120.1, 119.0, 111.7, 108.0, 60.5, 49.9, 46.1, 40.9, 39.0, 25.7, 14.0; **IR** (film, cm^{-1}): 3455, 3048, 2981, 1720, 1637, 1451, 1262, 736; **HRMS (ESI) calcd.** for $C_{18}H_{18}ClNO_2$ (m/z $M+H^+$): 316.1104, found: 316.1104.

FIG. S3

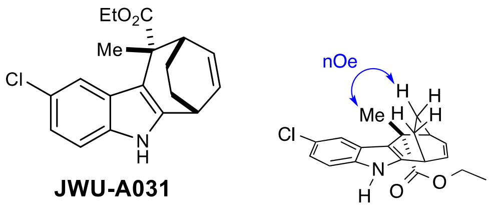

JWU-A031

By following the general procedure **A** described above, **JWU-A031** was prepared in 11% yield. **^{1}H NMR** (CD_2Cl_2, 600 MHz): δ, ppm 7.84 (1H, s), 7.31 (1H, d, J = 2.0 Hz), 7.13 (1H, d, J = 9.4 Hz), 6.93 (1H, dd, J = 2.2, 8.6 Hz), 6.45 (1H, dd, J = 8.0, 8.0 Hz), 6.00 (1H, t, J = 8.0 Hz), 4.11−4.05 (1H, m), 4.02−3.96 (1H, m), 3.24 (1H, t, J = 4.4 Hz), 2.87 (1H, t, J = 7.6 Hz), 2.08−1.96 (2H, m), 1.85−1.78 (1H, m), 1.71−1.65 (1H, m), 1.64 (3H, s), 1.12 (3H, t, J = 7.8 Hz); **^{13}C NMR** (CD_2Cl_2, 150 MHz): δ 175.6, 139.3, 135.9, 132.7, 130.9, 130.4, 124.6, 120.7, 120.6, 119.9, 110.6, 60.7, 42.2, 34.2, 30.2, 24.3, 19.0, 14.1, 14.0; **IR** (film, cm^{-1}): 3399, 2923, 2853, 1720, 1637, 796; **HRMS (ESI) calcd.** for $C_{19}H_{20}ClNO_2$ (m/z $M+H^+$): 330.1261, found: 330.1254.

FIG. S4

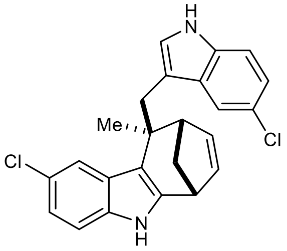

JWU-A033

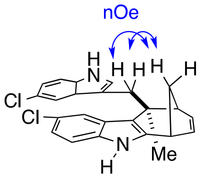

By following the general procedure **A** described above, **JWU-A033** was prepared in 25% yield. **¹H NMR** (CD_2Cl_2, 600 MHz): δ, ppm 8.18 (1H, s), 7.95 (1H, s), 7.38 (1H, d, J = 1.6 Hz), 7.24 (1H, d, J = 8.1 Hz), 7.14 (2H, d, J = 8.9 Hz), 7.01 (1H, dd, J = 2.8, 8.9 Hz), 6.94 (1H, d, J = 2.8 Hz), 6.88 (1H, dd, J = 2.0, 8.1 Hz), 6.32 (1H, dd, J = 3.1, 5.1 Hz), 5.71 (1H, dd, J = 3.4, 5.5 Hz), 3.29 (1H, d, J = 15.7 Hz), 3.27 (1H, dd, J = 3.3, 3.3 Hz), 3.11 (1H, d, J = 14.2 Hz), 2.70 (1H, dd, J = 3.3, 4.8 Hz), 2.25 (1H, d, J = 8.4 Hz), 2.12−2.08 (1H, m), 1.19 (3H, d, J = 11.3 Hz); **¹³C NMR** (CD_2Cl_2, 150 MHz): δ 140.9, 139.6, 134.4, 133.1, 132.8, 130.2, 128.7, 125.4, 124.8, 124.4, 121.6, 119.7, 118.7, 118.6, 113.0, 112.1, 112.0, 111.6, 48.8, 40.2, 40.1, 39.0, 37.0, 21.7; **IR** (film, cm^{-1}): 3427, 2920, 2853, 1648, 1436, 1098, 737; **HRMS (ESI) calcd.** for $C_{24}H_{20}Cl_2N_2$ (m/z $M+H^+$): 407.1082, found: 407.1074.

FIG. S5

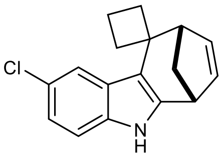

JWU-A034

By following the general procedure **A** described above, **JWU-A034** was prepared in 58% yield. **¹H NMR** (CD_2Cl_2, 600 MHz): δ, ppm 7.87 (1H, s), 7.63 (1H, d, J = 2.5 Hz), 7.14 (1H, d, J = 8.6 Hz), 6.92 (1H, dd, J = 2.7, 8.4 Hz), 6.43 (1H, dd, J = 2.9, 5.9 Hz), 5.85 (1H, dd, J = 3.5, 5.3 Hz), 3.22 (1H, dd, J = 3.0, 3.9 Hz), 3.19 (1H, dd, J = 3.2, 5.2 Hz), 2.69−2.63

(1H, m), 2.56−2.50 (1H, m), 2.20−2.07 (3H, m), 2.06−1.99 (2H, m), 1.92 (1H, d, J = 10.8 Hz); **^{13}C NMR** (CD_2Cl_2, 150 MHz): δ 142.0, 141.3, 133.2, 131.7, 128.9, 124.7, 119.8, 117.6, 111.8, 111.6, 49.8, 41.6, 41.1, 39.1, 35.3, 27.5, 15.0; **IR** (film, cm^{-1}): 3399, 2923, 2853, 1644, 1467, 1439, 1067, 741; **HRMS (ESI) calcd.** for $C_{17}H_{16}ClN$ (m/z $M+H^+$): 270.1050, found: 270.1050.

Characterization of Cyclopenta[b]indole products

FIG. S6

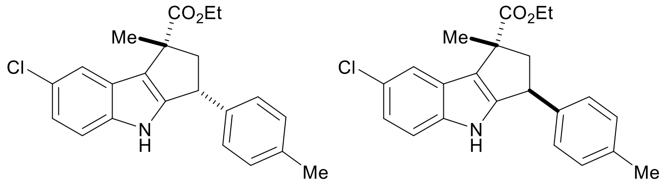

(syn)-JWU-B007 **(anti)-JWU-B008**

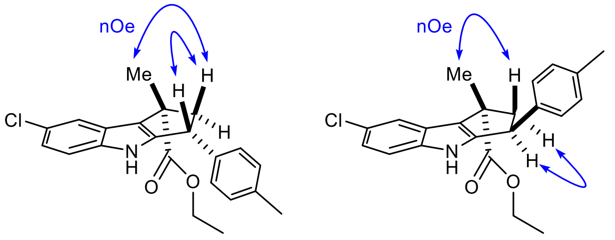

(syn)-JWU-B007 **(anti)-JWU-B008**

By following the general procedure **B** described above, a 1:1 diastereomeric mixture **(syn)-JWU-B007** and **(anti)-JWU-B008** was prepared in 65% combined yield. **For (syn)-JWU-B007**, **^{1}H NMR** (CD_2Cl_2, 600 MHz): δ, ppm 7.82 (1H, s), 7.57 (1H, d, J = 2.1 Hz), 7.12 (1H, d, J = 8.8 Hz), 7.04 (2H, d, J = 8.0 Hz), 6.99 (3H, ddd, J = 8.8, 8.8, 3.5 Hz), 4.36 (1H, dd, J = 6.9, 8.0 Hz), 4.12−4.07 (2H, m), 2.94 (1H, dd, J = 7.0, 13.5 Hz), 2.74 (1H, dd, J = 8.6, 13.3 Hz), 2.23 (3H, s), 1.54 (3H, s), 1.20 (3H, t, J = 7.9 Hz);

^{13}C NMR (CD_2Cl_2, 150 MHz): δ 176.0, 146.0, 139.9, 139.4, 136.6, 129.3, 127.4, 125.2, 124.6, 122.7, 121.2, 118.8, 112.5, 60.9, 51.5, 49.3, 43.3, 25.1, 20.7, 14.0; **IR** (film, cm^{-1}):

3423, 2923, 2860, 1644, 1449, 1289, 1025, 789; **HRMS (ESI) calcd.** for $C_{22}H_{22}ClNO_2$ (m/z $M+H^+$): 368.1417, found: 368.1417; **For (anti)-JWU-B008**, **^{1}H NMR** (CD_2Cl_2, 600 MHz): δ, ppm 7.83 (1H, s), 7.54 (1H, d, J = 1.5 Hz), 7.13 (1H, d, J = 8.9 Hz), 7.06 (2H, d, J = 7.5 Hz), 7.00 (3H, ddd, J = 5.7, 5.7, 13.9 Hz), 4.49 (1H, t, J = 7.9 Hz), 4.06−4.00 (2H, m), 3.54 (1H, dd, J = 9.0, 13.2 Hz), 2.25 (3H, s), 2.11 (1H, dd, J = 8.2, 12.9 Hz), 1.65 (3H, s), 1.15 (3H, t, J = 8.2 Hz); **^{13}C NMR** (CD_2Cl_2, 150 MHz): δ 175.7, 146.7, 140.2, 139.3, 136.6, 129.4, 127.4, 125.2, 124.7, 122.5, 121.1, 118.3, 112.6, 60.9, 52.7, 49.5, 44.2, 25.2, 20.7, 14.0; **IR** (film, cm^{-1}): 3421, 2918, 2853, 1640, 1374, 1287, 861; **HRMS (ESI) calcd.** for $C_{22}H_{22}ClNO_2$ (m/z $M+H^+$): 368.1417, found: 368.1409.

FIG. S7

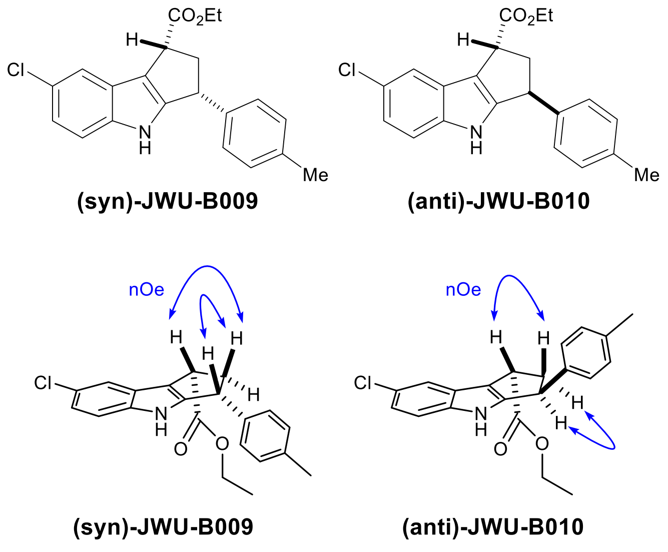

By following the general procedure **B** described above, a 1:1 diastereomeric mixture **(syn)-JWU-B009** and **(anti)-JWU-B010** as prepared in 44% combined yield. **For (syn)-JWU-B009**, **^{1}H NMR** (CD_2Cl_2, 600 MHz): δ, ppm 7.87 (1H, s), 7.54 (1H, s), 7.13 (1H, d, J = 8.3 Hz), 7.05 (4H, s), 7.00 (1H, d, J = 6.7 Hz), 4.32 (1H, dd, J = 7.6, 7.6 Hz), 4.15 (2H, dd, J = 6.4, 14.6 Hz), 4.03 (1H, dd, J = 7.0, 7.0 Hz), 3.19−3.13 (1H, m), 2.67−2.61 (1H, m), 2.23

(3H, s), 1.25 (3H, t, J = 7.2 Hz); **^{13}C-NMR** (CD_2Cl_2, 150 MHz): δ 173.6, 147.5, 140.0, 139.5, 136.7, 129.3, 127.5, 125.3, 125.1, 121.3, 118.8, 117.4, 112.5, 60.9, 44.0, 43.1, 42.7, 20.7, 14.1; **IR** (film, cm^{-1}): 3583, 3357, 2916, 2850, 1713, 1289, 1070, 657; **HRMS (ESI) calcd.** for $C_{21}H_{20}ClNO_2$ (m/z $M+H^+$): 354.1261, found: 354.1253; **For (anti)-JWU-B010, ^{1}H NMR** (CD_2Cl_2, 600 MHz): δ, ppm 7.89 (1H, s), 7.51 (1H, d, J = 2.2 Hz), 7.13 (1H, d, J = 9.1 Hz), 7.03 (2H, d, J = 8.0 Hz), 6.99 (1H, dd, J = 2.2, 8.8 Hz), 6.95 (2H, d, J = 8.0 Hz), 4.48 (1H, dd, J = 7.3, 7.3 Hz), 4.13−4.07 (3H, m), 3.30−3.24 (1H, m), 2.52−2.47 (1H, m), 2.23 (3H, s), 1.22 (3H, t, J = 8.1 Hz); **^{13}C NMR** (CD_2Cl_2, 150 MHz): δ 173.7, 147.9, 140.3, 139.4, 136.6, 129.4, 127.1, 125.3, 125.0, 121.3, 118.6, 117.5, 112.6, 60.9, 43.9, 43.5, 42.9, 20.6, 14.1; **IR**(film, cm^{-1}): 3359, 2919, 2846, 1707, 1640, 1204, 1037, 857, 726; **HRMS (ESI) calcd.** for $C_{21}H_{20}ClNO_2$ (m/z $M+H^+$): 354.1261, found: 354.1261.

FIG. S8

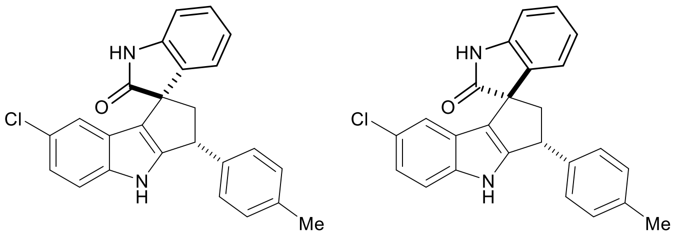

(anti)-JWU-B011 **(syn)-JWU-B012**

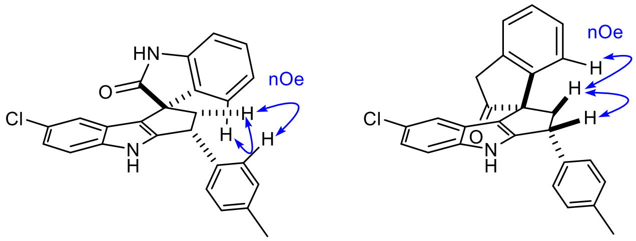

(anti)-JWU-B011 **(syn)-JWU-B012**

By following the general procedure **B** described above, a 1:1 diastereomeric mixture of **(anti)-JWU-B011** and **(syn)-JWU-B012** was prepared in 27% combined yield. **For (anti)-JWU-B011**, **^{1}H NMR** (CD_2Cl_2, 600 MHz): δ, ppm 8.01 (1H, s), 7.69 (1H, s), 7.25

(2H, d, J = 8.8 Hz), 7.18−7.10 (4H, m), 7.01 (1H, d, J = 7.7 Hz), 6.94 (1H, dd, J = 2.1, 8.8 Hz), 6.90 (2H, dd, J = 7.0, 7.0 Hz), 6.85 (1H, s), 4.76 (1H, t, J = 8.0 Hz), 3.11 (1H, dd, J = 8.0, 13.9 Hz), 2.96 (1H, dd, J = 8.8, 14.6 Hz), 2.26 (3H, s); **^{13}C NMR** (CD_2Cl_2, 150 MHz): δ 180.3, 148.9, 139.9, 139.6, 139.5, 137.0, 134.6, 129.4, 128.0, 127.8, 125.4, 123.4, 123.3, 122.7, 121.7, 120.1, 117.1, 112.8, 109.6, 54.0, 52.5, 44.5, 20.7; **IR** (film, cm^{-1}): 3397, 2918, 2360, 1709, 1465, 1290, 1098, 805; **HRMS (ESI) calcd.** for $C_{25}H_{19}ClN_2O$ (m/z $M+H^+$): 399.1261, found: 399.1257; **For (syn)-JWU-B012**, **^{1}H NMR** ($CDCl_3$, 600 MHz): δ, ppm 7.92 (1H, s), 7.47 (1H, s), 7.19 (3H, s), 7.11 (2H, ddt, J = 6.7, 6.7, 6.7 Hz), 6.99−6.94 (3H, m), 6.90 (2H, d, J = 7.8 Hz), 6.69 (1H, d, J = 1.7 Hz), 4.86 (1H, t, J = 8.8 Hz), 3.38 (1H, ddd, J = 6.8, 6.8, 6.8 Hz), 2.63 (1H, dd, J = 9.6, 14.4 Hz), 2.30 (3H, s); **^{13}C NMR** ($CDCl_3$, 150 MHz): δ 180.6, 148.6, 139.7, 139.6, 139.2, 137.1, 134.5, 129.6, 128.1, 127.9, 125.7, 123.6, 123.4, 122.9, 121.9, 120.1, 117.6, 112.6, 109.6, 54.1, 52.6, 44.6, 21.0; **IR** (film, cm^{-1}): 3273, 3186, 2923, 1692, 1619, 1468, 1339, 816; **HRMS (ESI) calcd.** for $C_{25}H_{19}ClN_2O$ (m/z $M+H^+$): 399.1261, found: 399.1249.

FIG. S9

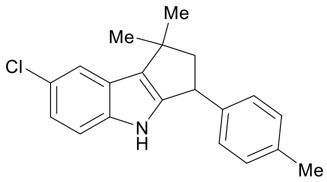

JWU-B014

By following the general procedure **B** described above, **JWU-B014** was prepared in 25% yield. **^{1}H NMR** ($CDCl_3$, 600 MHz): δ, ppm 7.70 (1H, s), 7.43 (1H, s), 7.12 (1H, d, J = 7.9 Hz), 7.03 (4H, dd, J = 8.3, 22.8 Hz), 6.96 (1H, dd, J = 1.4, 8.9 Hz), 4.39 (1H, t, J = 7.7 Hz), 2.71 (1H, dd, J = 8.8, 12.4 Hz), 2.23 (3H, s), 2.15 (1H, dd, J = 7.3, 11.7 Hz), 1.42 (3H, s), 1.31 (3H, s); **^{13}C NMR** ($CDCl_3$, 150 MHz): δ 144.7, 140.9, 139.4, 136.3, 129.3, 128.4,

127.7, 124.7, 124.5, 120.6, 117.6, 112.5, 57.2, 44.1, 39.3, 29.4, 28.5, 20.7; **IR** (film, cm^{-1}); 3418, 2923, 2853, 1633, 1443, 1287, 1044, 812; **HRMS (ESI) calcd.** for $C_{20}H_{20}ClN$ (m/z $M+H^+$); 310.1363, found: 310.1363.

1. Han, X.; Li, H.; Hughes, R. P.; Wu, J. Gallium(III)-Catalyzed Three-Component (4+3) Cycloaddition Reactions. *Angew. Chem. Int. Ed.* **2012**, *52*, 10390–10393.

MT-N3-P106-TT29-8-4-5 11 1 N:\b600\wu\data\wu_guest\nmr

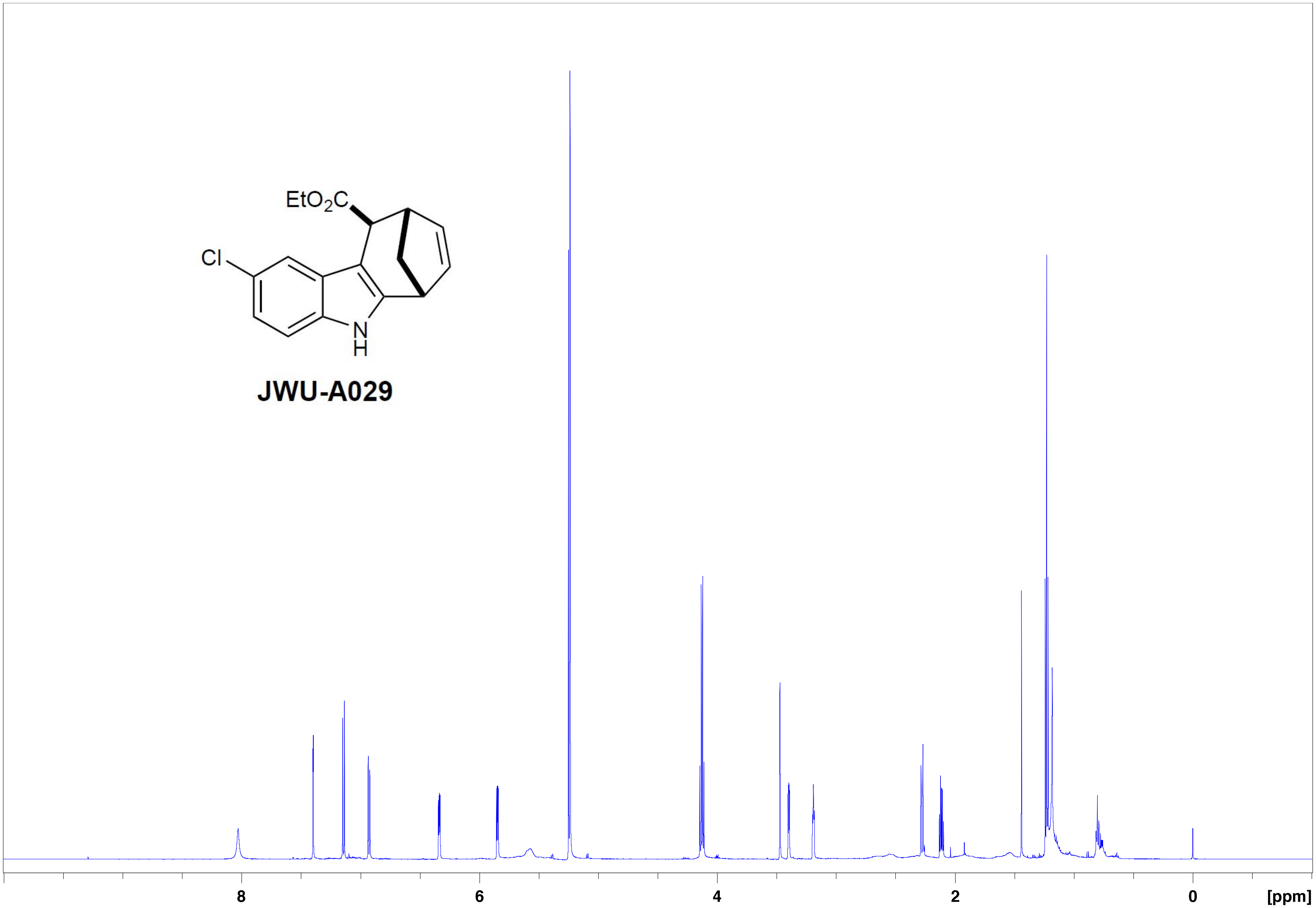

MT-N3-P106-TT29-8-4-5 10 1 N:\b600\wu\data\wu_guest\nmr

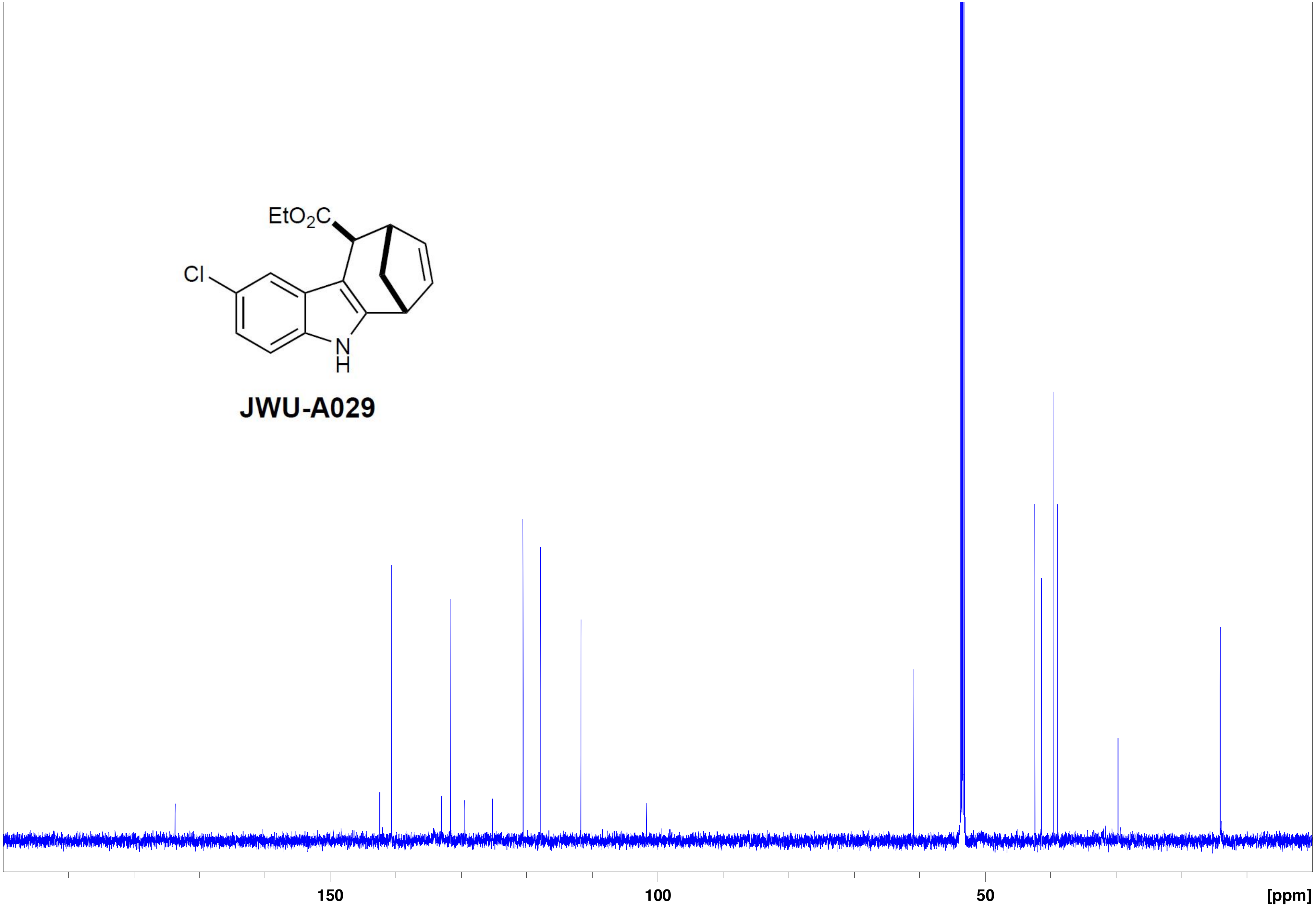

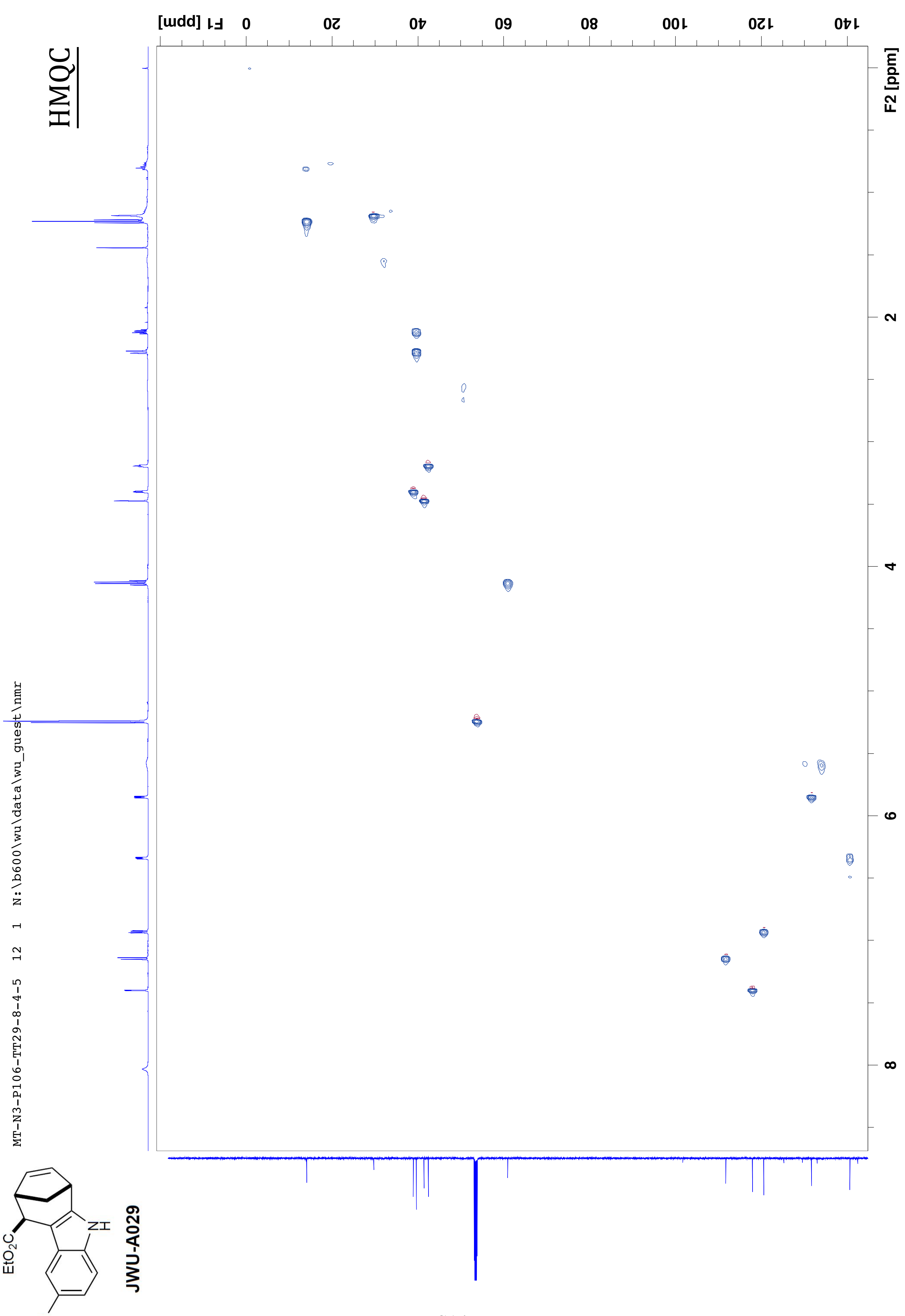
HMQC
MT-N3-P106-TT29-8-4-5 12 1 N:\b600\wu\data\wu_guest\nmr
F1 [ppm]
0 20 40 60 80 100 120 140
F2 [ppm]
2 4 6 8
EtO2C
NH
Cl
JWU-A029

HMBC

MT-N3-P106-TT29-8-4-5 13 1 N:\b600\wu\data\wu_guest\nmr

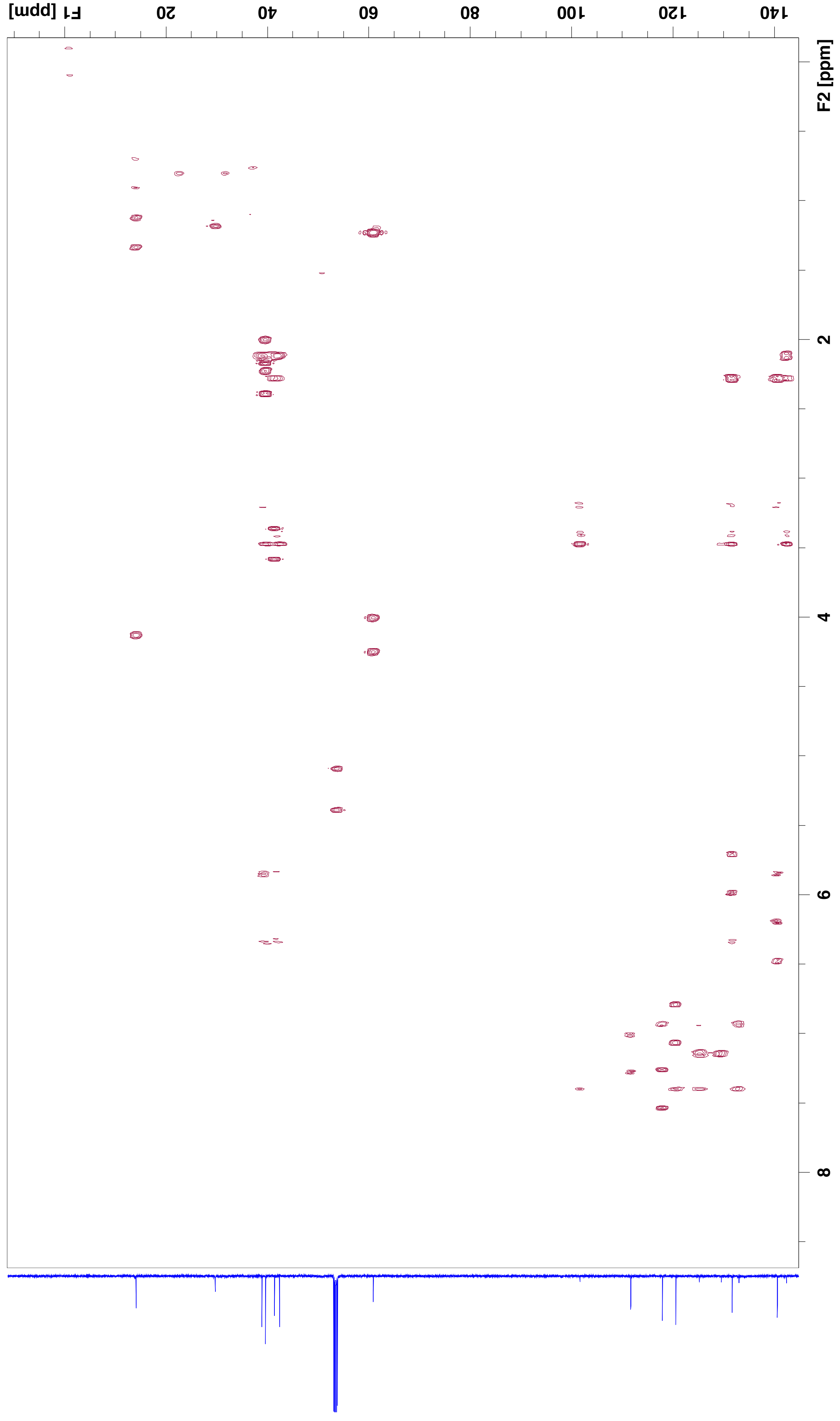

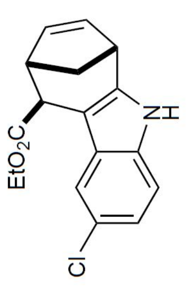

JWU-A029

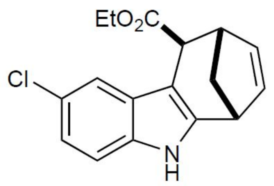
EtO2C
Cl
N
H

JWU-A029

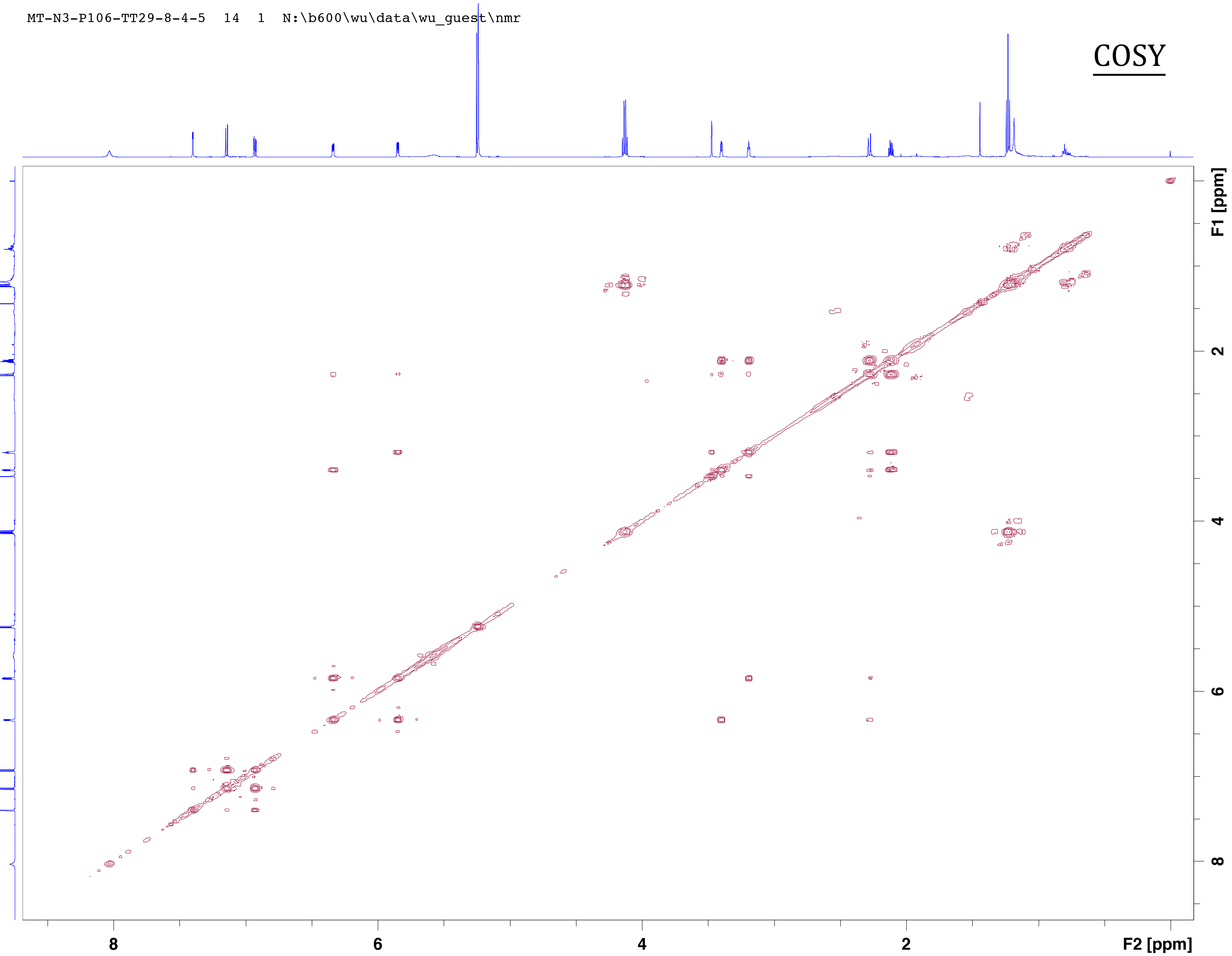
MT-N3-P106-TT29-8-4-5 14 1 N:\b600\wu\data\wu_guest\nmr
COSY
F1 [ppm]
F2 [ppm]
2
4
6
8

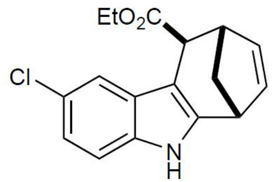
EtO2C
Cl
N
H

JWU-A029

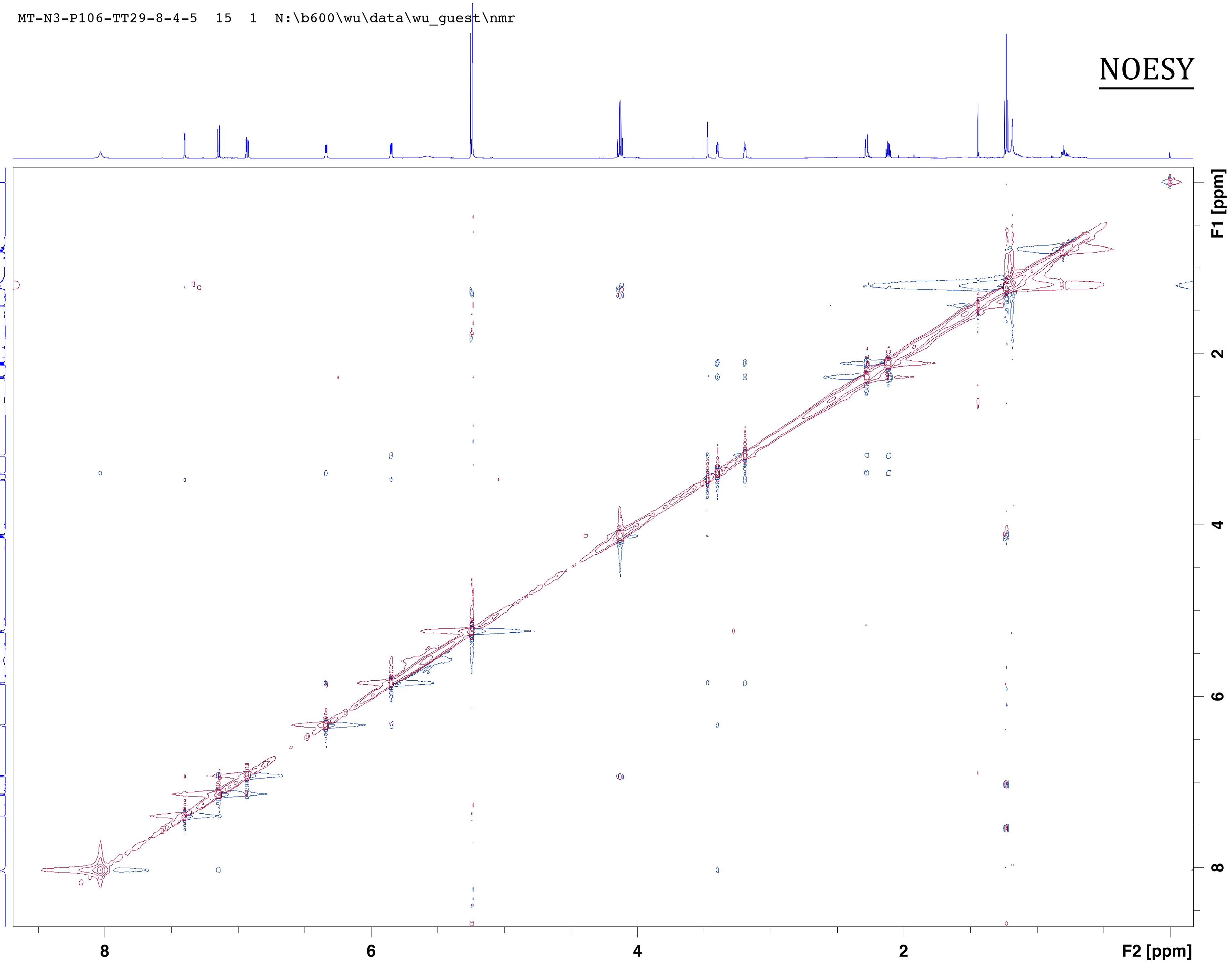
MT-N3-P106-TT29-8-4-5 15 1 N:\b600\wu\data\wu_guest\nmr
NOESY
F1 [ppm]
F2 [ppm]
8
6
4
2

MT-N3-P102-TT26-5-5 10 1 N:\b600\wu\data\wu_guest\nmr

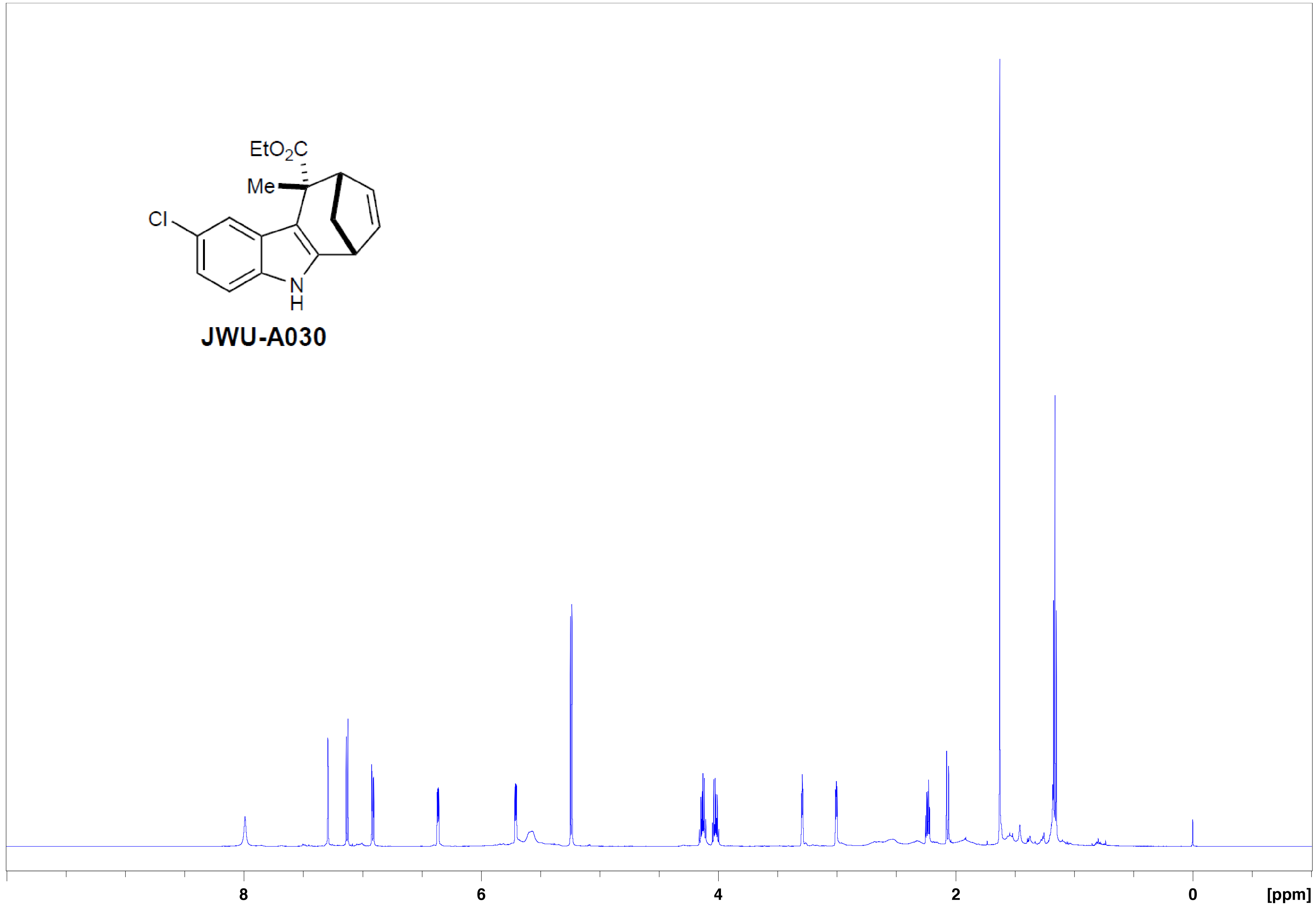

MT-N3-P102-TT26-5-5   11   1   N:\b600\wu\data\wu_guest\nmr

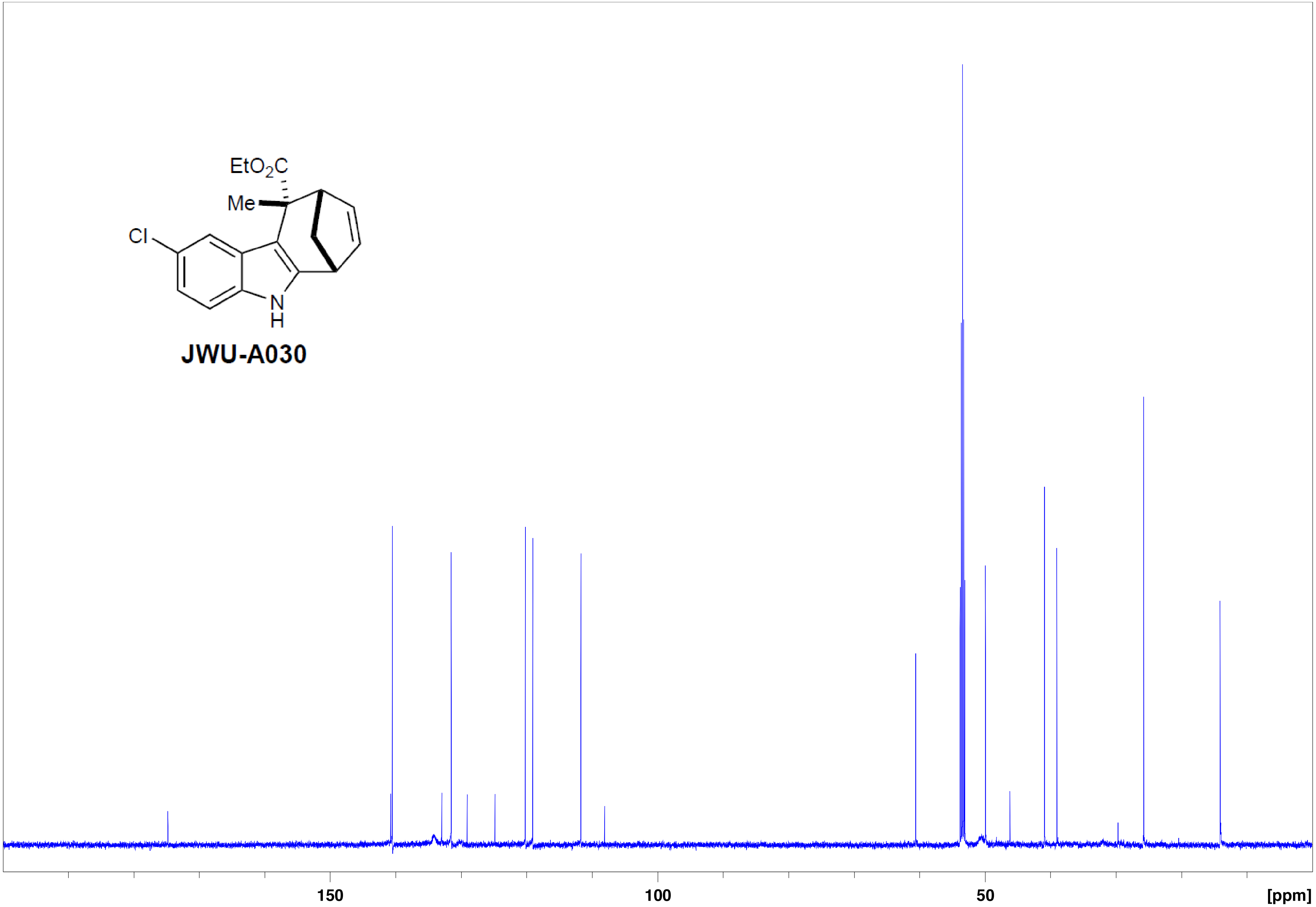

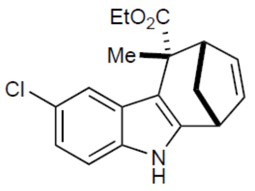
JWU-A030

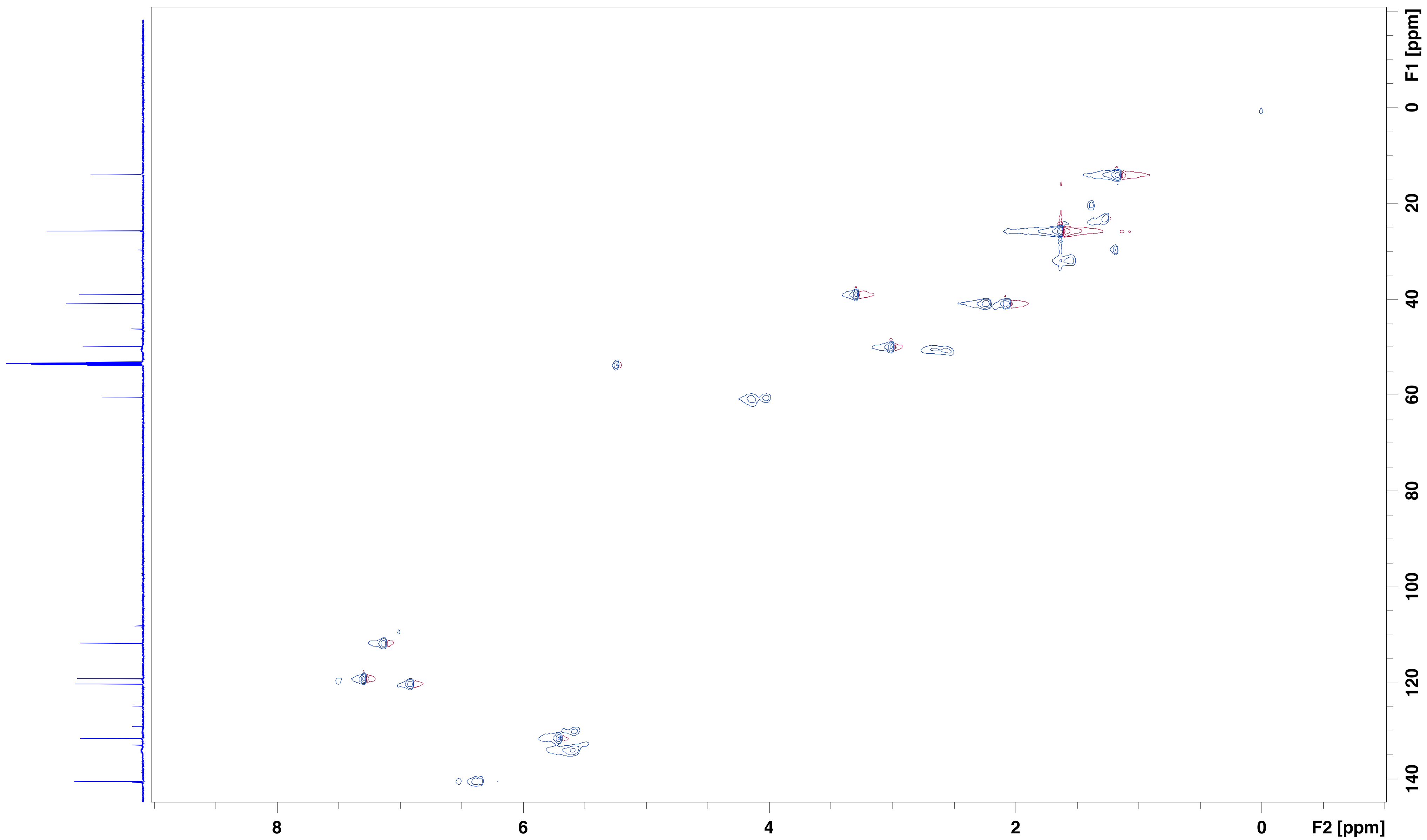
MT-N3-P102-TT26-5-5 13 1 N:\b600\wu\data\wu_guest\nmr
HMQC
F1 [ppm]
F2 [ppm]

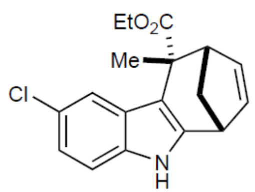

EtO2C
Me
Cl
N
H

JWU-A030

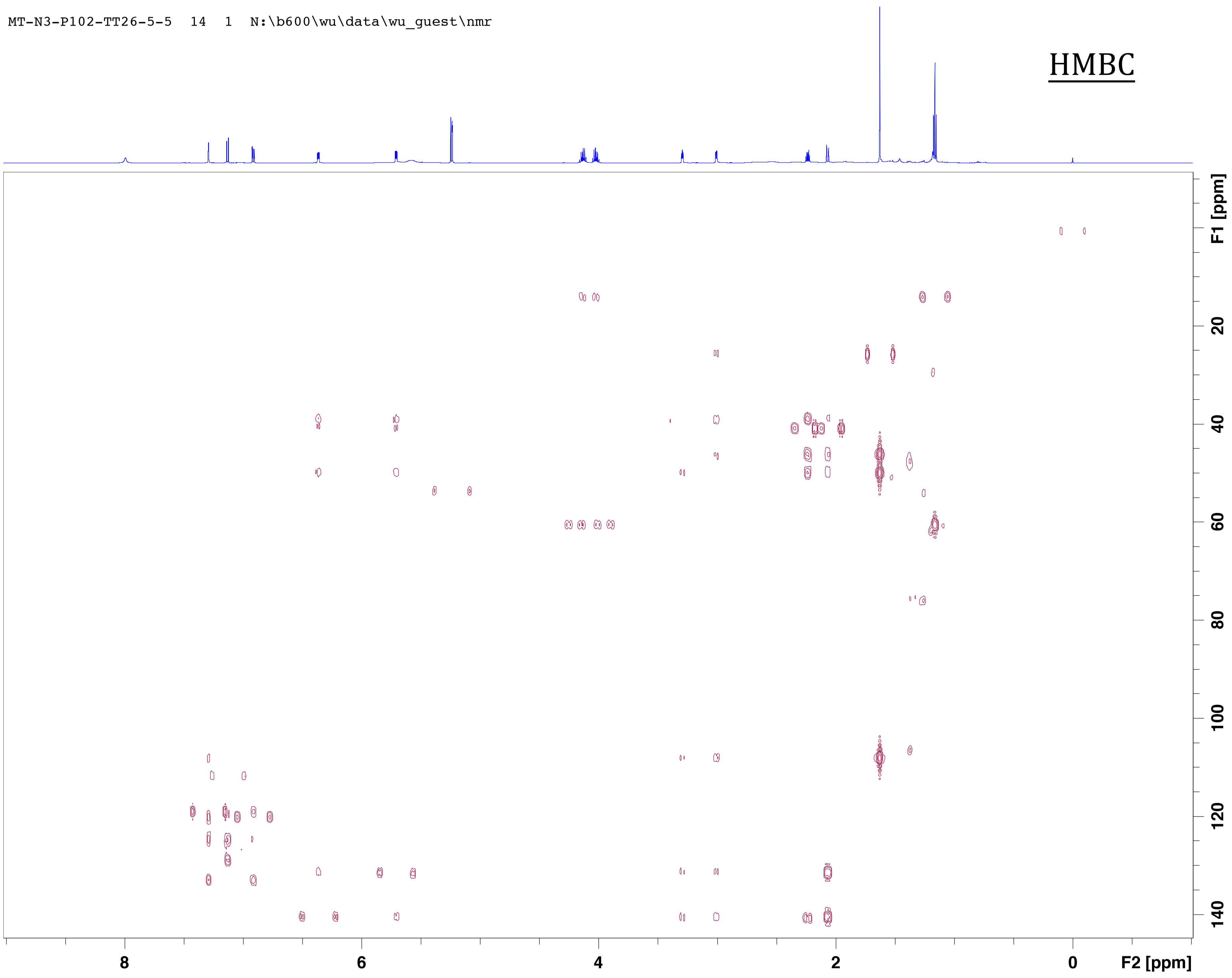

MT-N3-P102-TT26-5-5 14 1 N:\b600\wu\data\wu_guest\nmr
HMBC
F1 [ppm]
F2 [ppm]
8
6
4
2
0
20
40
60
80
100
120
140

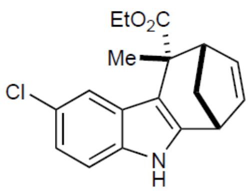

JWU-A030

MT-N3-P102-TT26-5-5 16 1 N:\b600\wu\data\wu_guest\nmr

COSY

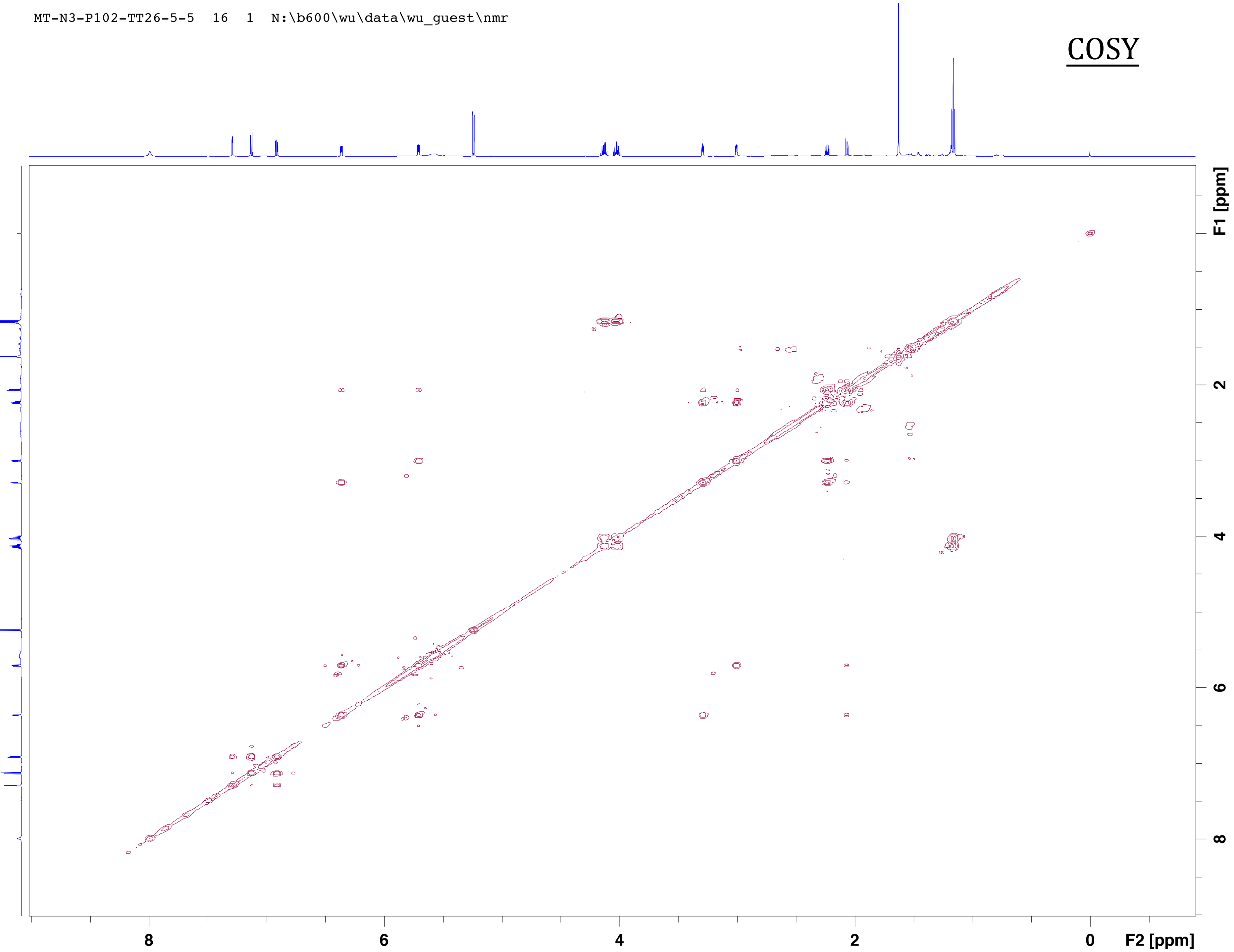

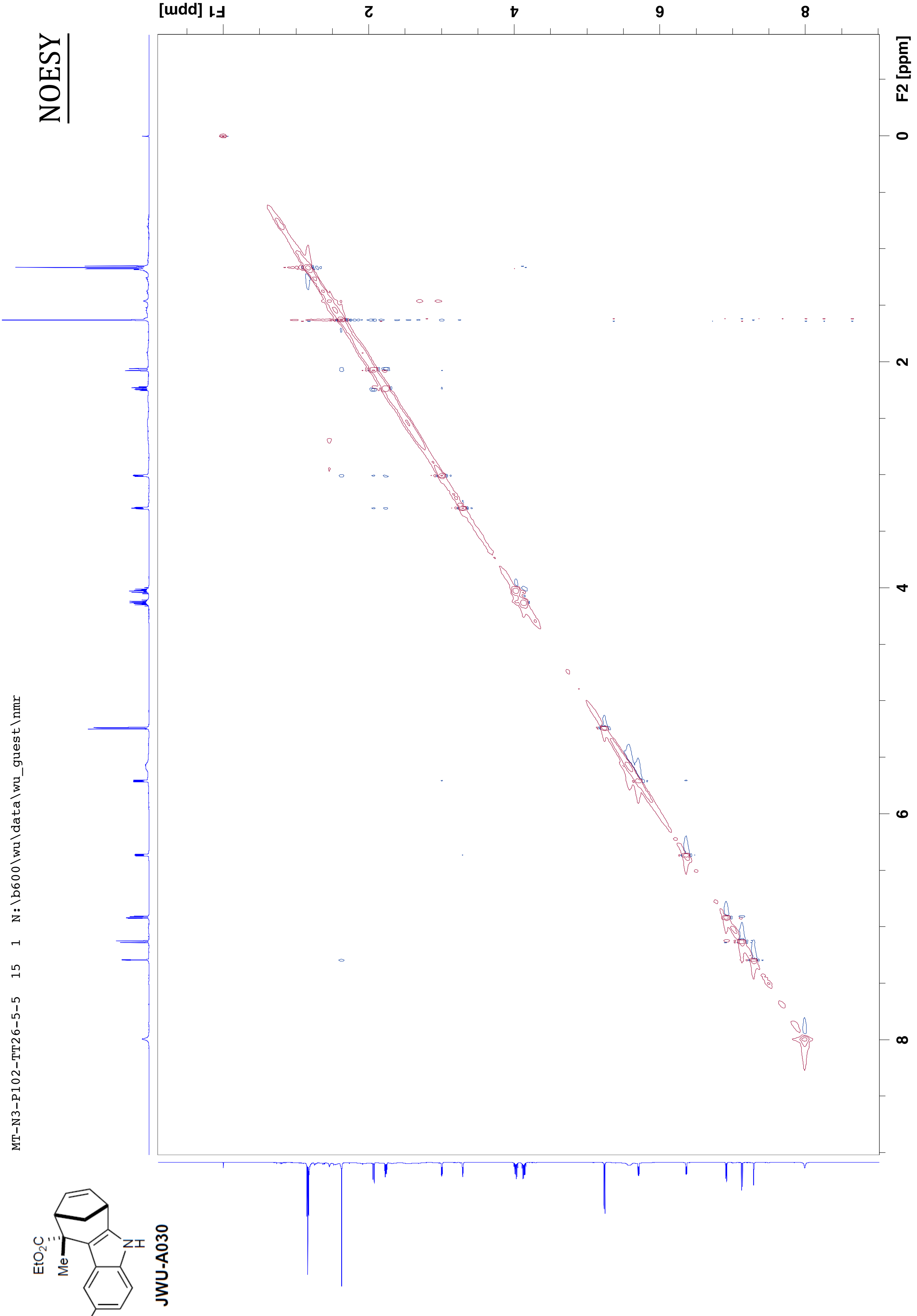
NOESY
MT-N3-P102-TT26-5-5 15 1 N:\b600\wu\data\wu_guest\nmr
F1 [ppm]
F2 [ppm]
JWU-A030
EtO2C
Me
Cl
NH

MT-N3-P154-TT14-15-ETOAc 10 1 N:\b600\wu\data\wu_guest\nmr

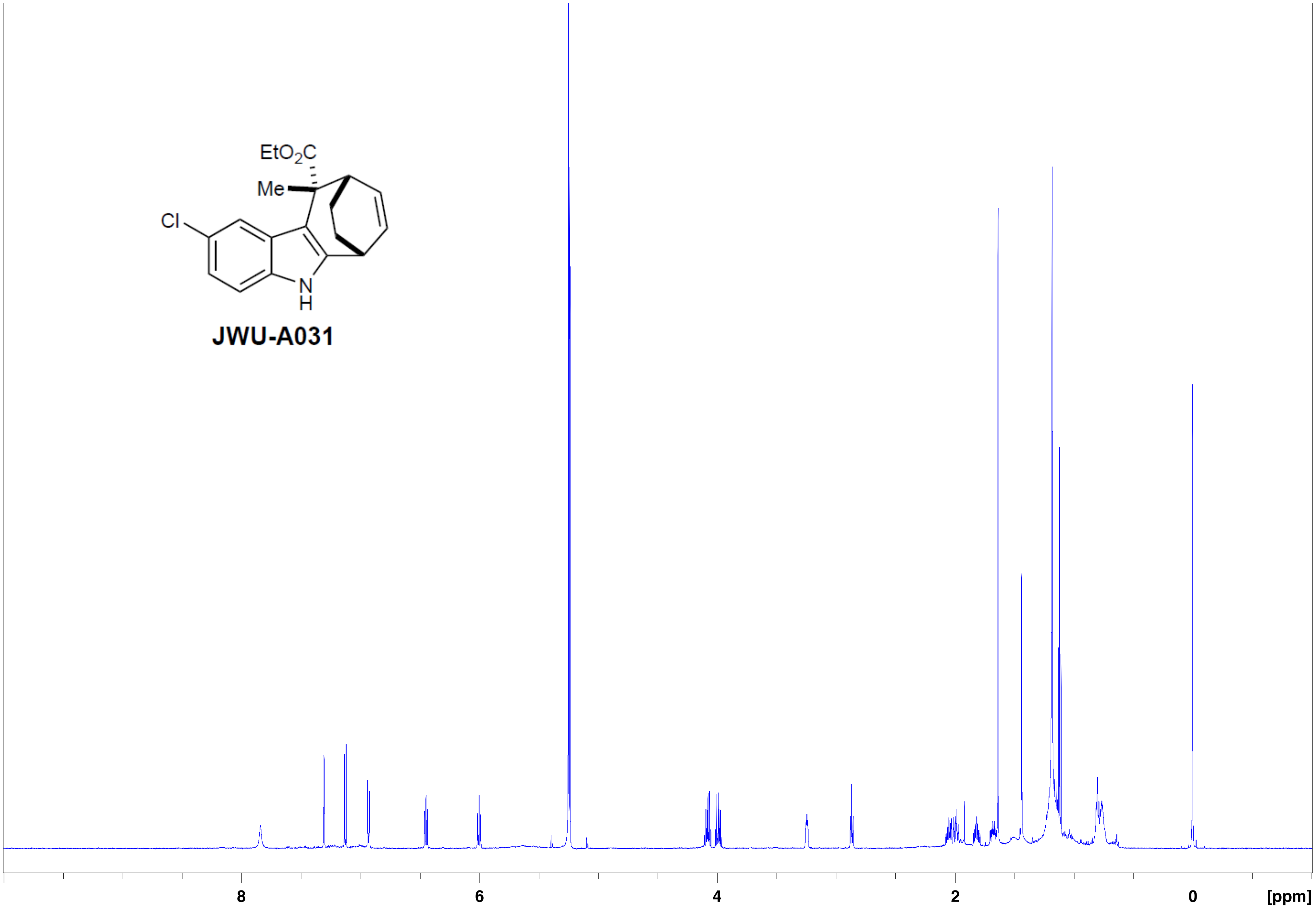

JWU-A031

MT-N3-P154-TT14-15-ETOAc 12 1 N:\b600\wu\data\wu_guest\nmr

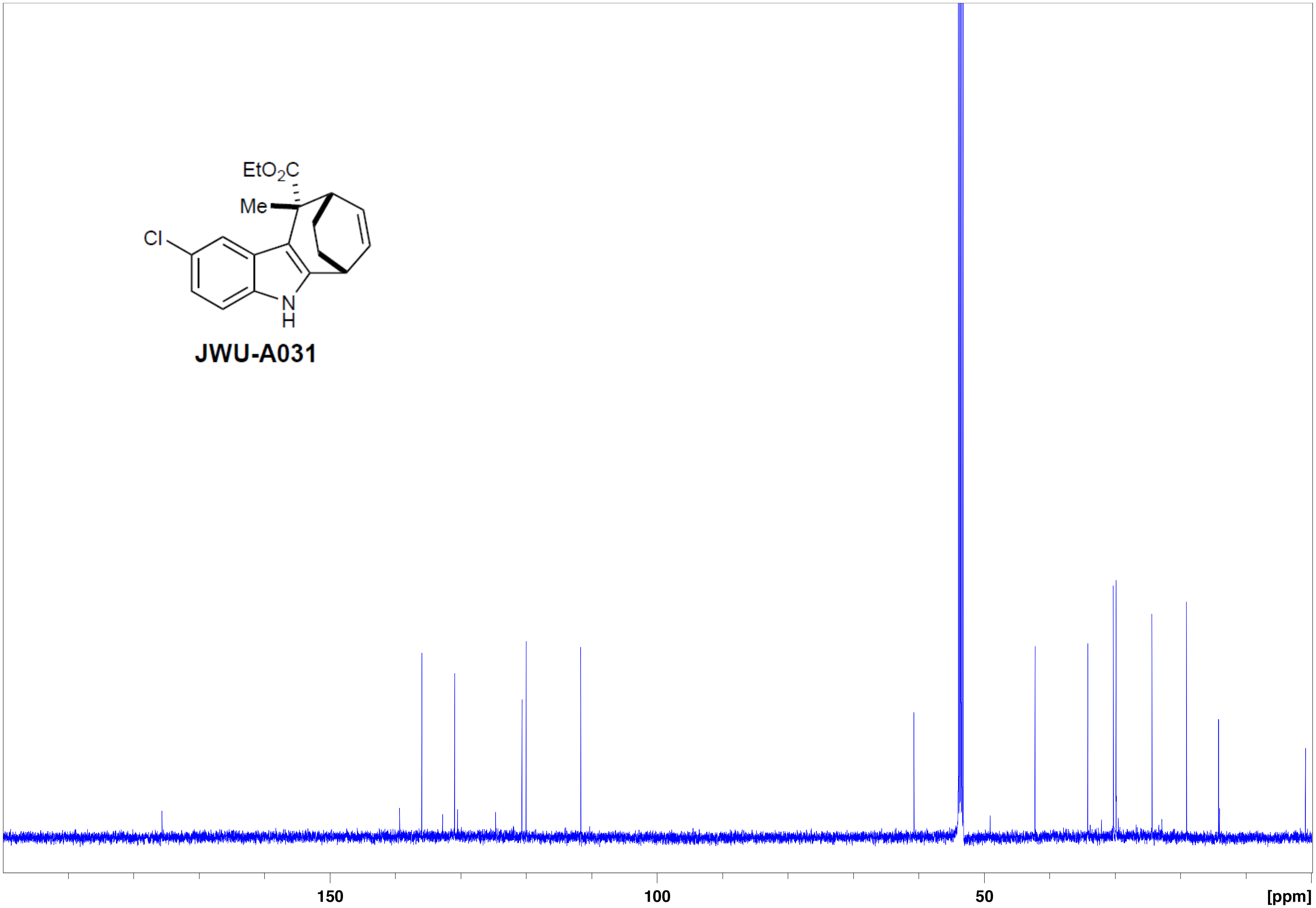

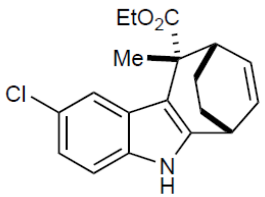
EtO2C
Me
Cl
N
H

JWU-A031

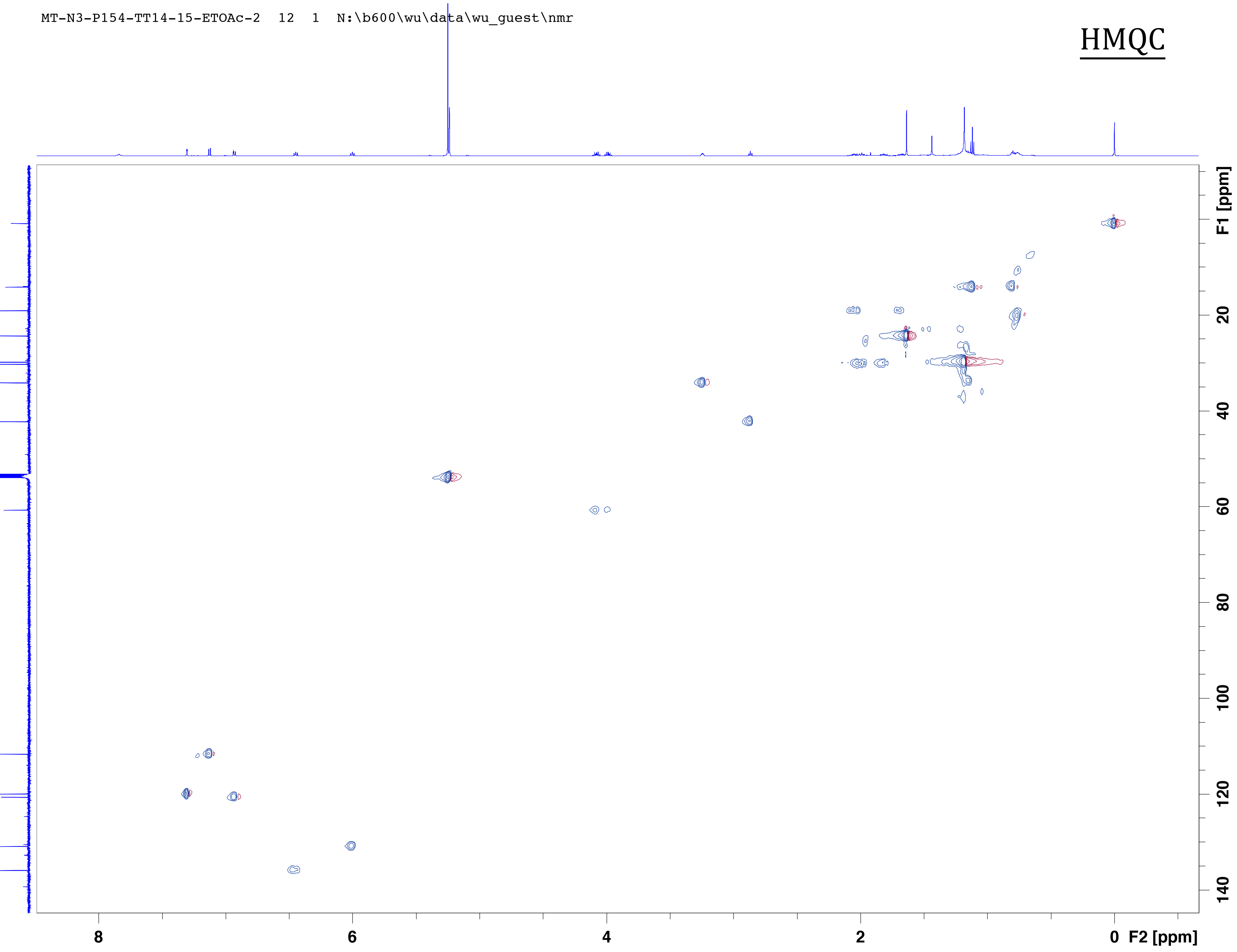
MT-N3-P154-TT14-15-ETOAc-2 12 1 N:\b600\wu\data\wu_guest\nmr
HMQC
F1 [ppm]
F2 [ppm]
20
40
60
80
100
120
140
8
6
4
2
0

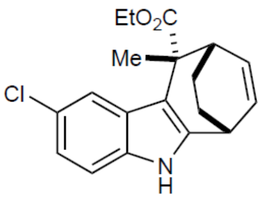

JWU-A031

HMBC

MT-N3-P154-TT14-15-ETOAc-2 11 1 N:\b600\wu\data\wu_guest\nmr

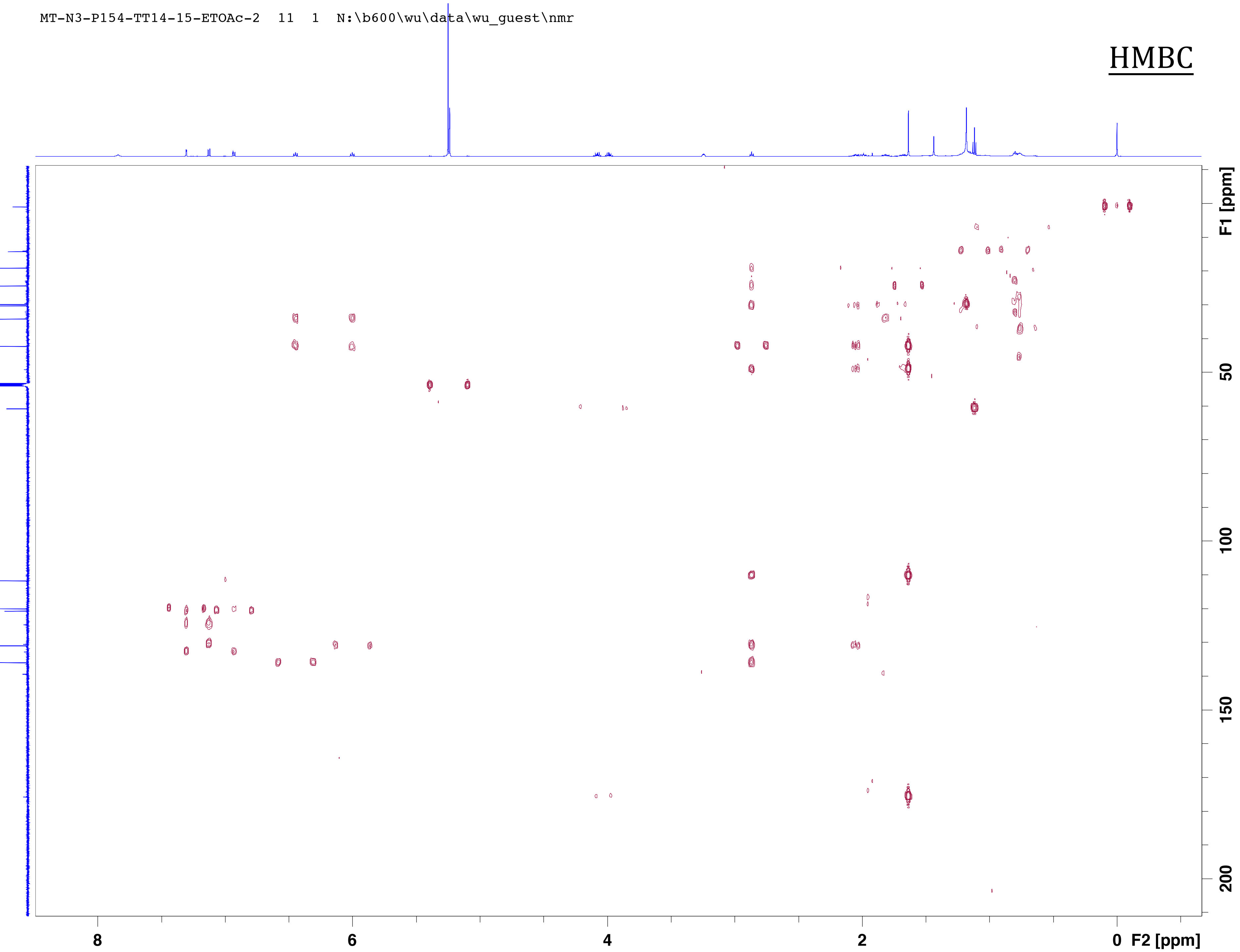

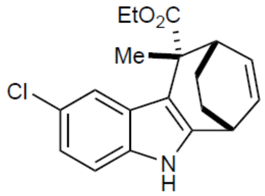

JWU-A031

MT-N3-P154-TT14-15-ETOAc-3   11   1   N:\b600\wu\data\wu_guest\nmr

COSY

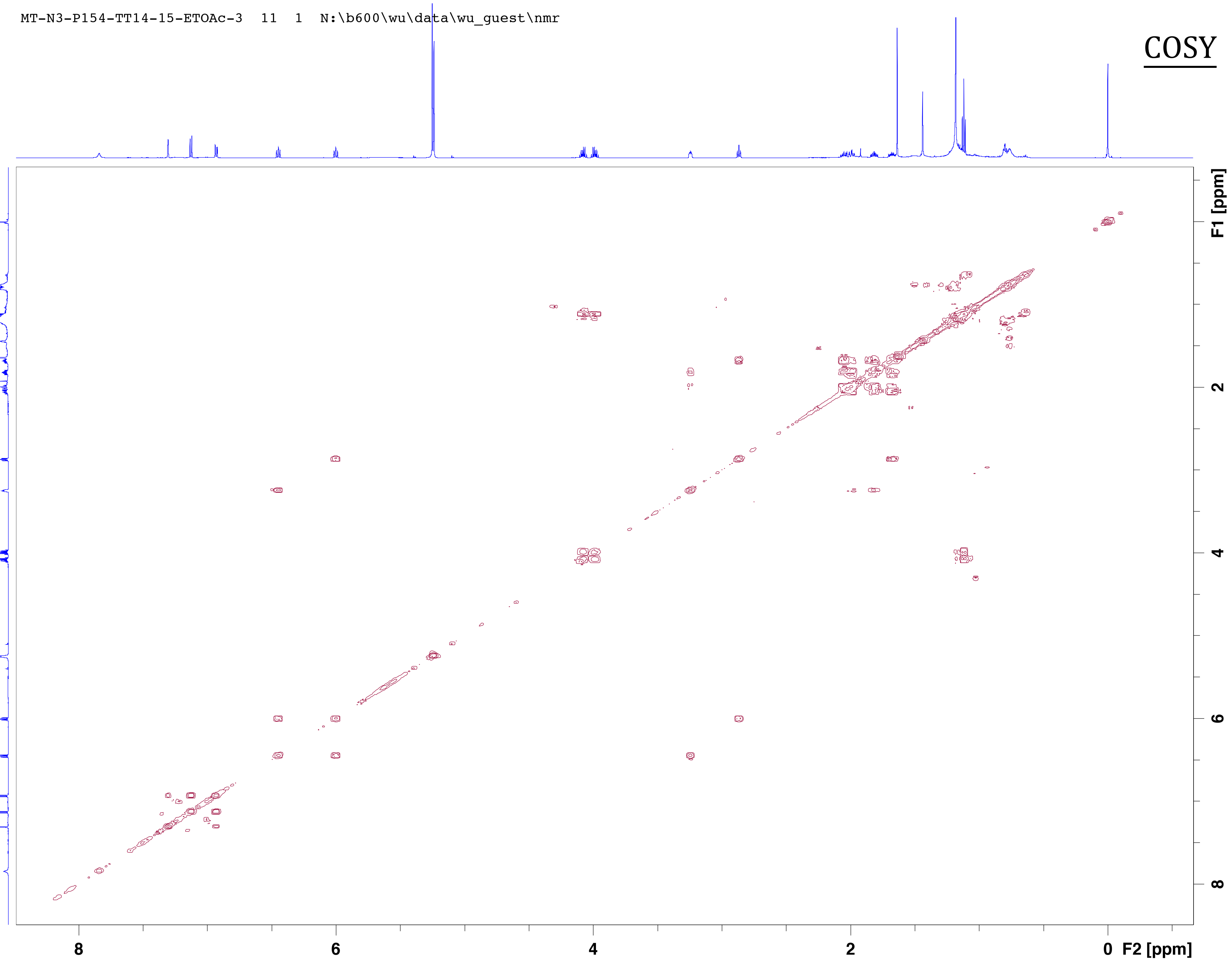

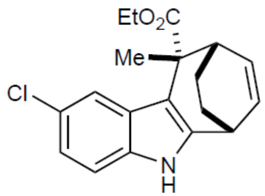

JWU-A031

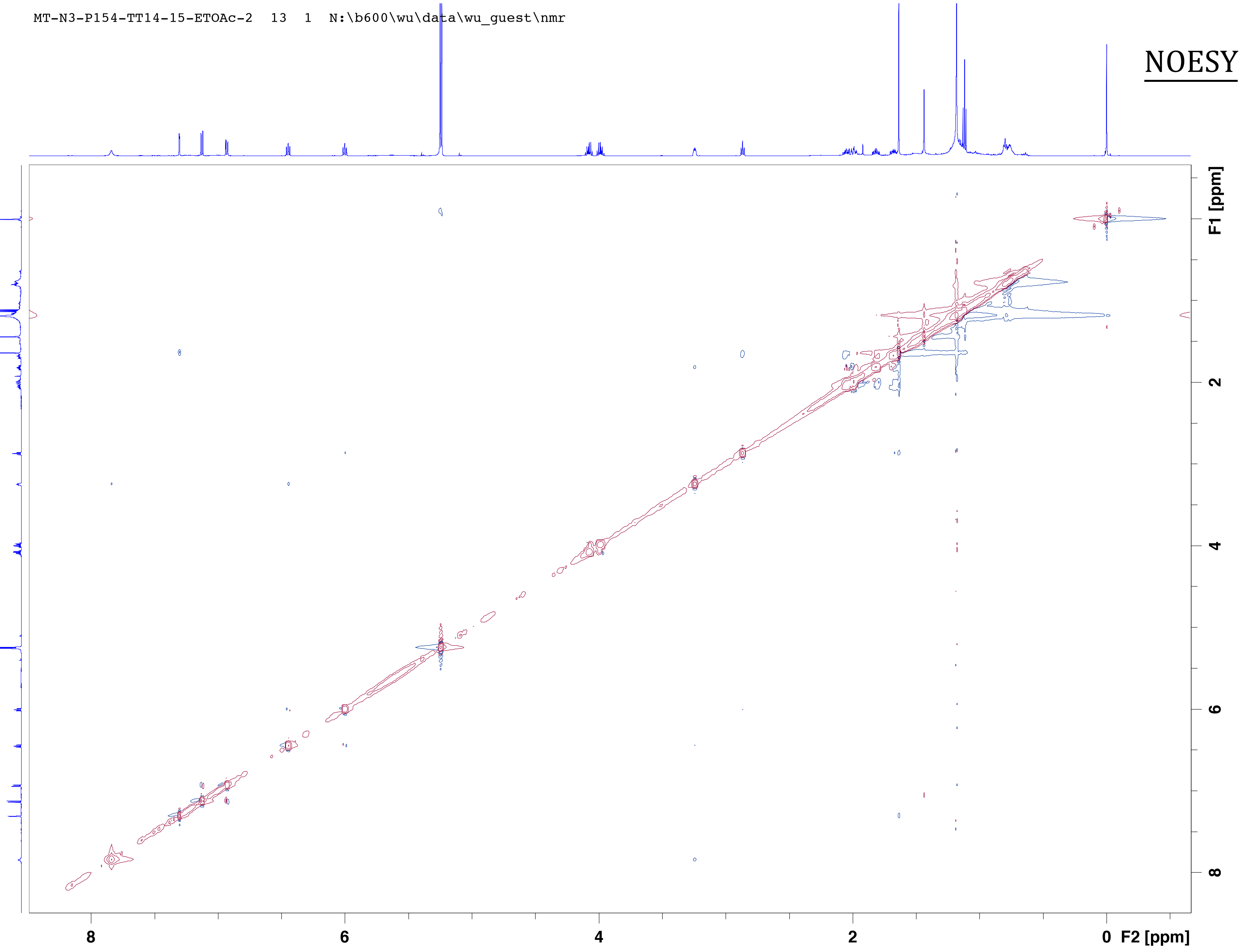
NOESY
MT-N3-P154-TT14-15-ETOAc-2   13   1   N:\b600\wu\data\wu_guest\nmr
F1 [ppm]
F2 [ppm]
8
6
4
2
0

MT-N3-P128-TT22-24-5-6-C 11 1 N:\b600\wu\data\wu_guest\nmr

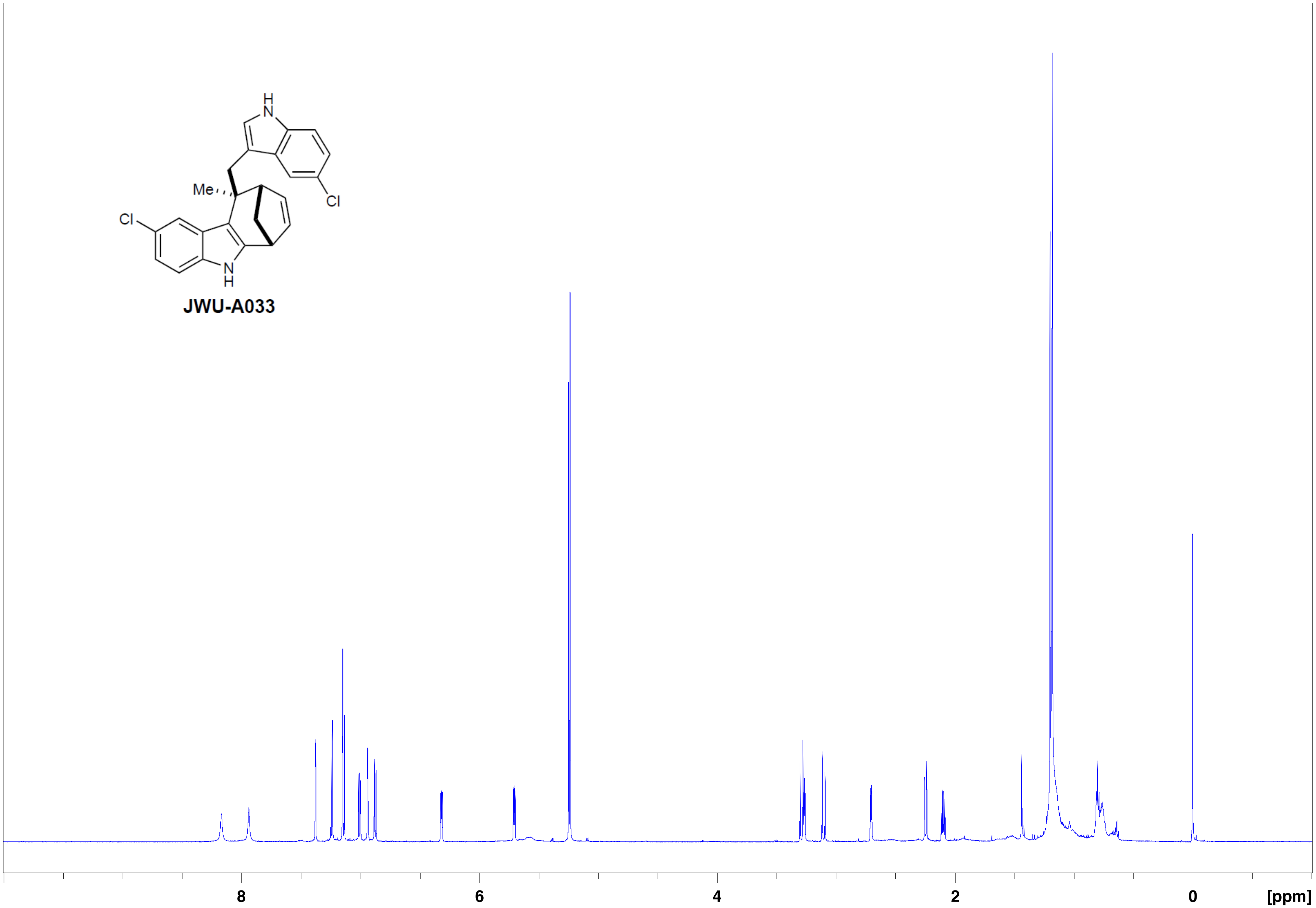

MT-N3-P128-TT22-24-5-6-C   12   1   N:\b600\wu\data\wu_guest\nmr

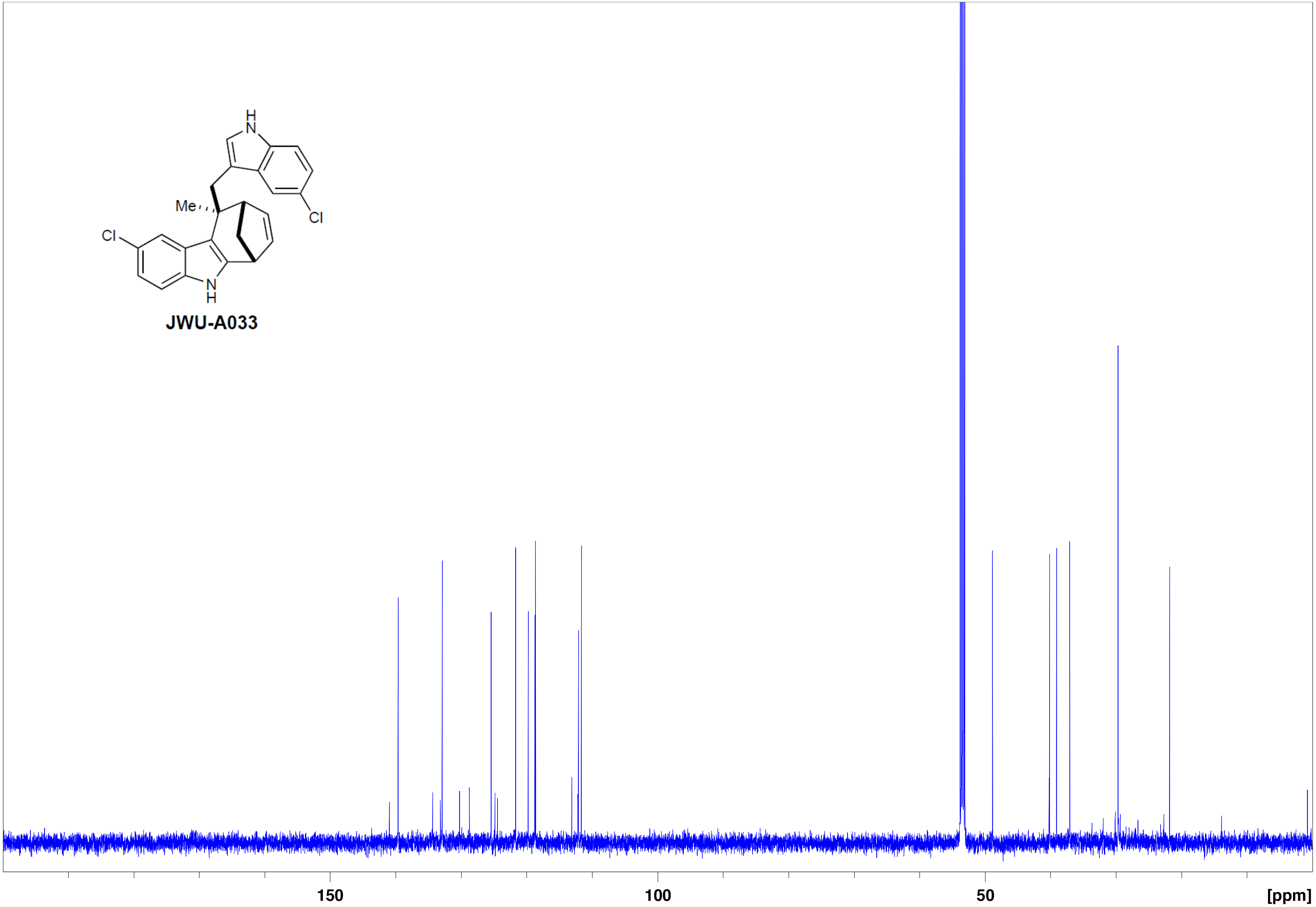

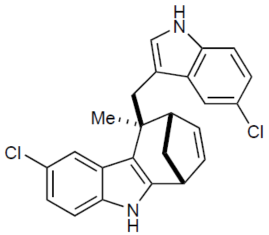

JWU-A033

MT-N3-P128-TT22-24-5-6-C 14 1 N:\b600\wu\data\wu_guest\nmr

HMQC

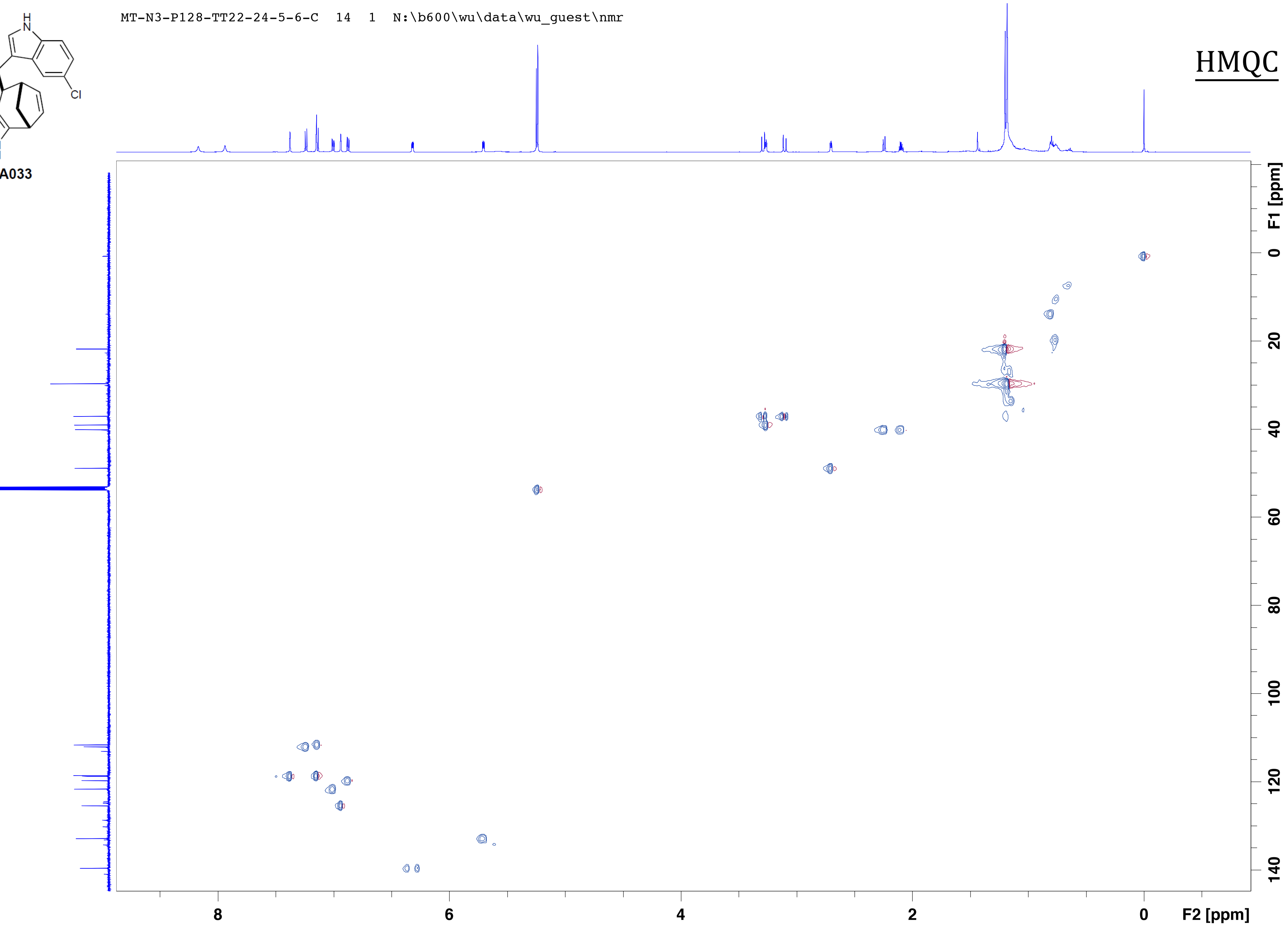

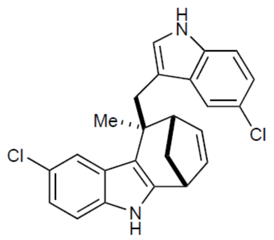

JWU-A033

MT-N3-P128-TT22-24-5-6-C 15 1 N:\b600\wu\data\wu_guest\nmr

HMBC

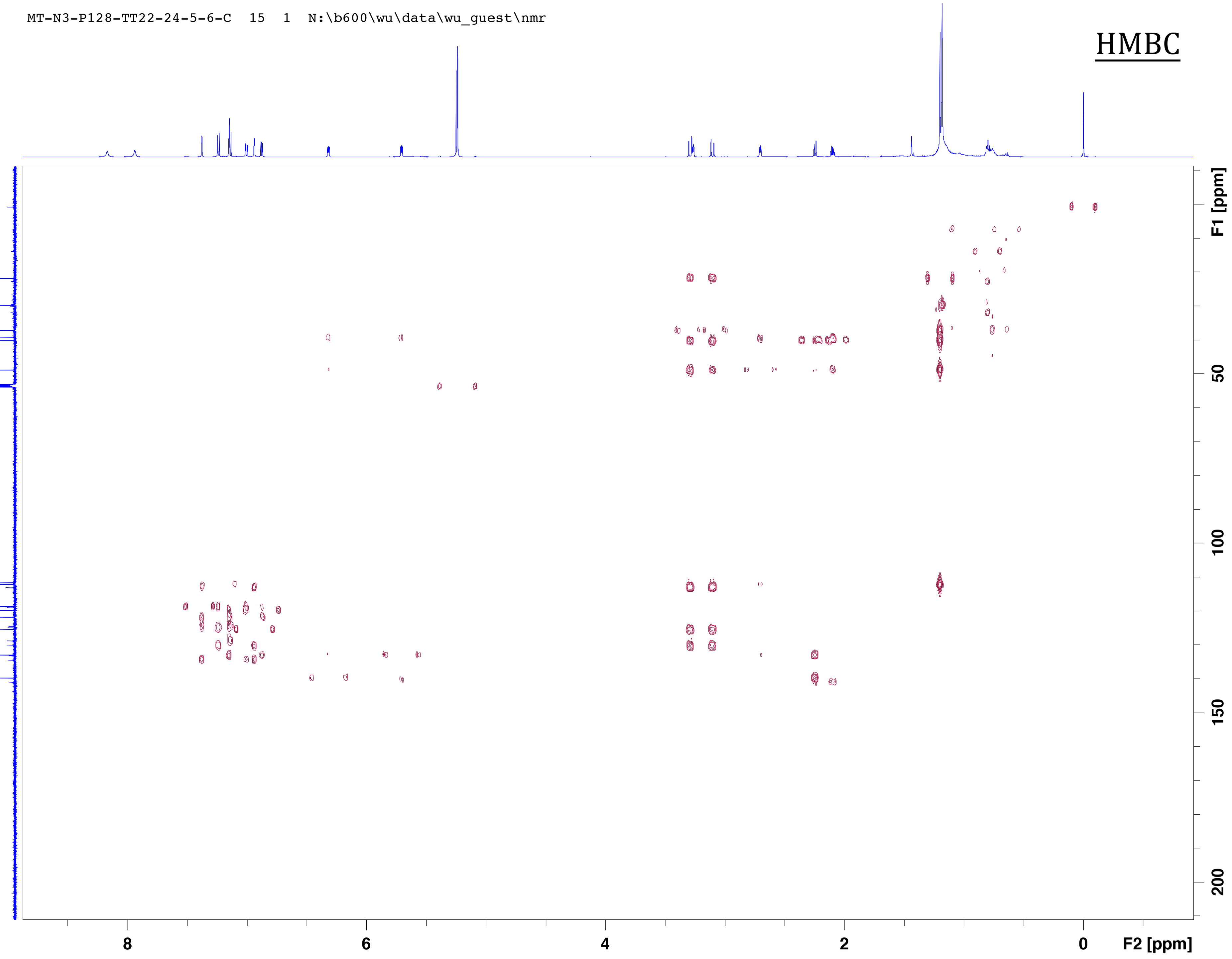

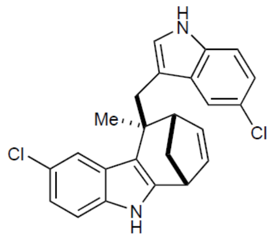

JWU-A033

MT-N3-P128-TT22-24-5-6-C 16 1 N:\b600\wu\data\wu_guest\nmr

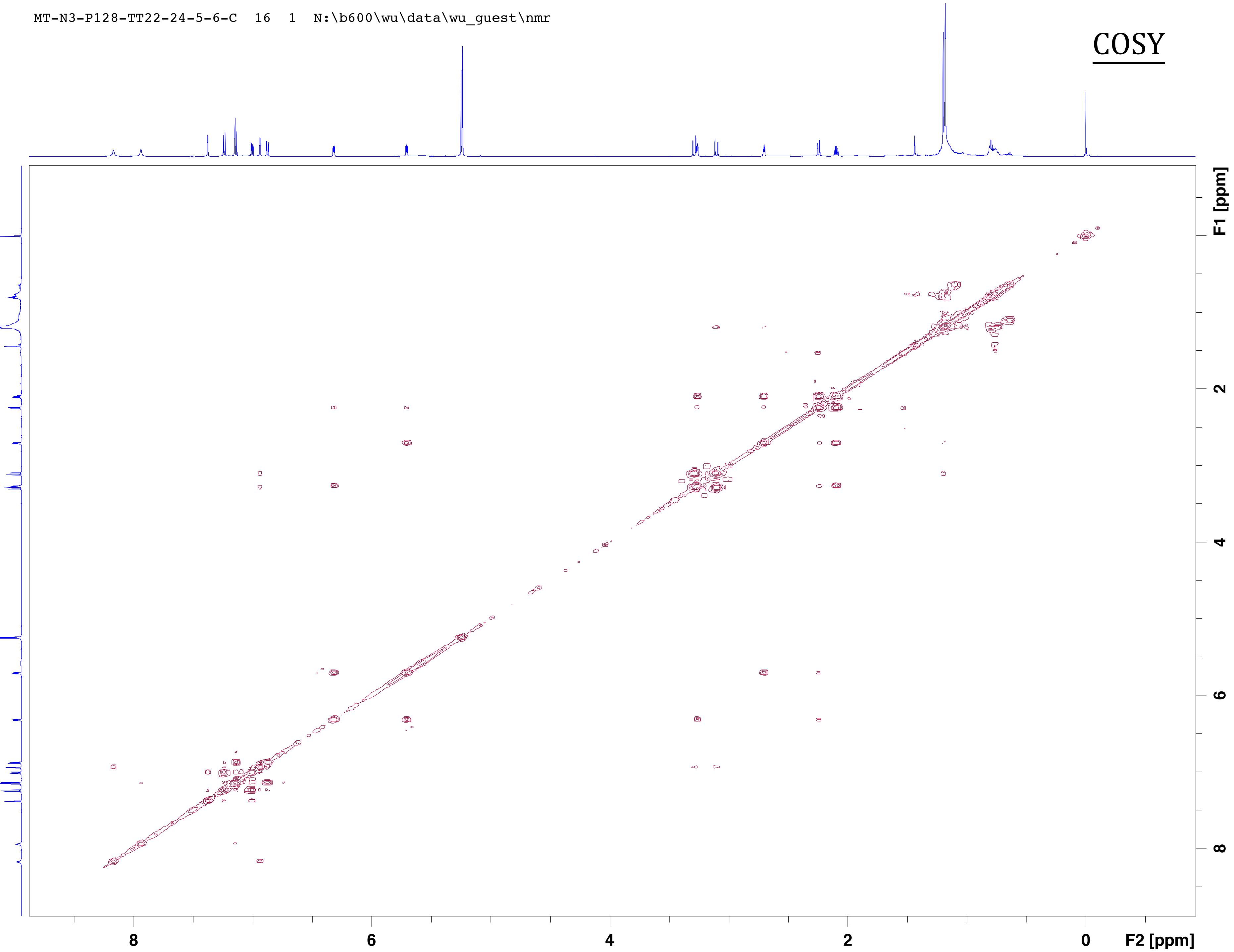

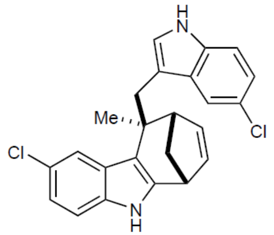

JWU-A033

MT-N3-P128-TT22-24-5-6-C 17 1 N:\b600\wu\data\wu_guest\nmr

NOESY

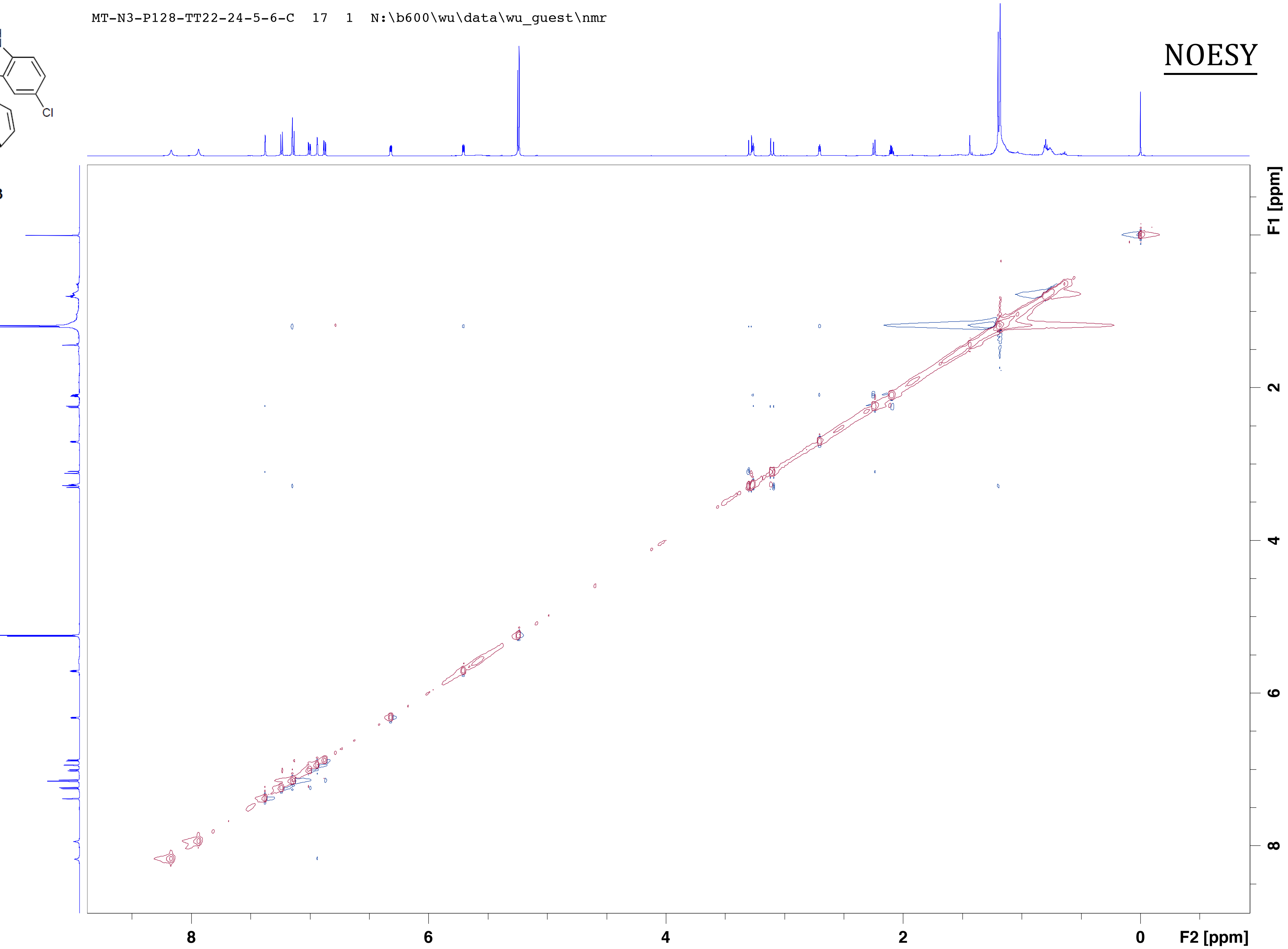

MT-N3-P114-TT12-17-10 10 1 N:\b600\wu\data\wu_guest\nmr

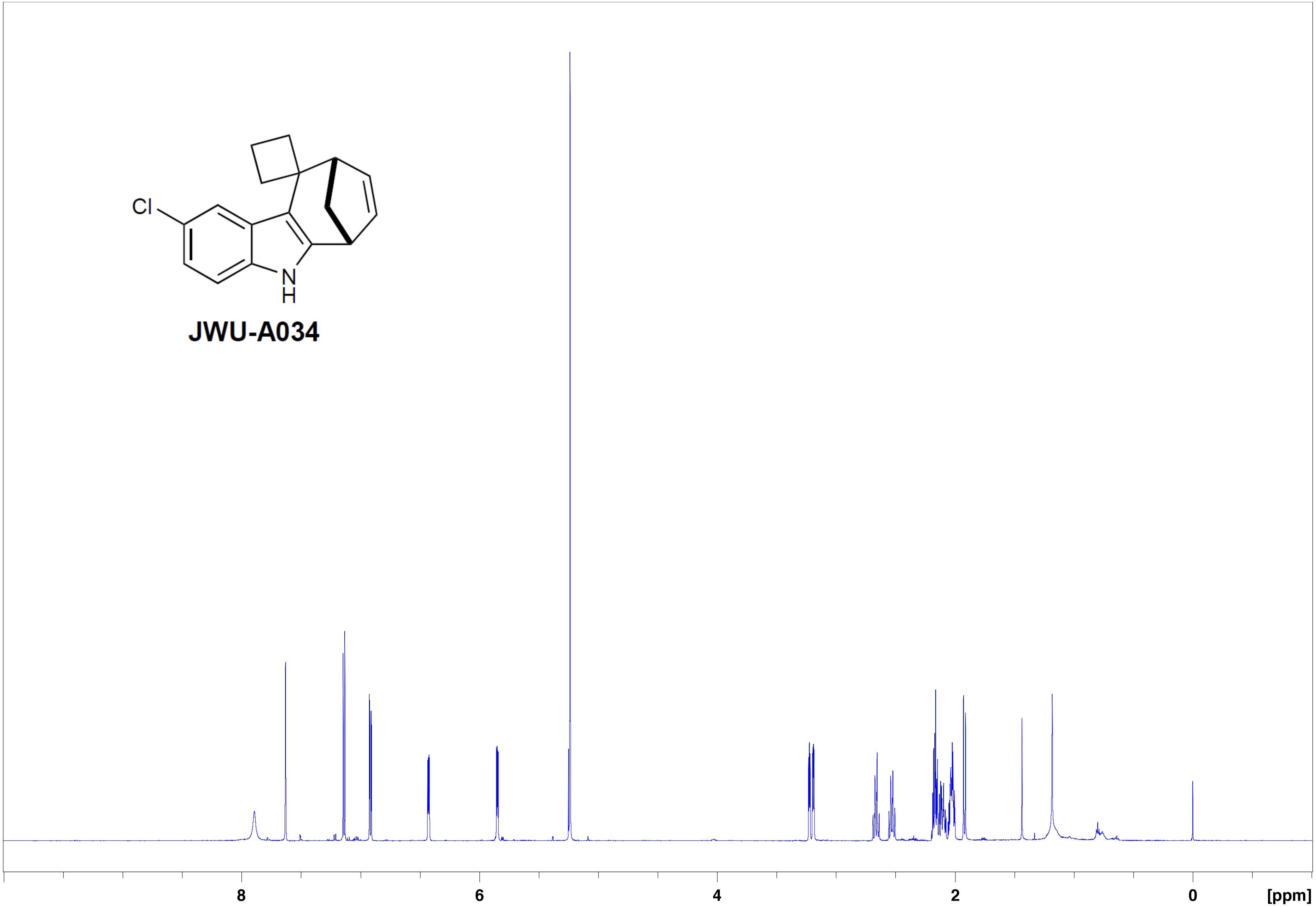

MT-N3-P114-TT12-17-10 12 1 N:\b600\wu\data\wu_guest\nmr

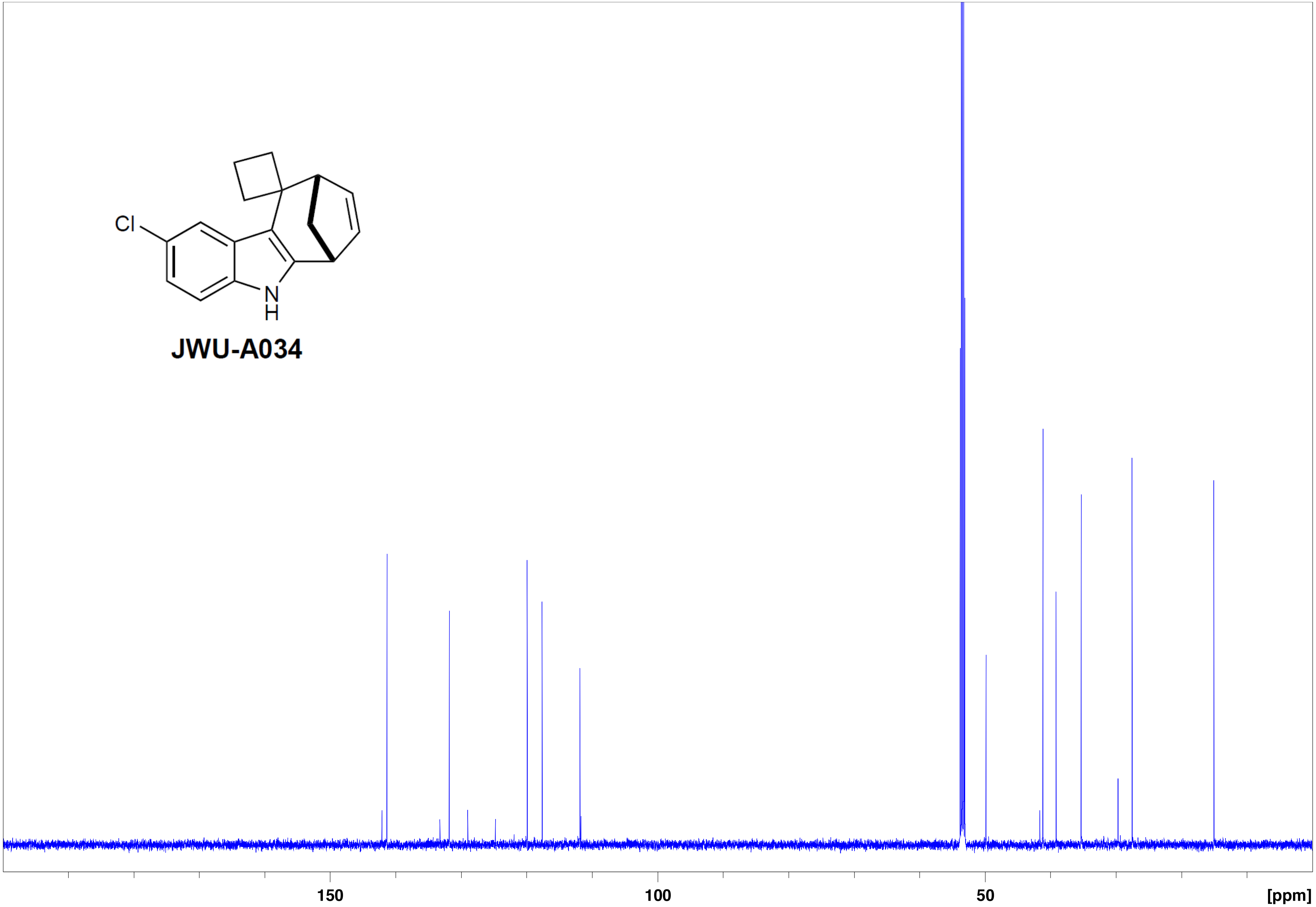

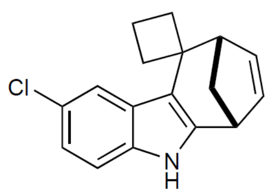
Cl
N
H

JWU-A034

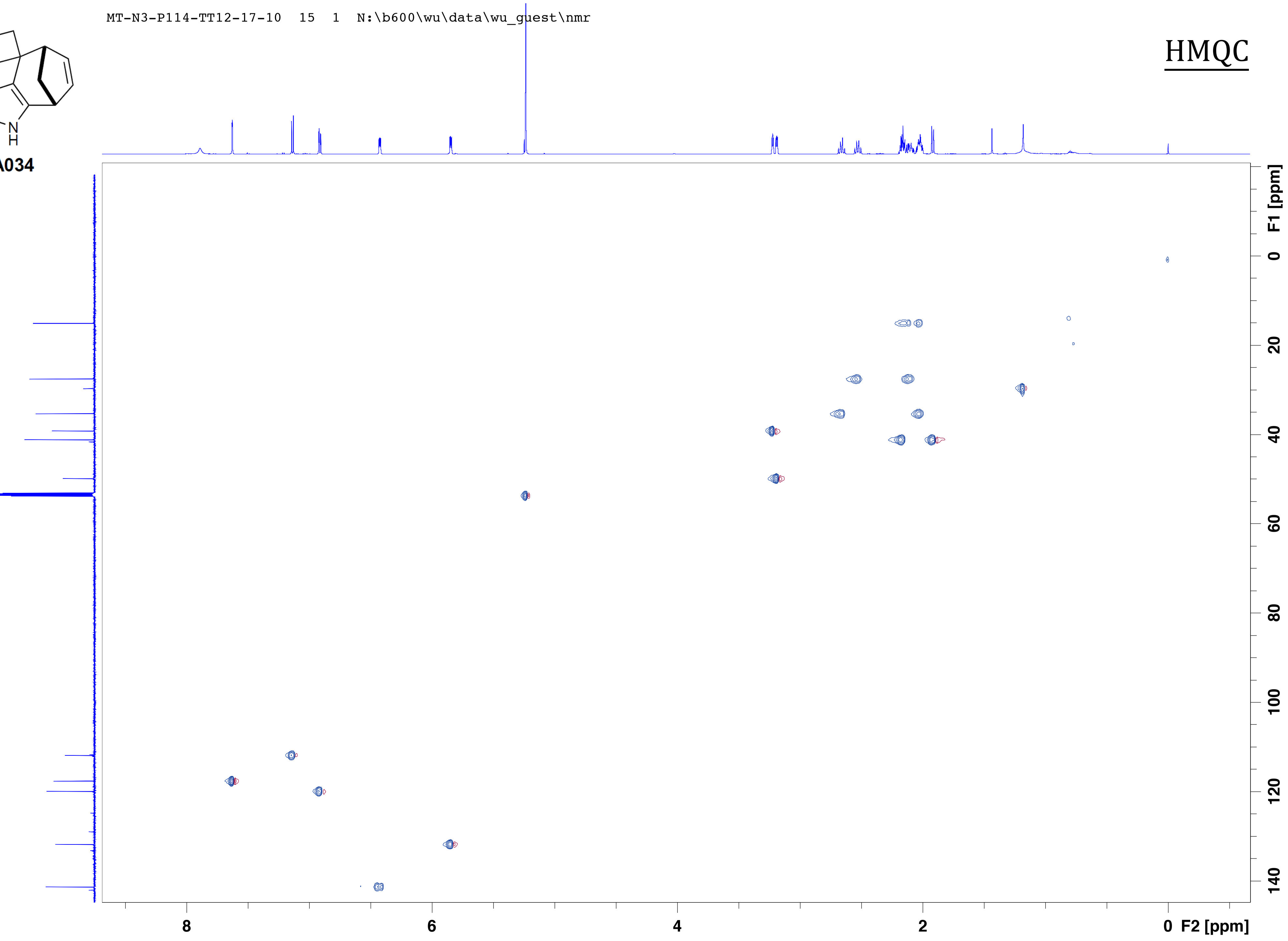
MT-N3-P114-TT12-17-10 15 1 N:\b600\wu\data\wu_guest\nmr
HMQC
F1 [ppm]
0
20
40
60
80
100
120
140
8
6
4
2
0 F2 [ppm]

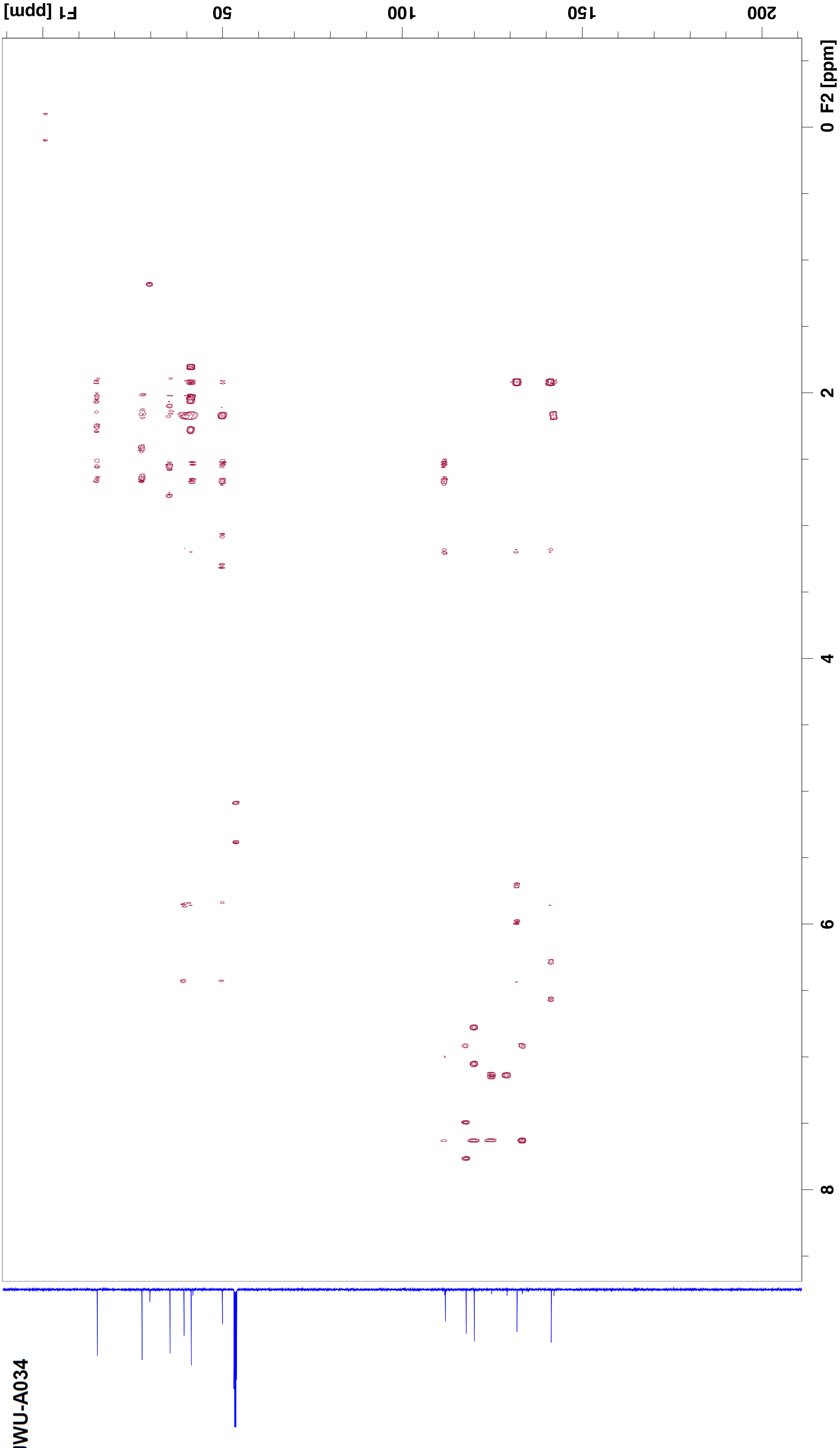

HMBC
MT-N3-P114-TT12-17-10 14 1 N:\b600\wu\data\wu_guest\nmr
F1 [ppm]
50
100
150
200
F2 [ppm]
0
2
4
6
8

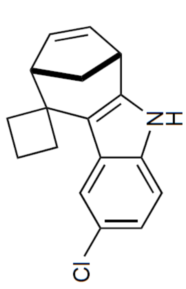

NH
Cl

JWU-A034

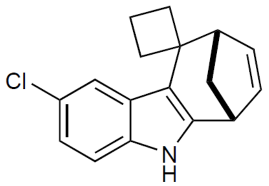

JWU-A034

MT-N3-P114-TT12-17-10   16   1   N:\b600\wu\data\wu_guest\nmr

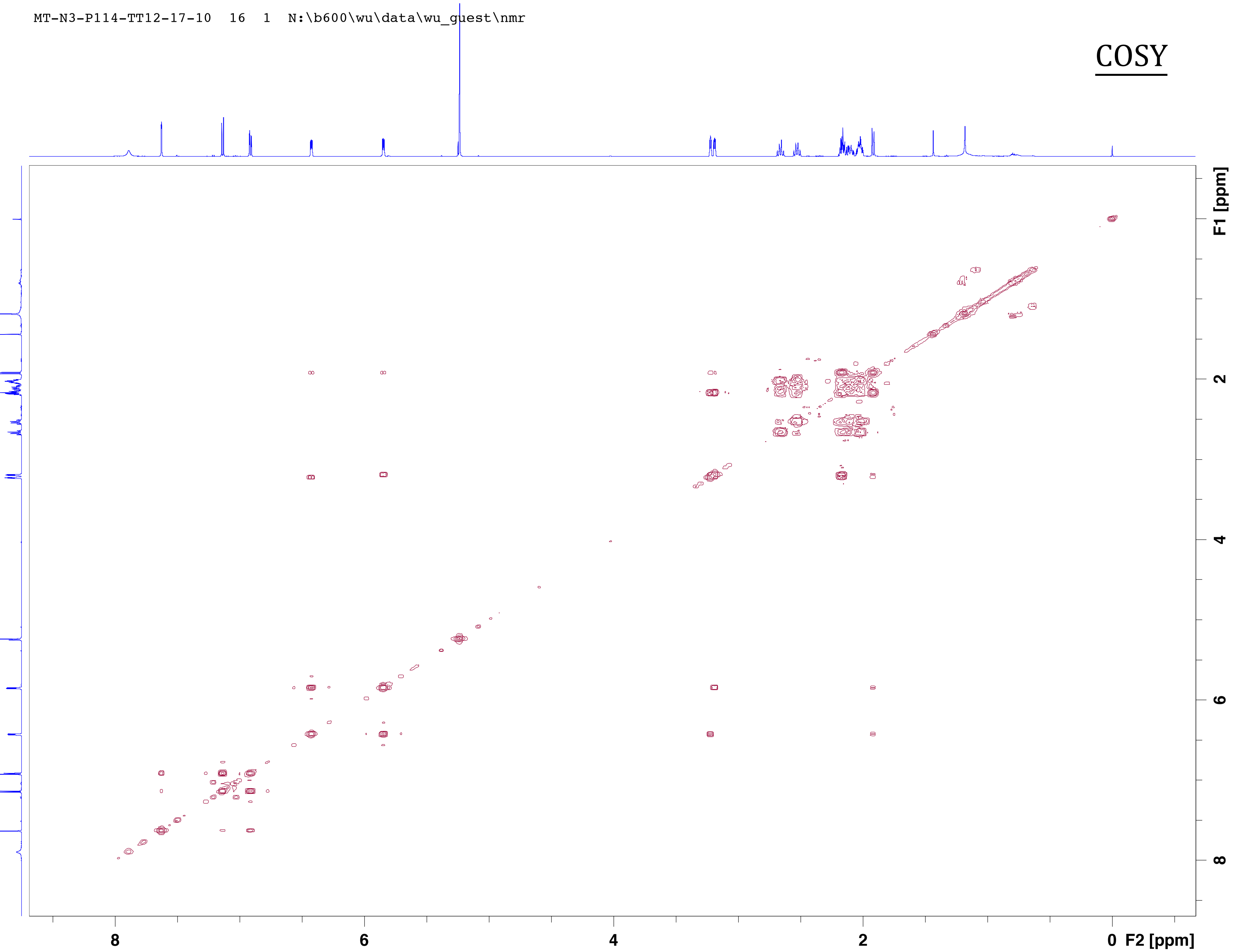

MT-N3-P30-TT19-7-8   11   1   "I:\3+2 Products"

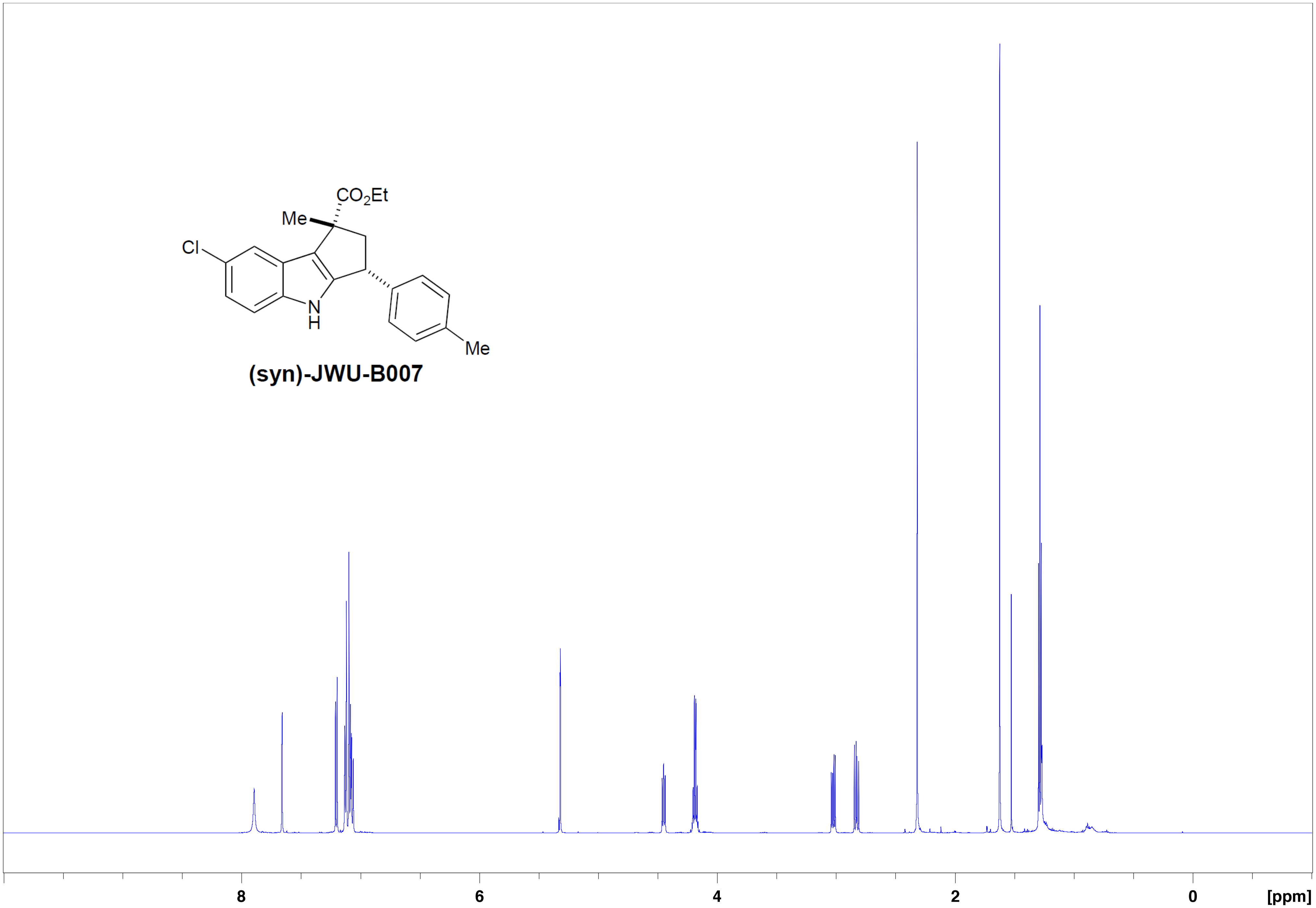

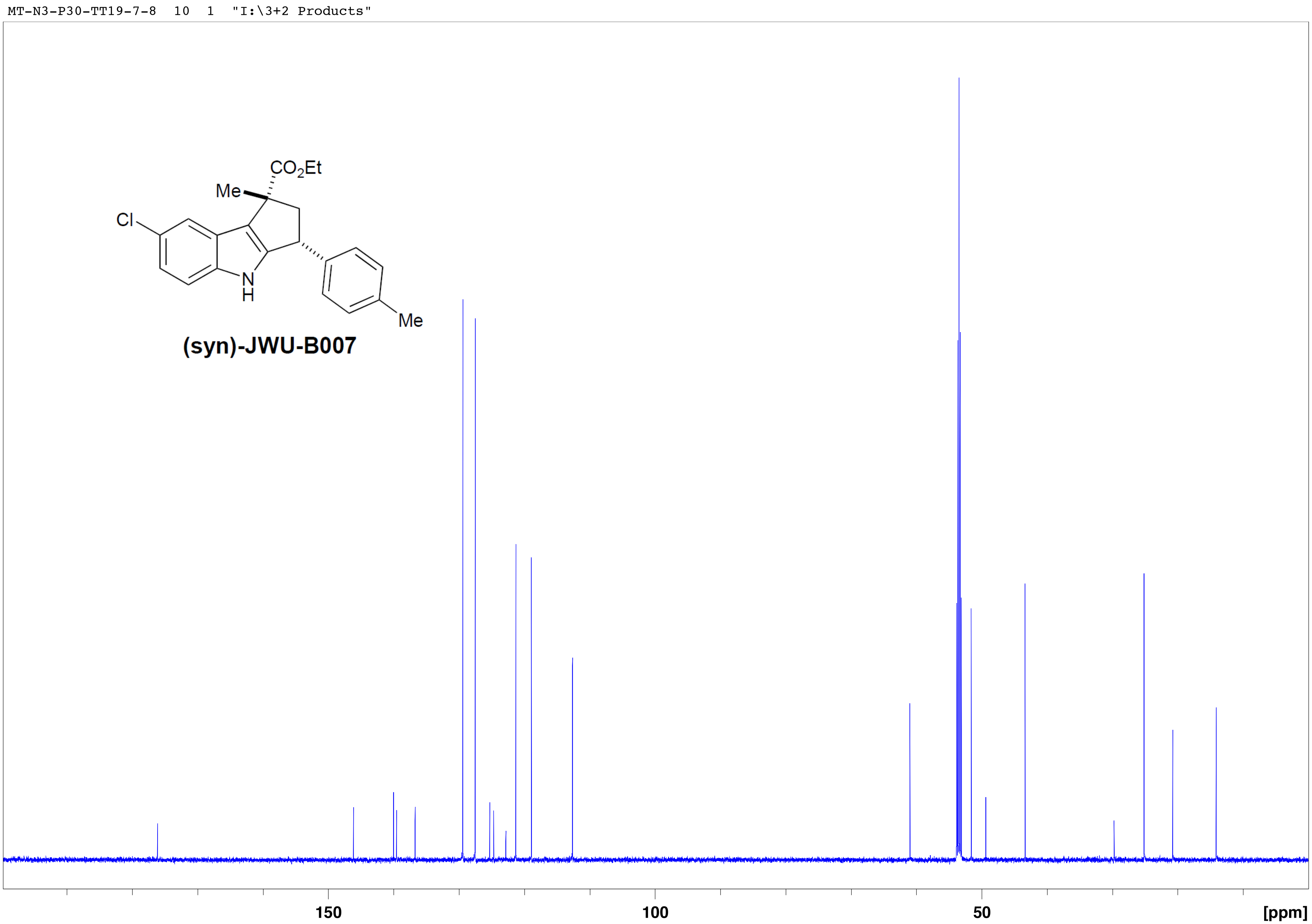
MT-N3-P30-TT19-7-8 10 1 "I:\3+2 Products"
CO2Et
Me
Cl
N
H
Me
(syn)-JWU-B007
150
100
50
[ppm]

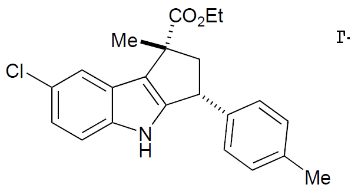
CO2Et
Me
Cl
N
H
Me

(syn)-JWU-B007

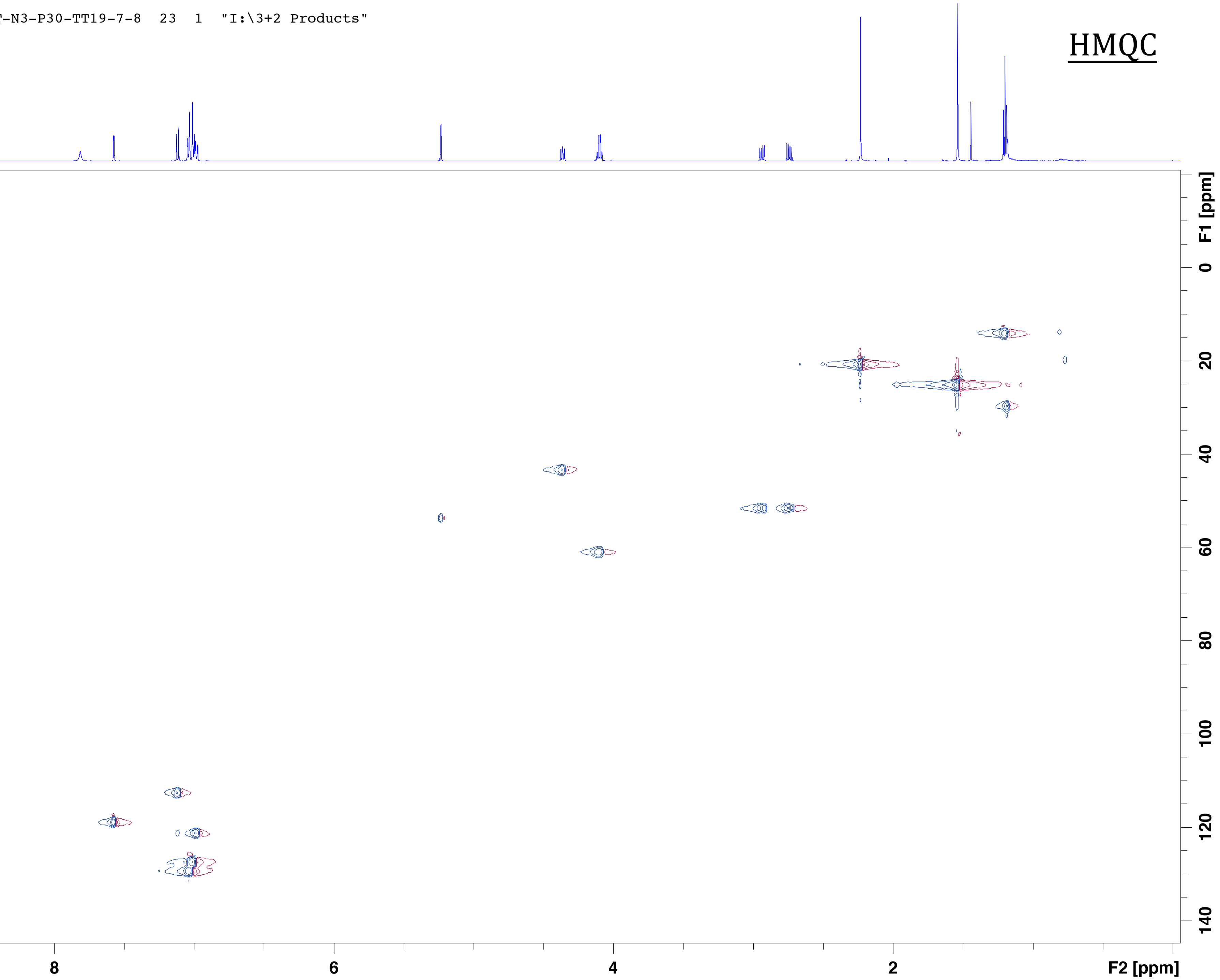
I-N3-P30-TT19-7-8 23 1 "I:\3+2 Products"
HMQC
F1 [ppm]
F2 [ppm]
0
20
40
60
80
100
120
140
8
6
4
2

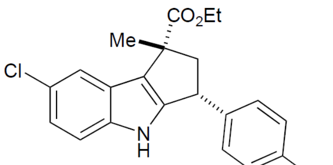

(syn)-JWU-B007

T-N3-P30-TT19-7-8 24 1 "I:\3+2 Products"

HMBC

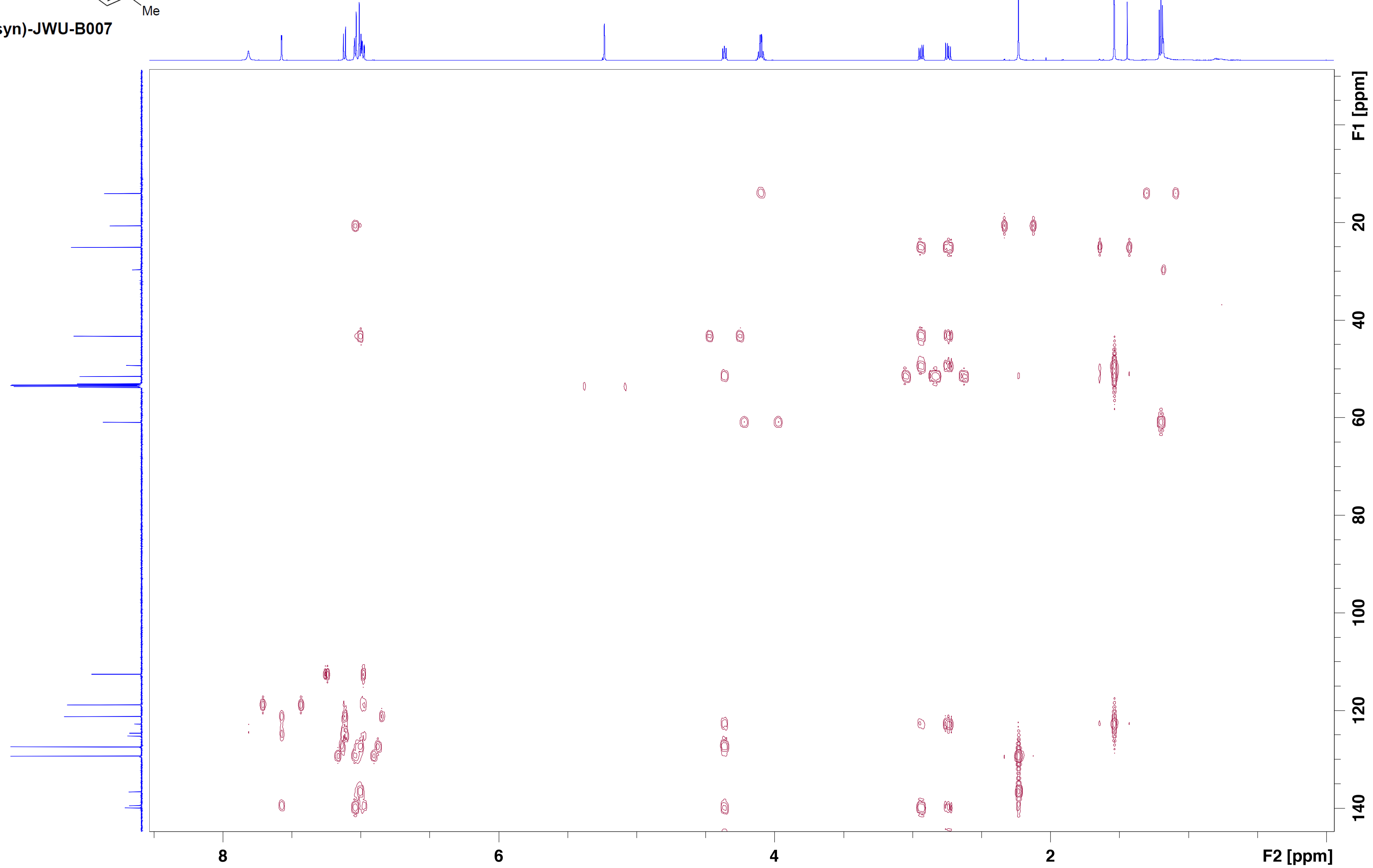

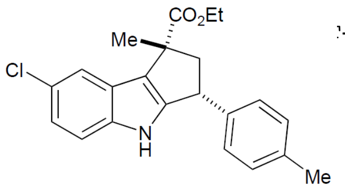

(syn)-JWU-B007

COSY

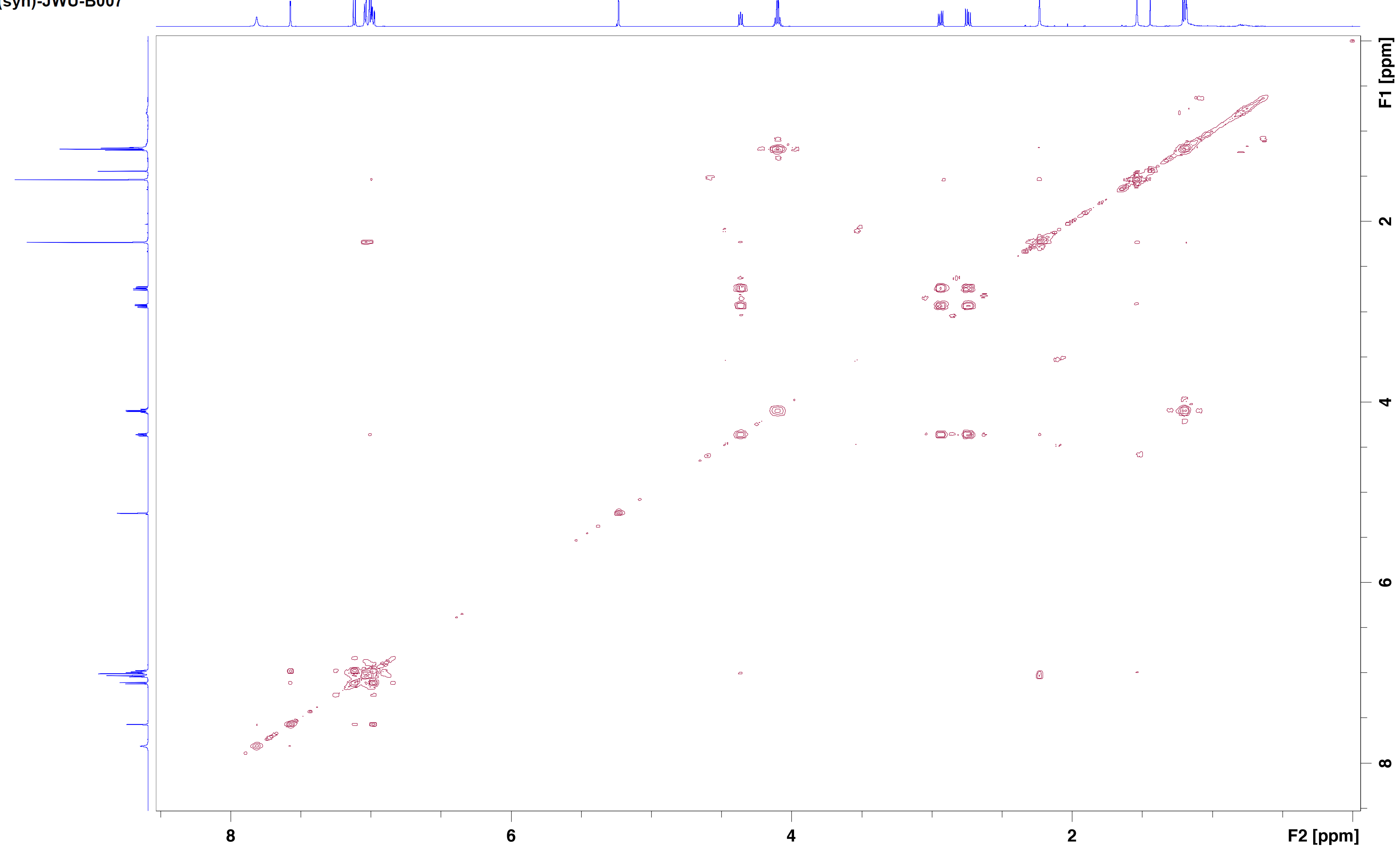

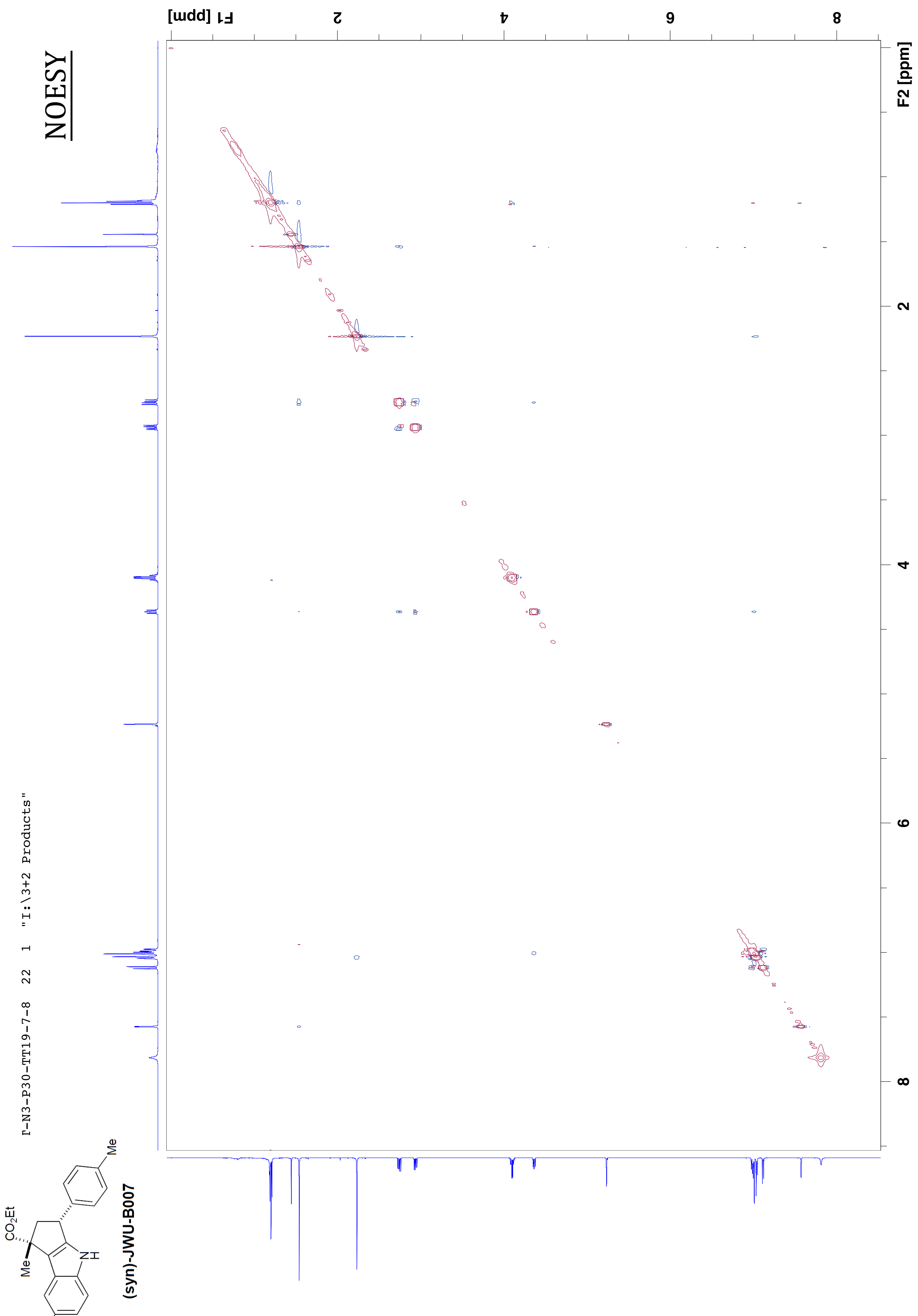
NOESY
(syn)-JWU-B007
I-N3-P30-TT19-7-8 22 1 "I:\3+2 Products"
F1 [ppm]
F2 [ppm]

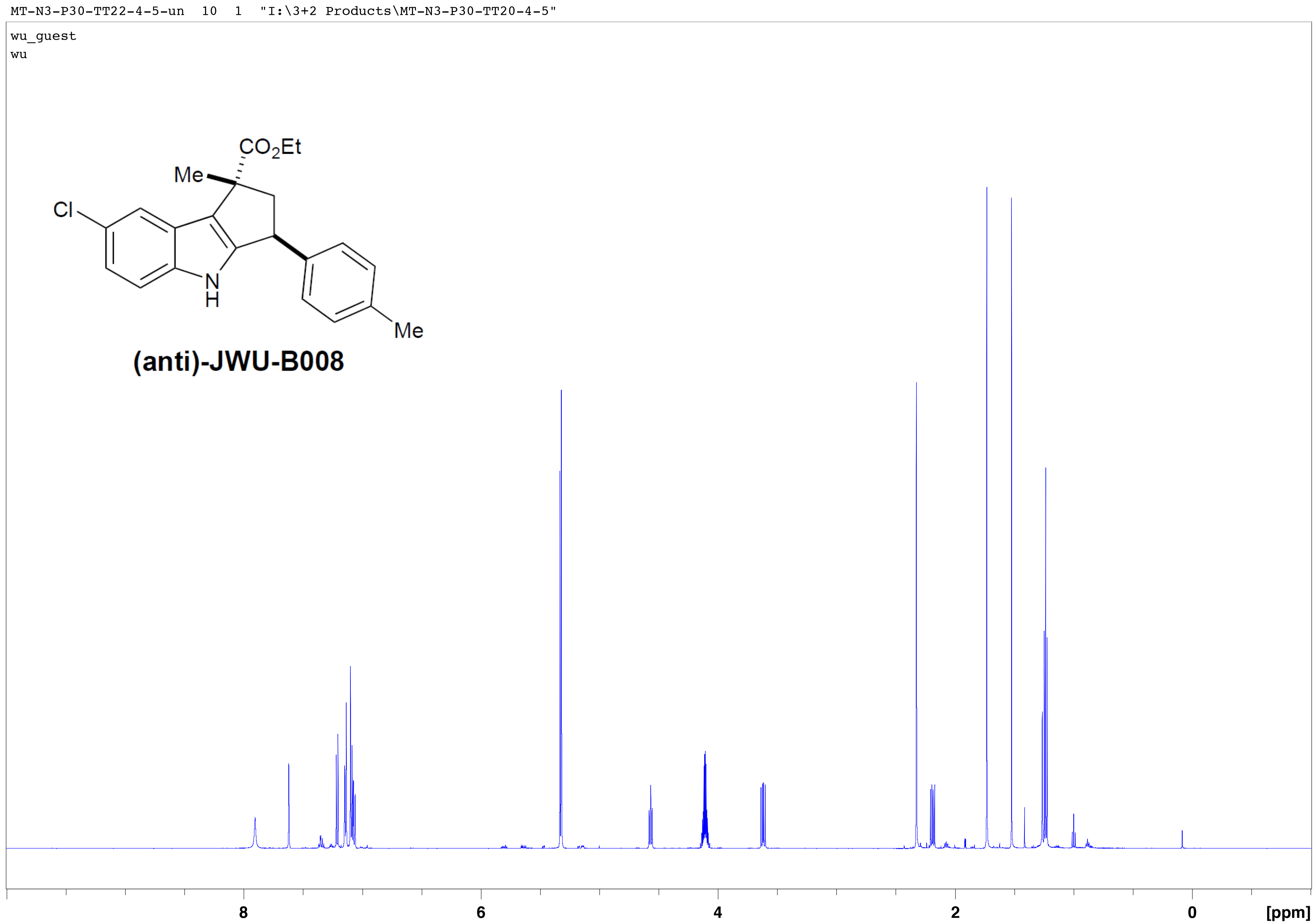
MT-N3-P30-TT22-4-5-un 10 1 "I:\3+2 Products\MT-N3-P30-TT20-4-5"
wu_guest
wu
CO2Et
Me
Cl
N
H
Me
(anti)-JWU-B008
8
6
4
2
0
[ppm]

MT-N3-P30-TT22-4-5-carbon 11 1 "I:\3+2 Products\MT-N3-P30-TT20-4-5"

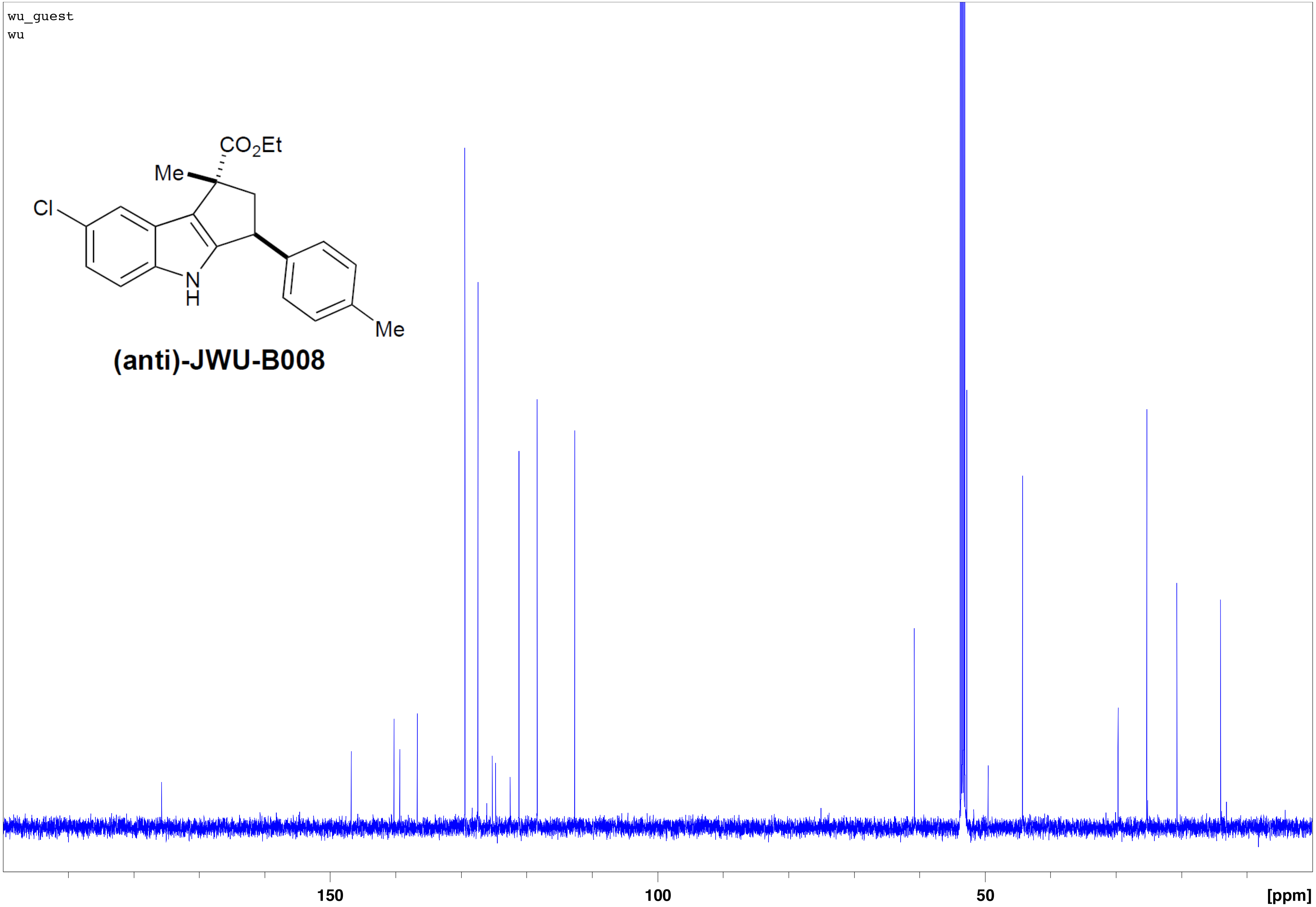

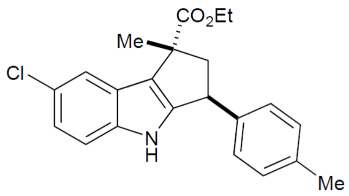

(anti)-JWU-B008

HMQC

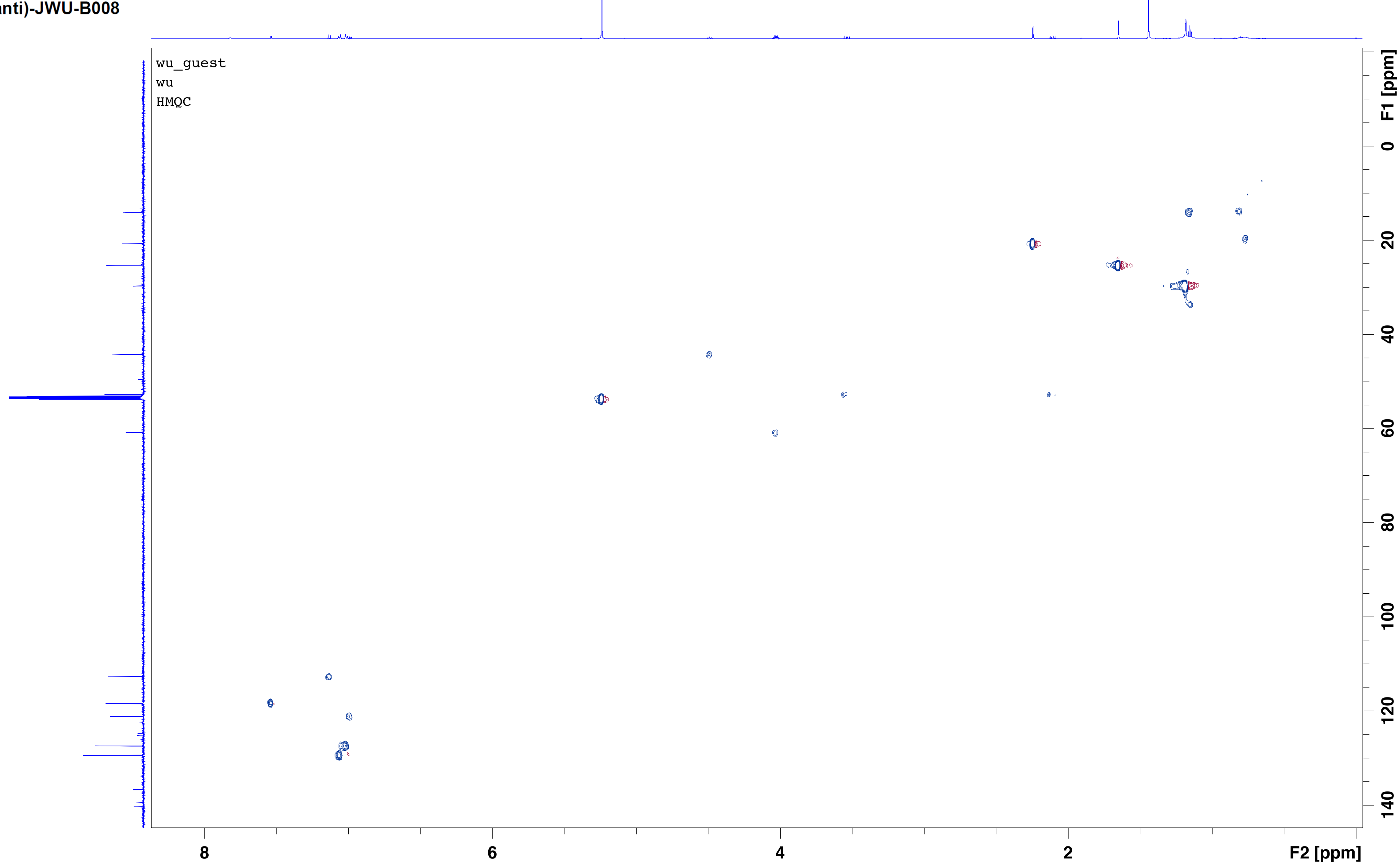

HMBC

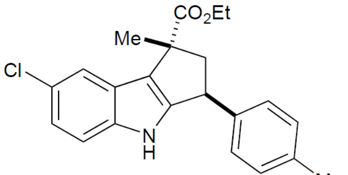

(anti)-JWU-B008

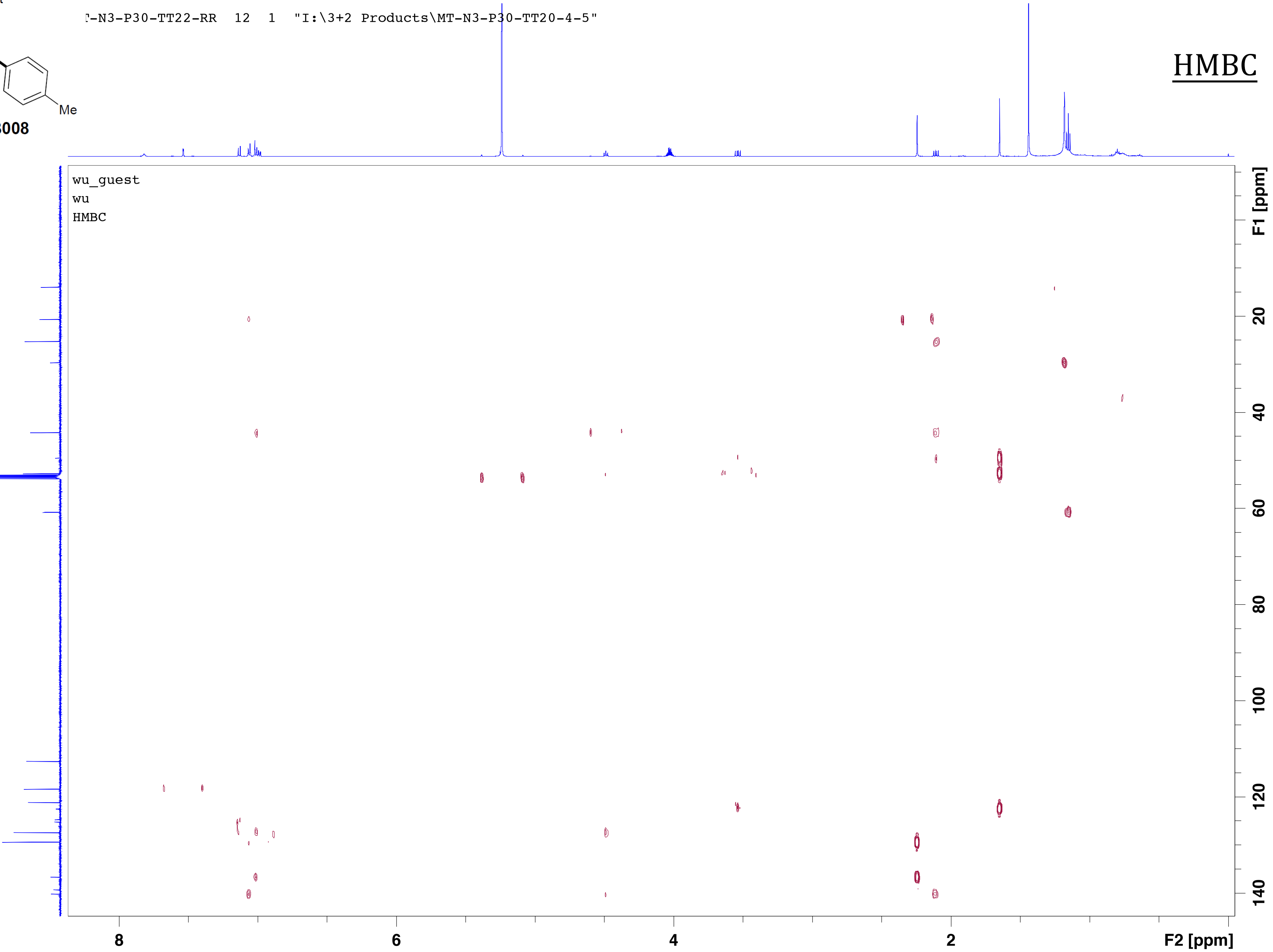

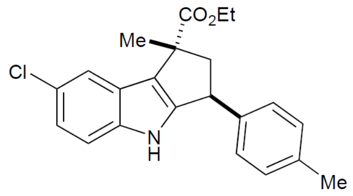

(anti)-JWU-B008

COSY

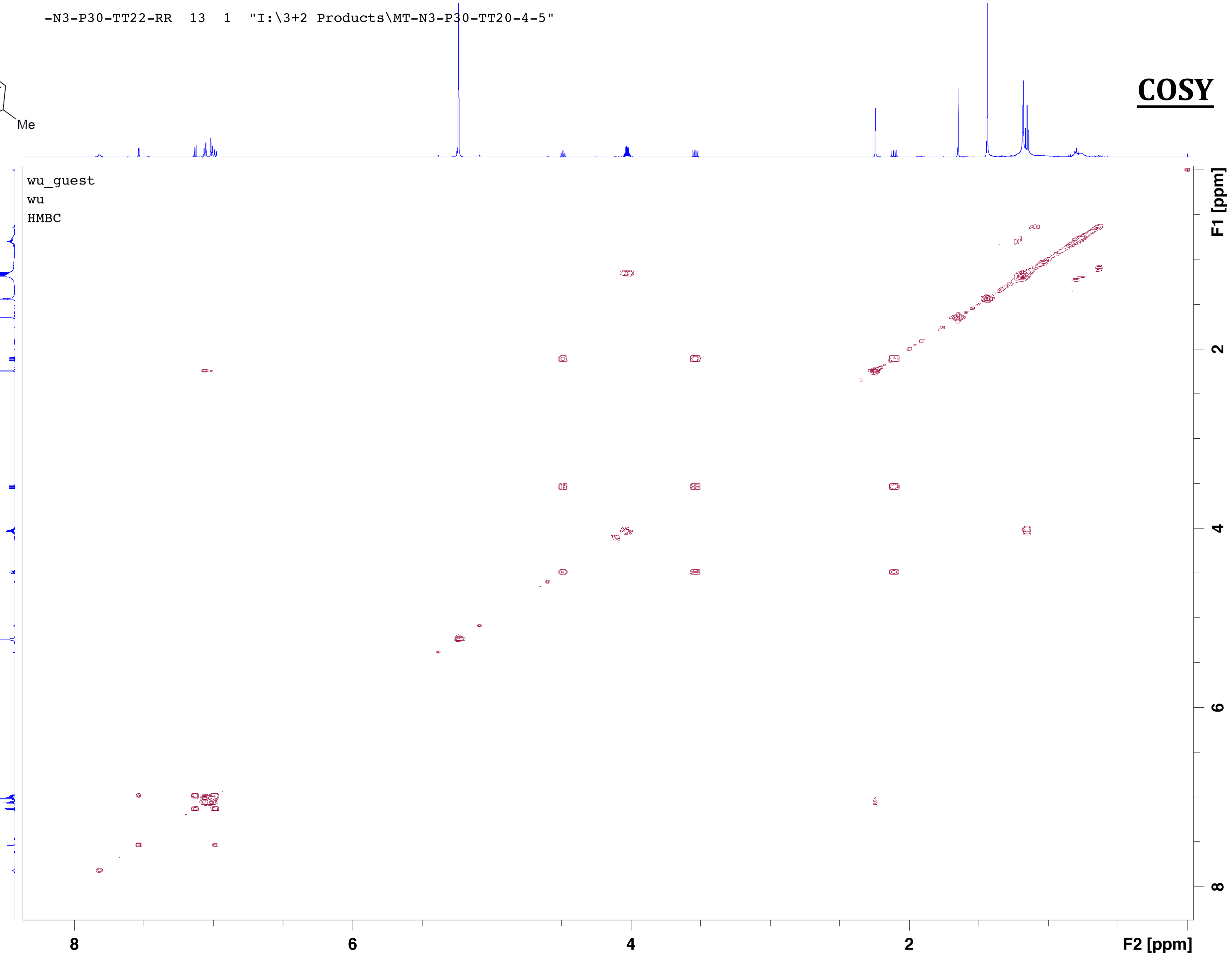

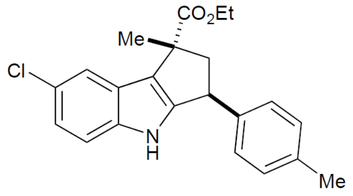

(anti)-JWU-B008

NOESY

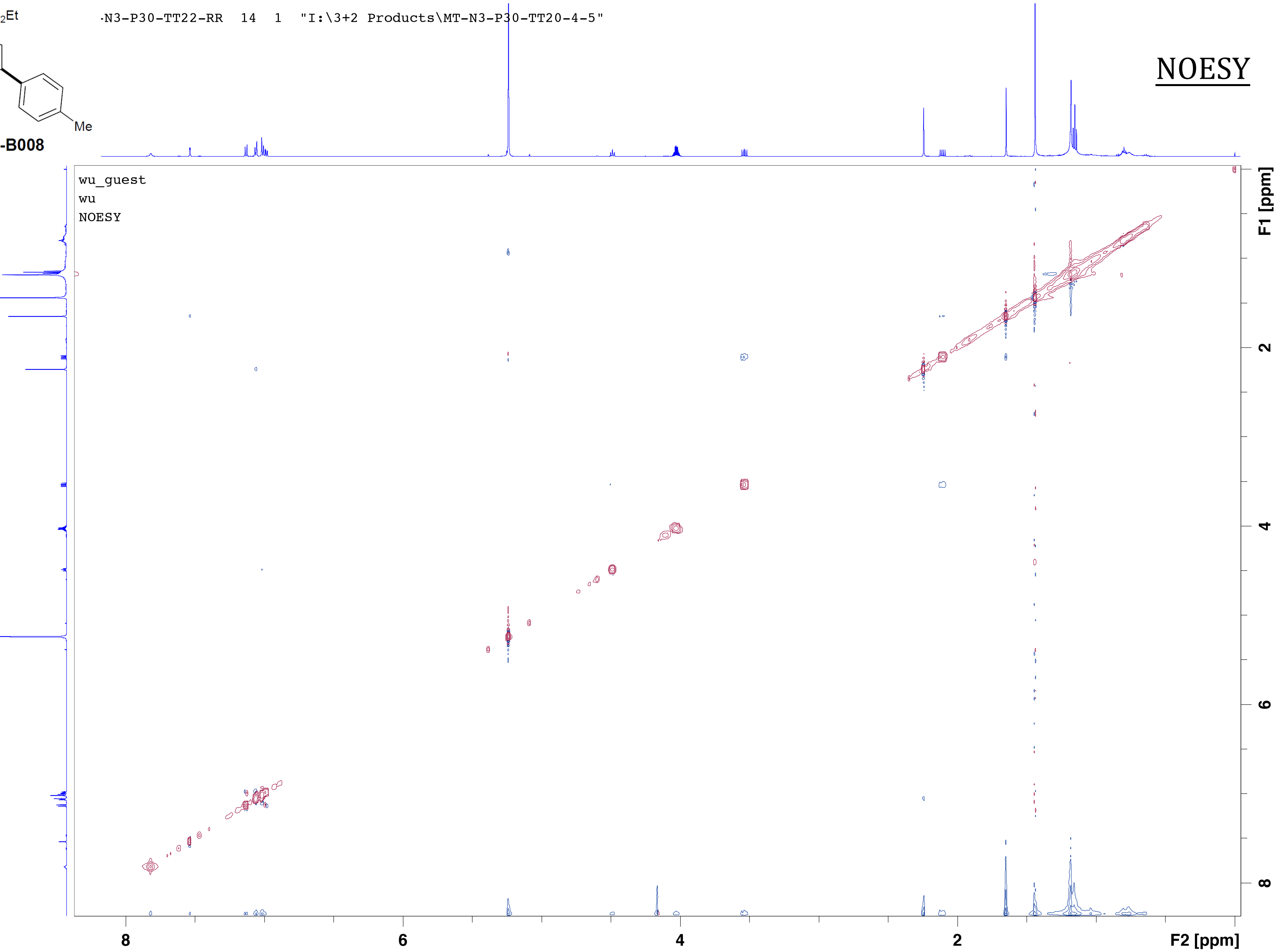

Mt-N3-P48-TT21-5-6 10 1 N:\b600\wu\data\wu_guest\nmr

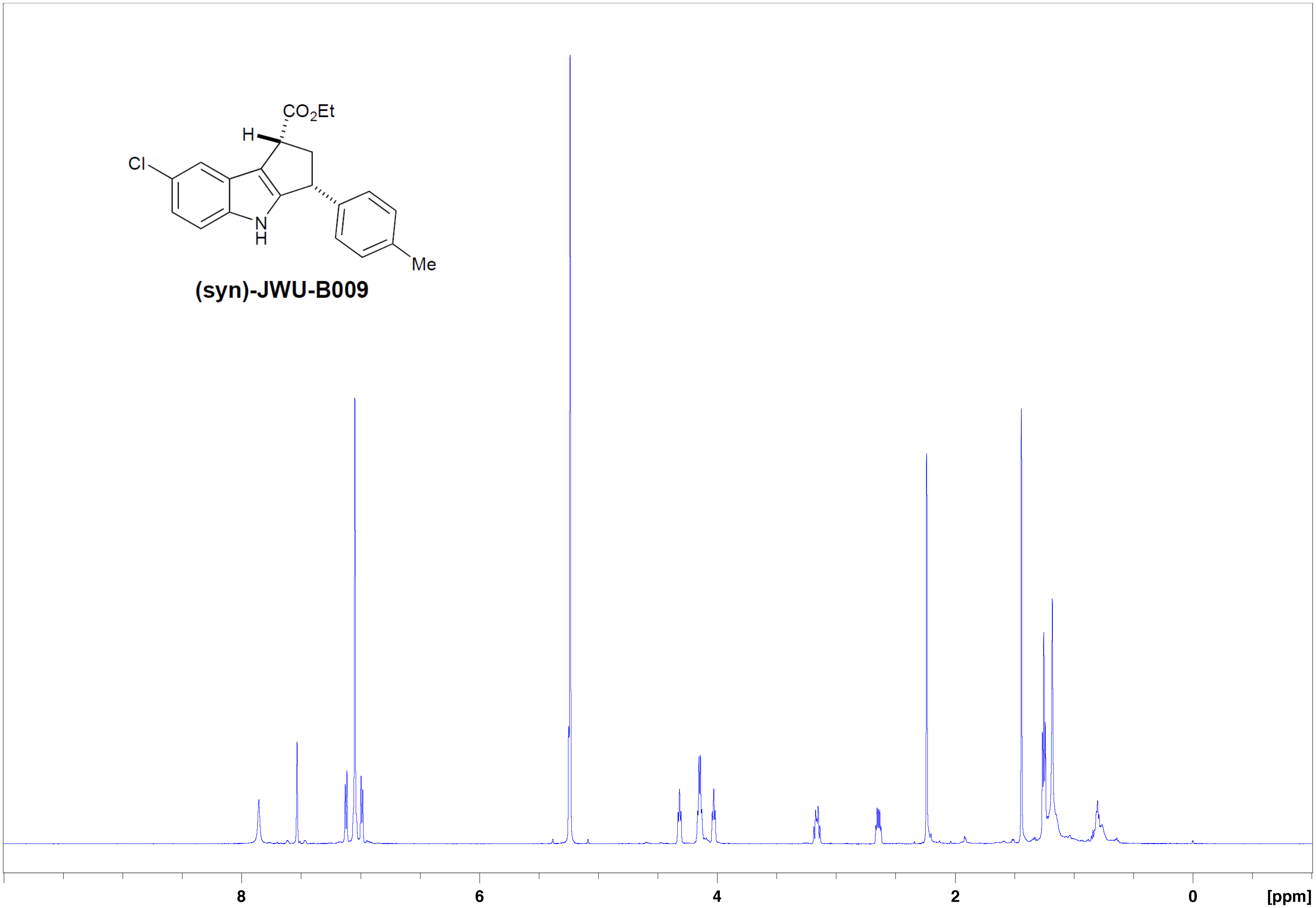

MT-N3-P48-TT21-5-6   20   1   N:\b600\wu\data\wu_guest\nmr

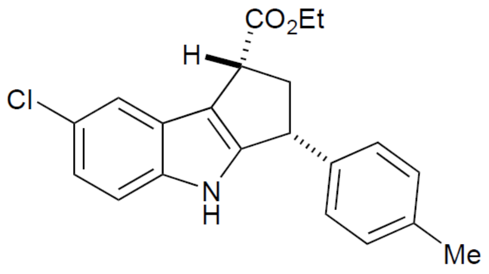

(syn)-JWU-B009

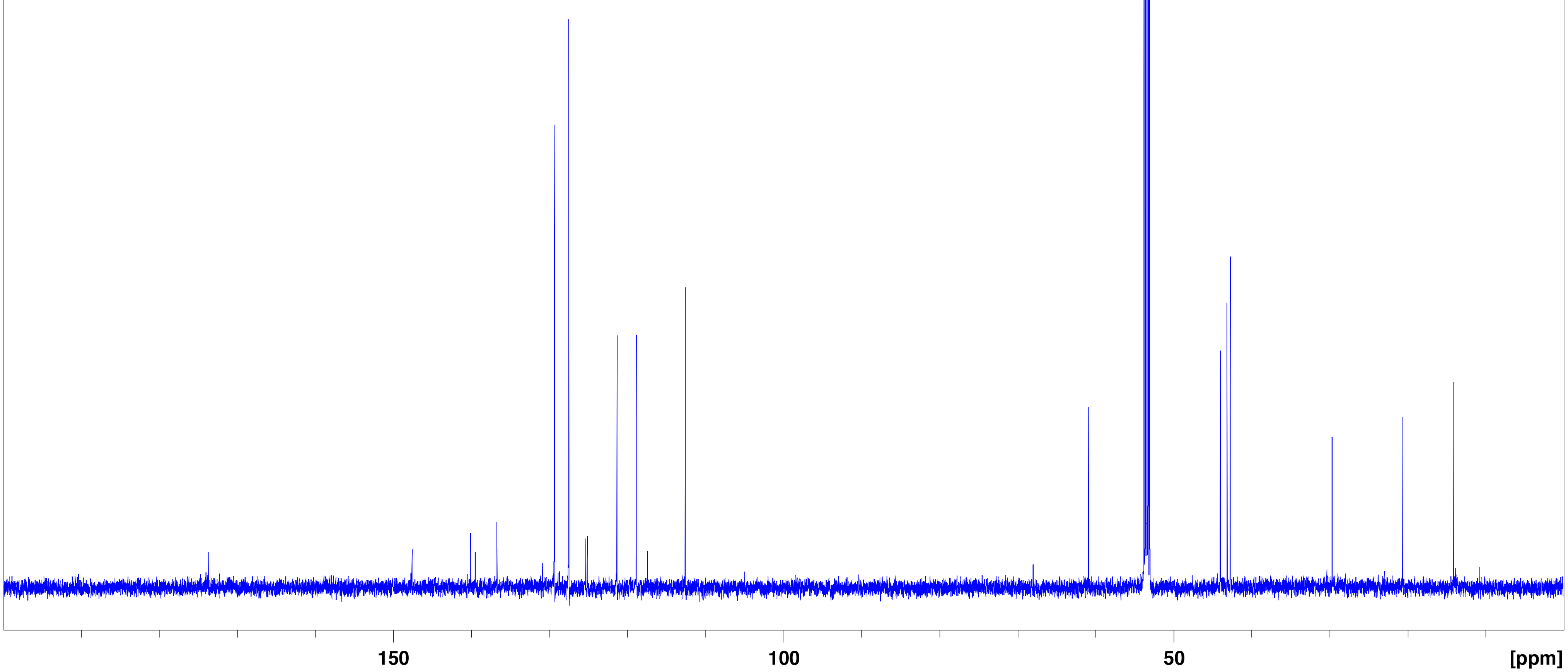

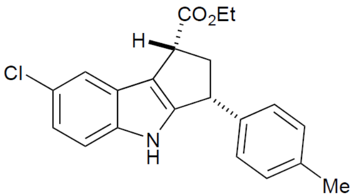
CO2Et
H
Cl
N
H
Me

(syn)-JWU-B009

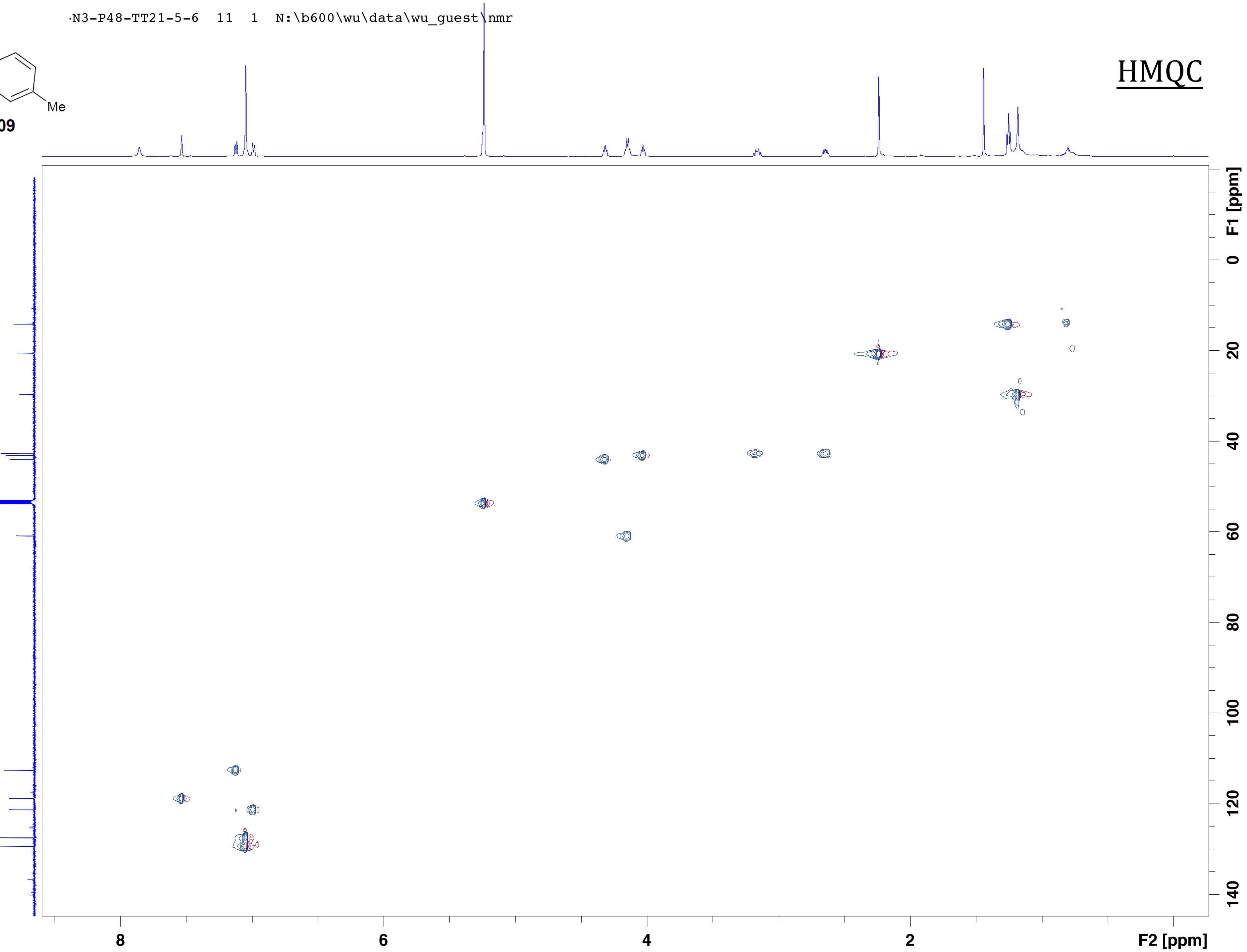
·N3-P48-TT21-5-6 11 1 N:\b600\wu\data\wu_guest\nmr
HMQC
F1 [ppm]
0
20
40
60
80
100
120
140
8
6
4
2
F2 [ppm]

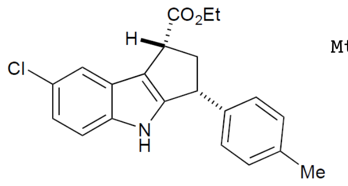
CO2Et
H
Cl
N
H
Me

(syn)-JWU-B009

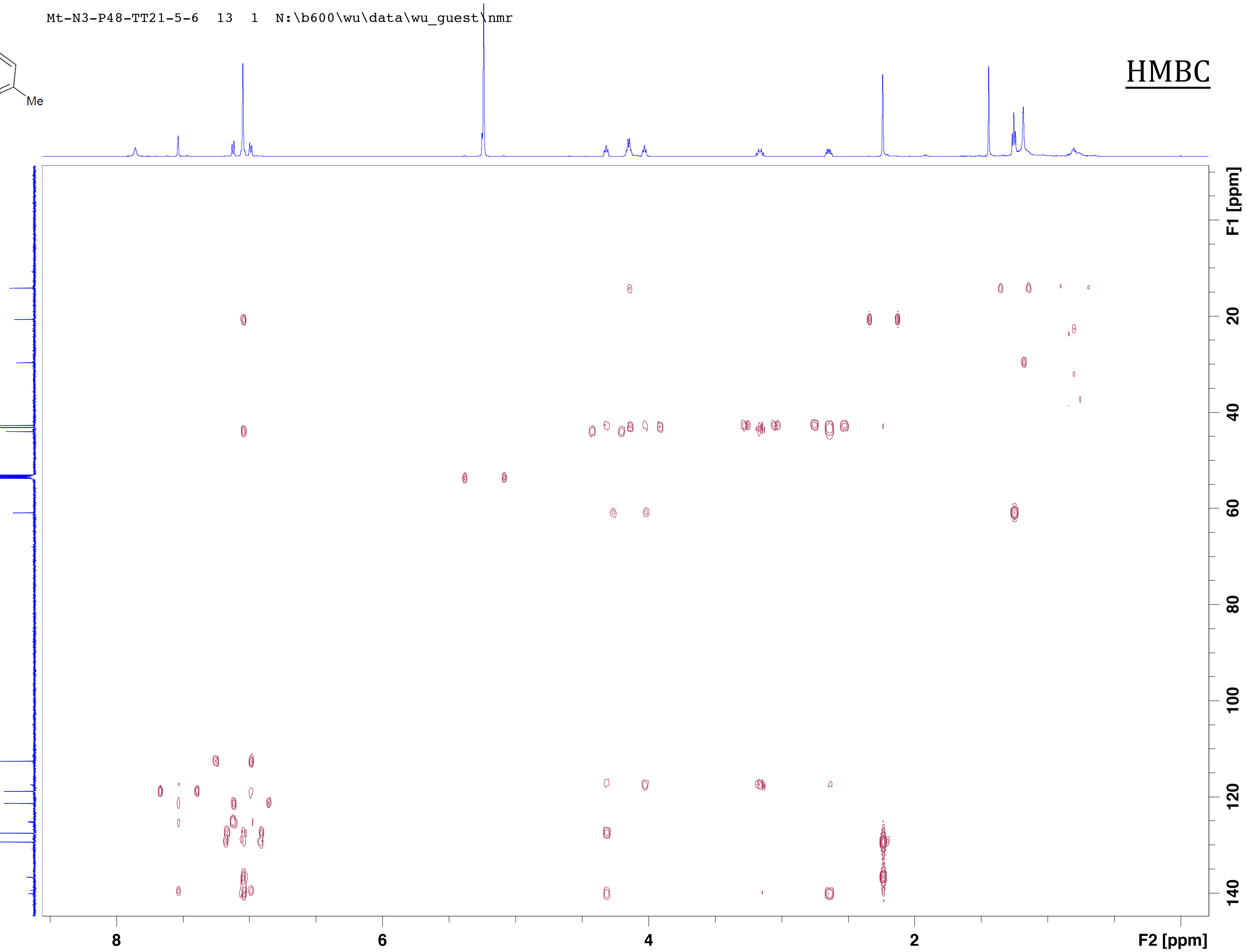
Mt-N3-P48-TT21-5-6 13 1 N:\b600\wu\data\wu_guest\nmr
HMBC
F1 [ppm]
F2 [ppm]
20
40
60
80
100
120
140
8
6
4
2

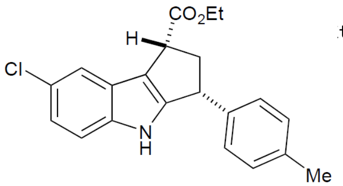

(syn)-JWU-B009

COSY

t-N3-P48-TT21-5-6 15 1 N:\b600\wu\data\wu_guest\nmr

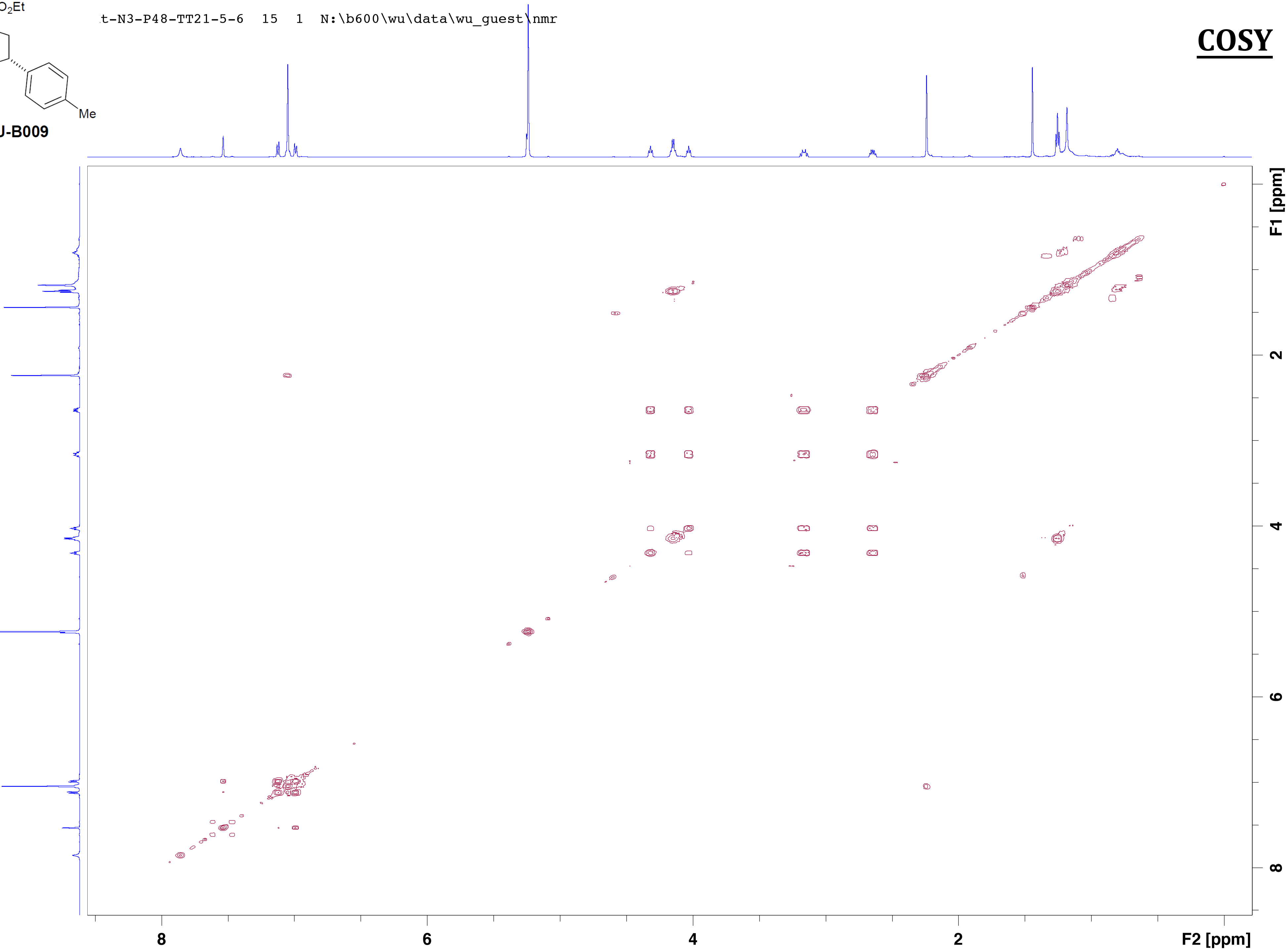

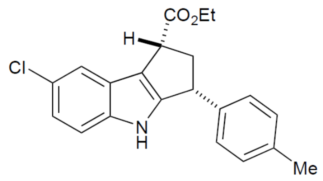

(syn)-JWU-B009

NOESY

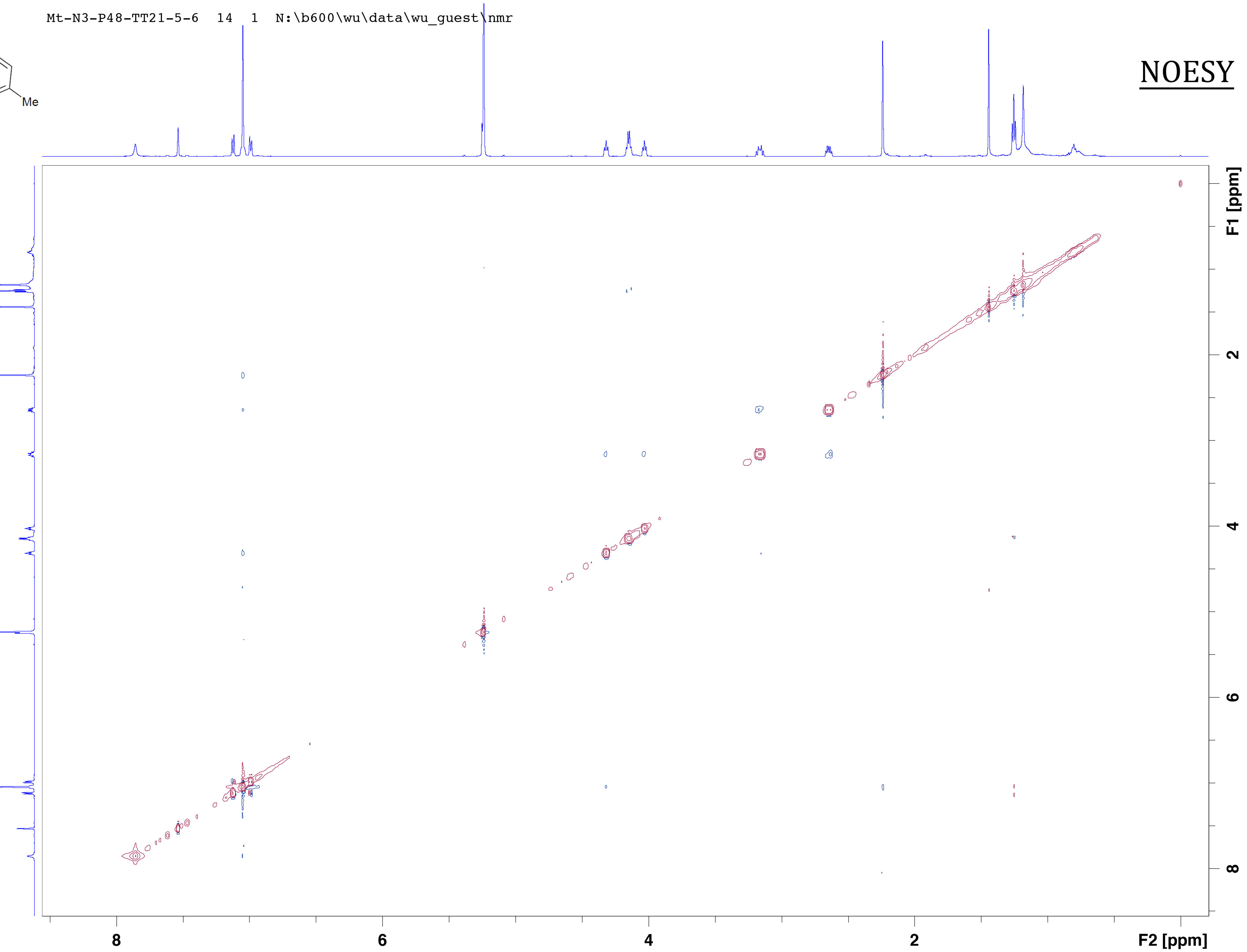

MT-N3-P48-TT23-3-4 10 1 N:\b600\wu\data\wu_guest\nmr

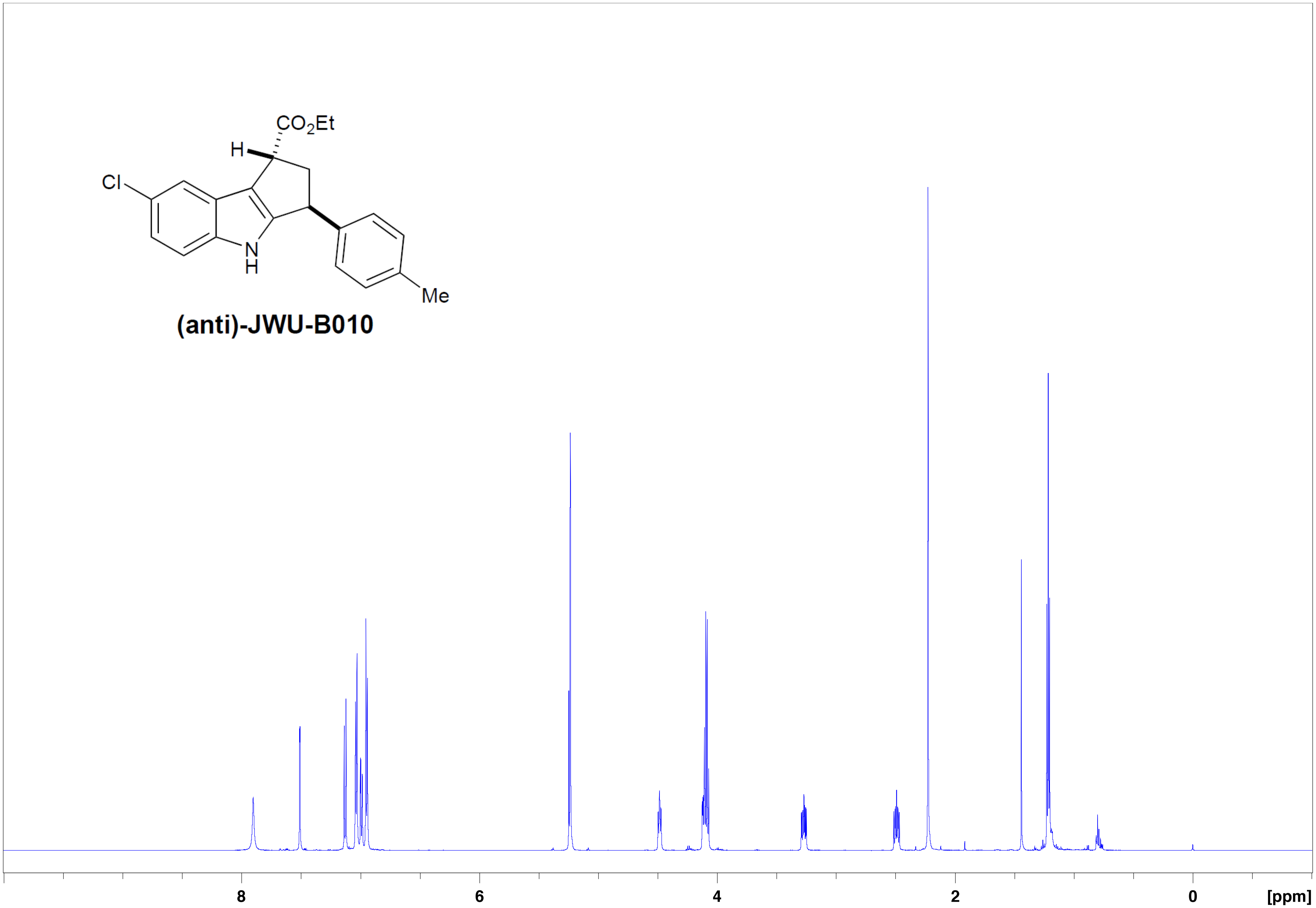

MT-N3-P48-TT23-3-4   25   1   N:\b600\wu\data\wu_guest\nmr

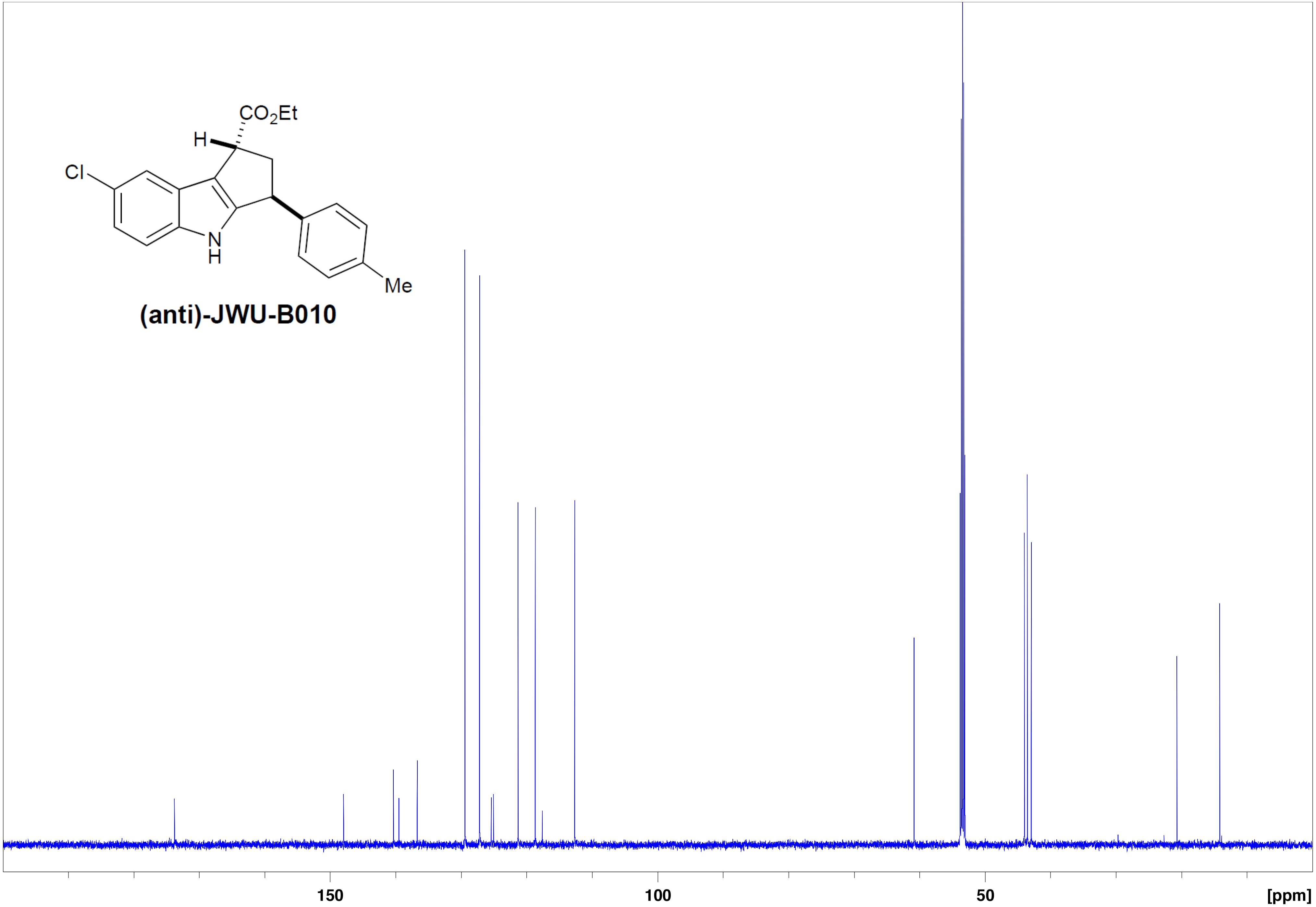

(anti)-JWU-B010

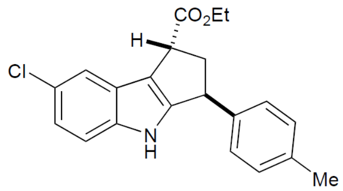

(anti)-JWU-B010

HMQC

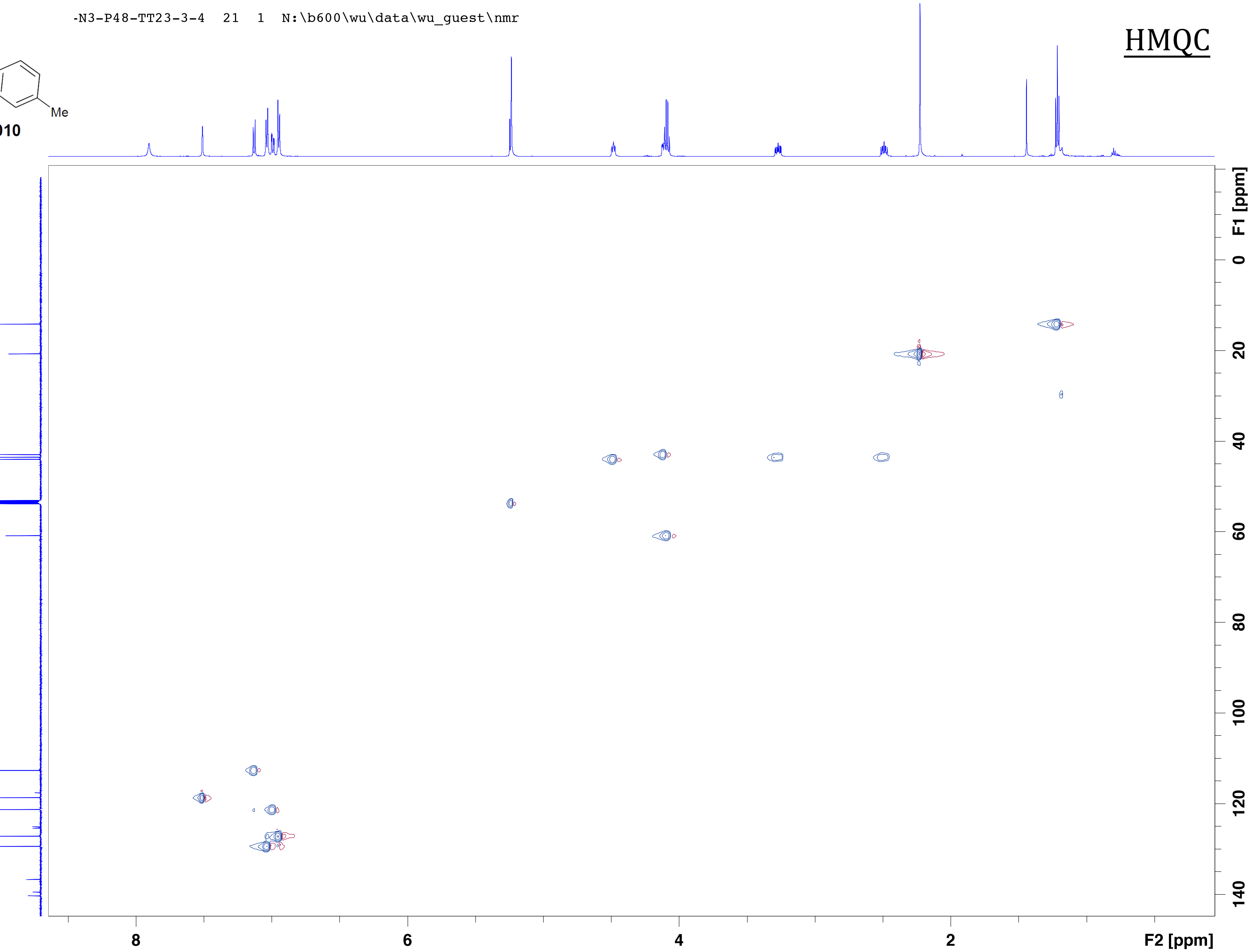

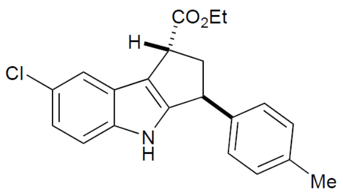

(anti)-JWU-B010

HMBC

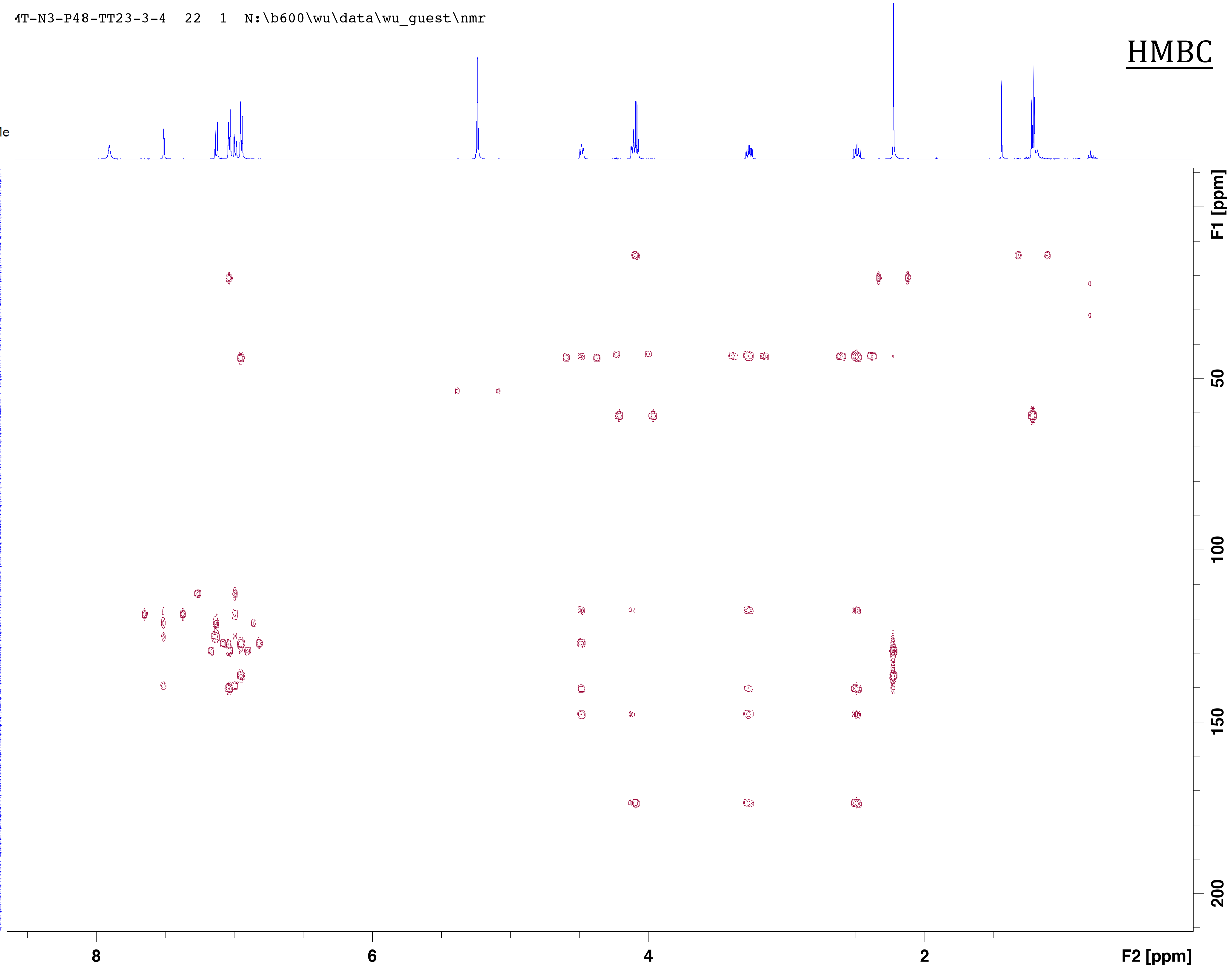

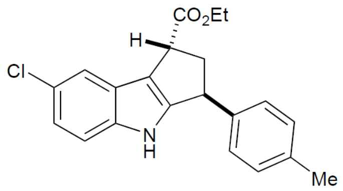

(anti)-JWU-B010

MT-N3-P48-TT23-3-4   24   1   N:\b600\wu\data\wu_guest\nmr

COSY

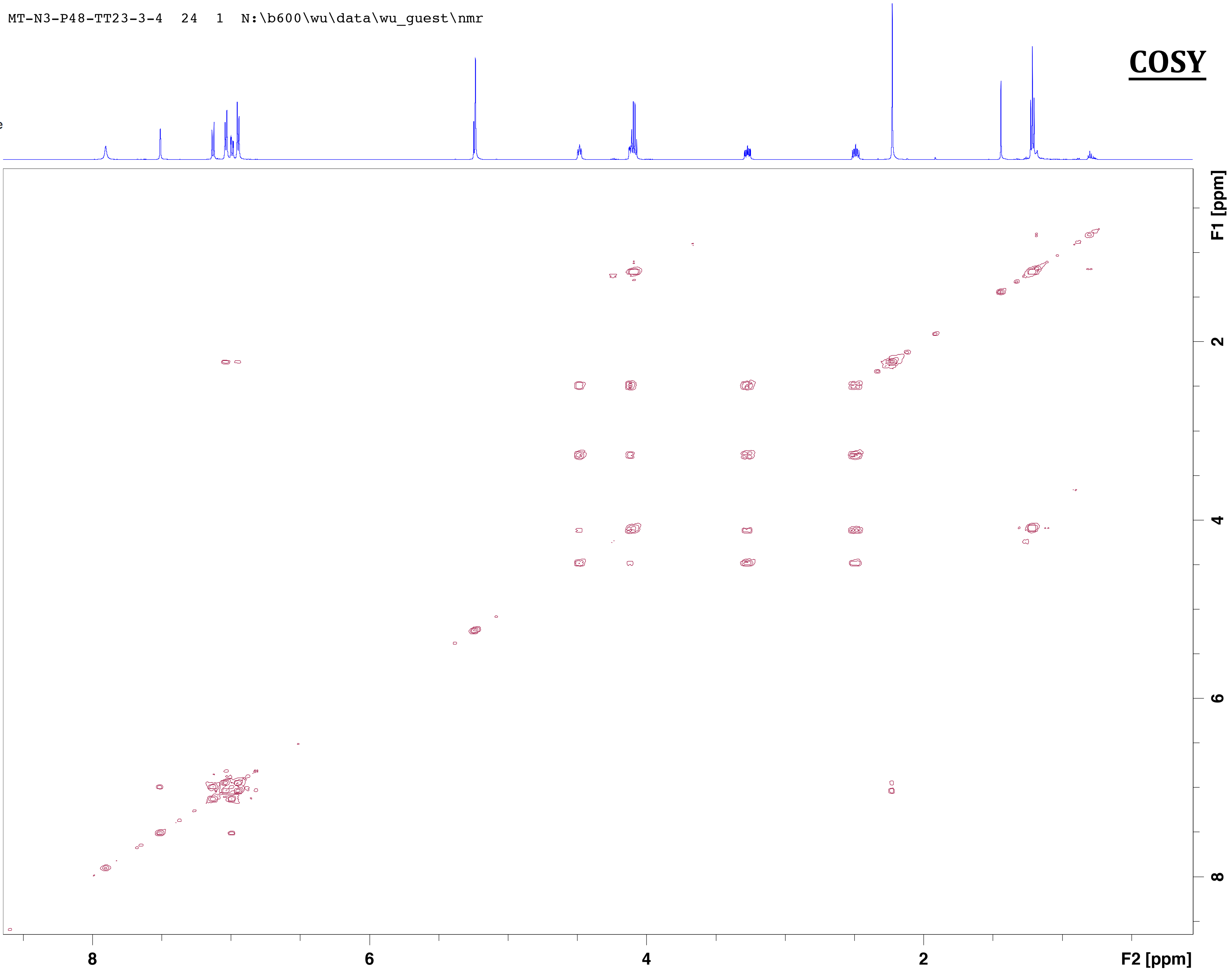

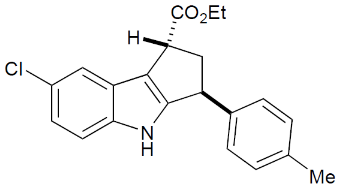

(anti)-JWU-B010

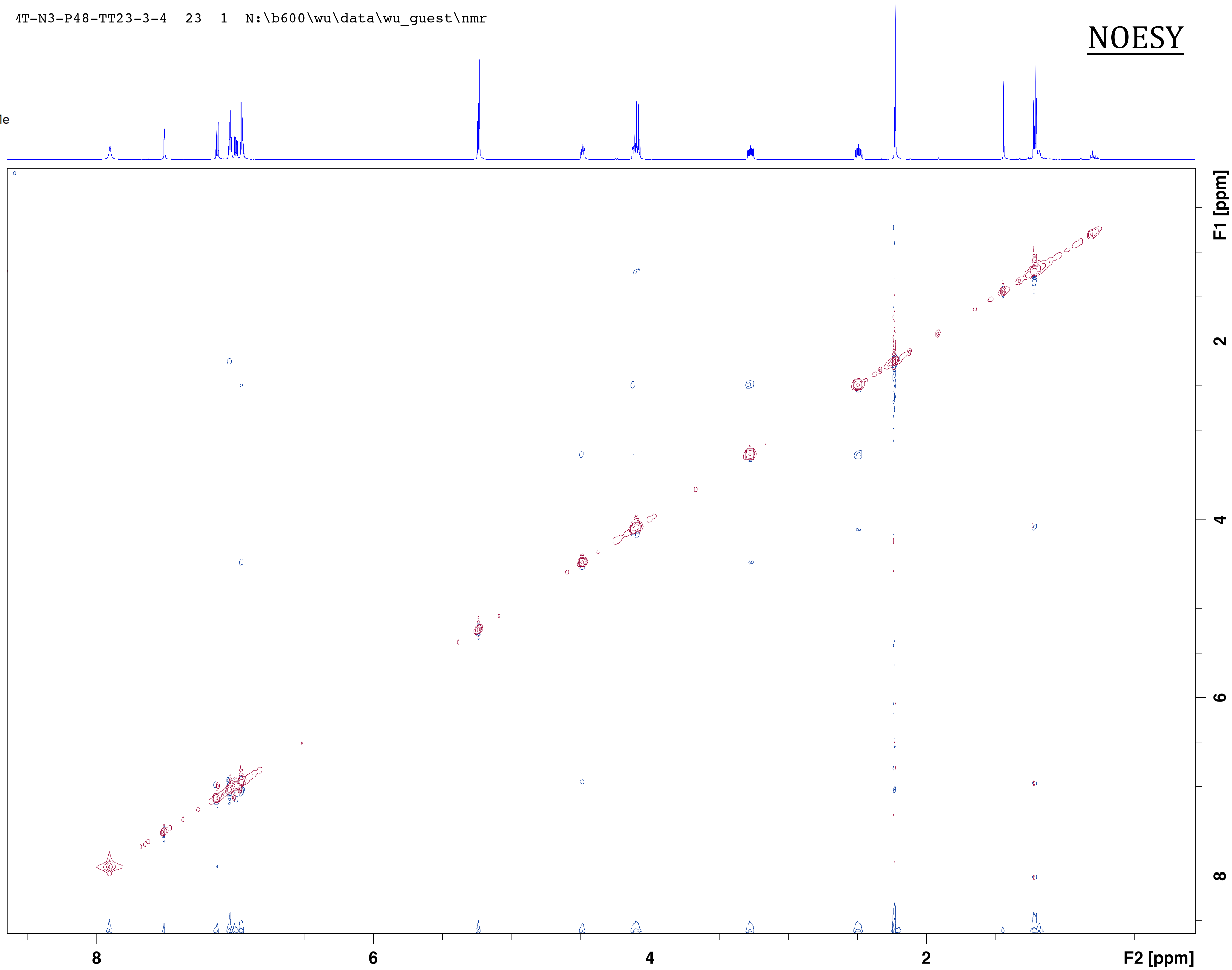

MT-N3-P86-46-48-6-7 10 1 "I:\3+2 Products"

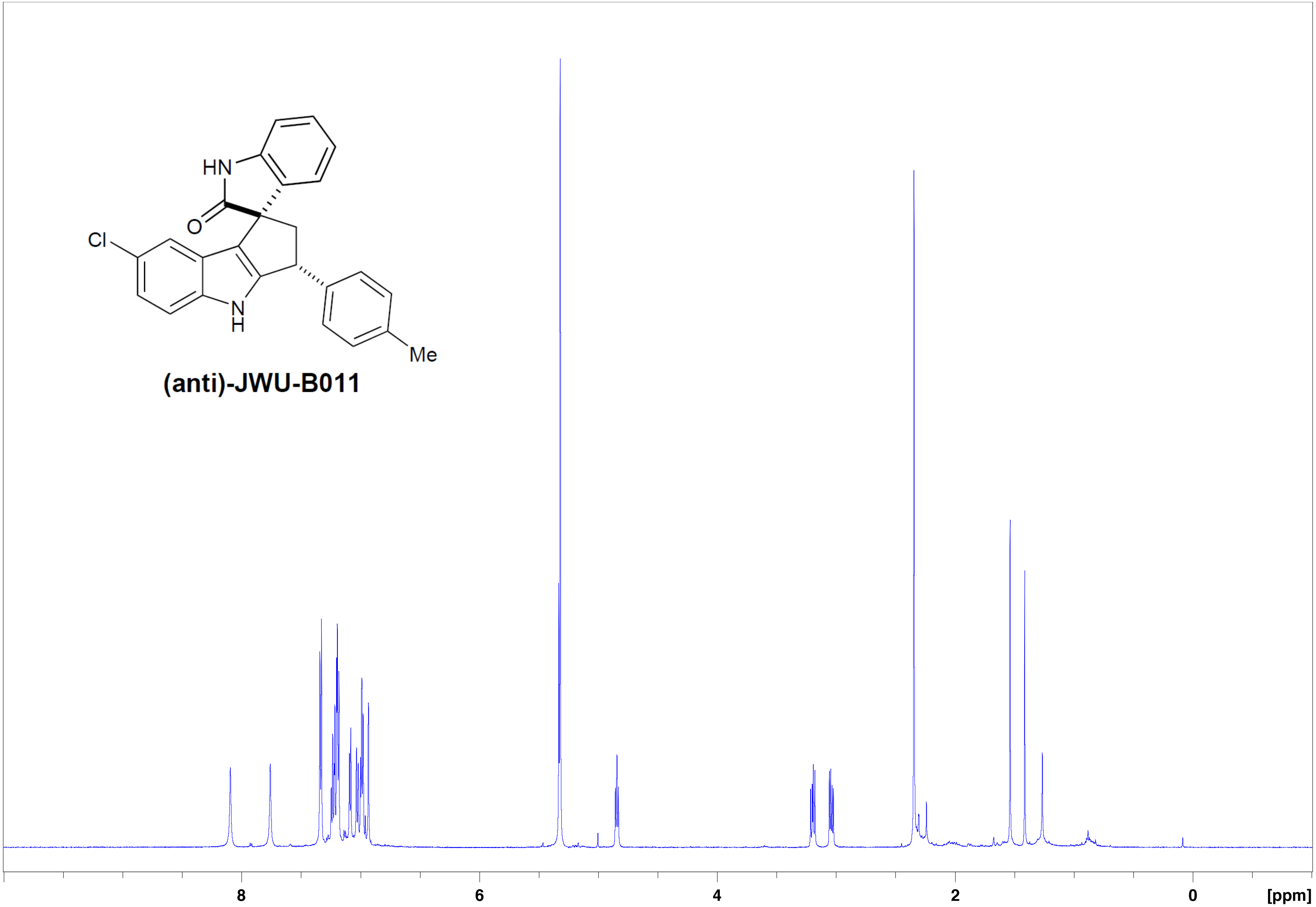

MT-N3-P86-46-48-6-7 20 1 "I:\3+2 Products"

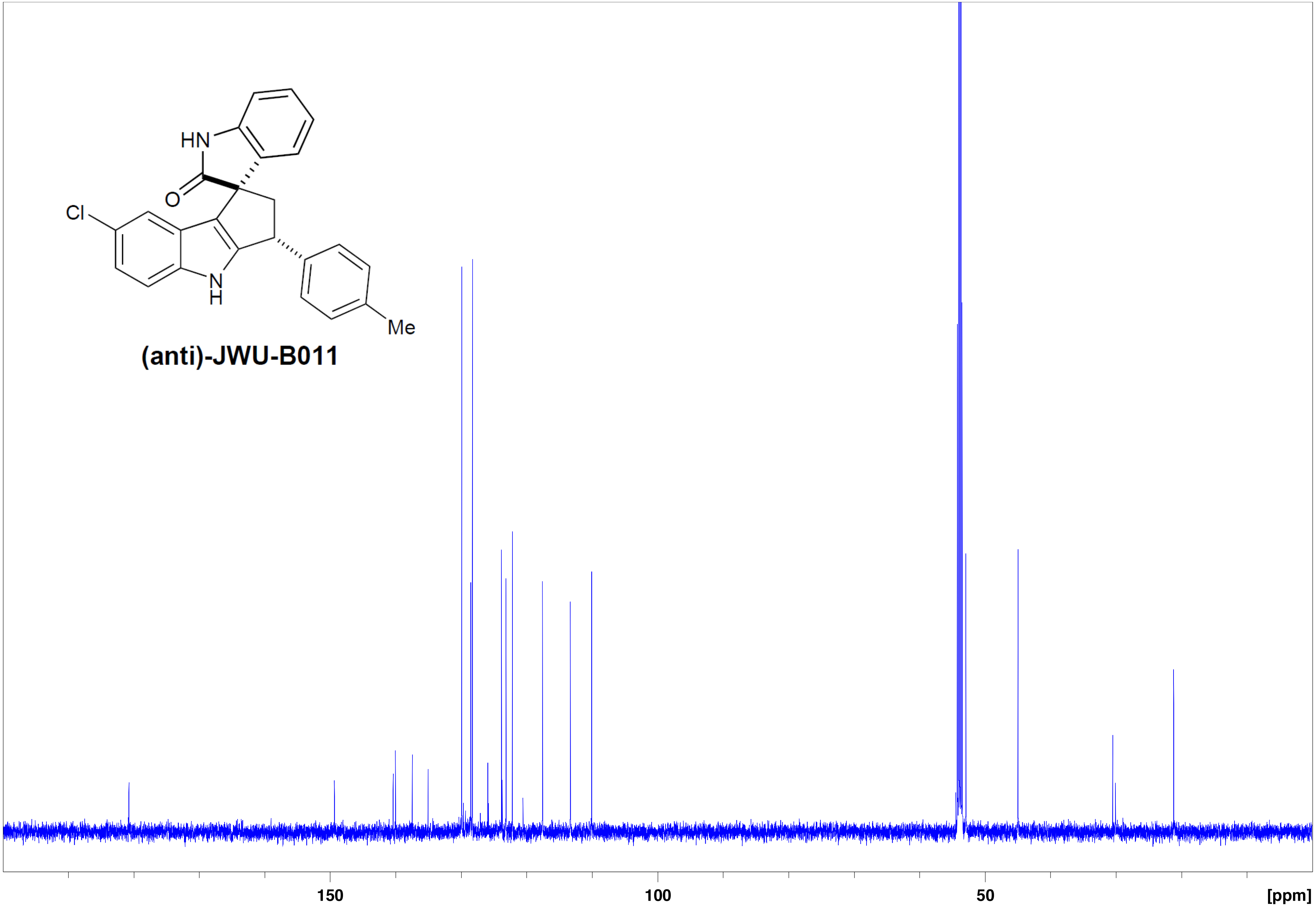

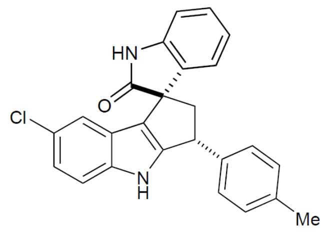

(anti)-JWU-B011

HMQC

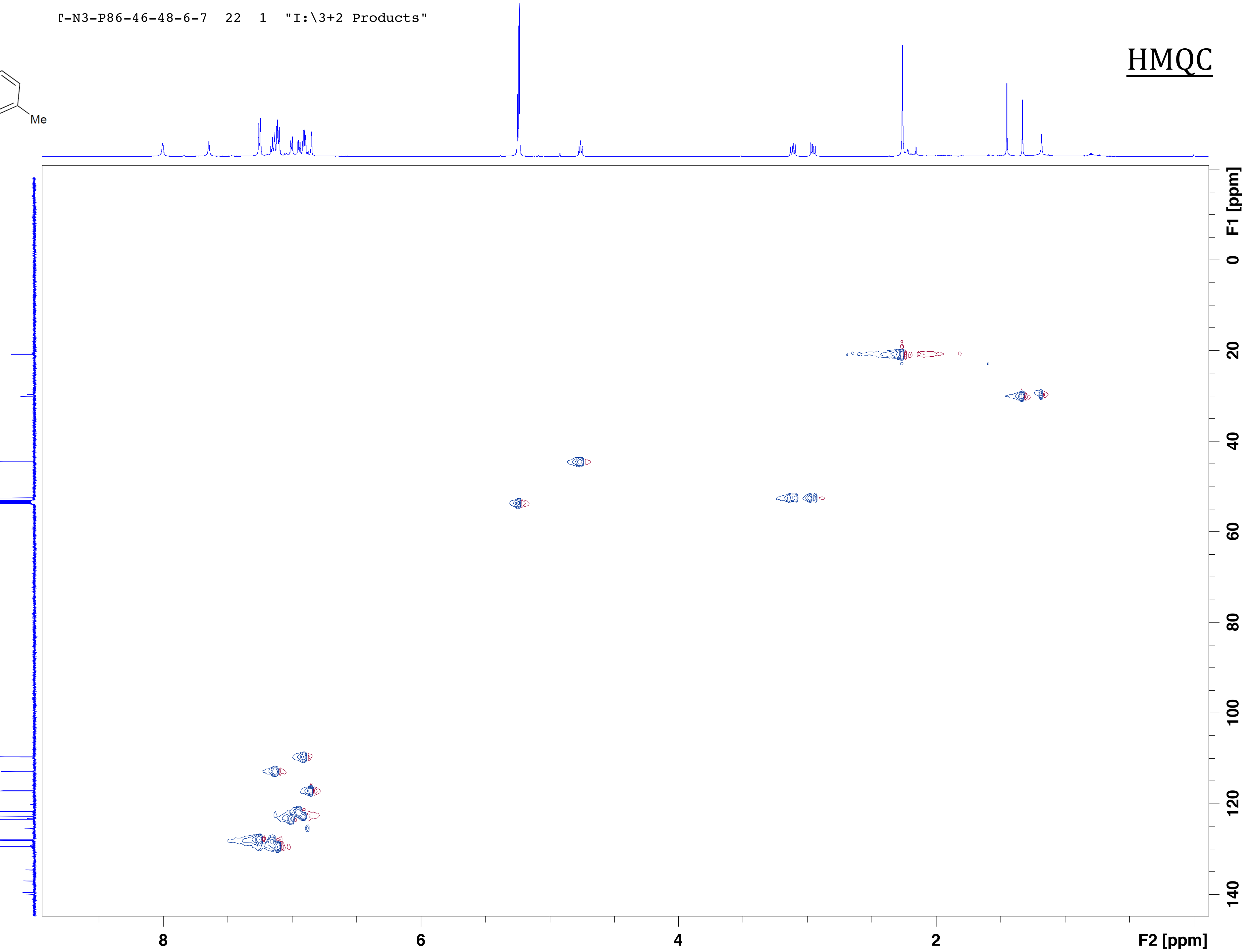

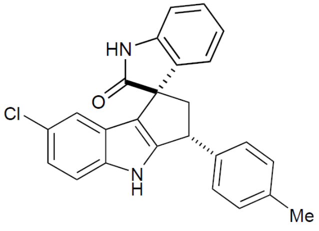

(anti)-JWU-B011

HMBC

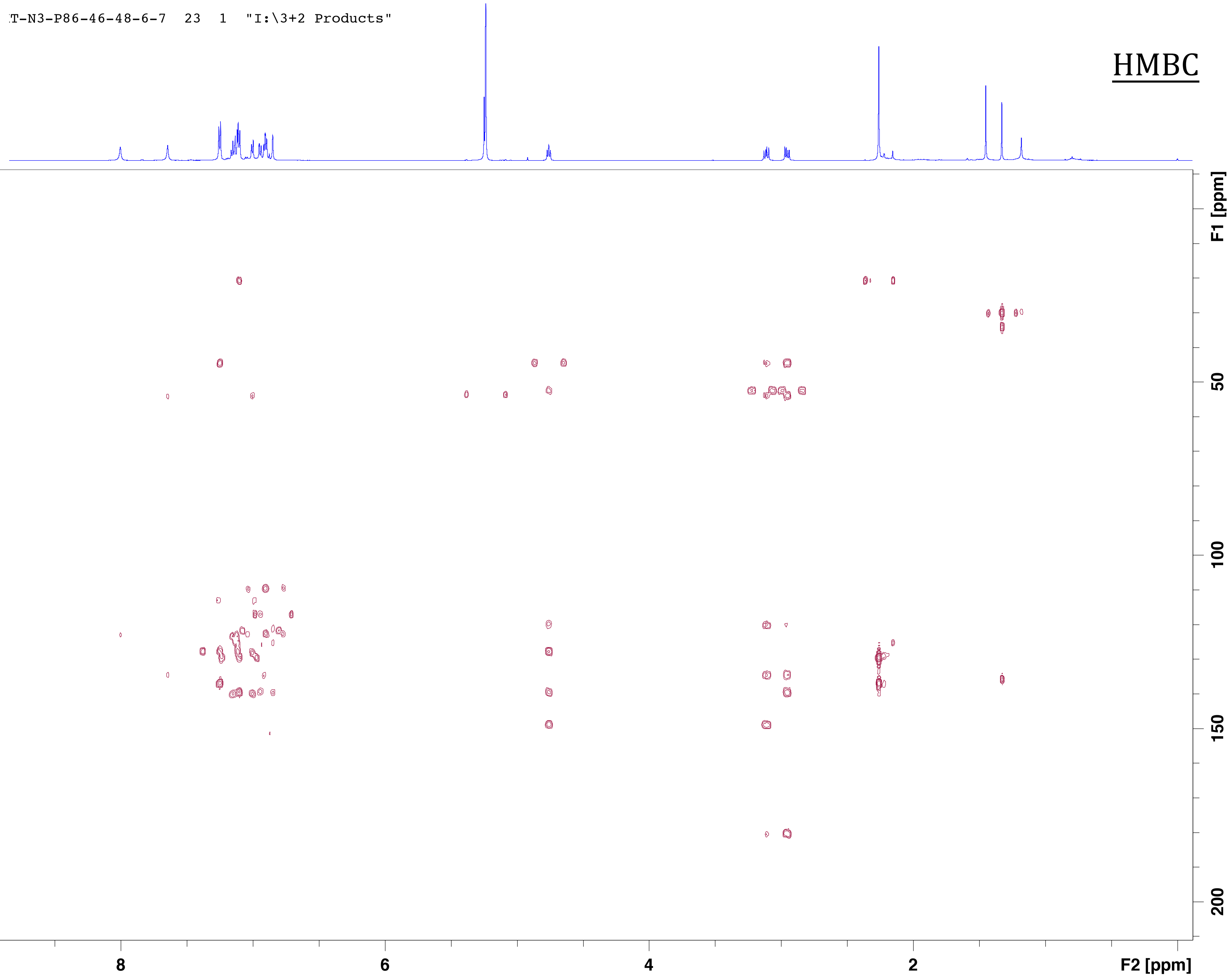

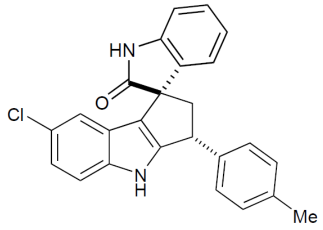

(anti)-JWU-B011

COSY

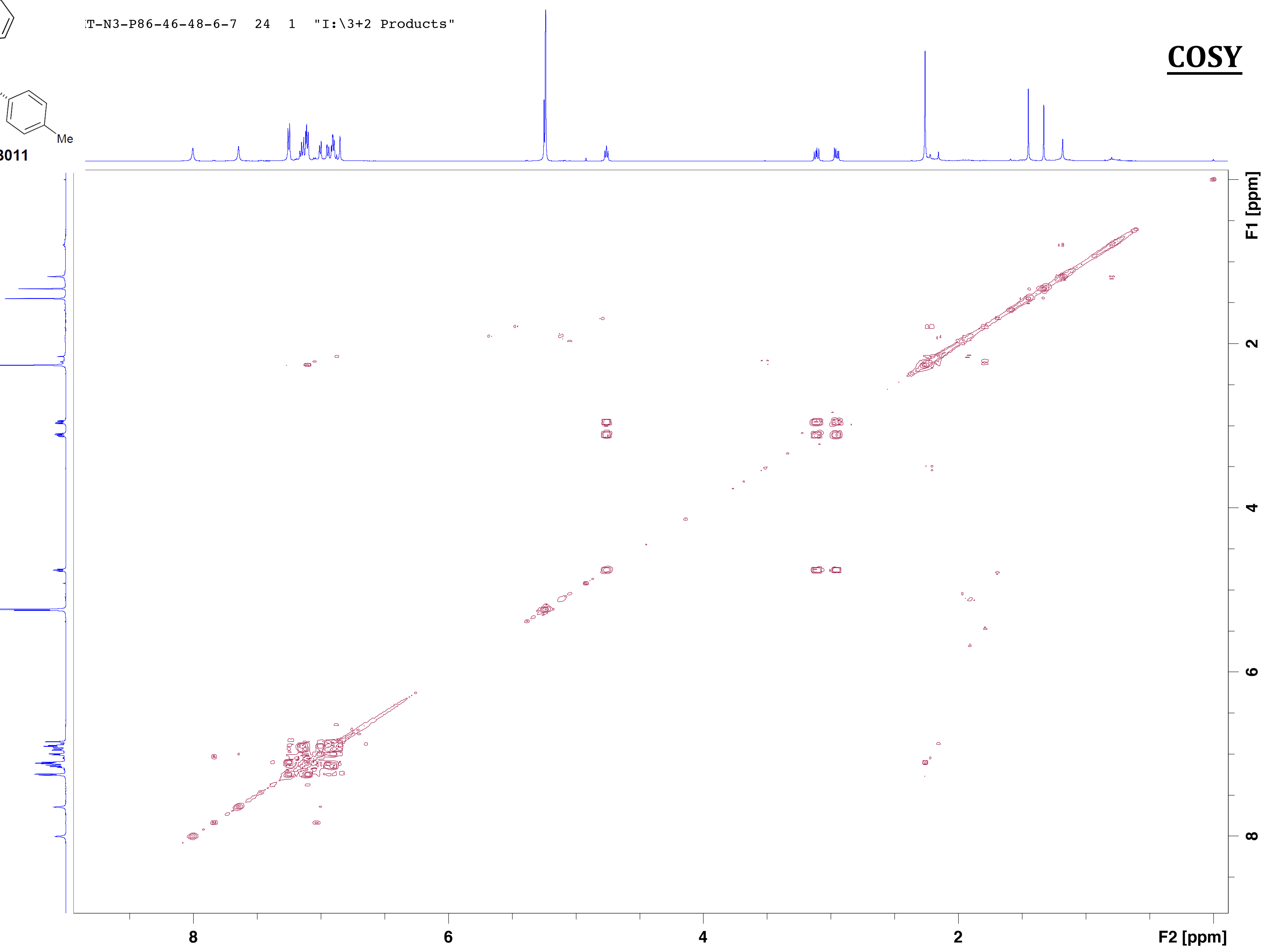

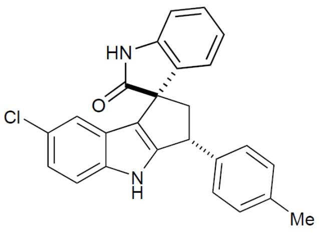

(anti)-JWU-B011

NOESY

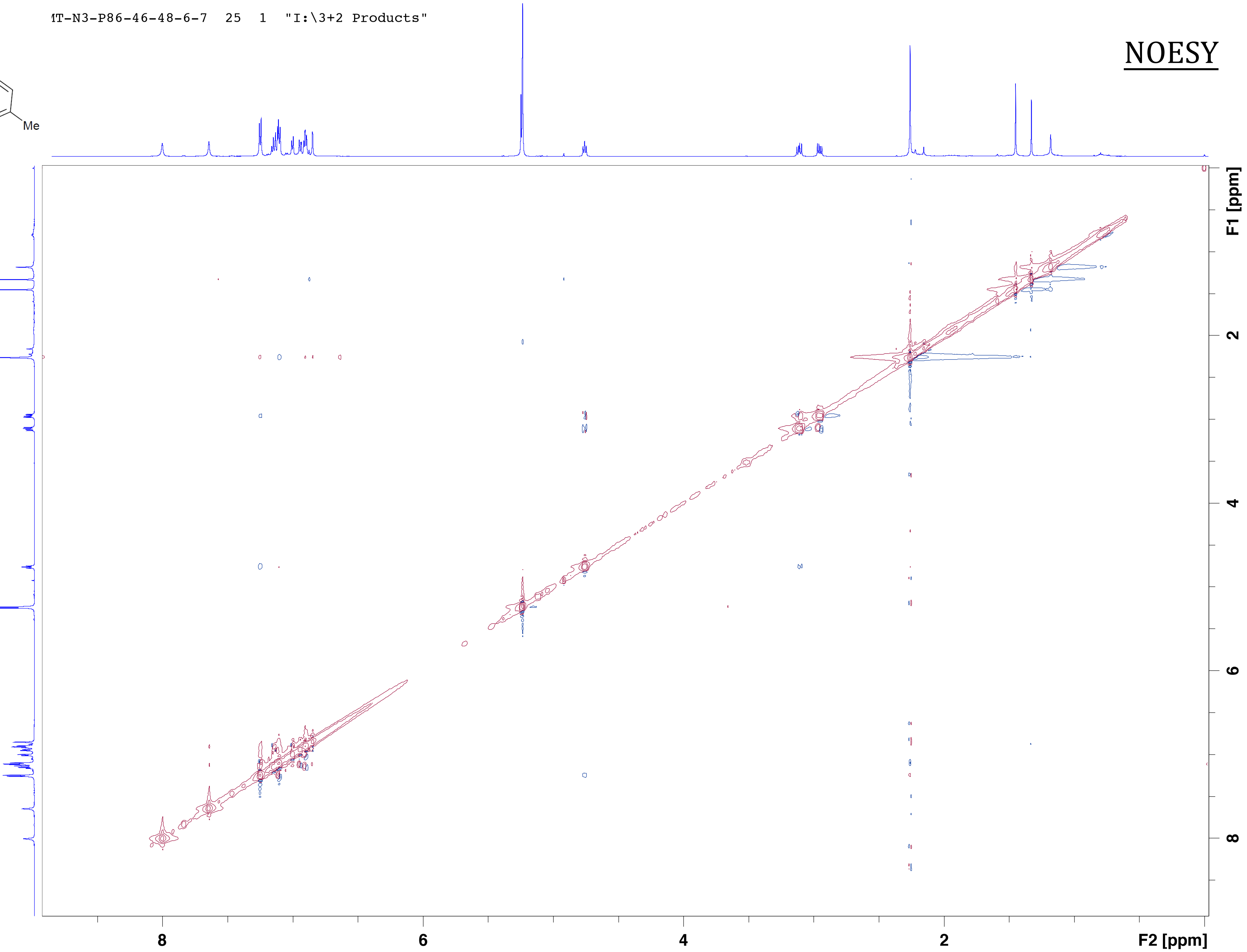

MT-N3-P86-55-59-7-9   10   1   "I:\3+2 Products"

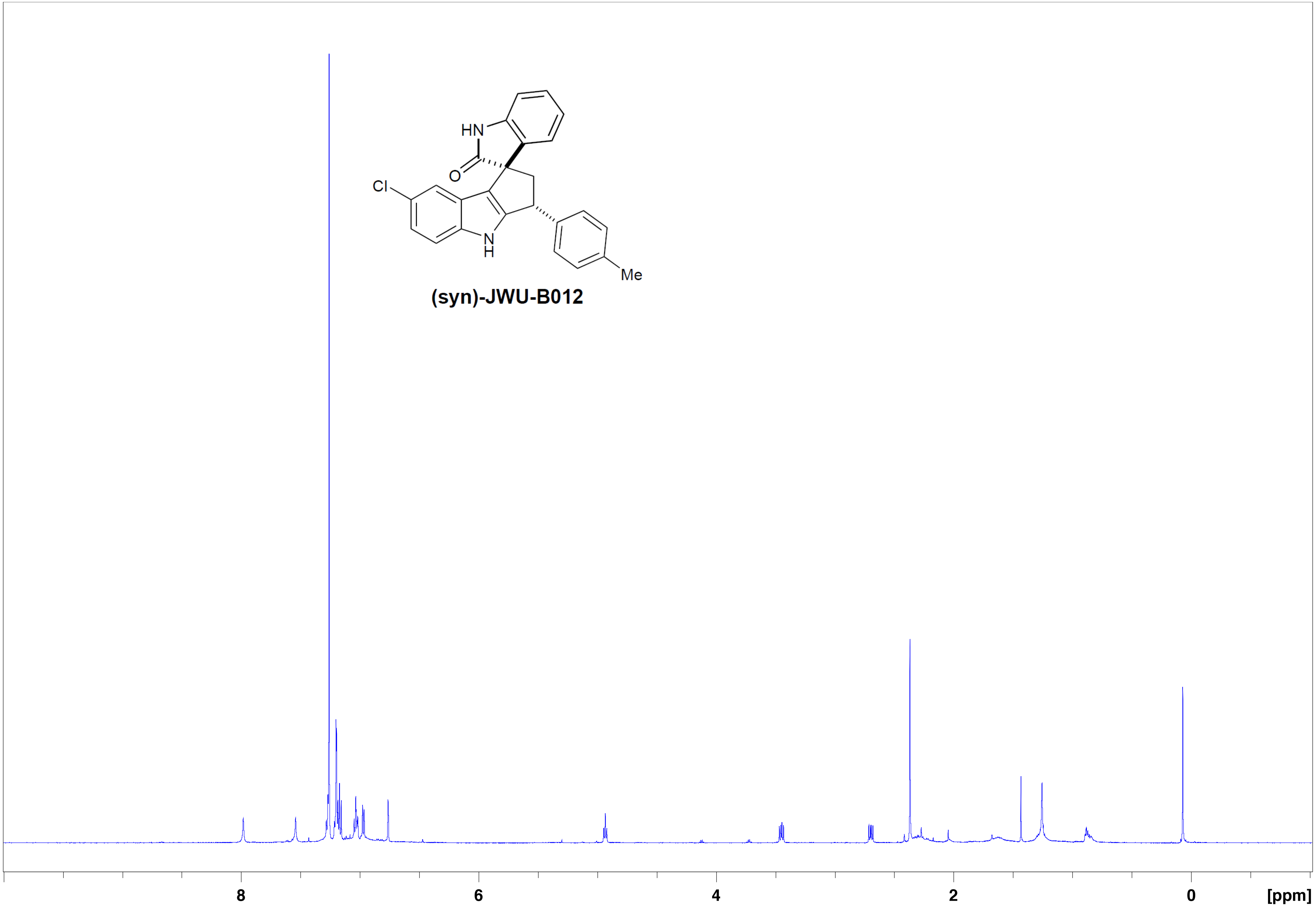

"MCD-06-02c carbon" 11 1 /Users/mariacdipoto/Desktop/NMR

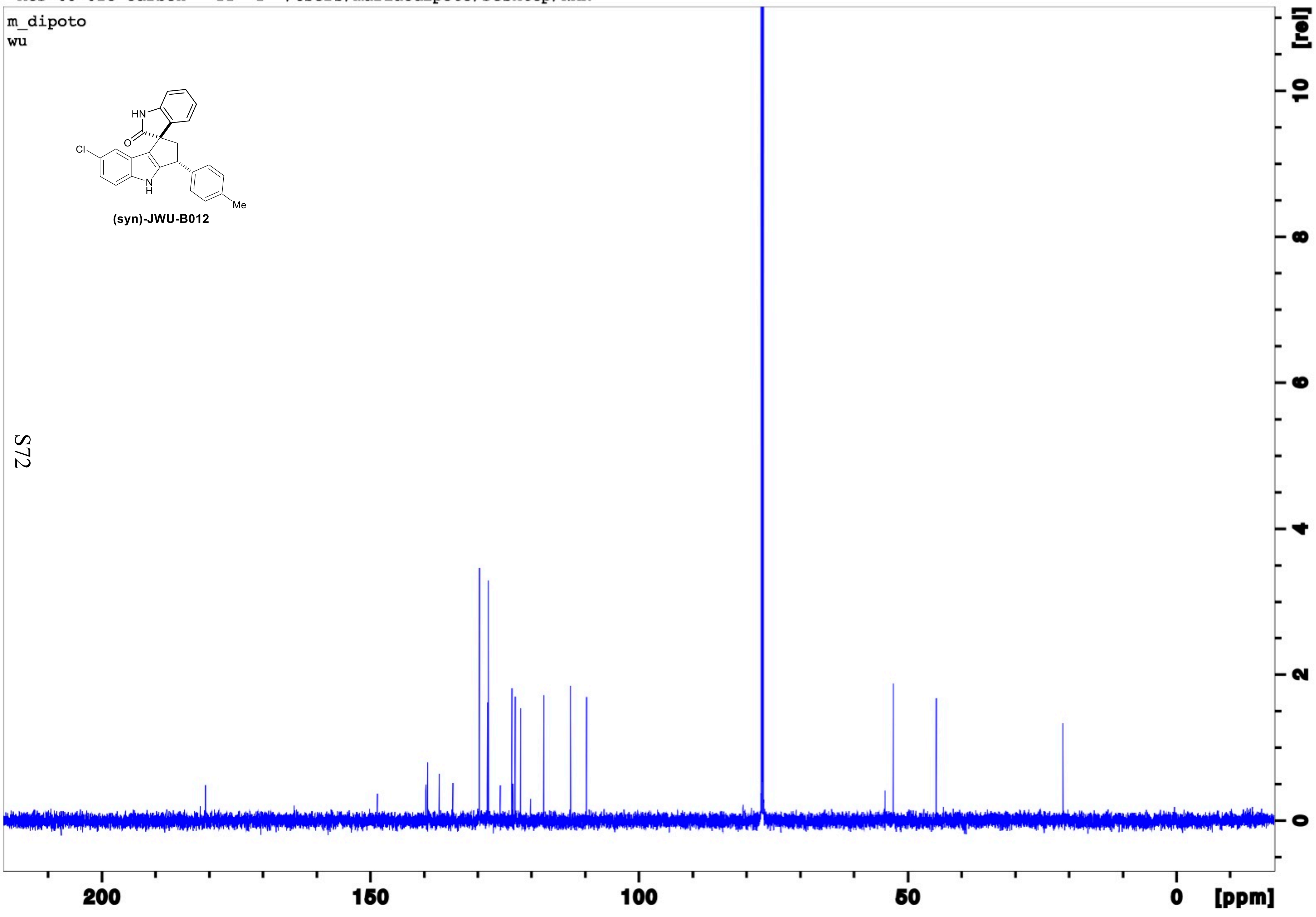

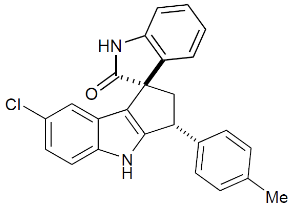

(syn)-JWU-B012

HMQC

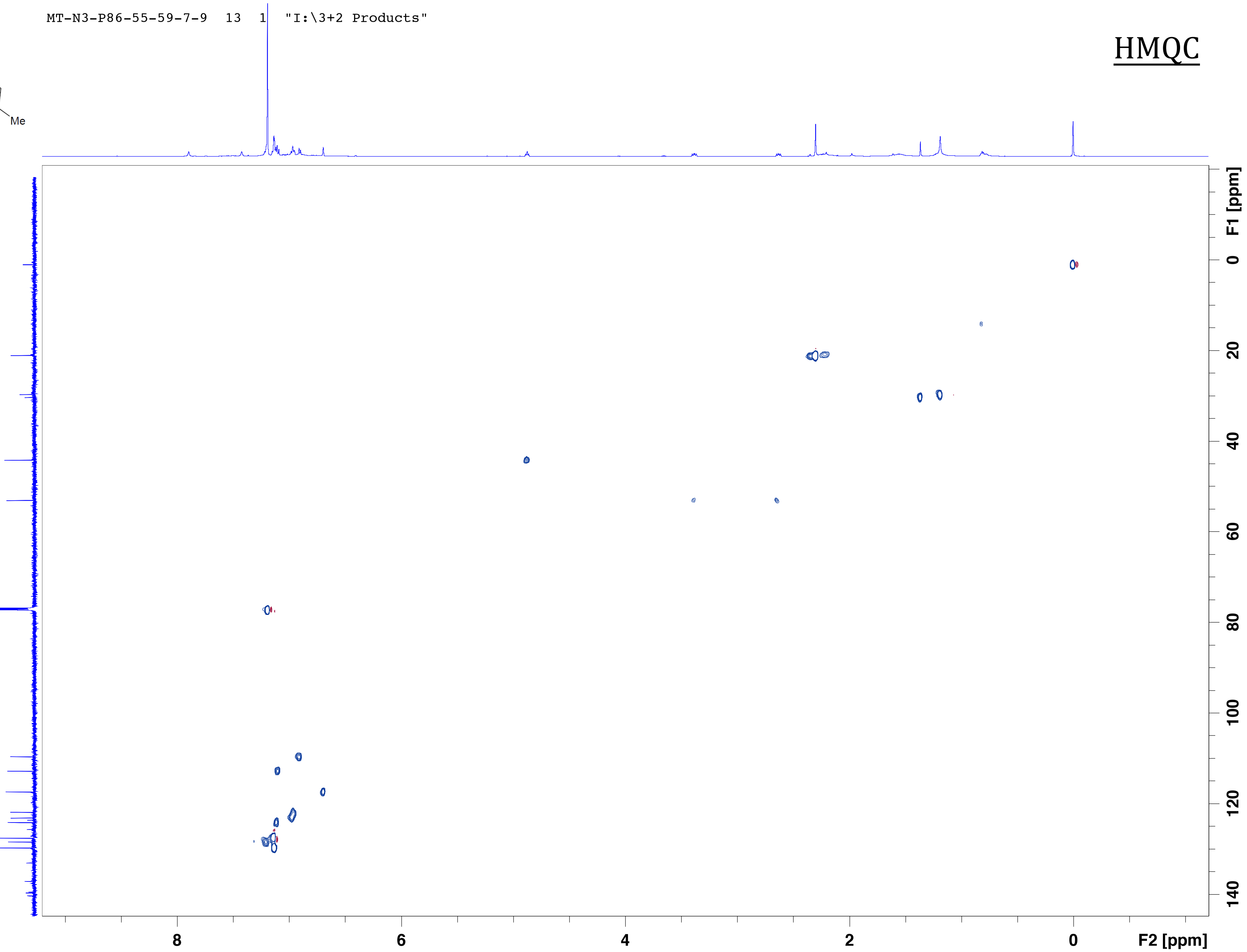

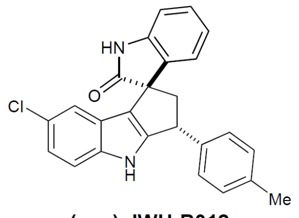
HN
O
Cl
N
H
Me

(syn)-JWU-B012

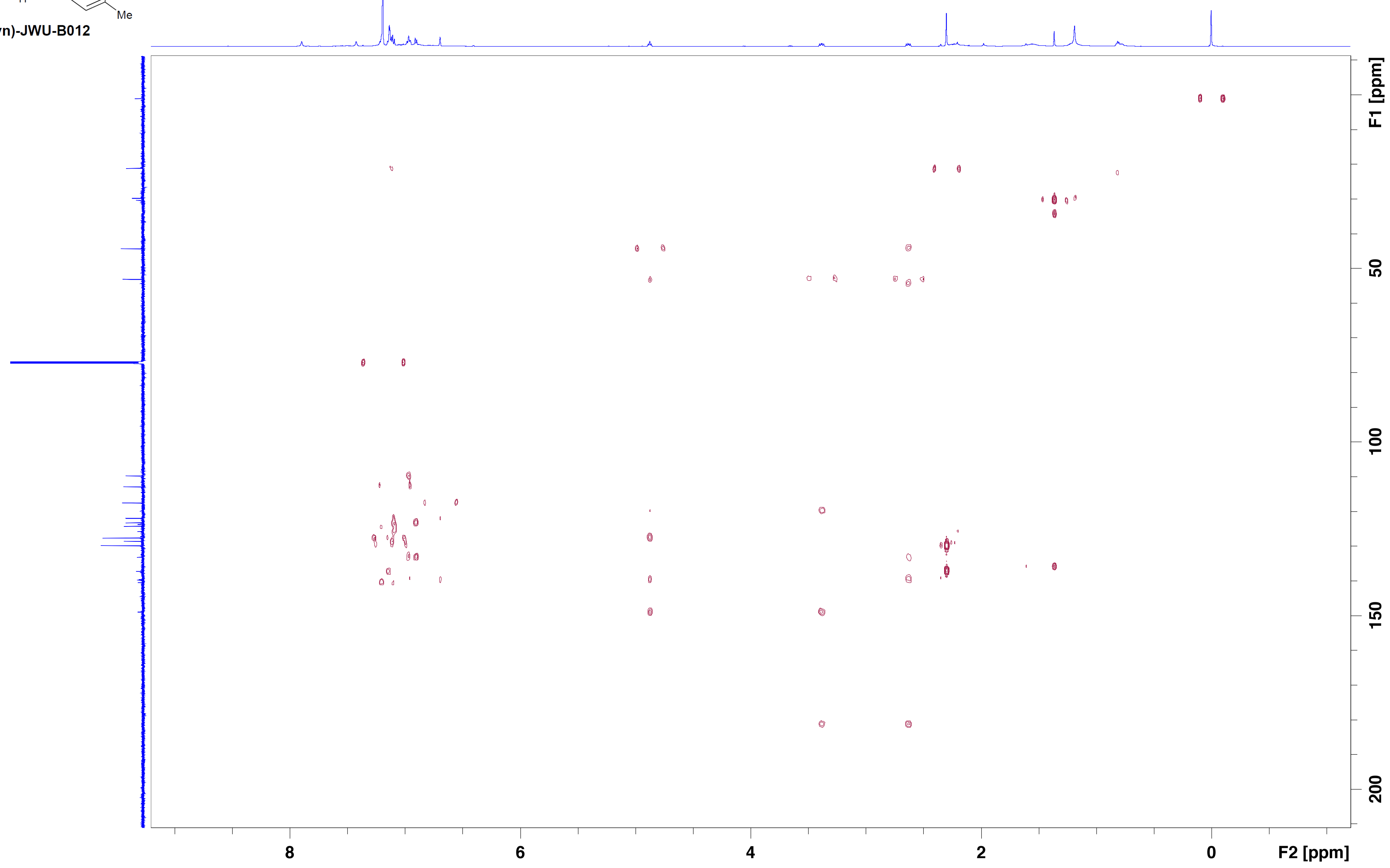
MT-N3-P86-55-59-7-9 14 1 "I:\3+2 Products"
HMBC
F1 [ppm]
50
100
150
200
8
6
4
2
0
F2 [ppm]

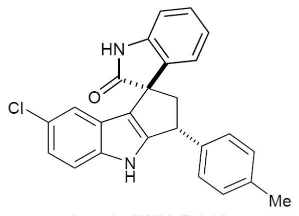

(syn)-JWU-B012

COSY

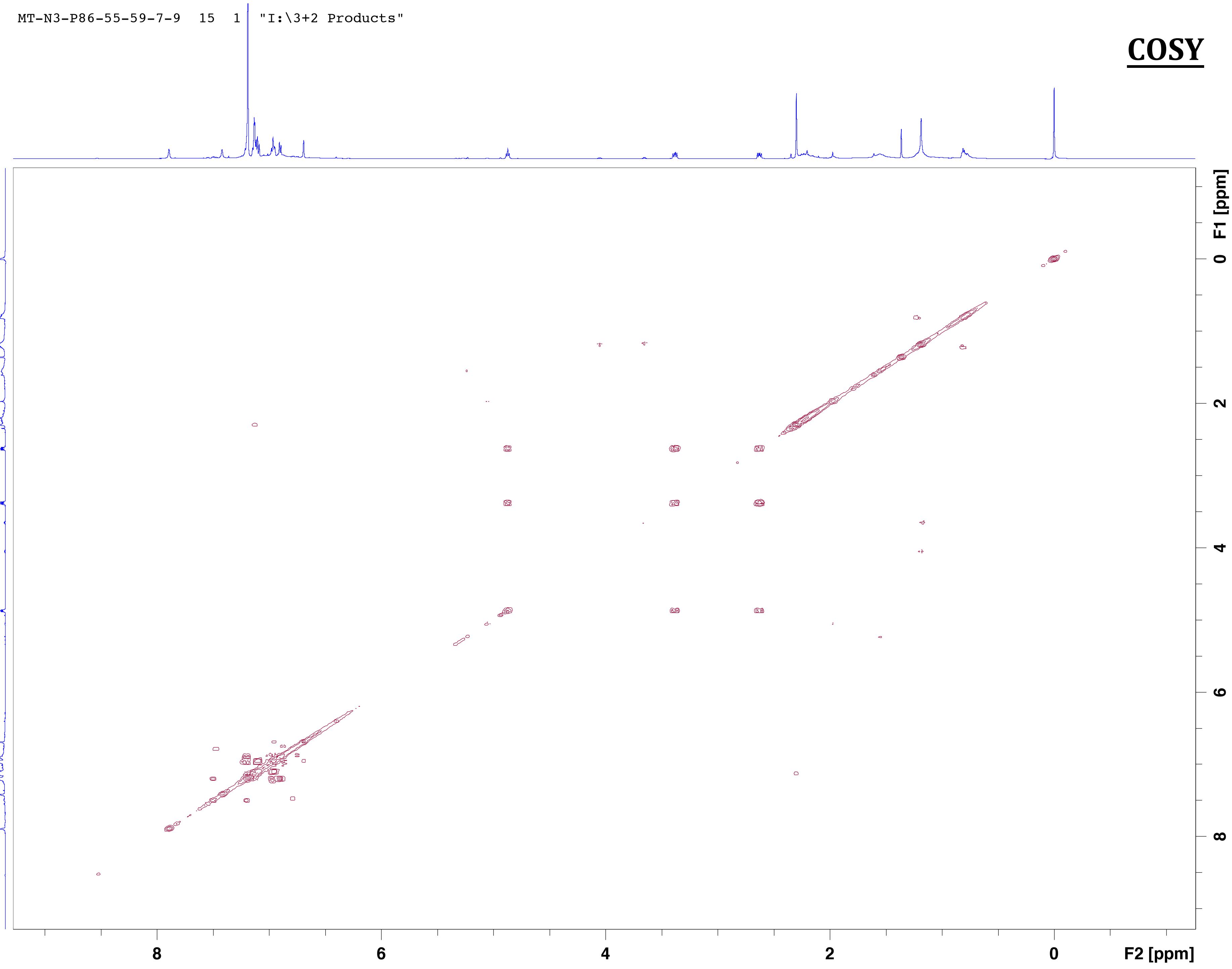

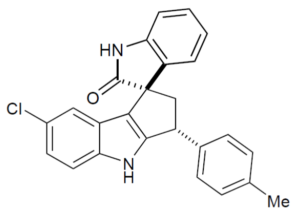

(syn)-JWU-B012

NOESY

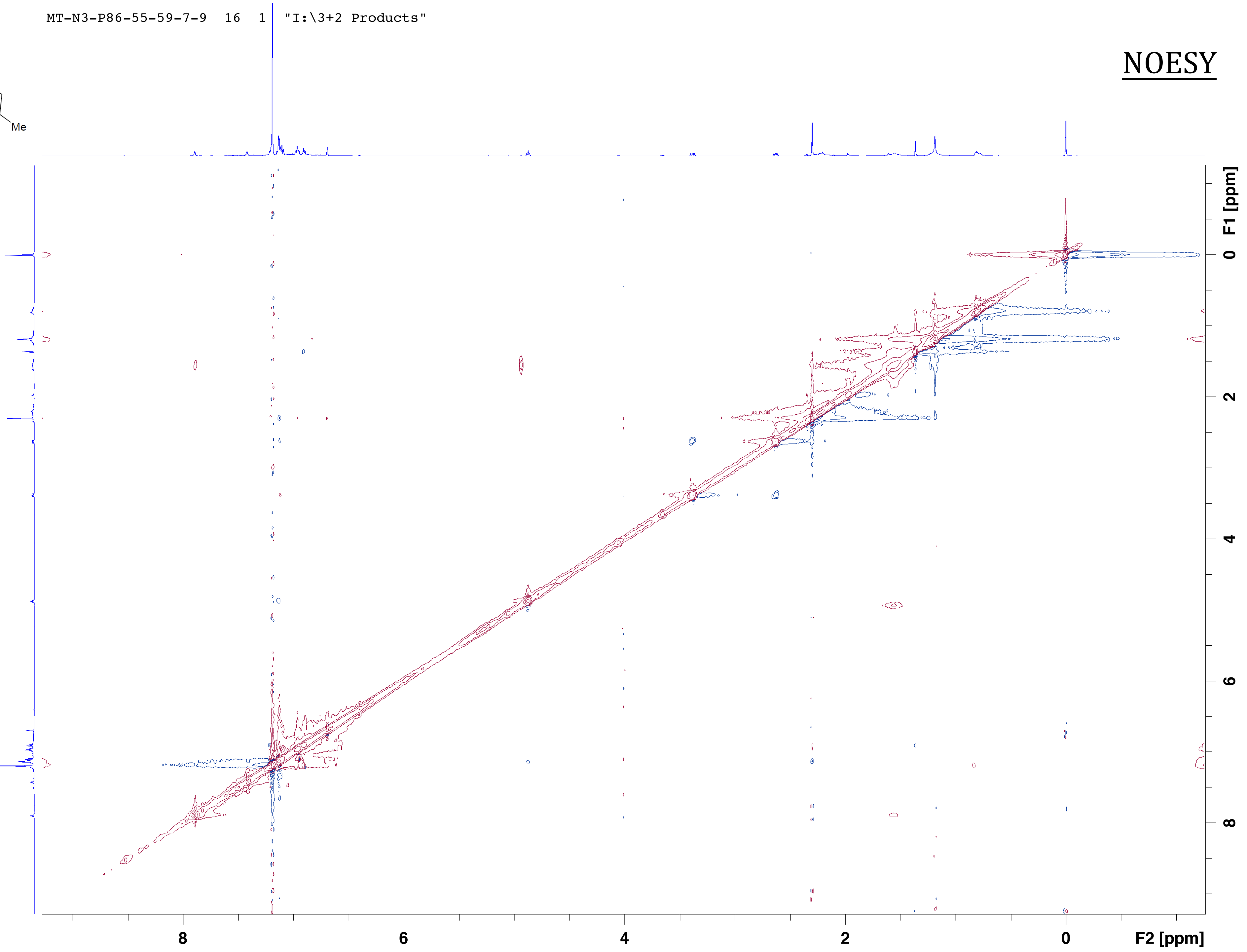

MT-N3-P42-TT6-10-16-17 10 1 "I:\3+2 Products"

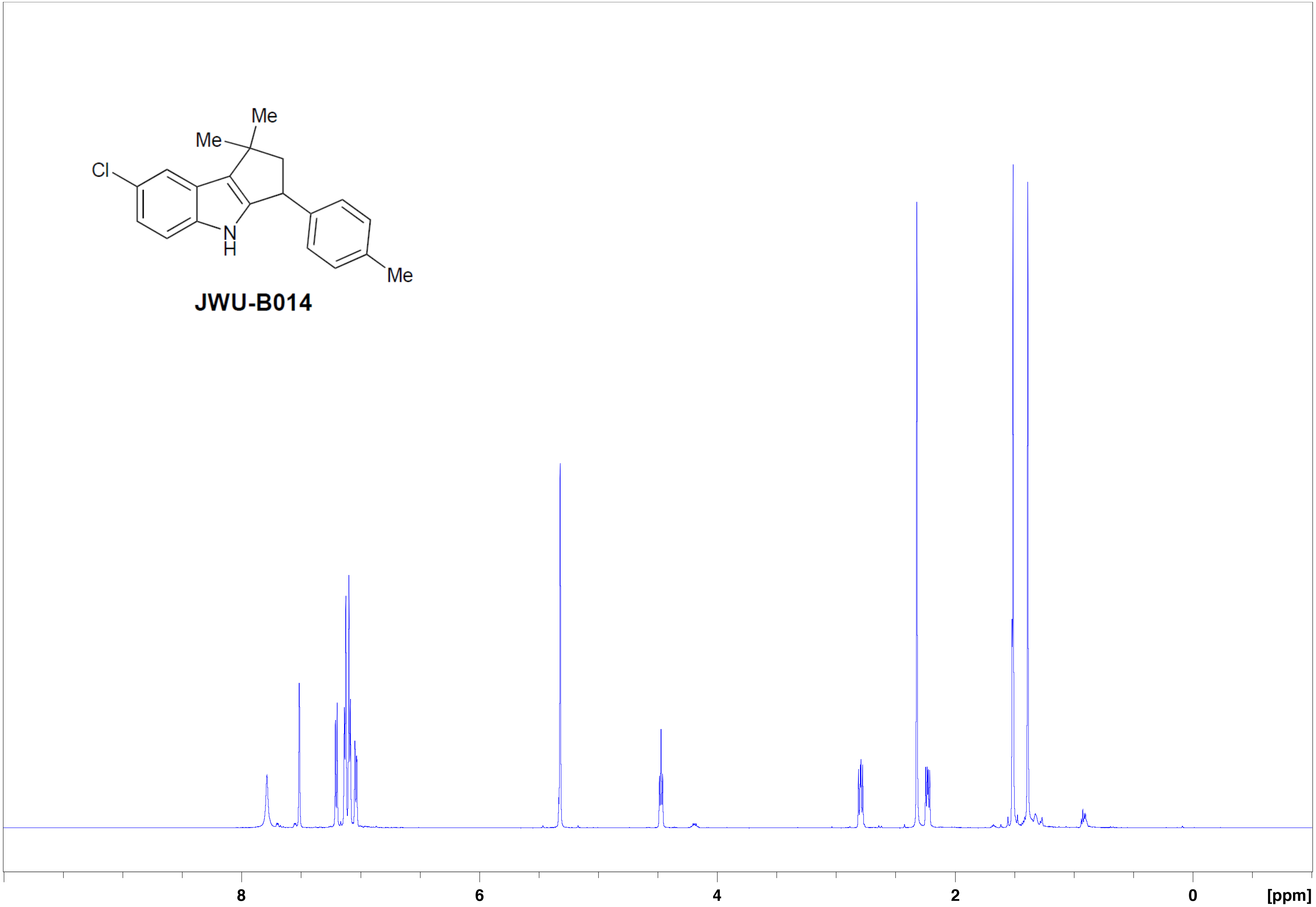

MT-N3-P42-TT6-8-16-17   10   1   "I:\3+2 Products\MT-N3-P42-TT6-10-16-17"

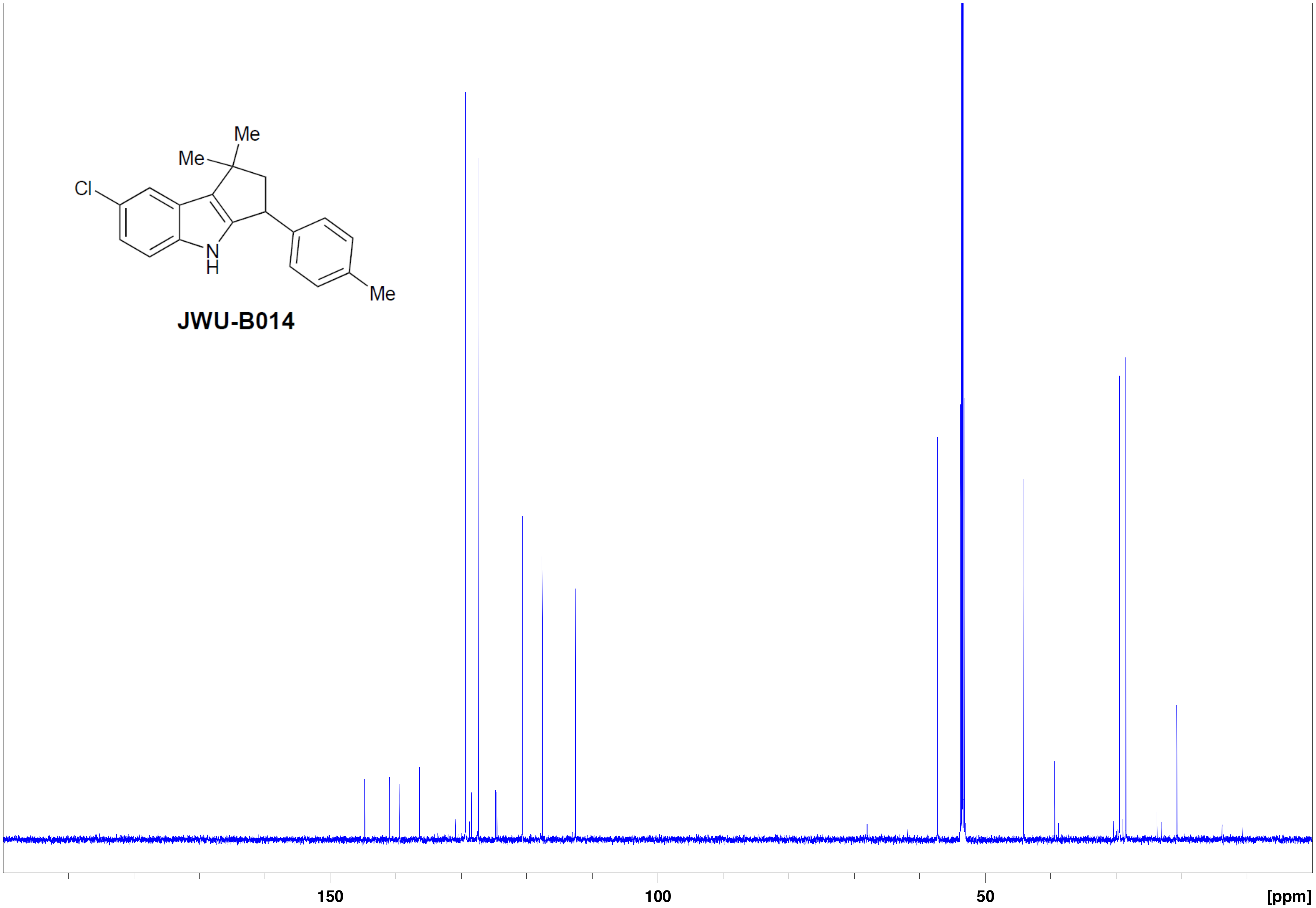

JWU-B014

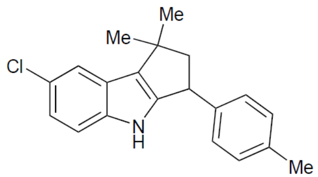
Me
Me
Cl
N
H
Me

JWU-B014

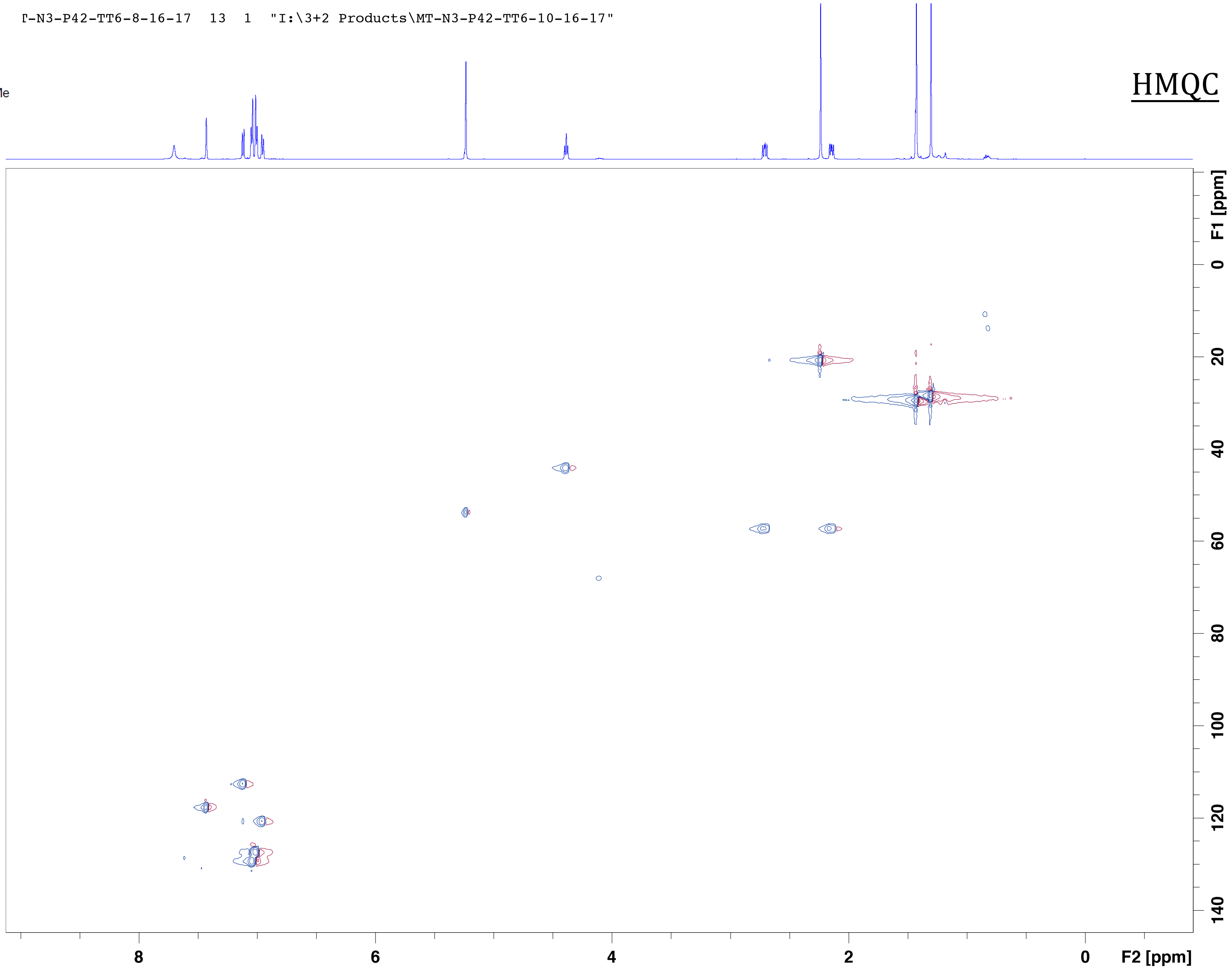
Γ-N3-P42-TT6-8-16-17 13 1 "I:\3+2 Products\MT-N3-P42-TT6-10-16-17"
HMQC
F1 [ppm]
F2 [ppm]
0
20
40
60
80
100
120
140
8
6
4
2
0

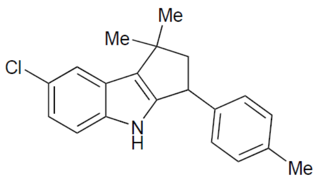

JWU-B014

HMBC

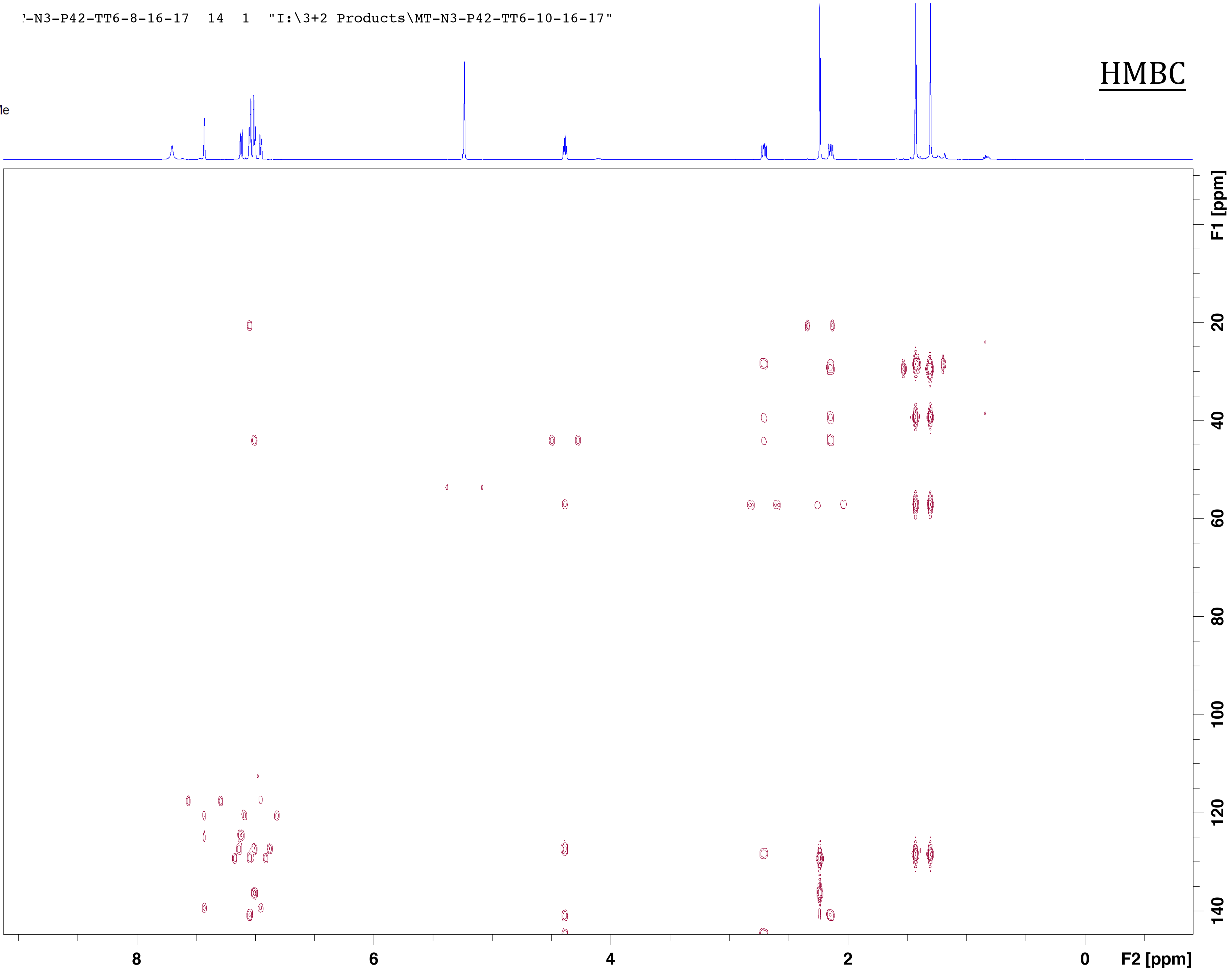

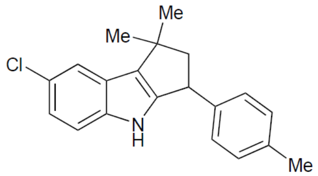

JWU-B014

COSY

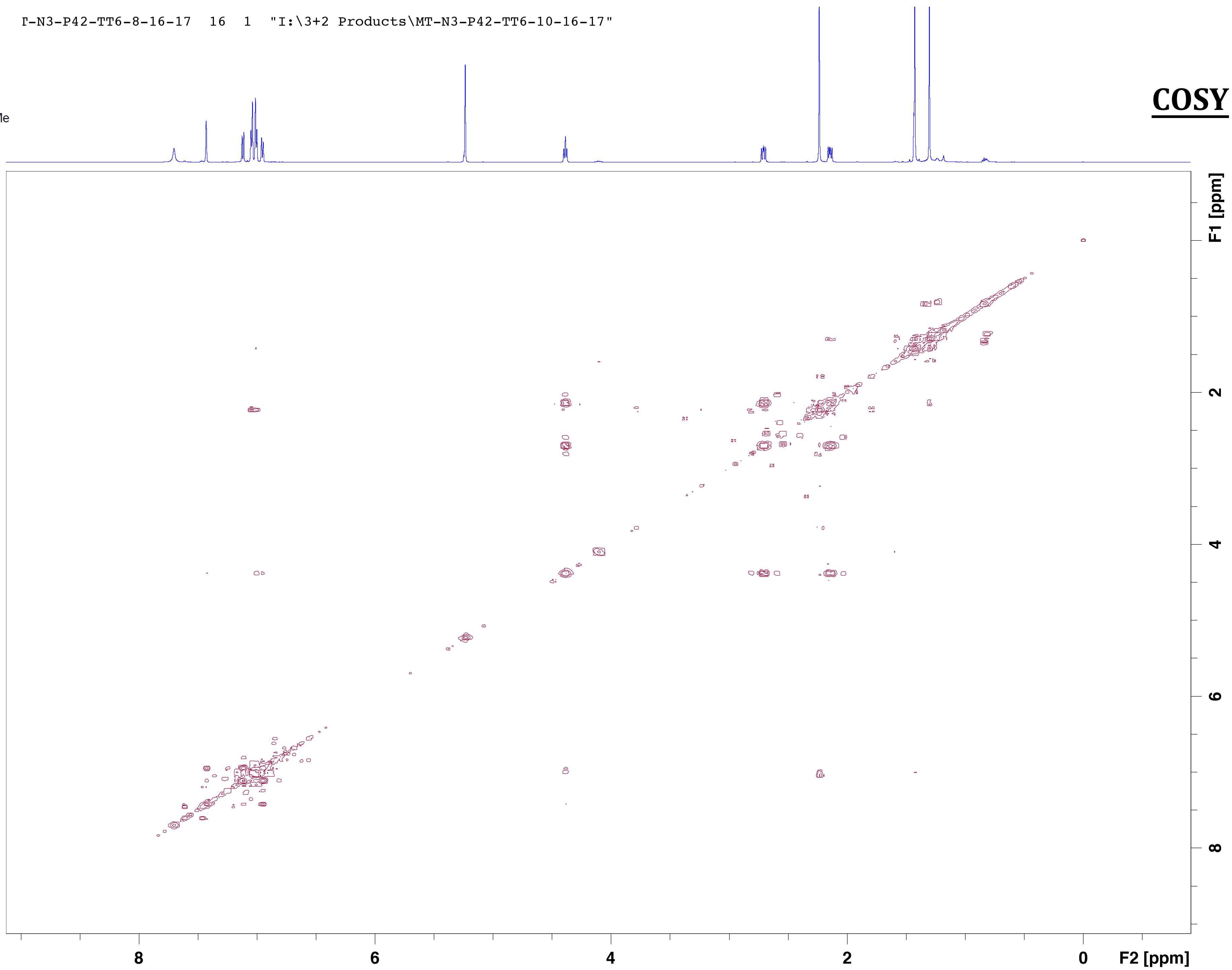

Structural Data

Date: March 17, 2015

Submitter: Jimmy Wu (Dartmouth)

Sample Reference Number: (-)-JWU-A021

X-ray Number: JW315a

Center Crystallographic for Research
Michigan State University
Department of Chemistry
East Lansing, MI 48824
Dr. Richard J. Staples
staples@chemistry.msu.edu
(Shannon M. Biros, PhD)

Introduction:
Single crystal study to confirm the identity and chirality of the sample submitted. There are eight crystallographically unique molecules of $C_{16}H_{16}ClN$ in the asymmetric unit. Each molecule is labeled with the same atom numbering scheme, but has a different suffix (a-h). One molecule (f) was disordered over two positions with a 60:40 ratio; its counterpart is labeled with the same atom numbering scheme using a z-suffix. Shown below is a drawing of molecule (c) as a representative example.
The chirality of the compound was established by anomalous dispersion techniques using copper radiation with the use of the Flack parameter and Bayesian statistics of Bijvoet differences. Chiral carbons are labeled in the molecule below; C9 = *S*, C11 = *R*.

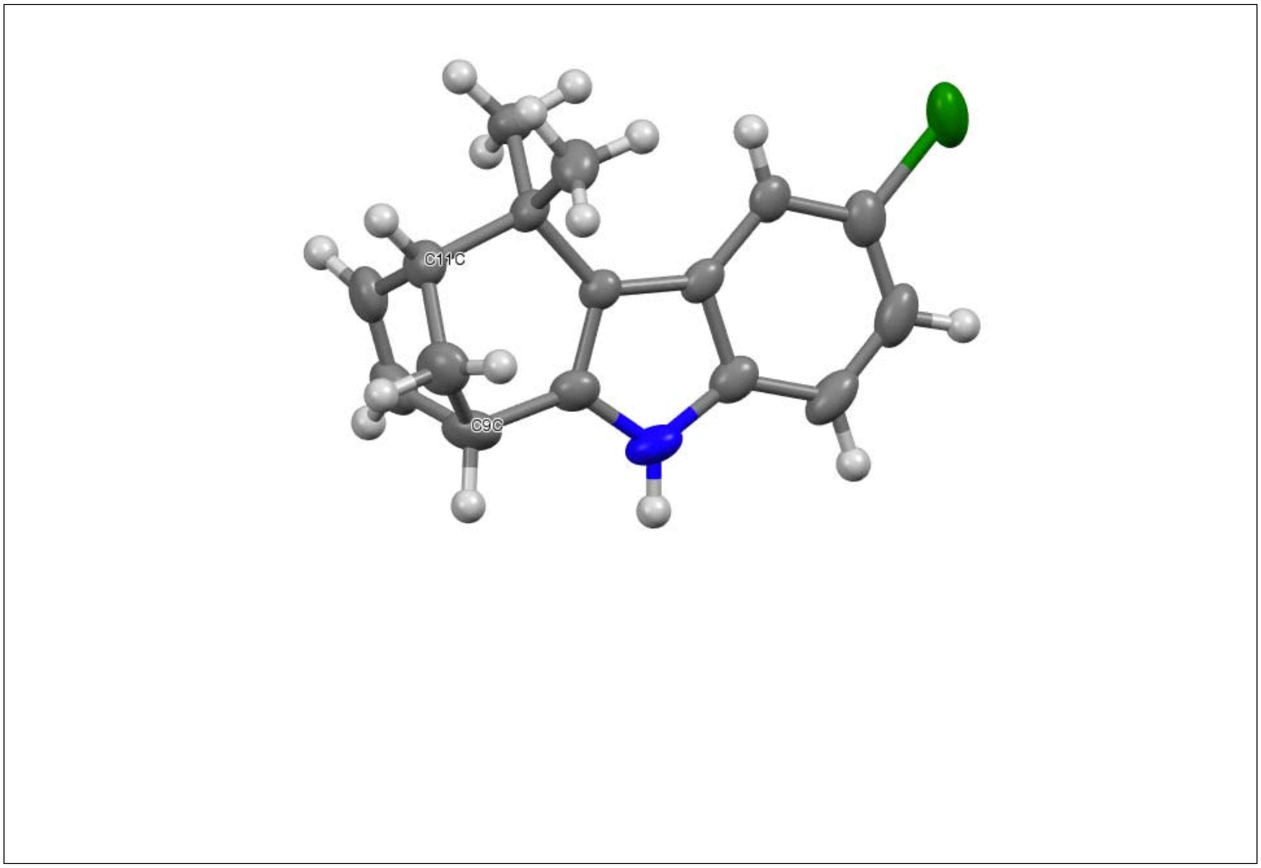

Experimental Section:

A yellow block crystal with dimensions 0.239 x 0.156 x 0.132 mm was mounted on a Nylon loop using very small amount of paratone oil.

Data were collected using a Bruker CCD (charge coupled device) based diffractometer equipped with an Oxford Cryostream low-temperature apparatus operating at 173 K. Data were measured using omega and phi scans of 1.0° per frame for 10 s. The total number of images was based on results from the program COSMO[1] where redundancy was expected to be 4.0 and completeness of 100% out to 0.83 Å. Cell parameters were retrieved using APEX II software[2] and refined using SAINT on all observed reflections. Data reduction was performed using the SAINT software[3] which corrects for Lp. Scaling and absorption corrections were applied using SADABS[4] multi-scan technique, supplied by George Sheldrick. The structures are solved by the direct method using the SHELXS-97 program and refined by least squares method on F^2, SHELXL- 97[5], which are incorporated in OLEX2.[6]

The structure was solved in the space group $P2_1$ (# 4). All non-hydrogen atoms are refined anisotropically. Hydrogens were calculated by geometrical methods and refined as a riding model. The Flack[7] parameter is used to determine chirality of the crystal studied, the value should be near zero, a value of one is the other enantiomer and a value of 0.5 is racemic. The Flack parameter was refined to -0.017(9), confirming the absolute stereochemistry. Determination of absolute structure using Bayesian statistics on Bijvoet differences using the program within Platon[8] also report that we have the correct enantiomer based on this comparison.[9] The crystal used for the diffraction study showed no decomposition during data collection. All drawings are done at 50% ellipsoids.

Acknowledgement. The CCD based x-ray diffractometer at Michigan State University were upgraded and/or replaced by departmental funds.

References

1. COSMO V1.61, *Software for the CCD Detector Systems for Determining Data Collection Parameters.* Bruker Analytical X-ray Systems, Madison, WI (2009).

2. APEX2 V2010.11-3. Software *for the CCD Detector System*; Bruker Analytical X-ray Systems, Madison, WI (2010).

3. SAINT V 7.68A Software *for the Integration of CCD Detector System* Bruker Analytical X-ray Systems, Madison, WI (2010).

4. SADABS V2.008/2 Program for absorption corrections using Bruker-AXS CCD based on the method of Robert Blessing; Blessing, R.H. Acta Cryst. A51, 1995, 33-38.

5. Sheldrick, G.M. "A short history of SHELX". *Acta Cryst.* **A64**, 2008, 112-122.

6. O. V. Dolomanov, L. J. Bourhis, R. J. Gildea, J. A. K. Howard and H. Puschmann, OLEX2: a complete structure solution, refinement and analysis program. J. Appl. Cryst. (2009). 42, 339-341.

7. Flack, H. D. *Acta Cryst.* **A39**, 1983, 876-881.

8. Spek, A.L. (2003), J.Appl.Cryst. **36**, 7-13.

9 Hooft, R.W.W.; Straver, L.H. & A. L. Spek, A.L.. *J. Appl. Cryst.* **41**, *2008,* 96-103

[a] Obtained with graphite monochromated Mo Kα (λ= 0.71073 Å) radiation.
[b] $R1 = \sum ||F_o| - |F_c / |F_o|$ [c] $wR_2 = \{ | [w(F_o^2 - F_c^2)^2 . \{\sum[w(F_o^2)^2]\}^{1/2}$.

The following are 50% thermal ellipsoidal drawings of one molecule (c) in the asymmetric cell with various amount of labeling.

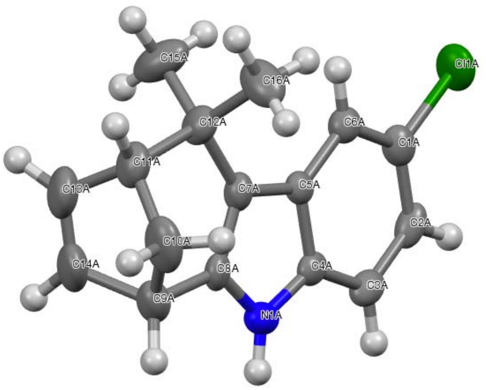

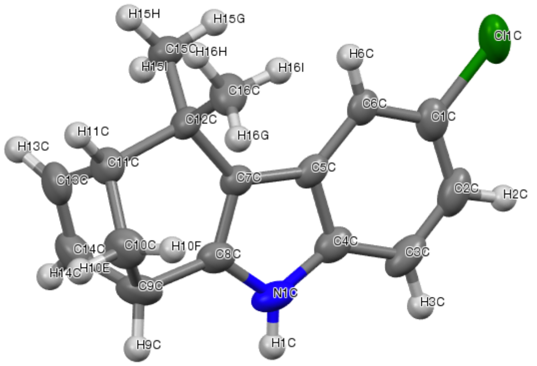

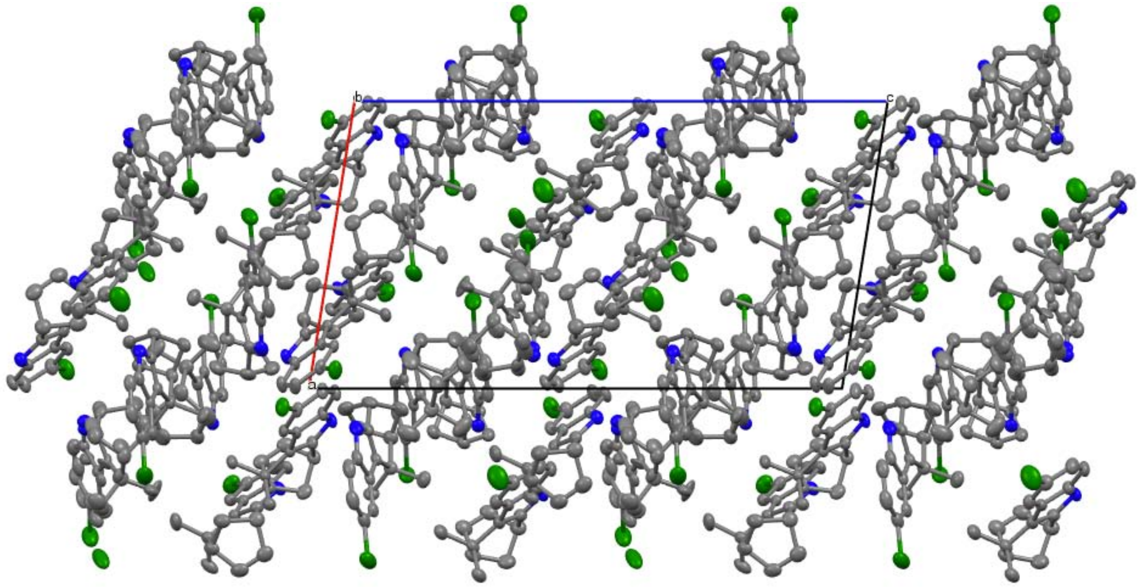

This is a drawing of the packing along the *b*-axis; hydrogen atoms have been omitted for clarity.

Table 1 Crystal data and structure refinement for JW315a.

Identification code	JW315a
Empirical formula	$C_{16}H_{16}ClN$
Formula weight	257.75
Temperature/K	173.0
Crystal system	monoclinic
Space group	$P2_1$
a/Å	12.4101(3)
b/Å	18.9939(3)
c/Å	22.7815(4)
α/°	90
β/°	99.0520(10)
γ/°	90
Volume/Å^3	5303.09(18)
Z	16
ρ_{calc}g/cm^3	1.291
μ/mm^{-1}	2.372
F(000)	2176.0
Crystal size/mm^3	0.239 × 0.156 × 0.132
Radiation	CuKα (λ = 1.54178)
2Θ range for data collection/°	3.928 to 144.904
Index ranges	-15 ≤ h ≤ 15, -23 ≤ k ≤ 23, -28 ≤ l ≤ 28
Reflections collected	66305
Independent reflections	20467 [R_{int} = 0.0891, R_{sigma} = 0.0731]
Data/restraints/parameters	20467/522/1477
Goodness-of-fit on F^2	1.009
Final R indexes [I>=2σ (I)]	R_1 = 0.0522, wR_2 = 0.1151
Final R indexes [all data]	R_1 = 0.0758, wR_2 = 0.1280
Largest diff. peak/hole / e Å^{-3}	0.49/-0.54
Flack parameter	0.017(9)

Table 2 Fractional Atomic Coordinates (×10^4) and Equivalent Isotropic Displacement Parameters (Å^2×10^3) for JW315a. U_{eq} is defined as 1/3 of of the trace of the orthogonalised U_{IJ} tensor.

**Atom**	***x***	***y***	***z***	**U(eq)**
Cl1A	3006.9(13)	6780.1(8)	2095.4(7)	49.6(4)
N1A	-1227(3)	5354(2)	1701(2)	36.9(10)
C1A	1708(5)	6368(3)	1963(2)	36.0(12)
C2A	812(5)	6774(3)	1713(2)	36.7(12)

C3A	-212(5)	6477(3)	1609(2)	36.0(12)
C4A	-316(4)	5770(3)	1756(2)	30.3(11)
C5A	588(4)	5359(3)	2012(2)	27.5(10)
C6A	1625(4)	5675(3)	2116(2)	32.6(11)
C7A	167(4)	4664(3)	2104(2)	32.8(11)
C8A	-930(4)	4695(3)	1914(2)	32.3(11)
C9A	-1679(5)	4083(3)	1951(3)	46.9(14)
C10A	-976(6)	3413(4)	1821(3)	63(2)
C11A	-146(5)	3419(3)	2391(3)	47.0(14)
C12A	736(5)	4001(3)	2366(3)	41.9(13)
C13A	-839(6)	3597(4)	2847(3)	66.6(19)
C14A	-1686(6)	3943(4)	2607(3)	67(2)
C15A	1348(7)	4161(4)	2995(3)	68(2)
C16A	1558(6)	3739(3)	1985(4)	61.0(19)
Cl1B	6660.4(14)	7221.4(8)	1166.4(7)	53.5(4)
N1B	6521(4)	4347(2)	264(2)	37.8(10)
C1B	6680(5)	6365(3)	889(2)	37.2(12)
C2B	7239(4)	6235(3)	415(2)	38.7(13)
C3B	7250(4)	5565(3)	183(2)	37.8(12)
C4B	6676(4)	5040(3)	423(2)	33.0(11)
C5B	6096(4)	5165(3)	902(2)	28.8(10)
C6B	6122(4)	5849(3)	1139(2)	32.7(11)
C7B	5582(4)	4513(3)	1017(2)	30.5(10)
C8B	5860(4)	4042(3)	625(2)	32.7(11)
C9B	5443(5)	3293(3)	588(3)	42.9(13)
C10B	5386(5)	3093(3)	1235(3)	43.0(13)
C11B	4421(4)	3560(3)	1346(3)	40.4(13)
C12B	4795(4)	4346(3)	1447(2)	36.6(12)
C13B	3671(5)	3464(3)	757(3)	46.5(14)
C14B	4234(5)	3305(3)	330(3)	47.4(14)
C15B	3812(5)	4848(3)	1334(3)	48.5(15)
C16B	5354(6)	4429(4)	2096(3)	54.0(16)
Cl1C	4016.6(14)	3823.9(9)	8376.9(8)	55.4(4)
N1C	8621(4)	4598(3)	8996(2)	42.1(11)
C1C	5417(5)	4010(3)	8569(3)	41.8(13)
C2C	6110(6)	3497(3)	8841(3)	48.7(15)
C3C	7203(6)	3636(3)	9002(3)	47.3(15)
C4C	7589(5)	4309(3)	8885(2)	36.7(12)
C5C	6883(4)	4826(3)	8590(2)	31.0(11)
C6C	5772(4)	4668(3)	8429(2)	32.3(11)
C7C	7539(4)	5439(3)	8525(2)	29.1(10)
C8C	8581(4)	5273(3)	8774(2)	36.5(12)

C9C	9506(5)	5790(4)	8815(3)	48.8(15)
C10C	9284(5)	6197(4)	8222(3)	51.0(16)
C11C	8288(5)	6623(3)	8320(3)	41.1(12)
C12C	7237(4)	6142(3)	8229(2)	33.8(11)
C13C	8615(5)	6835(3)	8961(3)	47.2(14)
C14C	9298(5)	6370(3)	9242(3)	51.5(15)
C15C	6335(4)	6503(3)	8512(3)	41.6(13)
C16C	6831(5)	6042(3)	7572(3)	46.4(14)
Cl1D	6895(2)	450.6(9)	6189.5(11)	82.8(7)
N1D	6318(4)	3322(3)	5309(2)	40.8(11)
C1D	6716(5)	1308(3)	5902(3)	43.8(14)
C2D	7296(5)	1484(4)	5449(3)	48.1(15)
C3D	7199(5)	2152(4)	5218(2)	44.1(14)
C4D	6539(4)	2628(3)	5455(2)	36.2(12)
C5D	5941(4)	2448(3)	5915(2)	31.7(11)
C6D	6049(5)	1760(3)	6147(2)	39.1(12)
C7D	5330(4)	3061(3)	6029(2)	31.9(11)
C8D	5582(4)	3572(3)	5654(2)	37.1(12)
C9D	5155(5)	4311(3)	5660(3)	47.4(14)
C10D	3967(5)	4229(4)	5774(3)	53.2(16)
C11D	4184(5)	3967(3)	6423(3)	46.0(14)
C12D	4509(4)	3175(3)	6454(3)	37.7(12)
C13D	5137(5)	4429(3)	6675(3)	48.6(14)
C14D	5678(5)	4633(3)	6253(3)	48.7(15)
C15D	4994(5)	2967(3)	7094(3)	47.5(14)
C16D	3495(5)	2721(3)	6252(3)	46.5(15)
Cl1E	9334.9(17)	9852.4(8)	5388.4(8)	59.8(4)
N1E	8997(4)	6959(2)	4509(2)	36.8(10)
C1E	9243(5)	8986(3)	5105(3)	41.5(13)
C2E	9844(5)	8828(3)	4650(3)	45.6(14)
C3E	9799(5)	8154(3)	4421(3)	40.6(13)
C4E	9173(4)	7656(3)	4655(2)	31.7(11)
C5E	8556(4)	7813(3)	5116(2)	30.5(11)
C6E	8613(4)	8506(3)	5343(2)	33.7(11)
C7E	8002(4)	7183(3)	5232(2)	29.6(10)
C8E	8283(4)	6681(3)	4853(2)	34.6(11)
C9E	7855(5)	5942(3)	4831(3)	46.9(14)
C10E	7746(5)	5767(3)	5476(3)	49.4(15)
C11E	6794(5)	6259(3)	5562(3)	45.3(14)
C12E	7194(4)	7039(3)	5651(3)	39.2(13)
C13E	6058(5)	6152(3)	4972(3)	50.6(15)
C14E	6647(5)	5979(3)	4561(3)	51.1(15)

C15E	6210(5)	7544(3)	5512(3)	48.8(15)
C16E	7728(5)	7147(4)	6303(3)	53.7(16)
Cl1F	5156(3)	9335.4(17)	6338.3(13)	70.9(9)
N1F	1135(11)	7702(7)	5878(9)	40(3)
C1F	3937(10)	8869(6)	6224(6)	40(2)
C2F	3038(11)	9222(6)	5892(6)	49(3)
C3F	2043(10)	8872(5)	5764(5)	44(2)
C4F	1993(11)	8175(5)	5951(6)	31(3)
C5F	2890(12)	7837(9)	6292(14)	32(3)
C6F	3882(8)	8199(5)	6422(5)	33(2)
C7F	2523(13)	7148(7)	6441(10)	28(4)
C8F	1482(13)	7074(8)	6143(11)	33(3)
C9F	823(11)	6434(7)	6158(7)	37(3)
C10F	1643(14)	5820(9)	6120(8)	48(4)
C11F	2351(13)	5894(8)	6736(8)	46(3)
C12F	3141(13)	6536(8)	6759(9)	40(4)
C13F	1466(16)	6017(13)	7103(8)	46(4)
C14F	611(16)	6326(9)	6793(8)	42(4)
C15F	3560(20)	6730(15)	7419(8)	67(7)
C16F	4123(18)	6338(11)	6446(12)	55(5)
Cl1Z	5933(4)	8893(3)	6606(2)	69.1(14)
N1Z	1442(14)	7916(9)	5918(11)	36(4)
C1Z	4560(12)	8643(8)	6401(7)	45(3)
C2Z	3807(17)	9122(9)	6083(10)	48(4)
C3Z	2733(16)	8935(8)	5909(8)	42(4)
C4Z	2441(15)	8245(9)	6047(11)	36(4)
C5Z	3192(16)	7782(12)	6372(19)	29(4)
C6Z	4272(13)	7982(8)	6547(9)	41(4)
C7Z	2625(18)	7123(11)	6388(18)	33(6)
C8Z	1564(18)	7245(11)	6158(19)	33(5)
C9Z	720(19)	6693(11)	6139(12)	50(6)
C10Z	1310(20)	5996(12)	6052(11)	52(6)
C11Z	2052(19)	5930(14)	6657(13)	57(6)
C12Z	3039(19)	6441(12)	6691(12)	37(5)
C13Z	1270(30)	6160(20)	7059(14)	62(8)
C14Z	520(30)	6573(14)	6776(15)	54(7)
C15Z	3550(20)	6590(20)	7343(12)	52(7)
C16Z	3940(30)	6137(16)	6362(18)	53(7)
Cl1G	3039.4(14)	1003.8(9)	7182.2(8)	61.1(4)
N1G	-1298(3)	2261(2)	6715.6(19)	33.9(10)
C1G	1712(5)	1354(3)	7035(3)	42.4(14)
C2G	885(5)	923(3)	6759(3)	44.8(14)

C3G	-170(5)	1188(3)	6634(2)	40.6(13)
C4G	-353(4)	1881(3)	6787(2)	33.4(12)
C5G	495(4)	2324(3)	7072(2)	29.2(10)
C6G	1560(5)	2043(3)	7199(2)	35.8(12)
C7G	2(4)	2983(3)	7166(2)	28.8(10)
C8G	-1082(4)	2918(3)	6949(2)	30.5(11)
C9G	-1893(4)	3507(3)	6938(2)	36.8(12)
C10G	-1518(5)	3900(3)	7528(3)	41.8(13)
C11G	-449(5)	4228(3)	7395(2)	36.7(12)
C12G	477(4)	3666(3)	7444(2)	31.5(11)
C13G	-806(4)	4463(3)	6754(3)	38.0(12)
C14G	-1617(4)	4055(3)	6497(2)	36.4(11)
C15G	1415(4)	3929(3)	7135(2)	38.2(12)
C16G	926(5)	3545(3)	8109(2)	45.1(13)
Cl1H	644.6(13)	2852.4(7)	9559.9(6)	45.4(3)
N1H	1106(4)	5702(2)	10517.4(19)	33.8(10)
C1H	743(4)	3712(3)	9848(2)	31.4(11)
C2H	86(5)	3883(3)	10263(2)	39.3(13)
C3H	143(5)	4551(3)	10515(2)	38.4(13)
C4H	876(4)	5024(3)	10333(2)	31.3(11)
C5H	1540(4)	4860(3)	9899(2)	27.9(10)
C6H	1474(4)	4181(3)	9654(2)	29.8(10)
C7H	2180(4)	5479(3)	9836(2)	28.2(10)
C8H	1891(4)	5964(3)	10224(2)	32.4(11)
C9H	2393(5)	6686(3)	10296(3)	36.9(12)
C10H	3596(5)	6580(3)	10223(3)	42.9(13)
C11H	3439(4)	6410(3)	9552(3)	36.4(12)
C12H	3037(4)	5629(3)	9437(2)	30.8(11)
C13H	2555(5)	6937(3)	9324(3)	43.1(13)
C14H	1975(5)	7100(3)	9732(3)	45.0(14)
C15H	2562(4)	5532(3)	8778(2)	37.6(12)
C16H	4023(4)	5134(3)	9599(2)	37.4(12)

Table 3 Anisotropic Displacement Parameters ($Å^2 \times 10^3$) for JW315a. The Anisotropic displacement factor exponent takes the form: $-2\pi^2[h^2a^{*2}U_{11}+2hka^*b^*U_{12}+\ldots]$.

Atom	U_{11}	U_{22}	U_{33}	U_{23}	U_{13}	U_{12}
Cl1A	55.0(8)	39.7(7)	54.4(9)	-4.5(6)	9.6(7)	-21.8(6)
N1A	32(2)	47(3)	32(2)	0(2)	4.5(18)	2(2)
C1A	46(3)	34(3)	30(3)	-10(2)	11(2)	-7(2)
C2A	66(4)	20(2)	26(3)	-3(2)	13(2)	0(2)
C3A	46(3)	34(3)	30(3)	-1(2)	10(2)	12(2)
C4A	37(3)	35(3)	19(2)	-2(2)	8(2)	5(2)

C5A	31(2)	30(3)	23(2)	-3.5(19)	8.8(19)	0.1(19)
C6A	40(3)	30(3)	29(3)	-3(2)	8(2)	1(2)
C7A	43(3)	30(3)	28(3)	0(2)	10(2)	-6(2)
C8A	42(3)	35(3)	21(2)	1(2)	11(2)	-4(2)
C9A	45(3)	56(4)	41(3)	2(3)	10(3)	-14(3)
C10A	75(5)	51(4)	65(4)	-5(3)	23(4)	-35(4)
C11A	62(4)	35(3)	47(3)	8(3)	14(3)	-7(3)
C12A	49(3)	30(3)	45(3)	9(2)	4(3)	-5(2)
C13A	75(5)	73(5)	55(4)	24(4)	23(4)	-13(4)
C14A	58(4)	90(6)	56(4)	22(4)	18(3)	-23(4)
C15A	82(5)	46(4)	66(5)	12(3)	-24(4)	-1(3)
C16A	57(4)	33(3)	99(6)	9(3)	29(4)	6(3)
Cl1B	70.3(10)	29.7(7)	57.5(9)	-2.9(6)	0.4(7)	-8.5(6)
N1B	40(2)	36(2)	41(3)	-3(2)	17(2)	2.9(19)
C1B	45(3)	32(3)	33(3)	1(2)	-2(2)	-4(2)
C2B	37(3)	41(3)	38(3)	10(2)	3(2)	-9(2)
C3B	36(3)	45(3)	34(3)	5(2)	12(2)	-8(2)
C4B	34(3)	38(3)	28(3)	0(2)	5(2)	1(2)
C5B	27(2)	33(3)	26(2)	3(2)	2.9(19)	0.0(19)
C6B	32(2)	34(3)	32(3)	-3(2)	4(2)	-1(2)
C7B	28(2)	33(3)	30(3)	1(2)	4.7(19)	-2(2)
C8B	31(2)	30(3)	38(3)	-3(2)	6(2)	1(2)
C9B	50(3)	28(3)	51(3)	-5(2)	9(3)	5(2)
C10B	39(3)	33(3)	58(4)	10(3)	8(3)	-1(2)
C11B	37(3)	36(3)	49(3)	9(2)	11(2)	-6(2)
C12B	34(3)	40(3)	37(3)	0(2)	11(2)	-5(2)
C13B	38(3)	35(3)	64(4)	8(3)	3(3)	-9(2)
C14B	53(3)	32(3)	54(4)	-5(2)	1(3)	-7(2)
C15B	46(3)	39(3)	68(4)	1(3)	32(3)	0(3)
C16B	64(4)	69(4)	32(3)	-2(3)	17(3)	-14(3)
Cl1C	58.3(9)	49.5(9)	62.4(10)	-19.5(8)	22.0(8)	-20.7(7)
N1C	47(3)	42(3)	39(3)	11(2)	12(2)	19(2)
C1C	52(3)	40(3)	37(3)	-9(2)	19(3)	-9(3)
C2C	78(4)	31(3)	42(3)	-5(2)	25(3)	-6(3)
C3C	77(4)	31(3)	37(3)	7(2)	20(3)	13(3)
C4C	50(3)	35(3)	29(3)	3(2)	16(2)	8(2)
C5C	44(3)	27(2)	25(2)	0(2)	14(2)	6(2)
C6C	43(3)	30(3)	26(2)	-7(2)	12(2)	1(2)
C7C	33(2)	29(2)	27(2)	3(2)	12(2)	3(2)
C8C	36(3)	41(3)	35(3)	3(2)	13(2)	8(2)
C9C	30(3)	62(4)	56(4)	14(3)	11(2)	8(2)
C10C	44(3)	55(4)	59(4)	11(3)	22(3)	-5(3)

C11C	41(3)	35(3)	48(3)	8(2)	10(2)	-5(2)
C12C	39(3)	25(2)	38(3)	4(2)	7(2)	2(2)
C13C	46(3)	41(3)	54(4)	-4(3)	7(3)	-17(3)
C14C	40(3)	61(4)	52(4)	2(3)	1(3)	-16(3)
C15C	37(3)	35(3)	51(3)	-1(2)	4(2)	7(2)
C16C	58(4)	42(3)	37(3)	10(3)	2(3)	-5(3)
Cl1D	125.0(18)	33.8(9)	97.8(16)	8.2(9)	43.2(14)	12.2(10)
N1D	41(2)	50(3)	34(2)	10(2)	15(2)	3(2)
C1D	51(3)	32(3)	47(3)	-1(3)	4(3)	1(3)
C2D	56(4)	50(4)	39(3)	-8(3)	7(3)	14(3)
C3D	48(3)	59(4)	27(3)	2(3)	11(2)	8(3)
C4D	38(3)	40(3)	31(3)	0(2)	8(2)	3(2)
C5D	29(2)	38(3)	29(3)	-4(2)	6(2)	-2(2)
C6D	45(3)	39(3)	33(3)	-6(2)	6(2)	-6(2)
C7D	32(2)	34(3)	30(3)	-1(2)	6(2)	-4(2)
C8D	33(3)	42(3)	36(3)	-3(2)	4(2)	2(2)
C9D	48(3)	41(3)	55(4)	8(3)	10(3)	6(3)
C10D	43(3)	48(4)	67(4)	2(3)	4(3)	12(3)
C11D	37(3)	47(3)	57(4)	-11(3)	16(3)	-1(2)
C12D	37(3)	41(3)	38(3)	-9(2)	14(2)	-2(2)
C13D	51(3)	35(3)	62(4)	-16(3)	17(3)	1(2)
C14D	46(3)	33(3)	69(4)	-4(3)	15(3)	3(2)
C15D	46(3)	59(4)	41(3)	-12(3)	16(2)	-3(3)
C16D	41(3)	54(4)	47(3)	-12(3)	17(3)	-9(3)
Cl1E	90.9(12)	31.5(7)	63.9(10)	-7.1(7)	33.5(9)	-3.4(8)
N1E	43(2)	35(2)	35(2)	-4.7(19)	12(2)	3.0(19)
C1E	57(3)	32(3)	38(3)	-1(2)	15(3)	-4(3)
C2E	61(4)	40(3)	40(3)	2(3)	23(3)	-8(3)
C3E	50(3)	42(3)	34(3)	-1(2)	22(3)	-5(3)
C4E	33(3)	33(3)	30(3)	-2(2)	8(2)	2(2)
C5E	33(2)	33(3)	25(2)	3(2)	3.8(19)	0(2)
C6E	40(3)	32(3)	31(3)	-2(2)	11(2)	5(2)
C7E	33(2)	30(3)	25(2)	3(2)	2.1(19)	2(2)
C8E	37(3)	34(3)	33(3)	5(2)	6(2)	0(2)
C9E	51(3)	34(3)	56(4)	1(3)	8(3)	-4(2)
C10E	48(3)	38(3)	61(4)	16(3)	4(3)	-2(3)
C11E	40(3)	45(3)	51(4)	20(3)	9(3)	-6(2)
C12E	38(3)	42(3)	39(3)	10(2)	13(2)	1(2)
C13E	44(3)	44(3)	62(4)	14(3)	1(3)	-10(3)
C14E	57(4)	37(3)	56(4)	5(3)	-2(3)	-12(3)
C15E	39(3)	55(4)	56(4)	12(3)	20(3)	3(3)
C16E	59(4)	70(4)	35(3)	10(3)	18(3)	-3(3)

Cl1F	85(2)	71.3(19)	59.6(16)	-9.6(14)	21.3(15)	-50.0(17)
N1F	33(6)	48(7)	36(5)	2(6)	1(5)	-2(4)
C1F	59(6)	31(6)	33(7)	-3(5)	16(5)	-12(5)
C2F	75(7)	34(6)	40(6)	7(5)	11(6)	-8(5)
C3F	55(6)	39(5)	38(5)	4(4)	7(5)	10(5)
C4F	32(8)	34(5)	28(6)	1(5)	9(7)	-2(5)
C5F	33(7)	33(6)	29(8)	-1(5)	6(7)	-2(6)
C6F	34(5)	40(6)	25(5)	4(4)	4(4)	-11(4)
C7F	40(7)	30(6)	14(5)	0(5)	5(6)	-6(5)
C8F	39(6)	39(7)	21(6)	3(7)	3(4)	-3(5)
C9F	39(6)	38(7)	32(5)	-2(6)	-3(4)	-14(5)
C10F	64(9)	38(7)	45(7)	-4(6)	14(6)	-7(6)
C11F	59(8)	38(6)	41(7)	8(5)	7(5)	3(5)
C12F	45(7)	36(7)	38(9)	11(6)	5(5)	-2(5)
C13F	58(8)	39(9)	41(6)	15(5)	8(5)	-10(7)
C14F	39(7)	54(11)	33(6)	4(7)	7(5)	-19(7)
C15F	91(14)	72(11)	27(7)	15(7)	-24(7)	6(10)
C16F	60(9)	39(11)	69(11)	9(8)	16(8)	15(7)
Cl1Z	81(3)	67(3)	67(3)	-30(2)	36(2)	-49(3)
N1Z	28(10)	40(11)	39(9)	17(11)	9(10)	10(6)
C1Z	54(8)	39(8)	47(9)	-8(7)	20(7)	-6(7)
C2Z	86(9)	21(8)	40(10)	-2(7)	22(7)	-14(7)
C3Z	70(10)	26(8)	35(8)	5(8)	21(9)	9(8)
C4Z	34(10)	31(7)	42(11)	-4(6)	4(9)	-2(7)
C5Z	36(10)	28(7)	25(12)	-4(6)	12(10)	7(7)
C6Z	46(8)	31(8)	42(10)	1(7)	-1(8)	-7(6)
C7Z	24(7)	35(8)	45(14)	-4(8)	22(7)	11(5)
C8Z	40(9)	25(9)	37(11)	-1(9)	12(8)	0(6)
C9Z	54(10)	53(13)	45(9)	-1(11)	13(7)	-18(9)
C10Z	67(15)	43(12)	49(10)	-4(10)	19(10)	-40(11)
C11Z	62(12)	52(11)	61(14)	17(10)	21(10)	-11(8)
C12Z	46(10)	35(9)	27(10)	0(7)	-1(7)	9(8)
C13Z	82(17)	57(19)	55(12)	18(11)	32(13)	3(13)
C14Z	45(10)	54(17)	64(11)	3(11)	14(9)	-16(10)
C15Z	29(10)	80(18)	50(11)	5(10)	15(8)	0(9)
C16Z	55(13)	35(16)	73(14)	7(12)	18(12)	17(11)
Cl1G	65.2(10)	61(1)	57.4(9)	16.0(8)	10.5(8)	32.6(8)
N1G	35(2)	32(2)	36(2)	-3.2(19)	7.0(18)	-10.9(18)
C1G	50(3)	42(3)	37(3)	11(2)	11(3)	14(3)
C2G	72(4)	27(3)	39(3)	8(2)	22(3)	6(3)
C3G	67(4)	24(3)	33(3)	2(2)	16(3)	-4(2)
C4G	47(3)	30(3)	26(3)	4(2)	13(2)	-4(2)

C5G	41(3)	27(2)	21(2)	4.5(19)	10.9(19)	0(2)
C6G	44(3)	37(3)	26(3)	7(2)	8(2)	3(2)
C7G	36(3)	29(3)	22(2)	3.9(19)	7.8(19)	-4(2)
C8G	35(3)	31(3)	26(2)	2(2)	9(2)	-5(2)
C9G	31(2)	42(3)	39(3)	-3(2)	11(2)	0(2)
C10G	42(3)	45(3)	41(3)	-11(3)	15(2)	0(2)
C11G	45(3)	32(3)	34(3)	-7(2)	13(2)	-5(2)
C12G	36(3)	34(3)	25(2)	1(2)	5.7(19)	-3(2)
C13G	40(3)	29(3)	44(3)	7(2)	6(2)	7(2)
C14G	36(3)	39(3)	35(3)	1(2)	5(2)	11(2)
C15G	34(3)	39(3)	40(3)	2(2)	2(2)	-7(2)
C16G	56(3)	42(3)	32(3)	-4(2)	-7(2)	-6(3)
Cl1H	73.0(9)	24.2(6)	39.0(7)	-4.9(5)	8.8(6)	-8.0(6)
N1H	46(2)	30(2)	29(2)	-8.8(18)	16.1(19)	-6.9(19)
C1H	49(3)	22(2)	23(2)	-3.9(19)	3(2)	0(2)
C2H	55(3)	35(3)	29(3)	3(2)	10(2)	-18(2)
C3H	54(3)	38(3)	27(3)	-5(2)	16(2)	-9(3)
C4H	40(3)	33(3)	22(2)	-3(2)	9(2)	-5(2)
C5H	31(2)	33(3)	20(2)	1(2)	5.1(19)	3(2)
C6H	35(2)	35(3)	19(2)	-2(2)	2.6(19)	1(2)
C7H	31(2)	29(2)	24(2)	2.2(19)	4.1(18)	-2.2(19)
C8H	39(3)	32(3)	27(2)	-3(2)	6(2)	-4(2)
C9H	47(3)	26(3)	39(3)	-6(2)	11(2)	-5(2)
C10H	48(3)	31(3)	47(3)	-1(2)	-2(3)	-12(2)
C11H	31(3)	38(3)	42(3)	7(2)	10(2)	-7(2)
C12H	29(2)	38(3)	27(2)	2(2)	7.5(19)	0(2)
C13H	43(3)	35(3)	50(3)	14(2)	3(3)	-8(2)
C14H	45(3)	25(3)	64(4)	7(2)	6(3)	-1(2)
C15H	39(3)	48(3)	27(3)	1(2)	10(2)	-5(2)
C16H	34(3)	41(3)	38(3)	-1(2)	8(2)	2(2)

Table 4 Bond Lengths for JW315a.

Atom	Atom	Length/Å	Atom	Atom	Length/Å
Cl1A	C1A	1.774(5)	C7E	C12E	1.514(7)
N1A	C4A	1.370(7)	C8E	C9E	1.499(8)
N1A	C8A	1.372(7)	C9E	C10E	1.535(9)
C1A	C2A	1.399(8)	C9E	C14E	1.530(9)
C1A	C6A	1.370(7)	C10E	C11E	1.543(9)
C2A	C3A	1.376(8)	C11E	C12E	1.566(8)
C3A	C4A	1.395(7)	C11E	C13E	1.514(9)
C4A	C5A	1.415(7)	C12E	C15E	1.547(8)
C5A	C6A	1.406(7)	C12E	C16E	1.540(8)

C5A	C7A	1.448(7)	C13E	C14E	1.318(9)
C7A	C8A	1.363(7)	Cl1F	C1F	1.737(11)
C7A	C12A	1.518(7)	N1F	C4F	1.382(13)
C8A	C9A	1.498(7)	N1F	C8F	1.375(13)
C9A	C10A	1.598(9)	C1F	C2F	1.416(16)
C9A	C14A	1.519(8)	C1F	C6F	1.355(13)
C10A	C11A	1.524(9)	C2F	C3F	1.391(15)
C11A	C12A	1.563(7)	C3F	C4F	1.396(12)
C11A	C13A	1.489(9)	C4F	C5F	1.408(14)
C12A	C15A	1.544(8)	C5F	C6F	1.401(13)
C12A	C16A	1.523(8)	C5F	C7F	1.443(12)
C13A	C14A	1.286(10)	C7F	C8F	1.368(13)
Cl1B	C1B	1.747(6)	C7F	C12F	1.514(13)
N1B	C4B	1.371(7)	C8F	C9F	1.469(13)
N1B	C8B	1.376(7)	C9F	C10F	1.558(14)
C1B	C2B	1.396(8)	C9F	C14F	1.526(13)
C1B	C6B	1.374(8)	C10F	C11F	1.540(14)
C2B	C3B	1.378(8)	C11F	C12F	1.561(14)
C3B	C4B	1.388(7)	C11F	C13F	1.499(14)
C4B	C5B	1.418(7)	C12F	C15F	1.555(15)
C5B	C6B	1.405(7)	C12F	C16F	1.552(14)
C5B	C7B	1.437(7)	C13F	C14F	1.316(15)
C7B	C8B	1.347(7)	Cl1Z	C1Z	1.759(14)
C7B	C12B	1.521(7)	N1Z	C4Z	1.378(17)
C8B	C9B	1.512(7)	N1Z	C8Z	1.386(17)
C9B	C10B	1.536(8)	C1Z	C2Z	1.419(19)
C9B	C14B	1.523(8)	C1Z	C6Z	1.360(17)
C10B	C11B	1.543(8)	C2Z	C3Z	1.38(2)
C11B	C12B	1.569(8)	C3Z	C4Z	1.408(17)
C11B	C13B	1.520(9)	C4Z	C5Z	1.405(17)
C12B	C15B	1.537(8)	C5Z	C6Z	1.391(17)
C12B	C16B	1.540(8)	C5Z	C7Z	1.439(17)
C13B	C14B	1.319(9)	C7Z	C8Z	1.358(17)
Cl1C	C1C	1.760(6)	C7Z	C12Z	1.520(17)
N1C	C4C	1.379(8)	C8Z	C9Z	1.478(18)
N1C	C8C	1.377(7)	C9Z	C10Z	1.539(19)
C1C	C2C	1.381(9)	C9Z	C14Z	1.525(19)
C1C	C6C	1.379(8)	C10Z	C11Z	1.54(2)
C2C	C3C	1.375(10)	C11Z	C12Z	1.555(18)
C3C	C4C	1.406(8)	C11Z	C13Z	1.505(19)
C4C	C5C	1.415(7)	C12Z	C15Z	1.546(18)
C5C	C6C	1.402(8)	C12Z	C16Z	1.548(18)

C5C	C7C	1.440(7)	C13Z	C14Z	1.30(2)
C7C	C8C	1.365(8)	Cl1G	C1G	1.759(6)
C7C	C12C	1.516(7)	N1G	C4G	1.365(7)
C8C	C9C	1.503(8)	N1G	C8G	1.366(7)
C9C	C10C	1.544(9)	C1G	C2G	1.385(9)
C9C	C14C	1.518(9)	C1G	C6G	1.382(8)
C10C	C11C	1.523(8)	C2G	C3G	1.389(9)
C11C	C12C	1.579(7)	C3G	C4G	1.390(7)
C11C	C13C	1.507(9)	C4G	C5G	1.421(8)
C12C	C15C	1.537(7)	C5G	C6G	1.412(8)
C12C	C16C	1.514(8)	C5G	C7G	1.426(7)
C13C	C14C	1.317(9)	C7G	C8G	1.364(7)
Cl1D	C1D	1.757(6)	C7G	C12G	1.521(7)
N1D	C4D	1.376(7)	C8G	C9G	1.502(7)
N1D	C8D	1.381(7)	C9G	C10G	1.544(8)
C1D	C2D	1.387(9)	C9G	C14G	1.522(8)
C1D	C6D	1.372(8)	C10G	C11G	1.539(7)
C2D	C3D	1.372(9)	C11G	C12G	1.560(8)
C3D	C4D	1.386(8)	C11G	C13G	1.524(8)
C4D	C5D	1.419(7)	C12G	C15G	1.535(7)
C5D	C6D	1.408(8)	C12G	C16G	1.547(7)
C5D	C7D	1.435(7)	C13G	C14G	1.331(8)
C7D	C8D	1.359(8)	Cl1H	C1H	1.757(5)
C7D	C12D	1.528(7)	N1H	C4H	1.371(7)
C8D	C9D	1.501(8)	N1H	C8H	1.360(7)
C9D	C10D	1.545(8)	C1H	C2H	1.380(8)
C9D	C14D	1.532(9)	C1H	C6H	1.392(7)
C10D	C11D	1.542(9)	C2H	C3H	1.389(8)
C11D	C12D	1.558(8)	C3H	C4H	1.386(7)
C11D	C13D	1.511(8)	C4H	C5H	1.418(7)
C12D	C15D	1.539(8)	C5H	C6H	1.402(7)
C12D	C16D	1.534(8)	C5H	C7H	1.439(7)
C13D	C14D	1.315(9)	C7H	C8H	1.364(7)
Cl1E	C1E	1.765(6)	C7H	C12H	1.530(6)
N1E	C4E	1.375(7)	C8H	C9H	1.505(7)
N1E	C8E	1.377(7)	C9H	C10H	1.541(8)
C1E	C2E	1.402(8)	C9H	C14H	1.526(8)
C1E	C6E	1.368(8)	C10H	C11H	1.545(8)
C2E	C3E	1.381(8)	C11H	C12H	1.573(7)
C3E	C4E	1.384(8)	C11H	C13H	1.515(8)
C4E	C5E	1.424(7)	C12H	C15H	1.535(7)
C5E	C6E	1.413(8)	C12H	C16H	1.542(7)

C5E	C7E	1.426(7)	C13H	C14H	1.300(8)
C7E	C8E	1.368(7)			

Table 5 Bond Angles for JW315a.

Atom	Atom	Atom	Angle/°	Atom	Atom	Atom	Angle/°
C4A	N1A	C8A	108.8(4)	C7E	C8E	N1E	110.1(5)
C2A	C1A	Cl1A	118.0(4)	C7E	C8E	C9E	123.3(5)
C6A	C1A	Cl1A	118.9(4)	C8E	C9E	C10E	104.8(5)
C6A	C1A	C2A	123.1(5)	C8E	C9E	C14E	106.9(5)
C3A	C2A	C1A	119.8(5)	C14E	C9E	C10E	99.6(5)
C2A	C3A	C4A	118.1(5)	C9E	C10E	C11E	100.1(5)
N1A	C4A	C3A	129.8(5)	C10E	C11E	C12E	110.8(5)
N1A	C4A	C5A	108.0(4)	C13E	C11E	C10E	100.0(5)
C3A	C4A	C5A	122.2(5)	C13E	C11E	C12E	112.1(5)
C4A	C5A	C7A	106.4(4)	C7E	C12E	C11E	108.5(5)
C6A	C5A	C4A	118.7(5)	C7E	C12E	C15E	109.7(4)
C6A	C5A	C7A	134.9(5)	C7E	C12E	C16E	110.9(5)
C1A	C6A	C5A	118.1(5)	C15E	C12E	C11E	109.6(5)
C5A	C7A	C12A	131.1(5)	C16E	C12E	C11E	109.3(5)
C8A	C7A	C5A	106.2(4)	C16E	C12E	C15E	108.8(5)
C8A	C7A	C12A	122.7(5)	C14E	C13E	C11E	109.9(6)
N1A	C8A	C9A	126.3(5)	C13E	C14E	C9E	110.0(6)
C7A	C8A	N1A	110.6(5)	C8F	N1F	C4F	109.2(10)
C7A	C8A	C9A	123.1(5)	C2F	C1F	Cl1F	115.7(9)
C8A	C9A	C10A	104.2(4)	C6F	C1F	Cl1F	121.2(10)
C8A	C9A	C14A	106.9(5)	C6F	C1F	C2F	123.1(10)
C14A	C9A	C10A	97.5(5)	C3F	C2F	C1F	119.0(10)
C11A	C10A	C9A	98.7(5)	C2F	C3F	C4F	118.0(10)
C10A	C11A	C12A	110.9(5)	N1F	C4F	C3F	130.5(12)
C13A	C11A	C10A	102.1(6)	N1F	C4F	C5F	107.3(9)
C13A	C11A	C12A	110.5(5)	C3F	C4F	C5F	122.1(11)
C7A	C12A	C11A	108.4(5)	C4F	C5F	C7F	106.9(9)
C7A	C12A	C15A	109.4(5)	C6F	C5F	C4F	119.0(10)
C7A	C12A	C16A	111.1(5)	C6F	C5F	C7F	134.1(11)
C15A	C12A	C11A	110.5(5)	C1F	C6F	C5F	118.6(10)
C16A	C12A	C11A	109.0(5)	C5F	C7F	C12F	130.8(11)
C16A	C12A	C15A	108.6(6)	C8F	C7F	C5F	106.6(10)
C14A	C13A	C11A	110.1(6)	C8F	C7F	C12F	121.8(11)
C13A	C14A	C9A	112.1(7)	N1F	C8F	C9F	126.2(11)
C4B	N1B	C8B	108.5(4)	C7F	C8F	N1F	109.5(10)
C2B	C1B	Cl1B	118.8(4)	C7F	C8F	C9F	124.0(11)
C6B	C1B	Cl1B	118.6(4)	C8F	C9F	C10F	104.3(11)

C6B	C1B	C2B	122.6(5)	C8F	C9F	C14F	108.2(12)
C3B	C2B	C1B	119.8(5)	C14F	C9F	C10F	99.3(10)
C2B	C3B	C4B	118.3(5)	C11F	C10F	C9F	99.6(9)
N1B	C4B	C3B	130.2(5)	C10F	C11F	C12F	111.6(11)
N1B	C4B	C5B	107.3(5)	C13F	C11F	C10F	99.1(11)
C3B	C4B	C5B	122.5(5)	C13F	C11F	C12F	111.8(12)
C4B	C5B	C7B	106.8(4)	C7F	C12F	C11F	108.3(11)
C6B	C5B	C4B	117.9(5)	C7F	C12F	C15F	110.4(13)
C6B	C5B	C7B	135.3(5)	C7F	C12F	C16F	110.0(13)
C1B	C6B	C5B	118.8(5)	C15F	C12F	C11F	109.3(13)
C5B	C7B	C12B	130.2(5)	C16F	C12F	C11F	109.3(12)
C8B	C7B	C5B	106.5(4)	C16F	C12F	C15F	109.5(13)
C8B	C7B	C12B	123.1(5)	C14F	C13F	C11F	111.7(11)
N1B	C8B	C9B	126.4(5)	C13F	C14F	C9F	108.9(11)
C7B	C8B	N1B	110.9(5)	C4Z	N1Z	C8Z	107.1(13)
C7B	C8B	C9B	122.7(5)	C2Z	C1Z	Cl1Z	119.6(12)
C8B	C9B	C10B	104.2(5)	C6Z	C1Z	Cl1Z	117.7(13)
C8B	C9B	C14B	108.4(4)	C6Z	C1Z	C2Z	122.7(14)
C14B	C9B	C10B	100.6(5)	C3Z	C2Z	C1Z	120.8(14)
C9B	C10B	C11B	99.7(5)	C2Z	C3Z	C4Z	116.6(15)
C10B	C11B	C12B	110.6(4)	N1Z	C4Z	C3Z	129.0(16)
C13B	C11B	C10B	99.8(5)	N1Z	C4Z	C5Z	109.1(12)
C13B	C11B	C12B	111.7(5)	C5Z	C4Z	C3Z	121.8(15)
C7B	C12B	C11B	108.0(4)	C4Z	C5Z	C7Z	105.8(13)
C7B	C12B	C15B	109.6(5)	C6Z	C5Z	C4Z	120.7(14)
C7B	C12B	C16B	111.1(4)	C6Z	C5Z	C7Z	133.1(16)
C15B	C12B	C11B	110.9(5)	C1Z	C6Z	C5Z	117.4(15)
C15B	C12B	C16B	108.9(5)	C5Z	C7Z	C12Z	128.5(16)
C16B	C12B	C11B	108.4(5)	C8Z	C7Z	C5Z	106.9(14)
C14B	C13B	C11B	110.9(5)	C8Z	C7Z	C12Z	124.0(15)
C13B	C14B	C9B	108.8(5)	N1Z	C8Z	C9Z	127.5(17)
C8C	N1C	C4C	108.8(5)	C7Z	C8Z	N1Z	110.4(15)
C2C	C1C	Cl1C	119.4(5)	C7Z	C8Z	C9Z	122.0(16)
C6C	C1C	Cl1C	117.6(5)	C8Z	C9Z	C10Z	105.4(16)
C6C	C1C	C2C	123.0(6)	C8Z	C9Z	C14Z	107(2)
C3C	C2C	C1C	120.2(6)	C14Z	C9Z	C10Z	98.3(15)
C2C	C3C	C4C	118.4(6)	C11Z	C10Z	C9Z	100.7(14)
N1C	C4C	C3C	131.3(5)	C10Z	C11Z	C12Z	110.6(16)
N1C	C4C	C5C	107.5(5)	C13Z	C11Z	C10Z	99.5(16)
C3C	C4C	C5C	121.2(5)	C13Z	C11Z	C12Z	112(2)
C4C	C5C	C7C	106.9(5)	C7Z	C12Z	C11Z	107.6(15)
C6C	C5C	C4C	119.1(5)	C7Z	C12Z	C15Z	110(2)

C6C	C5C	C7C	134.0(5)	C7Z	C12Z	C16Z	108.3(19)
C1C	C6C	C5C	118.1(5)	C15Z	C12Z	C11Z	111.3(17)
C5C	C7C	C12C	130.8(5)	C15Z	C12Z	C16Z	108.5(18)
C8C	C7C	C5C	106.5(5)	C16Z	C12Z	C11Z	111.3(17)
C8C	C7C	C12C	122.7(5)	C14Z	C13Z	C11Z	110.5(17)
N1C	C8C	C9C	127.1(5)	C13Z	C14Z	C9Z	110.6(17)
C7C	C8C	N1C	110.2(5)	C4G	N1G	C8G	108.9(4)
C7C	C8C	C9C	122.6(5)	C2G	C1G	Cl1G	117.9(5)
C8C	C9C	C10C	104.1(5)	C6G	C1G	Cl1G	118.1(5)
C8C	C9C	C14C	108.0(4)	C6G	C1G	C2G	124.0(5)
C14C	C9C	C10C	100.0(5)	C1G	C2G	C3G	119.1(5)
C11C	C10C	C9C	100.0(5)	C2G	C3G	C4G	118.5(6)
C10C	C11C	C12C	110.2(5)	N1G	C4G	C3G	130.0(5)
C13C	C11C	C10C	100.6(5)	N1G	C4G	C5G	107.6(5)
C13C	C11C	C12C	111.9(4)	C3G	C4G	C5G	122.4(5)
C7C	C12C	C11C	108.1(4)	C4G	C5G	C7G	106.5(4)
C7C	C12C	C15C	110.6(4)	C6G	C5G	C4G	118.2(5)
C15C	C12C	C11C	109.1(4)	C6G	C5G	C7G	135.3(5)
C16C	C12C	C7C	110.4(4)	C1G	C6G	C5G	117.7(5)
C16C	C12C	C11C	110.0(4)	C5G	C7G	C12G	131.7(5)
C16C	C12C	C15C	108.6(5)	C8G	C7G	C5G	106.7(5)
C14C	C13C	C11C	110.3(6)	C8G	C7G	C12G	121.6(5)
C13C	C14C	C9C	109.6(6)	N1G	C8G	C9G	125.7(5)
C4D	N1D	C8D	108.4(5)	C7G	C8G	N1G	110.2(5)
C2D	C1D	Cl1D	116.8(5)	C7G	C8G	C9G	123.9(5)
C6D	C1D	Cl1D	118.4(5)	C8G	C9G	C10G	103.6(5)
C6D	C1D	C2D	124.7(6)	C8G	C9G	C14G	107.4(4)
C3D	C2D	C1D	119.0(6)	C14G	C9G	C10G	100.2(5)
C2D	C3D	C4D	118.3(5)	C11G	C10G	C9G	100.1(4)
N1D	C4D	C3D	129.6(5)	C10G	C11G	C12G	110.9(5)
N1D	C4D	C5D	107.6(5)	C13G	C11G	C10G	100.2(5)
C3D	C4D	C5D	122.7(5)	C13G	C11G	C12G	111.6(4)
C4D	C5D	C7D	106.8(5)	C7G	C12G	C11G	108.7(4)
C6D	C5D	C4D	118.2(5)	C7G	C12G	C15G	111.0(4)
C6D	C5D	C7D	135.0(5)	C7G	C12G	C16G	109.7(4)
C1D	C6D	C5D	117.0(5)	C15G	C12G	C11G	110.4(4)
C5D	C7D	C12D	130.7(5)	C15G	C12G	C16G	108.4(4)
C8D	C7D	C5D	106.6(4)	C16G	C12G	C11G	108.6(4)
C8D	C7D	C12D	122.6(5)	C14G	C13G	C11G	109.7(5)
N1D	C8D	C9D	126.2(5)	C13G	C14G	C9G	109.9(5)
C7D	C8D	N1D	110.5(5)	C8H	N1H	C4H	108.9(4)
C7D	C8D	C9D	123.1(5)	C2H	C1H	Cl1H	117.5(4)

C8D	C9D	C10D	104.7(5)	C2H	C1H	C6H	123.4(5)
C8D	C9D	C14D	106.3(5)	C6H	C1H	Cl1H	119.1(4)
C14D	C9D	C10D	100.0(5)	C1H	C2H	C3H	120.0(5)
C11D	C10D	C9D	99.6(5)	C4H	C3H	C2H	117.4(5)
C10D	C11D	C12D	111.0(5)	N1H	C4H	C3H	129.1(5)
C13D	C11D	C10D	100.7(5)	N1H	C4H	C5H	107.8(4)
C13D	C11D	C12D	111.2(5)	C3H	C4H	C5H	123.2(5)
C7D	C12D	C11D	107.6(5)	C4H	C5H	C7H	106.3(4)
C7D	C12D	C15D	111.3(5)	C6H	C5H	C4H	118.4(5)
C7D	C12D	C16D	109.1(4)	C6H	C5H	C7H	135.3(4)
C15D	C12D	C11D	110.5(5)	C1H	C6H	C5H	117.6(4)
C16D	C12D	C11D	109.6(5)	C5H	C7H	C12H	131.1(4)
C16D	C12D	C15D	108.7(5)	C8H	C7H	C5H	106.4(4)
C14D	C13D	C11D	110.6(6)	C8H	C7H	C12H	122.5(5)
C13D	C14D	C9D	109.5(6)	N1H	C8H	C7H	110.7(5)
C4E	N1E	C8E	108.8(4)	N1H	C8H	C9H	126.6(5)
C2E	C1E	Cl1E	117.3(4)	C7H	C8H	C9H	122.7(5)
C6E	C1E	Cl1E	119.0(4)	C8H	C9H	C10H	105.0(4)
C6E	C1E	C2E	123.7(5)	C8H	C9H	C14H	107.2(5)
C3E	C2E	C1E	118.9(5)	C14H	C9H	C10H	100.6(4)
C2E	C3E	C4E	118.7(5)	C9H	C10H	C11H	99.3(4)
N1E	C4E	C3E	129.9(5)	C10H	C11H	C12H	110.4(4)
N1E	C4E	C5E	107.3(5)	C13H	C11H	C10H	100.0(5)
C3E	C4E	C5E	122.7(5)	C13H	C11H	C12H	111.9(4)
C4E	C5E	C7E	107.0(5)	C7H	C12H	C11H	108.0(4)
C6E	C5E	C4E	117.6(5)	C7H	C12H	C15H	111.3(4)
C6E	C5E	C7E	135.4(5)	C7H	C12H	C16H	110.1(4)
C1E	C6E	C5E	118.3(5)	C15H	C12H	C11H	109.8(4)
C5E	C7E	C12E	130.9(5)	C15H	C12H	C16H	109.2(4)
C8E	C7E	C5E	106.8(4)	C16H	C12H	C11H	108.4(4)
C8E	C7E	C12E	122.2(5)	C14H	C13H	C11H	111.4(5)
N1E	C8E	C9E	126.6(5)	C13H	C14H	C9H	109.1(5)

Table 6 Torsion Angles for JW315a.

A	B	C	D	Angle/°	A	B	C	D	Angle/°
Cl1A	C1A	C2A	C3A	-179.3(4)	C7E	C5E	C6E	C1E	-179.0(6)
Cl1A	C1A	C6A	C5A	179.6(4)	C7E	C8E	C9E	C10E	-34.8(7)
N1A	C4A	C5A	C6A	-179.7(4)	C7E	C8E	C9E	C14E	70.4(7)
N1A	C4A	C5A	C7A	0.6(5)	C8E	N1E	C4E	C3E	-178.2(6)
N1A	C8A	C9A	C10A	145.1(5)	C8E	N1E	C4E	C5E	1.0(6)
N1A	C8A	C9A	C14A	-112.3(6)	C8E	C7E	C12E	C11E	0.1(7)
C1A	C2A	C3A	C4A	-0.5(8)	C8E	C7E	C12E	C15E	-119.6(6)

C2A	C1A	C6A	C5A	0.3(8)	C8E	C7E	C12E	C16E	120.1(6)
C2A	C3A	C4A	N1A	179.7(5)	C8E	C9E	C10E	C11E	69.0(6)
C2A	C3A	C4A	C5A	0.8(7)	C8E	C9E	C14E	C13E	-82.1(6)
C3A	C4A	C5A	C6A	-0.5(7)	C9E	C10E	C11E	C12E	-76.4(6)
C3A	C4A	C5A	C7A	179.7(5)	C9E	C10E	C11E	C13E	42.1(5)
C4A	N1A	C8A	C7A	-0.7(6)	C10E	C9E	C14E	C13E	26.7(6)
C4A	N1A	C8A	C9A	178.4(5)	C10E	C11E	C12E	C7E	40.0(6)
C4A	C5A	C6A	C1A	-0.1(7)	C10E	C11E	C12E	C15E	159.8(5)
C4A	C5A	C7A	C8A	-1.0(5)	C10E	C11E	C12E	C16E	-81.1(6)
C4A	C5A	C7A	C12A	-179.5(5)	C10E	C11E	C13E	C14E	-27.5(6)
C5A	C7A	C8A	N1A	1.0(6)	C11E	C13E	C14E	C9E	0.6(7)
C5A	C7A	C8A	C9A	-178.1(5)	C12E	C7E	C8E	N1E	177.8(5)
C5A	C7A	C12A	C11A	176.4(5)	C12E	C7E	C8E	C9E	-2.2(8)
C5A	C7A	C12A	C15A	55.8(8)	C12E	C11E	C13E	C14E	90.0(6)
C5A	C7A	C12A	C16A	-64.0(8)	C13E	C11E	C12E	C7E	-70.8(6)
C6A	C1A	C2A	C3A	-0.1(8)	C13E	C11E	C12E	C15E	49.0(7)
C6A	C5A	C7A	C8A	179.3(5)	C13E	C11E	C12E	C16E	168.1(5)
C6A	C5A	C7A	C12A	0.8(10)	C14E	C9E	C10E	C11E	-41.4(6)
C7A	C5A	C6A	C1A	179.6(5)	Cl1F	C1F	C2F	C3F	177.7(11)
C7A	C8A	C9A	C10A	-35.9(7)	Cl1F	C1F	C6F	C5F	-176.9(18)
C7A	C8A	C9A	C14A	66.7(7)	N1F	C4F	C5F	C6F	179(2)
C8A	N1A	C4A	C3A	-179.0(5)	N1F	C4F	C5F	C7F	-2(3)
C8A	N1A	C4A	C5A	0.1(5)	N1F	C8F	C9F	C10F	145(2)
C8A	C7A	C12A	C11A	-2.0(7)	N1F	C8F	C9F	C14F	-110(2)
C8A	C7A	C12A	C15A	-122.5(6)	C1F	C2F	C3F	C4F	-2.5(19)
C8A	C7A	C12A	C16A	117.7(6)	C2F	C1F	C6F	C5F	0(3)
C8A	C9A	C10A	C11A	69.4(5)	C2F	C3F	C4F	N1F	-178.6(17)
C8A	C9A	C14A	C13A	-79.4(8)	C2F	C3F	C4F	C5F	4(2)
C9A	C10A	C11A	C12A	-77.2(6)	C3F	C4F	C5F	C6F	-3(4)
C9A	C10A	C11A	C13A	40.5(5)	C3F	C4F	C5F	C7F	175.8(18)
C10A	C9A	C14A	C13A	28.0(8)	C4F	N1F	C8F	C7F	6(3)
C10A	C11A	C12A	C7A	42.7(7)	C4F	N1F	C8F	C9F	-179(2)
C10A	C11A	C12A	C15A	162.6(6)	C4F	C5F	C6F	C1F	1(4)
C10A	C11A	C12A	C16A	-78.2(7)	C4F	C5F	C7F	C8F	6(3)
C10A	C11A	C13A	C14A	-25.8(8)	C4F	C5F	C7F	C12F	175(2)
C11A	C13A	C14A	C9A	-2.5(9)	C5F	C7F	C8F	N1F	-7(3)
C12A	C7A	C8A	N1A	179.7(5)	C5F	C7F	C8F	C9F	178(2)
C12A	C7A	C8A	C9A	0.6(8)	C5F	C7F	C12F	C11F	-174(3)
C12A	C11A	C13A	C14A	92.2(7)	C5F	C7F	C12F	C15F	66(3)
C13A	C11A	C12A	C7A	-69.7(6)	C5F	C7F	C12F	C16F	-55(3)
C13A	C11A	C12A	C15A	50.2(7)	C6F	C1F	C2F	C3F	1(2)
C13A	C11A	C12A	C16A	169.3(6)	C6F	C5F	C7F	C8F	-175(3)

C14A	C9A	C10A	C11A	-40.2(5)	C6F	C5F	C7F	C12F	-6(6)
Cl1B	C1B	C2B	C3B	-178.9(4)	C7F	C5F	C6F	C1F	-178(3)
Cl1B	C1B	C6B	C5B	177.3(4)	C7F	C8F	C9F	C10F	-41(3)
N1B	C4B	C5B	C6B	-179.3(5)	C7F	C8F	C9F	C14F	64(3)
N1B	C4B	C5B	C7B	-0.4(6)	C8F	N1F	C4F	C3F	-179.9(18)
N1B	C8B	C9B	C10B	146.8(5)	C8F	N1F	C4F	C5F	-2(3)
N1B	C8B	C9B	C14B	-106.7(6)	C8F	C7F	C12F	C11F	-6(3)
C1B	C2B	C3B	C4B	1.5(8)	C8F	C7F	C12F	C15F	-126(2)
C2B	C1B	C6B	C5B	-1.2(8)	C8F	C7F	C12F	C16F	113(3)
C2B	C3B	C4B	N1B	177.3(5)	C8F	C9F	C10F	C11F	69.4(14)
C2B	C3B	C4B	C5B	-1.0(8)	C8F	C9F	C14F	C13F	-82.0(18)
C3B	C4B	C5B	C6B	-0.6(8)	C9F	C10F	C11F	C12F	-75.2(14)
C3B	C4B	C5B	C7B	178.3(5)	C9F	C10F	C11F	C13F	42.8(14)
C4B	N1B	C8B	C7B	-0.3(6)	C10F	C9F	C14F	C13F	26.5(18)
C4B	N1B	C8B	C9B	177.3(5)	C10F	C11F	C12F	C7F	42.0(16)
C4B	C5B	C6B	C1B	1.7(7)	C10F	C11F	C12F	C15F	162.3(14)
C4B	C5B	C7B	C8B	0.2(6)	C10F	C11F	C12F	C16F	-77.8(16)
C4B	C5B	C7B	C12B	-175.1(5)	C10F	C11F	C13F	C14F	-29(2)
C5B	C7B	C8B	N1B	0.0(6)	C11F	C13F	C14F	C9F	1(2)
C5B	C7B	C8B	C9B	-177.7(5)	C12F	C7F	C8F	N1F	-178(2)
C5B	C7B	C12B	C11B	175.4(5)	C12F	C7F	C8F	C9F	7(4)
C5B	C7B	C12B	C15B	54.5(7)	C12F	C11F	C13F	C14F	89(2)
C5B	C7B	C12B	C16B	-65.9(7)	C13F	C11F	C12F	C7F	-68.1(16)
C6B	C1B	C2B	C3B	-0.4(8)	C13F	C11F	C12F	C15F	52.3(17)
C6B	C5B	C7B	C8B	178.8(6)	C13F	C11F	C12F	C16F	172.2(15)
C6B	C5B	C7B	C12B	3.5(10)	C14F	C9F	C10F	C11F	-42.2(13)
C7B	C5B	C6B	C1B	-176.8(5)	Cl1Z	C1Z	C2Z	C3Z	178.9(15)
C7B	C8B	C9B	C10B	-35.8(7)	Cl1Z	C1Z	C6Z	C5Z	-179(2)
C7B	C8B	C9B	C14B	70.6(7)	N1Z	C4Z	C5Z	C6Z	179(3)
C8B	N1B	C4B	C3B	-178.1(6)	N1Z	C4Z	C5Z	C7Z	5(4)
C8B	N1B	C4B	C5B	0.4(6)	N1Z	C8Z	C9Z	C10Z	142(4)
C8B	C7B	C12B	C11B	0.7(7)	N1Z	C8Z	C9Z	C14Z	-114(4)
C8B	C7B	C12B	C15B	-120.1(6)	C1Z	C2Z	C3Z	C4Z	-2(3)
C8B	C7B	C12B	C16B	119.5(6)	C2Z	C1Z	C6Z	C5Z	0(4)
C8B	C9B	C10B	C11B	70.2(5)	C2Z	C3Z	C4Z	N1Z	-179(2)
C8B	C9B	C14B	C13B	-80.9(6)	C2Z	C3Z	C4Z	C5Z	3(4)
C9B	C10B	C11B	C12B	-76.8(6)	C3Z	C4Z	C5Z	C6Z	-3(6)
C9B	C10B	C11B	C13B	41.0(5)	C3Z	C4Z	C5Z	C7Z	-176(3)
C10B	C9B	C14B	C13B	28.1(6)	C4Z	N1Z	C8Z	C7Z	-5(4)
C10B	C11B	C12B	C7B	39.4(6)	C4Z	N1Z	C8Z	C9Z	180(3)
C10B	C11B	C12B	C15B	159.5(5)	C4Z	C5Z	C6Z	C1Z	1(5)
C10B	C11B	C12B	C16B	-81.0(6)	C4Z	C5Z	C7Z	C8Z	-8(5)

C10B	C11B	C13B	C14B	-25.9(6)	C4Z	C5Z	C7Z	C12Z	-179(3)
C11B	C13B	C14B	C9B	-1.3(7)	C5Z	C7Z	C8Z	N1Z	8(5)
C12B	C7B	C8B	N1B	175.7(5)	C5Z	C7Z	C8Z	C9Z	-176(4)
C12B	C7B	C8B	C9B	-2.0(8)	C5Z	C7Z	C12Z	C11Z	171(4)
C12B	C11B	C13B	C14B	91.0(6)	C5Z	C7Z	C12Z	C15Z	50(5)
C13B	C11B	C12B	C7B	-70.7(6)	C5Z	C7Z	C12Z	C16Z	-68(5)
C13B	C11B	C12B	C15B	49.4(6)	C6Z	C1Z	C2Z	C3Z	0(3)
C13B	C11B	C12B	C16B	168.9(5)	C6Z	C5Z	C7Z	C8Z	180(5)
C14B	C9B	C10B	C11B	-42.1(5)	C6Z	C5Z	C7Z	C12Z	9(8)
Cl1C	C1C	C2C	C3C	-179.1(4)	C7Z	C5Z	C6Z	C1Z	172(4)
Cl1C	C1C	C6C	C5C	178.9(4)	C7Z	C8Z	C9Z	C10Z	-33(4)
N1C	C4C	C5C	C6C	-179.4(4)	C7Z	C8Z	C9Z	C14Z	71(4)
N1C	C4C	C5C	C7C	0.3(6)	C8Z	N1Z	C4Z	C3Z	-179(3)
N1C	C8C	C9C	C10C	144.8(6)	C8Z	N1Z	C4Z	C5Z	0(4)
N1C	C8C	C9C	C14C	-109.5(6)	C8Z	C7Z	C12Z	C11Z	2(5)
C1C	C2C	C3C	C4C	0.4(8)	C8Z	C7Z	C12Z	C15Z	-119(4)
C2C	C1C	C6C	C5C	-2.1(8)	C8Z	C7Z	C12Z	C16Z	123(4)
C2C	C3C	C4C	N1C	179.6(6)	C8Z	C9Z	C10Z	C11Z	69(2)
C2C	C3C	C4C	C5C	-2.4(8)	C8Z	C9Z	C14Z	C13Z	-81(3)
C3C	C4C	C5C	C6C	2.2(8)	C9Z	C10Z	C11Z	C12Z	-76.0(19)
C3C	C4C	C5C	C7C	-178.1(5)	C9Z	C10Z	C11Z	C13Z	42(2)
C4C	N1C	C8C	C7C	0.8(6)	C10Z	C9Z	C14Z	C13Z	28(3)
C4C	N1C	C8C	C9C	177.6(5)	C10Z	C11Z	C12Z	C7Z	39(3)
C4C	C5C	C6C	C1C	0.1(7)	C10Z	C11Z	C12Z	C15Z	159(2)
C4C	C5C	C7C	C8C	0.1(6)	C10Z	C11Z	C12Z	C16Z	-80(3)
C4C	C5C	C7C	C12C	177.9(5)	C10Z	C11Z	C13Z	C14Z	-26(4)
C5C	C7C	C8C	N1C	-0.6(6)	C11Z	C13Z	C14Z	C9Z	-1(4)
C5C	C7C	C8C	C9C	-177.6(5)	C12Z	C7Z	C8Z	N1Z	179(3)
C5C	C7C	C12C	C11C	177.7(5)	C12Z	C7Z	C8Z	C9Z	-5(6)
C5C	C7C	C12C	C15C	58.3(7)	C12Z	C11Z	C13Z	C14Z	91(3)
C5C	C7C	C12C	C16C	-62.0(7)	C13Z	C11Z	C12Z	C7Z	-71(3)
C6C	C1C	C2C	C3C	1.9(9)	C13Z	C11Z	C12Z	C15Z	49(2)
C6C	C5C	C7C	C8C	179.8(5)	C13Z	C11Z	C12Z	C16Z	170(2)
C6C	C5C	C7C	C12C	-2.4(9)	C14Z	C9Z	C10Z	C11Z	-41.8(18)
C7C	C5C	C6C	C1C	-179.6(5)	Cl1G	C1G	C2G	C3G	-179.6(4)
C7C	C8C	C9C	C10C	-38.7(7)	Cl1G	C1G	C6G	C5G	179.1(4)
C7C	C8C	C9C	C14C	67.0(7)	N1G	C4G	C5G	C6G	179.5(4)
C8C	N1C	C4C	C3C	177.5(5)	N1G	C4G	C5G	C7G	0.2(5)
C8C	N1C	C4C	C5C	-0.7(6)	N1G	C8G	C9G	C10G	146.2(5)
C8C	C7C	C12C	C11C	-4.9(7)	N1G	C8G	C9G	C14G	-108.3(6)
C8C	C7C	C12C	C15C	-124.3(5)	C1G	C2G	C3G	C4G	0.5(8)
C8C	C7C	C12C	C16C	115.5(6)	C2G	C1G	C6G	C5G	-0.5(8)

C8C	C9C	C10C	C11C	70.7(6)	C2G	C3G	C4G	N1G	-179.9(5)
C8C	C9C	C14C	C13C	-82.1(6)	C2G	C3G	C4G	C5G	-0.7(8)
C9C	C10C	C11C	C12C	-77.2(6)	C3G	C4G	C5G	C6G	0.2(7)
C9C	C10C	C11C	C13C	41.0(6)	C3G	C4G	C5G	C7G	-179.1(5)
C10C	C9C	C14C	C13C	26.4(6)	C4G	N1G	C8G	C7G	1.0(6)
C10C	C11C	C12C	C7C	42.4(6)	C4G	N1G	C8G	C9G	177.5(5)
C10C	C11C	C12C	C15C	162.7(5)	C4G	C5G	C6G	C1G	0.4(7)
C10C	C11C	C12C	C16C	-78.2(6)	C4G	C5G	C7G	C8G	0.4(5)
C10C	C11C	C13C	C14C	-26.9(6)	C4G	C5G	C7G	C12G	-179.5(5)
C11C	C13C	C14C	C9C	0.0(7)	C5G	C7G	C8G	N1G	-0.9(6)
C12C	C7C	C8C	N1C	-178.6(5)	C5G	C7G	C8G	C9G	-177.4(4)
C12C	C7C	C8C	C9C	4.4(8)	C5G	C7G	C12G	C11G	176.9(5)
C12C	C11C	C13C	C14C	90.1(6)	C5G	C7G	C12G	C15G	55.4(7)
C13C	C11C	C12C	C7C	-68.7(6)	C5G	C7G	C12G	C16G	-64.4(7)
C13C	C11C	C12C	C15C	51.7(6)	C6G	C1G	C2G	C3G	0.0(9)
C13C	C11C	C12C	C16C	170.7(5)	C6G	C5G	C7G	C8G	-178.7(5)
C14C	C9C	C10C	C11C	-40.9(6)	C6G	C5G	C7G	C12G	1.4(9)
Cl1D	C1D	C2D	C3D	-178.8(5)	C7G	C5G	C6G	C1G	179.5(5)
Cl1D	C1D	C6D	C5D	178.7(4)	C7G	C8G	C9G	C10G	-37.8(6)
N1D	C4D	C5D	C6D	-179.1(5)	C7G	C8G	C9G	C14G	67.7(6)
N1D	C4D	C5D	C7D	1.4(6)	C8G	N1G	C4G	C3G	178.5(5)
N1D	C8D	C9D	C10D	146.0(6)	C8G	N1G	C4G	C5G	-0.8(5)
N1D	C8D	C9D	C14D	-108.7(6)	C8G	C7G	C12G	C11G	-2.9(6)
C1D	C2D	C3D	C4D	1.2(9)	C8G	C7G	C12G	C15G	-124.5(5)
C2D	C1D	C6D	C5D	0.8(9)	C8G	C7G	C12G	C16G	115.7(5)
C2D	C3D	C4D	N1D	179.1(6)	C8G	C9G	C10G	C11G	69.7(5)
C2D	C3D	C4D	C5D	-1.6(9)	C8G	C9G	C14G	C13G	-81.4(5)
C3D	C4D	C5D	C6D	1.5(8)	C9G	C10G	C11G	C12G	-76.6(5)
C3D	C4D	C5D	C7D	-178.0(5)	C9G	C10G	C11G	C13G	41.4(5)
C4D	N1D	C8D	C7D	1.0(6)	C10G	C9G	C14G	C13G	26.5(5)
C4D	N1D	C8D	C9D	177.1(5)	C10G	C11G	C12G	C7G	41.0(6)
C4D	C5D	C6D	C1D	-1.0(8)	C10G	C11G	C12G	C15G	163.0(4)
C4D	C5D	C7D	C8D	-0.8(6)	C10G	C11G	C12G	C16G	-78.3(5)
C4D	C5D	C7D	C12D	177.1(5)	C10G	C11G	C13G	C14G	-27.1(6)
C5D	C7D	C8D	N1D	-0.1(6)	C11G	C13G	C14G	C9G	0.3(6)
C5D	C7D	C8D	C9D	-176.4(5)	C12G	C7G	C8G	N1G	179.0(4)
C5D	C7D	C12D	C11D	175.3(5)	C12G	C7G	C8G	C9G	2.6(8)
C5D	C7D	C12D	C15D	54.1(7)	C12G	C11G	C13G	C14G	90.4(5)
C5D	C7D	C12D	C16D	-65.9(7)	C13G	C11G	C12G	C7G	-69.9(5)
C6D	C1D	C2D	C3D	-0.8(10)	C13G	C11G	C12G	C15G	52.1(6)
C6D	C5D	C7D	C8D	179.8(6)	C13G	C11G	C12G	C16G	170.8(4)
C6D	C5D	C7D	C12D	-2.3(10)	C14G	C9G	C10G	C11G	-41.3(5)

C7D	C5D	C6D	C1D	178.3(6)	Cl1H	C1H	C2H	C3H	178.8(5)
C7D	C8D	C9D	C10D	-38.3(8)	Cl1H	C1H	C6H	C5H	-179.3(4)
C7D	C8D	C9D	C14D	67.0(7)	N1H	C4H	C5H	C6H	178.1(5)
C8D	N1D	C4D	C3D	177.9(6)	N1H	C4H	C5H	C7H	0.1(6)
C8D	N1D	C4D	C5D	-1.5(6)	N1H	C8H	C9H	C10H	144.5(5)
C8D	C7D	C12D	C11D	-7.1(7)	N1H	C8H	C9H	C14H	-109.1(6)
C8D	C7D	C12D	C15D	-128.4(6)	C1H	C2H	C3H	C4H	-0.1(9)
C8D	C7D	C12D	C16D	111.7(6)	C2H	C1H	C6H	C5H	0.3(8)
C8D	C9D	C10D	C11D	68.8(6)	C2H	C3H	C4H	N1H	-178.6(6)
C8D	C9D	C14D	C13D	-80.9(6)	C2H	C3H	C4H	C5H	1.4(9)
C9D	C10D	C11D	C12D	-77.3(6)	C3H	C4H	C5H	C6H	-1.9(8)
C9D	C10D	C11D	C13D	40.5(6)	C3H	C4H	C5H	C7H	-179.9(5)
C10D	C9D	C14D	C13D	27.7(6)	C4H	N1H	C8H	C7H	1.2(6)
C10D	C11D	C12D	C7D	44.3(6)	C4H	N1H	C8H	C9H	-179.3(5)
C10D	C11D	C12D	C15D	166.0(5)	C4H	C5H	C6H	C1H	1.0(7)
C10D	C11D	C12D	C16D	-74.3(6)	C4H	C5H	C7H	C8H	0.6(6)
C10D	C11D	C13D	C14D	-25.6(6)	C4H	C5H	C7H	C12H	-179.7(5)
C11D	C13D	C14D	C9D	-1.4(7)	C5H	C7H	C8H	N1H	-1.1(6)
C12D	C7D	C8D	N1D	-178.2(5)	C5H	C7H	C8H	C9H	179.3(5)
C12D	C7D	C8D	C9D	5.5(8)	C5H	C7H	C12H	C11H	178.6(5)
C12D	C11D	C13D	C14D	92.1(6)	C5H	C7H	C12H	C15H	58.0(7)
C13D	C11D	C12D	C7D	-67.1(6)	C5H	C7H	C12H	C16H	-63.2(7)
C13D	C11D	C12D	C15D	54.7(6)	C6H	C1H	C2H	C3H	-0.7(9)
C13D	C11D	C12D	C16D	174.4(5)	C6H	C5H	C7H	C8H	-176.9(6)
C14D	C9D	C10D	C11D	-41.1(6)	C6H	C5H	C7H	C12H	2.7(10)
Cl1E	C1E	C2E	C3E	179.4(5)	C7H	C5H	C6H	C1H	178.3(5)
Cl1E	C1E	C6E	C5E	-179.4(4)	C7H	C8H	C9H	C10H	-36.0(7)
N1E	C4E	C5E	C6E	179.7(5)	C7H	C8H	C9H	C14H	70.4(7)
N1E	C4E	C5E	C7E	-0.5(6)	C8H	N1H	C4H	C3H	179.2(6)
N1E	C8E	C9E	C10E	145.3(6)	C8H	N1H	C4H	C5H	-0.8(6)
N1E	C8E	C9E	C14E	-109.6(6)	C8H	C7H	C12H	C11H	-1.9(7)
C1E	C2E	C3E	C4E	-0.8(10)	C8H	C7H	C12H	C15H	-122.4(5)
C2E	C1E	C6E	C5E	-0.5(9)	C8H	C7H	C12H	C16H	116.4(5)
C2E	C3E	C4E	N1E	-179.9(6)	C8H	C9H	C10H	C11H	70.0(5)
C2E	C3E	C4E	C5E	1.1(9)	C8H	C9H	C14H	C13H	-82.0(6)
C3E	C4E	C5E	C6E	-1.0(8)	C9H	C10H	C11H	C12H	-77.4(5)
C3E	C4E	C5E	C7E	178.7(5)	C9H	C10H	C11H	C13H	40.6(5)
C4E	N1E	C8E	C7E	-1.1(6)	C10H	C9H	C14H	C13H	27.5(6)
C4E	N1E	C8E	C9E	178.9(5)	C10H	C11H	C12H	C7H	41.8(6)
C4E	C5E	C6E	C1E	0.7(8)	C10H	C11H	C12H	C15H	163.2(4)
C4E	C5E	C7E	C8E	-0.1(6)	C10H	C11H	C12H	C16H	-77.6(5)
C4E	C5E	C7E	C12E	-176.8(5)	C10H	C11H	C13H	C14H	-26.2(6)

C5E	C7E	C8E	N1E	0.7(6)	C11H	C13H	C14H	C9H	-0.8(6)
C5E	C7E	C8E	C9E	-179.2(5)	C12H	C7H	C8H	N1H	179.2(4)
C5E	C7E	C12E	C11E	176.3(5)	C12H	C7H	C8H	C9H	-0.3(8)
C5E	C7E	C12E	C15E	56.6(8)	C12H	C11H	C13H	C14H	90.7(6)
C5E	C7E	C12E	C16E	-63.6(7)	C13H	C11H	C12H	C7H	-68.6(6)
C6E	C1E	C2E	C3E	0.5(10)	C13H	C11H	C12H	C15H	52.8(6)
C6E	C5E	C7E	C8E	179.5(6)	C13H	C11H	C12H	C16H	172.1(5)
C6E	C5E	C7E	C12E	2.9(10)	C14H	C9H	C10H	C11H	-41.3(5)

Table 7 Hydrogen Atom Coordinates (Å×10^4) and Isotropic Displacement Parameters (Å^2×10^3) for JW315a.

Atom	*x*	*y*	*z*	U(eq)
H1A	-1894	5487	1554	44
H2A	910	7253	1614	44
H3A	-830	6746	1442	43
H6A	2250	5416	2288	39
H9A	-2414	4122	1701	56
H10A	-1417	2976	1780	75
H10B	-626	3479	1463	75
H11A	200	2946	2469	56
H13A	-679	3474	3255	80
H14A	-2250	4093	2816	81
H15A	1926	4505	2970	102
H15B	1670	3726	3176	102
H15C	835	4353	3240	102
H16A	1176	3615	1588	92
H16B	1933	3322	2171	92
H16C	2093	4109	1951	92
H1B	6797	4133	-21	45
H2B	7611	6606	252	46
H3B	7642	5465	-134	45
H6B	5761	5952	1467	39
H9B	5886	2963	381	51
H10C	6067	3217	1504	52
H10D	5227	2587	1277	52
H11B	4078	3380	1685	48
H13B	2900	3512	703	56
H14B	3934	3213	-73	57
H15D	4055	5330	1432	73
H15E	3271	4709	1582	73
H15F	3483	4824	914	73
H16D	5886	4050	2196	81

H16E	4804	4406	2361	81
H16F	5728	4885	2145	81
H1C	9210	4385	9180	51
H2C	5830	3046	8917	58
H3C	7687	3285	9187	57
H6C	5279	5005	8230	39
H9C	10248	5574	8903	59
H10E	9904	6505	8167	61
H10F	9117	5876	7877	61
H11C	8188	7045	8054	49
H13C	8365	7245	9137	57
H14C	9613	6394	9650	62
H15G	5662	6225	8428	62
H15H	6203	6976	8344	62
H15I	6564	6537	8942	62
H16G	7417	5843	7381	70
H16H	6608	6498	7391	70
H16I	6206	5720	7520	70
H1D	6599	3565	5041	49
H2D	7753	1148	5302	58
H3D	7576	2285	4903	53
H6D	5675	1615	6460	47
H9D	5240	4597	5301	57
H10G	3570	4683	5737	64
H10H	3559	3877	5505	64
H11D	3540	4055	6627	55
H13D	5320	4554	7083	58
H14D	6297	4934	6308	58
H15J	5154	2461	7110	71
H15K	4469	3075	7360	71
H15L	5669	3232	7219	71
H16J	3228	2809	5830	70
H16K	2925	2841	6487	70
H16L	3686	2222	6309	70
H1E	9292	6728	4239	44
H2E	10274	9180	4501	55
H3E	10191	8034	4109	49
H6E	8223	8636	5654	40
H9E	8292	5596	4636	56
H10I	8419	5883	5754	59
H10J	7559	5266	5524	59
H11E	6430	6098	5900	54

H13E	5287	6203	4909	61
H14E	6363	5891	4155	61
H15M	6452	8029	5602	73
H15N	5654	7416	5754	73
H15O	5900	7507	5090	73
H16M	8253	6767	6421	81
H16N	7164	7140	6559	81
H16O	8107	7601	6342	81
H1F	473	7789	5691	48
H2F	3112	9690	5757	59
H3F	1417	9101	5555	53
H6F	4504	7980	6645	40
H9F	154	6417	5849	45
H10K	1270	5358	6072	58
H10L	2073	5892	5795	58
H11F	2758	5449	6855	55
H13F	1516	5887	7509	55
H14F	-33	6460	6942	50
H15P	4178	7055	7439	101
H15Q	3795	6301	7643	101
H15R	2971	6955	7591	101
H16P	3855	6180	6040	83
H16Q	4542	5959	6667	83
H16R	4593	6751	6434	83
H1Z	840	8098	5720	43
H2Z	4048	9578	5990	57
H3Z	2215	9255	5707	51
H6Z	4790	7669	6760	49
H9Z	45	6779	5845	60
H10M	789	5598	5981	62
H10N	1735	6027	5721	62
H11Z	2296	5433	6739	68
H13Z	1305	6022	7463	75
H14Z	-65	6771	6942	64
H15S	4170	6911	7351	78
H15T	3798	6148	7540	78
H15U	3001	6808	7552	78
H16S	3627	6044	5946	80
H16T	4216	5697	6553	80
H16U	4533	6478	6378	80
H1G	-1941	2108	6547	41
H2G	1037	453	6656	54

H3G	-753	901	6448	49
H6G	2151	2319	7391	43
H9G	-2674	3354	6875	44
H10O	-2050	4264	7604	50
H10P	-1388	3572	7870	50
H11G	-227	4639	7660	44
H13G	-497	4843	6567	46
H14G	-1971	4099	6097	44
H15V	2029	3601	7214	57
H15W	1649	4396	7289	57
H15X	1166	3960	6706	57
H16V	330	3397	8317	68
H16W	1245	3983	8285	68
H16X	1486	3177	8147	68
H1H	795	5930	10783	41
H2H	-405	3545	10377	47
H3H	-303	4680	10800	46
H6H	1912	4045	9367	36
H9H	2291	6931	10672	44
H10Q	3936	6183	10467	52
H10R	4034	7012	10320	52
H11H	4121	6501	9382	44
H13H	2440	7125	8932	52
H14H	1385	7424	9686	54
H15Y	2356	5038	8704	56
H	3111	5665	8533	56
HA	1916	5831	8677	56
H16Y	4336	5200	10018	56
HB	4576	5243	9349	56
HC	3786	4644	9533	56

Table 8 Atomic Occupancy for JW315a.

**Atom**	***Occupancy***	**Atom**	***Occupancy***	**Atom**	***Occupancy***
Cl1F	0.6	N1F	0.6	H1F	0.6
C1F	0.6	C2F	0.6	H2F	0.6
C3F	0.6	H3F	0.6	C4F	0.6
C5F	0.6	C6F	0.6	H6F	0.6
C7F	0.6	C8F	0.6	C9F	0.6
H9F	0.6	C10F	0.6	H10K	0.6
H10L	0.6	C11F	0.6	H11F	0.6
C12F	0.6	C13F	0.6	H13F	0.6
C14F	0.6	H14F	0.6	C15F	0.6

H15P 0.6	H15Q 0.6	H15R 0.6
C16F 0.6	H16P 0.6	H16Q 0.6
H16R 0.6	Cl1Z 0.4	N1Z 0.4
H1Z 0.4	C1Z 0.4	C2Z 0.4
H2Z 0.4	C3Z 0.4	H3Z 0.4
C4Z 0.4	C5Z 0.4	C6Z 0.4
H6Z 0.4	C7Z 0.4	C8Z 0.4
C9Z 0.4	H9Z 0.4	C10Z 0.4
H10M 0.4	H10N 0.4	C11Z 0.4
H11Z 0.4	C12Z 0.4	C13Z 0.4
H13Z 0.4	C14Z 0.4	H14Z 0.4
C15Z 0.4	H15S 0.4	H15T 0.4
H15U 0.4	C16Z 0.4	H16S 0.4
H16T 0.4	H16U 0.4	

Experimental

Single crystals of $C_{16}H_{16}ClN$ [JW315a] were used as received. A suitable crystal was selected and mounted on a nylon loop using a small amount of paratone oil on a 'Bruker APEX-II CCD' diffractometer. The crystal was kept at 173.0 K during data collection. Using Olex2 [1], the structure was solved with the ShelXS [2] structure solution program using Direct Methods and refined with the ShelXL [3] refinement package using Least Squares minimization.

1. Dolomanov, O.V., Bourhis, L.J., Gildea, R.J, Howard, J.A.K. & Puschmann, H. (2009), J. Appl. Cryst. 42, 339-341.

2. Sheldrick, G.M. (2008). Acta Cryst. A64, 112-122.

3. Sheldrick, G.M. (2008). Acta Cryst. A64, 112-122.

Crystal structure determination of [JW315a]

Crystal Data for $C_{16}H_{16}ClN$ (M =257.75 g/mol): monoclinic, space group $P2_1$ (no. 4), a = 12.4101(3) Å, b = 18.9939(3) Å, c = 22.7815(4) Å, β = 99.0520(10)°, V = 5303.09(18) Å^3, Z = 16, T = 173.0 K, μ(CuKα) = 2.372 mm^{-1}, $Dcalc$ = 1.291 g/cm^3, 66305 reflections measured (3.928 ≤ 2Θ ≤ 144.904°), 20467 unique (R_{int} = 0.0891, R_{sigma} = 0.0731) which were used in all calculations. The final R_1 was 0.0522 (I > 2σ(I)) and wR_2 was 0.1280 (all data).

Refinement model description

Number of restraints - 522, number of constraints - unknown.

Details:

1. Fixed Uiso

At 1.2 times of:

All C(H) groups, All C(H,H) groups, All N(H) groups

At 1.5 times of:

All C(H,H,H) groups

2. Rigid bond restraints

Cl1F, N1F, C1F, C2F, C3F, C4F, C5F, C6F, C7F, C8F, C9F, C10F, C11F, C12F, C13F, C14F, C15F, C16F
with sigma for 1-2 distances of 0.01 and sigma for 1-3 distances of 0.01
Cl1Z, N1Z, C1Z, C2Z, C3Z, C4Z, C5Z, C6Z, C7Z, C8Z, C9Z, C10Z, C11Z, C12Z, C13Z, C14Z, C15Z, C16Z
with sigma for 1-2 distances of 0.01 and sigma for 1-3 distances of 0.01
3. Uiso/Uaniso restraints and constraints
Cl1F ≈ N1F ≈ C1F ≈ C2F ≈ C3F ≈ C4F ≈ C5F ≈ C6F
≈ C7F ≈ C8F ≈ C9F ≈ C10F ≈ C11F ≈ C12F ≈ C13F ≈
C14F ≈ C15F ≈ C16F: within 1.7A with sigma of 0.04 and sigma for terminal atoms of 0.08
Cl1Z ≈ N1Z ≈ C1Z ≈ C2Z ≈ C3Z ≈ C4Z ≈ C5Z ≈ C6Z
≈ C7Z ≈ C8Z ≈ C9Z ≈ C10Z ≈ C11Z ≈ C12Z ≈ C13Z ≈
C14Z ≈ C15Z ≈ C16Z: within 1.7A with sigma of 0.04 and sigma for terminal atoms of 0.08
Uanis(C2Z) ≈ Ueq: with sigma of 0.01 and sigma for terminal atoms of 0.02
4. Same fragment restrains
{Cl1A, N1A, C1A, C2A, C3A, C4A, C5A, C6A, C7A, C8A, C9A, C10A, C11A, C12A, C13A, C14A, C15A, C16A} sigma for 1-2: 0.02, 1-3: 0.04
as
{Cl1F, N1F, C1F, C2F, C3F, C4F, C5F, C6F, C7F, C8F, C9F, C10F, C11F, C12F, C13F, C14F, C15F, C16F}
{Cl1A, N1A, C1A, C2A, C3A, C4A, C5A, C6A, C7A, C8A, C9A, C10A, C11A, C12A, C13A, C14A, C15A, C16A} sigma for 1-2: 0.02, 1-3: 0.04
as
{Cl1Z, N1Z, C1Z, C2Z, C3Z, C4Z, C5Z, C6Z, C7Z, C8Z, C9Z, C10Z, C11Z, C12Z, C13Z, C14Z, C15Z, C16Z}
5. Others
Fixed Sof: Cl1F(0.6) N1F(0.6) H1F(0.6) C1F(0.6) C2F(0.6) H2F(0.6) C3F(0.6) H3F(0.6) C4F(0.6) C5F(0.6) C6F(0.6) H6F(0.6) C7F(0.6) C8F(0.6) C9F(0.6) H9F(0.6) C10F(0.6) H10K(0.6) H10L(0.6) C11F(0.6) H11F(0.6) C12F(0.6) C13F(0.6) H13F(0.6) C14F(0.6) H14F(0.6) C15F(0.6) H15P(0.6) H15Q(0.6) H15R(0.6) C16F(0.6) H16P(0.6) H16Q(0.6) H16R(0.6) Cl1Z(0.4) N1Z(0.4) H1Z(0.4) C1Z(0.4) C2Z(0.4) H2Z(0.4) C3Z(0.4) H3Z(0.4) C4Z(0.4) C5Z(0.4) C6Z(0.4) H6Z(0.4) C7Z(0.4) C8Z(0.4) C9Z(0.4) H9Z(0.4) C10Z(0.4) H10M(0.4) H10N(0.4) C11Z(0.4) H11Z(0.4) C12Z(0.4) C13Z(0.4) H13Z(0.4) C14Z(0.4) H14Z(0.4) C15Z(0.4) H15S(0.4) H15T(0.4) H15U(0.4) C16Z(0.4) H16S(0.4) H16T(0.4) H16U(0.4)
6.a Ternary CH refined with riding coordinates:
C9A(H9A), C11A(H11A), C9B(H9B), C11B(H11B), C9C(H9C), C11C(H11C), C9D(H9D),
C11D(H11D), C9E(H9E), C11E(H11E), C9F(H9F), C11F(H11F), C9Z(H9Z), C11Z(H11Z),
C9G(H9G), C11G(H11G), C9H(H9H), C11H(H11H)
6.b Secondary CH2 refined with riding coordinates:
C10A(H10A,H10B), C10B(H10C,H10D), C10C(H10E,H10F), C10D(H10G,H10H), C10E(H10I,
H10J), C10F(H10K,H10L), C10Z(H10M,H10N), C10G(H10O,H10P),

C10H(H10Q,H10R)
6.c Aromatic/amide H refined with riding coordinates:
N1A(H1A), C2A(H2A), C3A(H3A), C6A(H6A), C13A(H13A), C14A(H14A), N1B(H1B),
C2B(H2B), C3B(H3B), C6B(H6B), C13B(H13B), C14B(H14B), N1C(H1C), C2C(H2C),
C3C(H3C), C6C(H6C), C13C(H13C), C14C(H14C), N1D(H1D), C2D(H2D), C3D(H3D),
C6D(H6D), C13D(H13D), C14D(H14D), N1E(H1E), C2E(H2E), C3E(H3E), C6E(H6E),
C13E(H13E), C14E(H14E), N1F(H1F), C2F(H2F), C3F(H3F), C6F(H6F), C13F(H13F),
C14F(H14F), N1Z(H1Z), C2Z(H2Z), C3Z(H3Z), C6Z(H6Z), C13Z(H13Z), C14Z(H14Z),
N1G(H1G), C2G(H2G), C3G(H3G), C6G(H6G), C13G(H13G), C14G(H14G), N1H(H1H),
C2H(H2H), C3H(H3H), C6H(H6H), C13H(H13H), C14H(H14H)
6.d Idealised Me refined as rotating group:
C15A(H15A,H15B,H15C), C16A(H16A,H16B,H16C), C15B(H15D,H15E,H15F), C16B(H16D,
H16E,H16F), C15C(H15G,H15H,H15I), C16C(H16G,H16H,H16I), C15D(H15J,H15K,H15L),
C16D(H16J,H16K,H16L), C15E(H15M,H15N,H15O), C16E(H16M,H16N,H16O), C15F(H15P,
H15Q,H15R), C16F(H16P,H16Q,H16R), C15Z(H15S,H15T,H15U), C16Z(H16S,H16T,H16U),
C15G(H15V,H15W,H15X), C16G(H16V,H16W,H16X), C15H(H15Y,H,HA), C16H(H16Y,HB,HC)

This report has been created with Olex2, compiled on 2015.01.26 svn.r3151 for OlexSys. Please let us know if there are any errors or if you would like to have additional features.
